# Supplementary figures and images for: Highly multiplexed quantitative PCR-based platform for evaluation of chicken immune responses
Source: PLoS One. 2019 Dec 3;14(12):e0225658. doi: 10.1371/journal.pone.0225658 (PMC6890255; doi:10.1371/journal.pone.0225658)

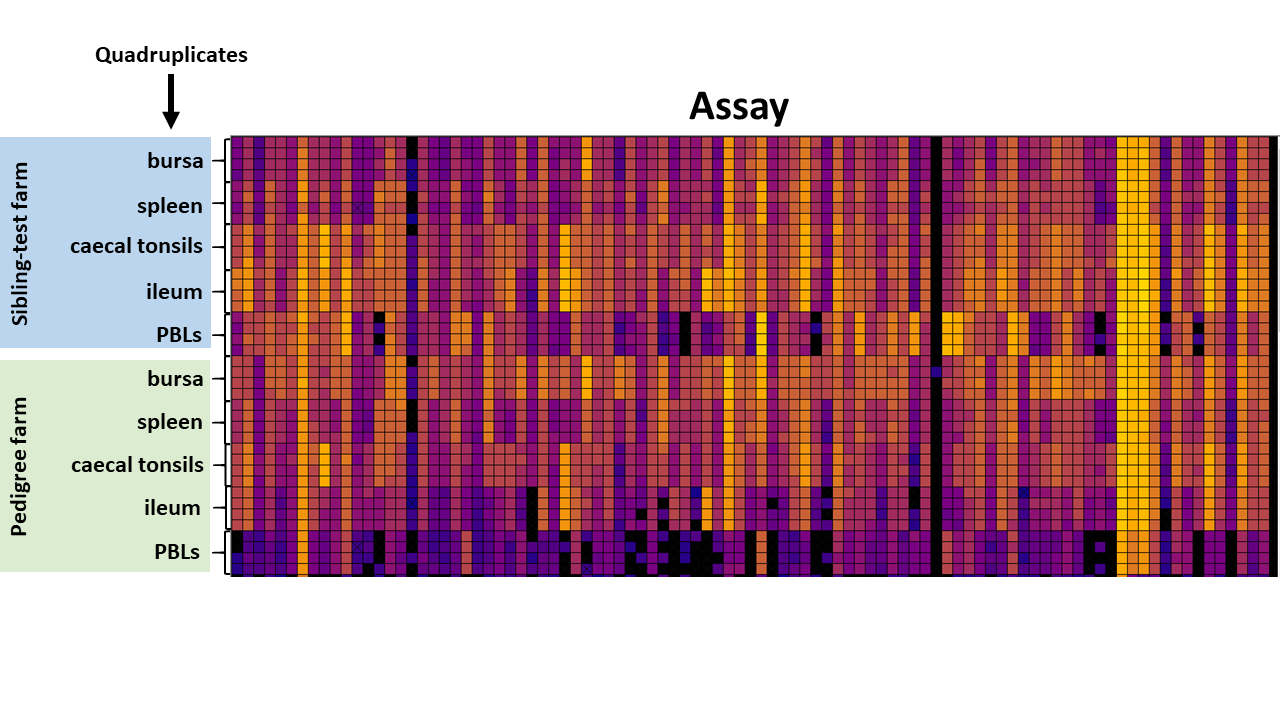

Supplement: S1 Fig — The individual assays on the x axis and selected individual samples from both farms in quadruplicates on y axis. (TIF) [file pone.0225658.s006.tif]
