# Supplementary material for: Highly multiplexed quantitative PCR-based platform for evaluation of chicken immune responses
Source: PLoS One. 2019 Dec 3;14(12):e0225658. doi: 10.1371/journal.pone.0225658 (PMC6890255; doi:10.1371/journal.pone.0225658)
Supplement: S1 File — (DOCX) [file pone.0225658.s007.docx]

**Gene sequences**

ABCG2

ABCG2 : ATGGGAACTGCTCAAAACAACAGTCACCTCAGAAACCCTCATGCGGTTGAGTTTGACCTGTGTAACAAGG : 70
FWD : ATGGGAACTGCTCAAAACAACAGTCACCTCAGAAACCCTCATGCGGTTGAGTTTGACCTGTGTAACAAGG : 70

ABCG2 : ATGTGATGGCGGACACATTTGATCACAGTGTCATTTCTGTTGGAGAAGAGGAAGGAGCAGACAGTTTCCA : 140
FWD : ATGTGATGGCGGACACATTTGATCACAGTGTCATTTCTGTTGGAGAAGAGGAAGGAGCAGACAGTTTCCA : 140

ABCG2 : ACGATCTCTTCCAACACGAGATTCTCTCCGATCCCCTCGAGGCTCCATTGTGAGTTTCCATAACATCCAG : 210
FWD : ACGATCTCTTCCAACACGAGATTCTCTCCGATCCCCTCGAGGCTCCATTGTGAGTTTCCATAACATCCAG : 210

ABCG2 : TACTCCGTTAAGCAGTCCAGTGGATTCCTATGTAGACGGAAGACTGTGGAAAAGAAGATCCTTCATAATG : 280
FWD : TACTCCGTTAAGCAGTCCAGTGGATTCCTATGTAGACGGAAGACTGTGGAAAAGAAGATCCTTCATAATG : 280

ABCG2 : TTTATGGCATTATGAAGCCAGGCTTGAATGCTATCCTGGGGCCAACAGGGAGCGGCAAATCTTCTCTGCT : 350
FWD : TTTATGGCATTATGAAGCCAGGCTTGAATGCTATCCTGGGGCCAACAGGGAGCGGCAAATCTTCTCTGCT : 350

ABCG2 : AGATGTGCTGGCTGCCAGAAAGGACCCAGCAGGCCTGTCTGGAGAAGTGCTCATAGATGGCATCCCACAA : 420
FWD : AGATGTGCTGGCTGCCAGAAAGGACCCAGCAGGCCTGTCTGGAGAAGTGCTCATAGATGGCATCCCACAA : 420

ABCG2 : CCTCCAAACTTCAAGTGCATCTCAGGATATGTTGTGCAGGATGATGTTGTCATGGGCACAATGACAGTGA : 490
FWD : CCTCCAAACTTCAAGTGCATCTCAGGATATGTTGTGCAGGATGATGTTGTCATGGGCACAATGACAGTGA : 490

ABCG2 : GGGAGAACCTGCACTTCTCTGCTGCCCTCCGGCTCCCCAGCTCCATCAGCTTTCAAGAGAAGGAAGAACG : 560
FWD : GGGAGAACCTGCACTTCTCTGCTGCCCTCCGGCTCCCCAGCTCCATCAGCTTTCAAGAGAAGGAAGAACG : 560

ABCG2 : AGTCACACAGATAATTGGCGAGCTGGGATTAAGCAAAGTGGCTGATGCTAAGGTAGGAACTGAATTGATC : 630
FWD : AGTCACACAGATAATTGGCGAGCTGGGATTAAGCAAAGTGGCTGATGCTAAGGTAGGAACTGAATTGATC : 630

ABCG2 : CGTGGAGTGTCTGGAGGGGAACGGAAAAGAACCAACATCGGGATGGAGCTCATCACAGAGCCACCAGTGC : 700
FWD : CGTGGAGTGTCTGGAGGGGAACGGAAAAGAACCAACATCGGGATGGAGCTCATCACAGAGCCACCAGTGC : 700

ABCG2 : TTTTTCTGGATGAGCCAACAACAGGCCTTGATGCCAGCACGGCCAATGCTGTCCTCATCCTCTTGAAGAA : 770
FWD : TTTTTCTGGATGAGCCAACAACAGGCCTTGATGCCAGCACGGCCAATGCTGTCCTCATCCTCTTGAAGAA : 770

ABCG2 : GCTCTCTAGAAGAGGCCGAACCATCATATTTTCCATCCATCAGCCCCGCTATTCCATATTCAAGCTGTTT : 840
FWD : GCTCTCTAGAAGAGGCCGAACCATCATATTTTCCATCCATCAGCCCCGCTATTCCATATTCAAGCTGTTT : 840

ABCG2 : GACAGTCTGACATTACTAGCTTTGGGTAAAGTGCTGTACCATGGTCCTGCAAAACAGGCCCTGGAGTATT : 910
FWD : GACAGTCTGACATTACTAGCTTTGGGTAAAGTGCTGTACCATGGTCCTGC.................... : 890

ABCG2 : TTAGTTCTATTGGATATGAATGTGAGCCATTTAACAACCCAGCTGACTTCTTCCTTGATATCATAAATGG : 980
REV : ...................................................................... : -

ABCG2 : TGATTCAACTGCTGTGGCAGCAAGCAAGGAAGATCACAAGCCTGCAGACACAGGAAAAGAAGCGAGCAAT : 1050
REV : ...................................................................... : -

ABCG2 : GAAAATGGAAGAGTAGAAGAGAACATGTCCATCAGTGTGGTAGATACACTACACCAGAAGTATCTCAACT : 1120
REV : ...................................................................... : -

ABCG2 : CCAGCCTATATGAGAGCACAAAGGAAGCACTGGGGAAAGTGGAGCGTGAACAGGGAAGGAAGAAGAAAGT : 1190
REV : ...................................................................... : -

ABCG2 : ATCCAAGAAGGGGCACGAGATTACCTATGCAAATGGGTTCTTCACACAGCTCTATTGGGTGTCCAAGCGT : 1260
REV : ..................................GGGTTCTTCACACAGCTCTATTGGGTGTCCAAGCGT : 36

ABCG2 : TCCCTGAAAAACCTCATTAGGAACCCACAAGCCTCGATTGCACAGATTGCAGTGACTGTAATTCTAGCCT : 1330
REV : TCCCTGAAAAACCTCATTAGGAACCCACAAGCCTCGATTGCACAGATTGCAGTGACTGTAATTCTAGCCT : 106

ABCG2 : TGGTTGTGGGGGCCATTTTTTTTGGAGTAAAATTGGACGAAAGTGGCATTCAGAATCGGGTTGGATCCTT : 1400
REV : TGGTTGTGGGGGCCATTTTTTT.GGAGTAAAATTGGACGAAAGTGGCATTCAGAATCGGGTTGGATCCTT : 175

ABCG2 : GTTCTTTGTCACCACAAACCAGTGTTTTTCCAGTGTCTCTGCAATTGAGCTGTTCATCAGAGATAAAAAA : 1470
REV : GTTCTTTGTCACCACAAACCAGTGTTTTTCCAGTGTCTCTGCAATTGAGCTGTTCATCAGAGATAAAAAA : 245

ABCG2 : CTCTTTGTCCATCAGTACACCAGCGGATATTATCGCGTGTCTGCCTACTTCCTGGCCTTGATGTTAGGAG : 1540
REV : CTCTTTGTCCATCAGTACACCAGCGGATATTATCGCGTGTCTGCCTACTTCCTGGCCTTGATGTTAGGAG : 315

ABCG2 : ATCTACTGCCCATGAGAACTGCTCCAGCCATCATATTCTCATGCATCACTTACTGGATGATTGGATTTCA : 1610
REV : ATCTACTGCCCATGAGAACTGCTCCAGCCATCATATTCTCATGCATCACTTACTGGATGATTGGATTTCA : 385

ABCG2 : AGCTATAGCAGGCCGATTCTTCTTCTTCATGCTGGCCCTGGTAATGGTGTCCTACACTGCTACAGCCATG : 1680
REV : AGCTATAGCAGGCCGATTCTTCTTCTTCATGCTGGCCCTGGTAATGGTGTCCTACACTGCTACAGCCATG : 455

ABCG2 : TCTCTGGCTATCAGTGCTGGGATGGAAGTGGTGGCTGTGGCCAATCTGCTCATCACTATTTGTTTTGTCC : 1750
REV : TCTCTGGCTATCAGTGCTGGGATGGAAGTGGTGGCTGTGGCCAATCTGCTCATCACTATTTGTTTTGTCC : 525

ABCG2 : TGATGCTTATCTTTTCTGGCCTCCTTGTAAACCTCCCTTCTGTAATGGGCTGGCTTAATTGGCTCAAGTA : 1820
REV : TGATGCTTATCTTTTCTGGCCTCCTTGTAAACCTCCCTTCTGTAATGGGCTGGCTTAATTGGCTCAAGTA : 595

ABCG2 : CTTCAGCATCCCTCGATACGGCCTTACTGCTCTTCAAGTGAATGAGTTCAGAGATCTCTACTTTTGTGGT : 1890
REV : CTTCAGCATCCCTCGATACGGCCTTACTGCTCTTCAAGTGAATGAGTTCAGAGATCTCTACTTTTGTGGT : 665

ABCG2 : GACAAACCAAACGTTACAGTGTCTGTGGGAAATGCAACCGCTTGTCCACCAGTTACTTCAGGAGTAAGGT : 1960
REV : GACAAACCAAACGTTACAGTGTCTGTGGGAAATGCAACCGCTTGTCCACCAGTTACTTCAGGAGTAAGGT : 735

ABCG2 : GCTCTGGTGAAGATTACCTGATCAGCCAAGGAATCGCACCTACTAACATGGCAATGTGGGAGAACATAGT : 2030
REV : GCTCTGGTGAAGATTACCTGATCAGCCAAGGAATCGCACCTACTAACATGGCAATGTGGGAGAACATAGT : 805

ABCG2 : GGCTCTCTTATGCATGACTGTTATCTTCCTTCTCATTGCCTATCTCAAACTCCGGTTTATGAGGAAGTTC : 2100
REV : GGCTCTCTTATGCATGACTGTTATCTTCCTTCTCATTGCCTATCTCAAACTCCGGTTTATGAGGAAGTTC : 875

ABCG2 : ACATAA : 2106
REV : ACATAA : 881

ATF
ATF : GAGGAACTTAGATTCGCCATCCAAAACAAACGTCAGTCCCACAGGATGTCTTCTACTTTGGATACGGTGA : 70
FWD : GAGGAACTTAGATTCGCCATCCAAAACAAACGTCAGTCCCACAGGATGTCTTCTACTTTGGATACGGTGA : 70

ATF : CAGTGTCCGAAAGGCCGATGGAAACATCGATCATGAAAACGGAGTTTTCTCCTGAAGAGGATGAAAGAAA : 140
FWD : CAGTGTCCGAAAGGCCGATGGAAACATCGATCATGAAAACGGAGTTTTCTCCTGAAGAGGATGAAAGAAA : 140

ATF : AAAGCGAAGAAGGGAAAGGAACAAAATTGCTGCTGCAAAGTGCCGAAACAAAAAGAAGGAAAAAACAGAA : 210
FWD : AAAGCGAAGAAGGGAAAGGAACAAAATTGCTGCTGCAAAGTGCCGAAACAAAAAGAAGGAAAAAACAGAA : 210

ATF : TGTTTGCAGAAAGAATCAGAAAAGCTGGAGACTATCAATGCGGAATTAAAAGCCCAGATTGAAGAGCTAA : 280
FWD : TGTTTGCAGAAAGAATCAGAAAAGCTGGAGACTATCAATGCGGAATTAAAAGCCCAGATTGAAGAGCTAA : 280

ATF : AGAATGAGAAGCAGCATTTGATATACATGCTAAATCTTCACAGGCCCACCTGTATAGTCCGGGCACAAAA : 350
FWD : AGAATGAGAAGCAGCATTTGATATACATGCTAAATCTTCACAGGCCCACCTGTATAGTCCGGGCACAAAA : 350

ATF : TGGAAGGACACCTGAAGATGAAAGGAATCTTTTTATTCAACAGATCAAAGAAGGAACATTACAAGGTTAA : 420
FWD : TGGAAGGACACCTGAAGATGAAAGGAATCTTTTTATTCAACAGATCAAAGAAGGAACATTACAAGGTTAA : 420

BATF3
BATF3 : GAGCCACGAAGAAGACGATAAGAAGGTAAGGAGGAGAGAGAAGAACCGAGTTGCTGCGCAGAGAAGTCGG : 70
FWD : GAGCCACGAAGAAGACGATAAGAAGGTAAGGAGGAGAGAGAAGAACCGAGTTGCTGCGCAGAGAAGTCGG : 70
REV : GAGCCACGAAGAAGACGATAAGAAGGTAAGGAGGAGAGAGAAGAACCGAGTTGCTGCGCAGAGAAGTCGG : 70

BATF3 : AAGAAACAAACGCAGAAAGCGGACAAACTTCACGAGGAATATGAATCTCTTGAGCAAGAAAATACCTCCC : 140
FWD : AAGAAACAAACGCAGAAAGCGGACAAACTTCACGAGGAATATGAATCTCTTGAGCAAGAAAATACCTCCC : 140
REV : AAGAAACAAACGCAGAAAGCGGACAAACTTCACGAGGAATATGAATCTCTTGAGCAAGAAAATACCTCCC : 140

BATF3 : TGAAAAAAGAAATCGGAAAGCTAACAGATGAAATGAAACACTTGAGTGAAGTGTTGAAGGATCACGAAAA : 210
FWD : TGAAAAAAGAAATCGGAAAGCTAACAGATGAAATGAAACACTTGAGTGAAGTGTTGAAGGATCACGAAAA : 210
REV : TGAAAAAAGAAATCGGAAAGCTAACAGATGAAATGAAACACTTGAGTGAAGTGTTGAAGGATCACGAAAA : 210

BATF3 : GATCTGTCCACTATTGCACTGCACCATGAACTTTGTGACCATACCAAGGCCTGATGCACTCAGCAGCTGC : 280
FWD : GATCTGTCCACTATTGCACTGCACCATGAACTTTGTGACCATACCAAGGCCTGATGCACTCAGCAGCTGC : 280
REV : GATCTGTCCACTATTGCACTGCACCATGAACTTTGTGACCATACCAAGGCCTGATGCACTCAGCAGCTGC : 280

BATF3 : CTGCCCAGATGA : 292
FWD : CTGCCCAGATGA : 292
REV : CTGCCCAGATGA : 292

BCL2A1
BCL2A1 : ATGGAAACTGCTGAGTTCTATTACGTTTATTATTTAGCTCAAGATTATCTGCAGTATGTGCTTCAGGAAT : 70
FWD : ATGGAAACTGCTGAGTTCTATTACGTTTATTATTTAGCTCAAGATTATCTGCAGTATGTGCTTCAGGAAT : 70

BCL2A1 : CACATCTCGGACCAGCCCAAACCAGAGTTGCTCATGTCTTGCGAAACATTGCATCTTCACTCCAAGATCA : 140
FWD : CACATCTCGGACCAGCCCAAACCAGAGTTGCTCATGTCTTGCGAAACATTGCATCTTCACTCCAAGATCA : 140

BCL2A1 : GACAGAGGAGGCTCTCAGACCCTTCTTGGACAGGATCGATATTACCTCCGTAGATGTTGCCAAGAGAATT : 210
FWD : GACAGAGGAGGCTCTCAGACCCTTCTTGGACAGGATCGATATTACCTCCGTAGATGTTGCCAAGAGAATT : 210

BCL2A1 : TTCAATGGAGTCATGGAAGAAAAATTTGCTGATGGAAATACTAACTGGGGACGAATTATGACCATATTTA : 280
FWD : TTCAATGGAGTCATGGAAGAAAAATTTGCTGATGGGAATACTAACTGGGGACGAATTATGACCATATTTA : 280

BCL2A1 : CTTTTGGAGGTCTTCTCACCAAGAAGCTTCAAGAGCACGGAGTTCAGCTCACTGGAGAGGAGAAGGAGAA : 350
FWD : CTTTTGGAGGTCTTCTCACCAAGAAGCTTCAAGAGCACGGAGTTCAGCTCACTGGAGAGGAGAAGGAGAA : 350

BCL2A1 : GATTTCTTATTTCATCACAGAGTACATCATAAATAACAAAGCCGCATGGATAGATGCAAACGGTGGCTGG : 420
FWD : GATTTCTTATTTCATCACAGAGTACATCATAAATAACAAAGCCGCATGGATAGATGCAAACGGTGGCTGG : 420

BCL2A1 : GAAAACGGTTTCCTAACGAAGTTTGAAAGAAGATCACCCCTATCTTTCTCTACAATTACAGACATATTTG : 490
FWD : GAAAACGGTTTCCTAACGAAGTTTGAAAGAAGATCACCCCTATCTTTCTCTACAATTACAGACATATTTG : 490

BCL2A1 : CAGCTGTTCTTTCCTTGTTCAGAGAGTACCACTGA : 525
FWD : CAGCTGTTCTTTCCTTGTTCAGAGAGTACCACTGA : 525

C3ORF52
FWD : ATGAGCTGGCTCCGTGCTTGCTACAGAGACAGAGGTCCTCGCACAGAAACAGAAGCTATGAAAAGGCAGA : 70
C3ORF52 : ATGAGCTGGCTCCGTGCTTGCTACAGAGACAGAGGTCCTCGCACAGAAACAGAAGCTATGAAAAGGCAGA : 70

FWD : GCTCTGGTGGGGAGCACGAGGTTATTGAATTGCCAGAAGTTAATGGGGAGGAAAGTACAGCTGATCATAA : 140
C3ORF52 : GCTCTGGTGGGGAGCACGAGGTTATTGAATTGCCAGAAGTTAATGGGGAGGAAAGTACAGCTGATCATAA : 140

FWD : GAAGCCTCTAAATCCTCAAGTACCTGCAACGAGCAAGGAGAGAGATCAGTGGAAATCATGCAGGAAGATC : 210
C3ORF52 : GAAGCCTCTAAATCCTCAAGTACCTGCAACGAGCAAGGAGAGAGATCAGTGGAAATCATGCAGGAAGATC : 210

FWD : ATTTTCTGGAAGTGTAAACTATGGATGGTTTTAACTACAATTTTTGTTGTTTTGTTCCTGGTCATTCTCA : 280
C3ORF52 : ATTTTCTGGAAGTGTAAACTATGGATGGTTTTAACTACAATTTTTGTTGTTTTGTTCCTGGTCATTCTCA : 280

FWD : TCAGTCTAGCTCTATATTCTAATGTTTACACAGATGAAGATGATTATTGGTATACAGATGAACTGCTACA : 350
C3ORF52 : TCAGTCTAGCTCTATATTCTAATGTTTACACAGATGAAGATGATTATTGGTATACAGATGAACTGCTACA : 350

FWD : GAATTATCACAATTTTTCTGGAAAGTTCAACTTACTGTGTGGTCTTCCACACGTTTTCTCTGAAGACATT : 420
C3ORF52 : GAATTATCACAATTTTTCTGGAAAGTTCAACTTACTGTGTGGTCTTCCACACGTTTTCTCTGAAGACATT : 420

FWD : ATTAAGAGGATAACAGATGTCTACAGCTCATCTCCAGCTCTCGGACGCTACTTTAGGTCTGCTAAAGTGG : 490
C3ORF52 : ATTAAGAGGATAACAGATGTCTACAGCTCATCTCCAGCTCTTGGACGCTACTTTAGGTCTGCTAAAGTGG : 490

FWD : ATTATTTCAGTAATGAAAGCTCCACTGTATTTTACCAACTAGAGTTCTTTGTACCGCCGTCAACAGAGGG : 560
C3ORF52 : ATTATTTCAGTAATGAAAGCTCCACTGTATTTTACCAACTAGAGTTCTTTGTACCGCCATCAACAGAGGG : 560

FWD : GTTTATGGAGAATGTAATGAACCCAGACTTTATAAGGAATGTTCTACTTCAAAATATTTACGATGAAGAA : 630
C3ORF52 : GTTTATGGAGAATGTAATGAACCCAGACTTTATAAGGAATGTTCTACTTCAAAATATTTACGATGAAGAA : 630

FWD : GATACTTCTAATCCCGGGACATCTGAATGTACCAGGTTAAAGCTTGACCCAGTTTCTCTCACATCAACAT : 700
C3ORF52 : GATACTTCTAATCCCGGGACATCTGAATGTACCAGGTTAAAGCTTGACCCAGTTTCTCTCACATCAACAT : 700

FWD : G..... : 701
C3ORF52 : G..... : 701

CD40

CD40 : ATGGGGCGGCTCGGGCTGCTGGGACTCCTCTGCGCGCTGCTCCTGGGCTGTGGGCAGCCTGGTGATGCTG : 70
FWD : ATGGGGCGGCTCGGGCTGCTGGGACTCCTCTGCGCGCTGCTCCTGGGCTGTGGGCAGCCTGGTGATGCTG : 70
REV : ...................................................................... : -

CD40 : TGAATTGCTCTGACAAGCAGTATGAGCACAAGGGCAGATGCTGCAACCGATGCCAGCCAGGGAAGAAGCT : 140
FWD : TGAATTGCTCTGACAAGCAGTATGAGCACAAGGGCAGATGCTGCAACCGATGCCAGCCAGGGAAGAAGCT : 140
REV : ...................................................................... : -

CD40 : GGCCTCTGAATGCAACGACACAGAAGACTCTGTCTGCACACCCTGTGAGAATGGTCAGTACCAGCATAGC : 210
FWD : GGCCTCTGAATGCAACGACACAGAAGACTCTGTCTGCACACCCTGTGAGAATGGTCAGTACCAGCAGAGC : 210
REV : ........AATGCAACGACACAGAAGACTCTGTCTGCACACCCTGTGAGAATGGTCAGTACCAGCAGAGC : 62

CD40 : TGGACAAAGGAAAGGCACTGCACGCCCCATGAAATCTGTGAGGACAACGCTGGCCTCATTGTGAAGAGAC : 280
FWD : TGGACAAAGGAAAGGCACTGCACGCCCCATGAAATCTGTGAGGACAACGCTGGCCTCATTGTGAAGAGAC : 280
REV : TGGACAAAGGAAAGGCACTGCACGCCCCATGAAATCTGTGAGGACAACGCTGGCCTCATTGTGAAGAGAC : 132

CD40 : ATGGAAACGCAACGCACAACACTGTGTGCCAGTGCCGGGCCGGCATGCACTGCTCTGATGCCAGCTGCCA : 350
FWD : ATGGAAACGCAACGCACAACACTGTGTGCCAGTGCCGGGCCGGCATGCGCTGCTCTGATGCCAGCTGCCA : 350
REV : ATGGAAACGCAACGCACAACACTGTGTGCCAGTGCCGGGCCGGCATGCGCTGCTCTGATGCCAGCTGCCA : 202

CD40 : GACCTGCGTGGAGAACGAGCCCTGCAAGCAGGGCTTTGGCTTTGTGGCAGCCATGGCTGAAGCCCGGATG : 420
FWD : GACCTGCGTGGAGAACGAGCCCTGCAAGCAGGGCTTTGGCTTTGTGGCAGCCATGGCTGAAGCCCGGATG : 420
REV : GACCTGCGTGGAGAACGAGCCCTGCAAGCAGGGCTTTGGCTTTGTGGCAGCCATGGCTGAAGCCCGGATG : 272

CD40 : ACCTCACCGTGTGAGCCCTGTGCAGAAGGCACCTTCTCCAATGTATCTTCCAAAACTGAGCCATGCCACT : 490
FWD : ACCTCACCGTGTGAGCCCTGTGCAGAAGGCACCTTCTCCAATGTATCTTCCAAAACTGAGCCATGCCACT : 490
REV : ACCTCACCGTGTGAGCCCTGTGCAGAAGGCACCTTCTCCAATGTATCTTCCAAAACTGAGCCATGCCACT : 342

CD40 : TCTGGACAAGCTGTGAGGAAAAGGGGCTTGTGGTGAAGGTGAAAGGGACGAACACTTCCGATGTGATCTG : 560
FWD : TCTGGACAAGCTGTGAGGAAAAGGGGCTTGTGGTGAAGGTGAAAGGGACGAACACTTCCGATGTGATCTG : 560
REV : TCTGGACAAGCTGTGAGGAAAAGGGGCTTGTGGTGAAGGTGAAAGGGACGAACACTTCCGATGTGATCTG : 412

CD40 : TGAGTCAAGCCGGCGCTCATCGCTGTCAGTGCTGATCCCCATCACAGCTGCAGTTGTCACCTGCCTGGTG : 630
FWD : TGAGTCAAGCCGGCGCTCATCGCTGTCAGTGCTGATCCCCATCACAGCTGCAGTTGCCACCTGCCTGGTG : 630
REV : TGAGTCAAGCCGGCGCTCATCGCTGTCAGTGCTGATCCCCATCACAGCTGCAGTTGCCACCTGCCTGGTG : 482

CD40 : GGCATCTGCATCTACTGCCTGGTGCACACAGACCTCAGGCGCCGTGGGCCAAAGCAGGCTGAGGCCGAGG : 700
FWD : GGCATCTGCATCTACTGCCTGGTGCACACAGACCTCAGGCGCCGTGGGCCAAAGCAGGCTGAGGCCGAGG : 700
REV : GGCATCTGCATCTACTGCCTGGTGCACACAGACCTCAGGCGCCGTGGGCCAAAGCAGGCTGAGGCCGAGG : 552

CD40 : CGCCCAGAGAGCTGGTGACGCAGCAGCCCGAGGAGGTGGACTTCCCGGTGCAGGAGACCCTGCTGGGAGG : 770
FWD : CGCCCAGAGAGCTGGTGACGCAGCAGCCCGAGGAGGTGGACTTCCCGGTGCAGGAGACCCTGCTGGGAGG : 770
REV : CGCCCAGAGAGCTGGTGACGCAGCAGCCCGAGGAGGTGGACTTCCCGGTGCAGGAGACCCTGCTGGGAGG : 622

CD40 : GCAGCCCGTGGCGCAGGAGGACGGCAAGGAGAGCCGCATCGCCGAGCAGGAGCAGCTGTGA : 831
FWD : G............................................................ : 771
REV : GCAGCCCGTGGCGCAGGAGGACGGCAAGGAGAGCCGCATCGCCGAGCAGGAGCAGCTGTGA : 683

CD72

CD72 : ATGGCCCAGAGCGTGCTCTACGCCGACCTGAGATTTGCCAAAGGGCCAGGGGGCCACAGCACAACCAGCC : 70
FWD : ATGGCCCAGAGCGTGCTCTACGCCGACCTGAGATTTGCCAAAGGGCCAGGGGGCCACAGCACAACCAGCC : 70
REV : ...................................................................... : -

CD72 : AGGTGCTGGAGGCAGACAGCGTGGATGACACAGACAGCCCCTATGAGAATGTTGTACCGGGACCAGCACC : 140
FWD : AGGTGCTGGAGGCAGACAGCGTGGATGACACAGACAGCCCCTATGAGAATGTTGTACCGGGACCAGCACC : 140
REV : ...................................................................... : -

CD72 : CGTGGGGACGGCAGGGGAAGGGACCCAGCACAGCCCAGGGCACTGGTCCCGACGGCGCTGTGTCCCTGTG : 210
FWD : CGTGGGGACGGCAGGGGAAGGGACCCAGCACAGCCCAGGGCACTGGTCCCGACGGCGCTGTGTCCCTGTG : 210
REV : ...................................................................... : -

CD72 : GGTCTGCTGGCAGCCATGCTGCTGCTGTTGGTGGCCCTGGTGGCTCTGGGGACCTGCTACTGGCAGGTGA : 280
FWD : GGTCTGCTGGCAGCCATGCTGCTGCTGTTGGTGGCCCTGGTGGCTCTGGGGACCTGCTACTGGCAGGTGA : 280
REV : ..................................CCCTGGTGGCTCTGGGGACCTGCTACTGGCAGGTGA : 36

CD72 : CCCACCAGCTGCAGAACGTATCCATGGAGCAGGCCACTGAGCGTGGCCACTTCTCACAGAAGGCACAGGT : 350
FWD : CCCACCAGCTGCAGAACGTATCCATGGAGCAGGCCACTGAGCGTGGCCACTTCTCACAGAAGGCACAGGT : 350
REV : CCCACCAGCTGCAGAACGTATCCATGGAGCAGGCCACTGAGCGTGGCCACTTCTCACAGAAGGCACAGGT : 106

CD72 : GTGGGAGCAGAACCTGGAGCAGACACAGCAGCAGTTGGCATTGGTGCAAGAGGAGCTGCAGCAAGCGTGG : 420
FWD : GTGGGAGCAGAACCTGGAGCAGACACAGCAGCAGTTGGCATTGGTGCAAGAGGAGCTGCAGCAAGCGTGG : 420
REV : GTGGGAGCAGAACCTGGAGCAGACACAGCAGCAGTTGGCATTGGTGCAAGAGGAGCTGCAGCAAGCGTGG : 176

CD72 : CAGGAG.CCCACCACAGCCAGCAGGAGCTGGCCAGGCGGG..GCTGAGCTGGTGCGTGTTTCAGGAGCCC : 487
FWD : CAGGAGGCCCACCACAGCCAGCAGGAGCTGGCCAGGCGGGAGGCTGAGCTGGTGCGTGTTTCAGGAGCCC : 490
REV : CAGGAGGCCCACCACAGCCAGCAGGAGCTGGCCAGGCGGGAGGCTGAGCTGGTGCGTGTTTCAGGAGCCC : 246

CD72 : TGAATGTGACCCAAAAGGAGCTGCAGGATGTGCAGGGGAAGCTCAGTGCCATCAAGGAAGCAGTGAGCAG : 557
FWD : TGAATGTGACCCAAAAGGAGCTGCAGGATGTGCAGGGGAAGCTCAGTGCCATCAAGGAAGCAGTGAGCAG : 560
REV : TGAATGTGACCCAAAAGGAGCTGCAGGATGTGCAGGGGAAGCTCAGTGCCATCAAGGAAGCAGTGAGCAG : 316

CD72 : CCTGCATGTCTGTCTGAACGGAGACTGCTGCCCCTCGGGCTGGCTGCTCTACCATGGCAAGTGCATCTTC : 627
FWD : CCTGCATGTCTGTCTGAACGGAGACTGCTGCCCCTCGGGCTGGCTGCTCTACCATGGCAAGTGCATCTTC : 630
REV : CCTGCATGTCTGTCTGAACGGAGACTGCTGCCCCTCGGGCTGGCTGCTCTACCATGGCAAGTGCATCTTC : 386

CD72 : ATCTCGGCGGCGAAGAAGAGCTGGTGGGGCAGCTACTGGGATTGTGTGGAGAAGCACTCCCGTCTGTTGG : 697
FWD : ATCTCGGCGGCGAAGAAGAGCTGGTGGGACAGCTACTGGGATTGTGTGGAGAAGCACTCCCGTCTGTTGG : 700
REV : ATCTCGGCGGCGAAGAAGAGCTGGTGGGACAGCTACTGGGATTGTGTGGAGAAGCACTCCCGTCTGTTGG : 456

CD72 : TCCAAGGTGAATCGGAGTTGTGGGTGCTGCCGCATTTTCTGCAATCAGACAGTGCCAAGTACTGGATTGG : 767
FWD : TCCAAGGTGAATCGGAGTTGTGGGTGCTGCCGCATTTTCTGCAATCAGACAGTGCCAAGTACTGGATTGG : 770
REV : TCCAAGGTGAATCGGAGTTGTGGGTGCTGCCGCATTTTCTGCAATCAGACAGTGCCAAGTACTGGATTGG : 526

CD72 : AGGAATATACCAATGGCCGTTAGATATTCAGTGGCTGGATAAGAAGCCGCTTAGCCAGGGAGAGTGGTTT : 837
FWD : AGGAATATACCAATGGCCGT.................................................. : 790
REV : AGGAATATACCAATGGCCGTTAGATATTCAGTGGCTGGATAAGAAGCCGCTTAGCCAGGGAGAGTGGTTT : 596

CD72 : GCCTACTGCGCAGCAACAGTCTCTGGGAAGATAGTCCGGAAAAGCTGCAGAGAAGAATATGCATGGATCT : 907
FWD : ...................................................................... : -
REV : GCCTACTGCGCAGCAACAGTCTCTGGGAAGATAGTCCGGAAAAGCTGCAGAGAAGAATATGCATGGATCT : 666

CD72 : GTGAGCAAGCCCCAAATATGAGCAGCGTATCAGAGAGGATCTTTCCTCTGCTGGCCGAAGGGTGA : 972
FWD : ................................................................. : -
REV : GTGAGCAAGCCCCAAATATGAGCAGCGTATCAGAGAGGATCTTTCCTCTGCTGGCCGAAGGGTGA : 731

CD80
CD80 : ATGAAGATGGGGTGCCTGAAGAGATGGCCCCTGAAGAGATGGCTCGGGCTCGGGCTTGGGCTCATCGTGC : 70
REV : ATGAAGATGGGGTGCCTGAAGAGATGGCCCCTGAAGAGATGGCTCGGGCTCGG......GCTCATCGTGC : 64

CD80 : TCCACTGCATCACTCTAGGCTGTGCACAGGAGAAGAAAGTGGCCAAAAGCAAAGTAGGGGAAAAAGTTGG : 140
REV : TCCACTGCATCACTCTAGGCTGTGCACAGGAGAAGAAAGTGGCCAAAAGCAAAGTAGGGGAAAAAGTTGG : 134

CD80 : CCTGCCTTGTTGTTACAAAATTCCCAGCTCGGAGAGCCTGCAAAACTACCGAGTGTATTGGCAGATGAAC : 210
REV : CCTGCCTTGTTGTTACAAAATTCCCAGCTCGGAGAGCCTGCAAAACTACCGAGTGTATTGGCAGATGAAC : 204

CD80 : GTCACGGATGTGGTGCTGGCCTACGCAGGGGAGAAAAAGATCAATGAGCACCCACGCTATGTCAACCGGA : 280
FWD : GTCACGGATGTGGTGCTGGCCTACGCAGGGGAGAAAAAGATCAATGAGCACTCACGCTATGTCAACCGGA : 99

CD80 : CAAAGCTGGACTTTGAGAACCTCACCCTGTGGATCTCCAGTGTGGAAATCCTGGACAGCGGCCCTTACCA : 350
FWD : CAAAGCTGGACTTTGAGAACCTCACCCTGTGGATCTCCAGCGTGGAAATCCTGGACAGCGGCCCTTACCA : 169

CD80 : ATGTATCGTTCAGAGTCTCCAGTCTTCACCAGACAAA.CCTGGATTTCATCTTTTGTGTGGAGAGCCTGT : 419
FWD : ATGTATCGTTCAGAGTCTCCAGTCTTCACCGGACAAAACCTGGATCTCATCTTTTGTGTGGAGAGCCTGT : 239

CD80 : GACCCTCTTTGTTACCGCTGACTTCAGCAAGCCGAACATAGAAAGAGAAGTAACCGCTAGTTCATGTGCA : 489
FWD : GACCCTCTTTGTTACCGCTGACTTCAGCAAGCCGAACATAGAAAGAGAAGTAACCGCTAGTTCATGTGCA : 309

CD80 : TCAACAGAAATGGTGGTAAGATGTTCTTCTCATGGAGGTTTTCCCAAACCCGAAATCCGTGGATTCCTCA : 559
FWD : TCAACAGAAATGGTGGTAAGATGTTCTTCTCATGGAGGTTTTCCCAAACCCGAAATCCGTGGATTCCTCA : 379

CD80 : ACGACGAACGTGTGGTGTTGAATACCACCTGGGAGTCTGAGTCCAGCCTCAGCCCGTACAACGTCACTGG : 629
FWD : ACGACGAACGTGTGGTGTTGAATACCACCTGGGAGTCTGAGTCCAGCCTCAGCCCGTACAACGTCACTGG : 449

CD80 : CACACTGTGGCTCAACATGACCAAAGATAGCAACTTCACTTGCTTCGTTGAATATGATGGCCTTCTCAGG : 699
FWD : CACACTGTGGCTCAACATGACCAAAGATAGCAACTTCACTTGCTTCGTTGAATATGATGGCCTTCTCAGG : 519

CD80 : TCCACCAGTTTGCTTCTAGCAAAAGCAAATGACTGCATCGTCTCTACTGCACTTCCATCCTATAATGTCA : 769
FWD : TCCACCAGTTTGCTTCTAGCAAAAGCAAATGACTGCATCGTCTCTACTGCACTTCCATCCTATAATGTCA : 589

CD80 : TTACTGCTTCAAGTATCATCATTATCACCTTCGTTTTGGCTGTCACCCTAGCAGTAAAGTACCTCCCAAG : 839
FWD : TTACTGCTTCAAGTATCATCATTATCACCTTCGTTGTGGCTGTCACCCTAGCAGTAAAGTACCTCCCAAG : 659

CD80 : GCACGCCTGTTCCCACTGTTCTAAGAACCAAGTTTCAGCAGAAGACGACGTGAAAGAAAGTATGAACCCA : 909
FWD : GCACGCCTGTCCCCACTGTTCTAAGAACCAAGTTTCAGCAGAAGACGACGTGAAAGAAAGTATGAACCCA : 729

CD80 : CCCCACAGCTGTAAATTGACATGTGAAATGTCATCTCTATGA : 951
FWD : CCCCACAGCTGTAAATTGACATGTGAAATGTCATCTCTATGA : 771

CD83
CD83 : ATGGCTTCAGCAGCCTACACTCTACTCTTCACCCTGTGCAATGTTTGGAGCTTGATCAATGGAGCTGCTG : 70
FWD : ATGGCTTCAGCAGCCTACCCTCTGCTCTTCACCCTGTGCAATGTTTGGAGCTTGATCAATGGAGCTGCTG : 70

CD83 : TGGCTGTCCCAGATGTTGCTGTGACATGCTTTGAAGAAGCTTTGCTGTCCTGTAAAGTTCTTCAGGATTC : 140
FWD : TGGCTGTCCCAGATGTTGCTGTGACATGCTTTGAAGAAGCTTTGCTGTCCTGTAAAGTTCTTCAGGATTC : 140

CD83 : CTCGATCGCCTACCAGGCAGTGTCTTGGCACAAAATGGCTGGAGTTGGCGACAGAATAGCATGGAAAGTC : 210
FWD : CTCGATCGCCTACCAGGCAGTGTCTTGGTACAAAATGGCTGGAGTTGGCGACAGAATAGCATGGAAAGTC : 210

CD83 : CTTGATGTGGAATCTCGTCATCCAAAAGGACTTGGAGGCTCCCTGGAGCTCTCCAACACCACCTTTCAAC : 280
FWD : CTTGATGTGGAATCTCGTCATCCAAAAGGACTTGGAGGCTCCCTGGAGCTCTCCAACACCACCTTTCAAC : 280

CD83 : TGAGGATCAGGAATGCCACCAGCCAGGACAGTGGCACGTACAAGTGCGCTTTGGGGGAACAGAGGGGAGA : 350
FWD : TGAGGATCAGGAATGCCACCAGCCAGGACAGTGGCACGTACAAGTGCGCTTTGGGGGAACAGAGGGGAGA : 350

CD83 : CCACAACCTGAGTGGCATCATCACATTAAAAGTAACAGGTTGCCCTAGAATAGAAGATGAAAAACTGAAA : 420
FWD : CCACAACCTGAGTGGCATCATCACATTAAAAGTAACAGGTTGCCCTAGAATAGAAGATGAAAAACTGAAA : 420

CD83 : AAATACAAGACTGAGCTTTTCATGCTGACTTGCCTCGGGATTTTTTACTTGCTGCTCATCTTCTTTACCT : 490
FWD : AAATACAAGACTGAGCTTTTCATGCTGACTTGCCTCGGGATTTTTTACTTGCTGCTCATCTTCTTTACCT : 490

CD83 : GTACGTGTCTAAGAAAAGAGAGTATGTCTCCCAGTGATAAAAGCAGAAGAGATTCAAAACGCACTCTCAC : 560
FWD : GTACATGTCTAGGAAAAGAGAGTATGTCTCCCAGTGATAAAAGCAGAAGAGATTCAAAACGCACTCTCAC : 560

CD83 : TCTCATCAACGCACATGAAATGACAACACTCCGGGTTTTAAACAGTGGCAGCACGTGCAAAAGTGGACTT : 630
FWD : TCTCATCAACGCACATGAAATGACAACACTCCGGGTTTTAAACAGTGGCAGCACGTGCAAAAGTGGACTT : 630

CD83 : ACTTCAAGTTCTATCTAA : 648
FWD : ACTTCAAGTTCTATCTAA : 648

CXORF21
CXORF21 : ATGCTGTCAGAAGGTTACCTTTACAGAATCGCCTACCTCTGTGAGGACTCTGAGCTCTGCACCAACCAAG : 70
FWD : ATGCTGTCAGAAGGTTACCTTTACAGAATCGCCTACCTCTGTGAGGACTCTGAGCTCTGCACCAACCAAG : 70
REV : ...................................................................... : -

CXORF21 : CAGTGGAGGAAGTGGTGTATGAAATGAGGTCCATTAATTGTTCCTCCAGGGATGAAGCACAAGTAAAAAG : 140
FWD : CAGTGGAGGAAGTGGTGTATGAAATGAGGTCCATTAATTGTTCCTCCAGGGATGAAGCACAAGTAAAAAG : 140
REV : ...................................................................... : -

CXORF21 : CCTCCTTCAGAGATGCAGATCTGCTGGCAAATGCATTTCTGCTGTTCACTCTAGGGGTAGCAAACACAGC : 210
FWD : CCTCCTTCAGAGATGCAGATCTGCTGGCAAATGCATTTCTGCTGTTCACTCTAGGGGTAGCAAACACAGC : 210
REV : ...........................................................GCAAACACAGC : 11

CXORF21 : AGAAGGCAGAAA.GACAATCTCCAACAGTCCGTGCAGAACCCACTGCCTGAAGGGCAGACATCTCCAACT : 279
FWD : AGAAGGCAGAAA.GACAATCTCCAACAGTCCGTGCAGAACCCACTGCCTGAAGGGCAGACATCTCCAACT : 279
REV : AGAAGGCAGAAAAGACAATCTCCAACAGTCCGTGCAGAACCCACTGCCTGAAGGGCAGACATCTCCAACT : 81

CXORF21 : ATGGATGTCTGTGAAGGGCTGGTAAGAAAAGACACCTATCTGGTTCCATCCTCCTGCAAAAGCATTTGCA : 349
FWD : ATGGATGTCTGTGAAGGGCTGGTAAGAAAAGACACCTATCTGGTTCCATCCTCCTGCAAAAGCATTTGCA : 349
REV : ATGGATGTCTGTGAAGGGCTGGTAAGAAAAGACACCTATCTGGTTCCATCCTCCTGCAAAAGCATTTGCA : 151

CXORF21 : AGAACTACAATGATTTGCACATAGCTGGGGACTGCGTGGTGCCTATTAGCTCAGTGGCAACTGATTTTAC : 419
FWD : AGAACTACAATGATTTGCACATAGCTGGGGACTGCGTGGTGCCTATTAGCTCAGTGGCAACTGATTTTAC : 419
REV : AGAACTACAATGATTTGCACATAGCTGGGGACTGCGTGGTGCCTATTAGCTCAGTGGCAACTGATTTTAC : 221

CXORF21 : CTGTGACAGTGGCATAGGCCCCTTCCTGGAGTCCTCAGAGATTCCTCCACCTATGGAGTCCGTGCGGGTC : 489
FWD : CTGTGACAGTGGCATAGGCCCCTTCCTGGAGTCCTCAGAGATTCCTCCACCTATGGAGTCCGTGCGGGTC : 489
REV : CTGTGACAGTGGCATAGGCCCCTTCCTGGAGTCCTCAGAGATTCCTCCACCTATGGAGTCCGTGCGGGTC : 291

CXORF21 : CCCCCCAGCGAGACGGGCCGCAAGCAAGCCCAAGGCTTCTCATCATGCTGGCGTGTGGCGAGCTTGGTGC : 559
FWD : CCCCCCAGCGAGACGGGCCGCAAGCAAGCCCAAGGCTTCTCATCATGCTGGCGTGTGGCGAGCTTGGTGC : 559
REV : CCCCCCAGCGAGACGGGCCGCAAGCAAGCCCAAGGCTTCTCATCATGCTGGCGTGTGGCGAGCTTGGTGC : 361

CXORF21 : CTCACCAGCAGCCCCTCTCCGACTCGGCCCTCAACGACTACCTGGAGCAGAAGCTGATGGAGCTGTACAA : 629
FWD : CTCACCAGCAGCCCCTCTCCGACTCGGCCCTCAACGACTACCTGGAGCAGAAGCTGATGGAGCTGTACAA : 629
REV : CTCACCAGCAGCCCCTCTCCGACTCGGCCCTCAACGACTACCTGGAGCAGAAGCTGATGGAGCTGTACAA : 431

CXORF21 : GCAGTACATCATGGACAGCACAGTCAACAGGGCATCCCCCACTCAGATCCTGGCCTCAGAGCTCATCATG : 699
FWD : GCAGTACATCATGGACAGCACAGTCAACAGGGCATCCCCCACTCAGATCCTGGCCTCAGAGCTAATCATG : 699
REV : GCAGTACATCATGGACAGCACAGTCAACAGGGCATCCCCCACTCAGATCCTGGCCTCAGAGCTAATCATG : 501

CXORF21 : ACTAATGTAGACCAAATCAGCACGCAGATATCACGAGAGAGGAAAATGGAGACCACCAAAGCCAAGGACA : 769
FWD : ACTAATGTAGACCAAATCAGCACGCAGATATCACGAGAGAGGAAAATGGAGACCACCAAAGCCAAGGACA : 769
REV : ACTAATGTAGACCAAATCAGCACGCAGATATCACGAGAGAGGAAAATGGAGACCACCAAAGCCAAGGACA : 571

CXORF21 : TTGTCATCAGCCGCTTCTTGCAAATAGCCAGTGAACAAATATTCTCAGAAATTAGCACGCCCAGTCTGCA : 839
FWD : TTGTCATCAGCCGCTTCTTGCAAATAG........................................... : 796
REV : TTGTCATCAGCCGCTTCTTGCAAATAGCCAGTGAACAAATATTCTCAGAAATTAGCACGCCCAGTCTGCA : 641

CXORF21 : TATTTCTCAGTATAGCAACACTAATGCATAG : 870
FWD : ............................... : -
REV : TATTTCTCAGTATAGCAACACTAATGCATAG : 672

DTX2
DTX2 : ATGGCAGCAGCTCAGGGAGCAGGAAGCAGCTCAGCCGGGCCCCCCGGAGCCCCTGTCTCCATCCCTGGGC : 70
FWD2 : ATGGCAGCAGCTCAGGGAGCAGGAAGCAGCTCACCTGGGCCCCCCGGAGCCCCTGTCTCCATCCCTGGGC : 70

DTX2 : ACAGCAGCTCCACGGCAGTGGTGGTGTGGGAATGGCAGGATGAGTTTGGCCGGTGGAGGCCGTACCGAGG : 140
FWD2 : ACAGCAGCTCCACGGCAGTGGTGGTGTGGGAATGGCAGGATGAGTTTGGCCGGTGGAGGCCGTACCGAGG : 140

DTX2 : GGACGTGTGCAAGTTCATTGAGCAGGGTTTCCAGGCCTCTCAGCAGAAGGGGCGGCGTTCAGGATCAGGG : 210
FWD2 : GGACGTGTGCAAGTTCATTGAGCAGGGTTTCCAGGCCTCTCATCAGAAGGGGCGGCGTTCAGGATCAGGG : 210

DTX2 : CTCGTCAGCAGCAGCATCTCTTTGGGACACGCGGATGCTGGCTTGGCCCCCTATGTCATCGACATCCCAA : 280
FWD2 : CTCGTCAGCAGCAGCATCTCTTTGGGACACGCGGATGCTGGCTTGGCCCCCTATGTCATCGACATCCCAA : 280

DTX2 : GCCTGACACAGTTTCGGCAGGATACAGGGACGATGCGGGCAGTCCGCAGGCACCTCTTTCCCAGGGACTC : 350
FWD2 : GCCTGACACAGTTTCGGCAGGATACAGGGACAATGCGGGCAGTCCGCAGGCACCTCTTTCCCATGGACTC : 350

DTX2 : AGCAGCCGAGCAGGGCATCATCTGGGAGTGGCAGAACGATGAACGTGGCTGGTTCCCCTACGAGATGAAT : 420
FWD2 : ATCAGCCGAGCAGGGCATCATCTGGGAGTGGCAGAATGATGAACGTGGCTGGTTCCCCTACGAGATGAAC : 420

DTX2 : GTCTGTGTGTTCCTGGAGCAAGCCCATGCCTCCAGCCACCAGCGGGTGGACCTCGGACCCTTAGGCTACA : 490
FWD2 : GTCTGTGTGTTCCTGGAGCAAGCCCATGCCTCCAGCCACCAGCGGGTGGACCTCGGACCCTTAGGCTACA : 490

DTX2 : ACTATGAGGTCGACCTGGTGGCTCAAGTGCAGACCAACAAGACCACGAGGTTCTGCCGCAGTGTTCGGAG : 560
FWD2 : ACTATGAGGTCGACCTGGTGGCTCAAGTGCAGACCAACAAGACCACGAGGTTCTGCCGCAGTGTTCGGAG : 560

DTX2 : GCGGGTGGACAATCCATACCCCGTGACGGCCGCCTTGGCTCCCGTTCACACGGGGACGGGTTGCTCGTGC : 630
FWD2 : GCGGGTGGACAATCCATACCCCGTGACGGCCGCCTTGGCTCCCGTTCACACGGGGACGGGTTGCTCGTGT : 630

DTX2 : CAGCAGTGCTGGCTGAACGGGGGGACTGGCCCCATCACCACGCGGTACCGACACTCCATGACAAACTTCC : 700
FWD2 : CAGCAGTGCTG........................................................... : 641

DTX2 : CAGCAGTGCTGGCTGAACGGGGGGACTGGCCCCATCACCACGCGGTACCGACACTCCATGACAAACTTCC : 700
REV : ...................................................................... : -
REV2 : .......................GACTGGCCCCATCACCACGCGGTACCGACACTCCATGACAAACTTCC : 47

DTX2 : CCAACTCCTCCGCTGCACCTCAGGTTTCCGGTAGGACGACATCAGTGAGTTCTTCCGGCGTCGGCTTTGT : 770
REV : ...................................................................... : -
REV2 : CCAACTCCTCCGCTGCACCTCAGGTTTCCGGTAGGACGACATCAGTGAGTTCTTCCGGCGTCGGCTTTGT : 117

DTX2 : GCCCTATAACAAACCCGCTTTGTCTGGAGCGAGGTCAGCACCACGACTCAATGCACAGAGCAGCTGGGCT : 840
REV : ...................................................................... : -
REV2 : GCCCTATAACAAACCCGCTTTGTCTGGAGCGAGGTCAGCACCACGACTCAATGCACAGAGCAGCTGGGCT : 187

DTX2 : TTTCCCCAGGCTGGAGGTGCTGTGGGCCCCAGCACAGGACTGTCCACATCGAATGGAGTCAGTGCTCTAA : 910
REV : ...................................................................... : -
REV2 : TTTCCCCAGGCTGGAGGTGCTGTGGGCCCCAGCACAGGACTGTCCACATCGAATGGAGTCAGTGCTCTAA : 257

DTX2 : ACCTCCCAGTGAAGATGTCCAAGCCCAGCAAACTGAACCAAGCCTTGGCAGCAGCACCGACTGACCCAGA : 980
REV : ...................................................................... : -
REV2 : ACCTCCCAGTGAAGATGTCCAAGCCCAGCAAACTGAACCAAGCCTTGGCAGCAGCACCGACTGACCCAGA : 327

DTX2 : AGCAGTGGTGAGGAAGTACCTGGTGGAGGTGAAGGGTACTCCTGTGGATGAGGACTGTATCATCTGCATG : 1050
REV : ...................................................................... : -
REV2 : AGCAGTGGTGAGGAAGTACCTGGTGGAGGTGAAGGGCACTCCTGCGGATGAGGACTGTATCATCTGCATG : 397

DTX2 : GAGAAGCTGGCCTCTCCTTCAGGTTACAGTGACACTTGTGAATGCAGAACAATCAAGCCAGAGATGGTCG : 1120
REV : ......CTGGCCTCTCCTTCAGGTTACAGTGACACTTGTGAATGCAGAACAATCAAGCCAGAGATGGTCG : 64
REV2 : GAGAAGCTGGCCTCTCCTTCAGGTTACAGTGACACTTGTGAATGCAGAACAATCAAGCCAGAGATGGTCG : 467

DTX2 : GTCGCCTGACAAACTGCCAGCACTCCTTCCACATGCTCTGTGTGCTGGCCATGTACTCCAATGGAAACAA : 1190
REV : GTCGCCTGACAAACTGCCAGCACTCCTTCCACATGCTCTGTGTGCTGGCCATGTACTCCAATGGAAACAA : 134
REV2 : GTCGCCTGACAAACTGCCAGCACTCCTTCCACATGCTCTGTGTGCTGGCCATGTACTCCAATGGAAACAA : 537

DTX2 : GGATGGCAGCTTGCAGTGCCCCTCCTGTAAGACCATCTATGGAGAGAAAACTGGTACTCAGCCCAAAGGA : 1260
REV : GGATGGCAGCTTGCAGTGCCCCTCCTGTAAGACCATCTACGGAGAGAAAACTGGTACTCAGCCCAAAGGA : 204
REV2 : GGATGGCAGCTTGCAGTGCCCCTCCTGTAAGACCATCTACGGAGAGAAAACTGGTACTCAGCCCAAAGGA : 607

DTX2 : AAAATGGAGGTCTCCACTTTCCCTCAATCCCTCCCTGGTCACAGGGACTGTGGGACAATCCAGATCGTGT : 1330
REV : AAAATGGAGGTCTCCACTTTCCCTCAATCCCTCCCTGGTCACAGGGACTGTGGGACAATTCAGATCGTGT : 274
REV2 : AAAATGGAGGTCTCCACTTTCCCTCAATCCCTCCCTGGTCACAGGGACTGTGGGACAATTCAGATCGTGT : 677

DTX2 : ACCACATCAGCAGAGGCATTCAGGGCCCTGAGCACCCCAACCCAGGGATGCCTTACACAGCAAGAGGCTT : 1400
REV : ACCACATCAGCAGAGGCATTCAGGGCCCTGAGCACCCCAACCCAGGGATGCCTTACACAGCAAGAGGCTT : 344
REV2 : ACCACATCAGCAGAGGCATTCAGGGCCCTGAGCACCCCAACCCAGGGATGCCTTACACAGCAAGAGGCA. : 746

DTX2 : TCCTCGCTATTGCTACCTACCAGACAATGAGAAGGGCAGAAAGGTCCTGGAGCTCCTGAAGGTGGCCTGG : 1470
REV : TCCTCGCTATTGCTGCCTACCAGACAATGAGAAGGGCAGAAAGGTCCTGGAGCTCCTGAAGGTGGCCTGG : 414
REV2 : ...................................................................... : -

DTX2 : AACAG : 1475
REV : AACAG : 419

REV2 : ..... : -

EAF2
EAF2 : ATGAACGGGATGGCCCCGCCGCTCTTCGAGCCCAAGGAGCGGGTGCTGCAGCTGGGCGAGACCTTCGAGA : 70
FWD : ATGAACGGGATGGCCCCGCCGCTCTTCGAGCCCAAGGAGCGGGTGCTGCAGCTGGGCGAGACCTTCGAGA : 70
REV : ...................................................................... : -

EAF2 : AGCAGCCGCGCTGCGCCTTCCACACCGTGCGCTATGACTTTAAGCCTGCATCTATTGATACCGCTTGTGA : 140
FWD : AGCAGCCGCGCTGCGCCTTCCACACCGTGCGCTATGACTTTAAGCCTGCATCTATTGATACCGCTTGTGA : 140
REV : ..................................................................GTGA : 4

EAF2 : AGGAGACCTTGAAGTTGGCAAAGGTGAACAGGTGACGATAACACTGCCGAATATTGAGGGCTCGACTCCA : 210
FWD : AGGAGACCTTGAAGTTGGCAAAGGTGAACAGGTGACGATAACACTGCCGAATATTGAGGGCTCGACTCCA : 210
REV : AGGAGACCTTGAAGTTGGCAAAGGTGAACAGGTGACGATAACACTGCCGAATATTGAGGGCTCGACTCCA : 74

EAF2 : CCAGTGACAGTGTTCAAGGGCTCGAAGAAGCCTTACCTAAAGGAATGTATCTTAATTATCAATCATGACA : 280
FWD : CCAGTGACAGTGTTCAAGGGCTCGAAGAAGCCTTACCTAAAGGAATGTACCTTAATTATCAATCATGACA : 280
REV : CCAGTGACAGTGTTCAAGGGCTCGAAGAAGCCTTACCTAAAGGAATGTACCTTAATTATCAATCATGACA : 144

EAF2 : CTGGAGAATGTCGTCTAGAGAAACTTAGTAGCAACATCACTGTGAAGAAAATCAGAGCTGAAGGAAGCAG : 350
FWD : CTGGAGAATGTCGTCTAGAGAAACTTAGTAGCAACATCACTGTGAAGAAAATCAGAGCTAAAGGAAGCAG : 350
REV : CTGGAGAATGTCGTCTAGAGAAACTTAGTAGCAACATCACTGTGAAGAAAATCAGAGCTAAAGGAAGCAG : 214

EAF2 : TAAGGTTCAGTCTCGTATAGAACAGCAACAGCAGCAAATCCGGAACTCATCTAAGACTCCAAACAACATC : 420
FWD : TAAGGTTCAGTCTCGTATAGAACAGCAACAGCAGCAAATCCGGAACTCATCTAAGACTCCAAACAACATC : 420
REV : TAAGGTTCAGTCTCGTATAGAACAGCAACAGCAGCAAATCCGGAACTCATCTAAGACTCCAAACAACATC : 284

EAF2 : AAAAATTCCCCACCAAAAGACAAGATGTTTCCATCTTCTCCTATGGATGACATTGAACGAGAACTAAAAG : 490
FWD : AAAAATTCCCCACCAAAAGACAAGATGTTTCCATCTTCTCCTATGGATGACATTGAACGAGAACTAAAAG : 490
REV : AAAAATTCCCCACCAAAAGACAAGATGTTTCCATCTTCTCCTATGGATGACATTGAACGAGAACTAAAAG : 354

EAF2 : CAGAAGCCAGCATCATGGATCAGCTGAGTAGCTCTGACAGTTCATCTGACTCCAAAAGTTCTTCCTCCTC : 560
FWD : CAGAAGCCAGCATCATGGATCAGCTGAGTAGCTCTGACAGTTCATCTGACTCCAAAAGTTCTTCCTCCTC : 560
REV : CAGAAGCCAGCATCATGGATCAGCTGAGTAGCTCTGACAGTTCATCTGACTCCAAAAGTTCTTCCTCCTC : 424

EAF2 : TTCATCAAGTAGTGAAAATAGTTCCAGTGATTCTGAAGATGAGGAAGCAAGACCCTCTCTTCCCATGTCA : 630
FWD : TTCATCAAGTAGTGAAAATAGTTCCAGTGATTCTGAAGATGAGGAAGCAAGACCCTCTCTT......... : 621
REV : TTCATCAAGTAGTGAAAATAGTTCCAGTGATTCTGAAGATGAGGAAGCAAGACCCTCTCTTCCCATGTCA : 494

EAF2 : ATGCCATATCTGCAGCCACAGCCTACTCTGTCTGCCATACCACATCAGGCTGTCCCTGACAAAGATGCCA : 700
FWD : ...................................................................... : -
REV : ATGCCATATCTGCAGCCACAGCCTACTCTGTCTGCCATACCACATCAGGCTGTCCCTGACAAAGATGCCA : 564

EAF2 : GTCATAACAGATCCCAGGAGAACAGTGGTCATATGATGAATACACTACGAAGTGACTTGCAGCTGAGTGA : 770
FWD : ...................................................................... : -
REV : GTCATAACAGATCCCAGGAGAACAGTGGTCATATGATGAATACACTACGAAGTGACTTGCAGCTGAGTGA : 634

EAF2 : ATCTGGAAGCGATAGTGATGACTGA : 795
FWD : ......................... : -
REV : ATCTGGAAGCGATAGTGATGACTGA : 659

EDN1
EDN1 : ATGGATTGCAGCCGCCTGTTCCTCCCGCTGCTCGTCGCGCTGTGCCCGGCGCTGCTGCCGGCAGCACCCG : 70
FWD : ...................................................................... : -

EDN1 : GAGCCGAAGTGAACGCCGCATCGCCGCCGTCCCCCGCTGCCGCTTCGCACCGCC.GAGCCCGGCGCTGCT : 139
FWD : ......................................ATGGATTGCAGCCGCCTGTTCCTCCCGCTGCT : 32

EDN1 : CCTGTTCGTCGCTGCTGGACGAGGAGTGCGTGTATTTCTGCCACCTGGATATCATCTGGATCAACACCCC : 209
FWD : CCTGTTCGTCGCTGCTGGACGAGGAGTGCGTGTATTTCTGCCACCTGGATATCATCTGGATCAACACCCC : 102

EDN1 : CGAGAAGACTGTTCCCTATGGTCTTGGAGGCCCTTCTCGGTCCAGAAGATCACTGAAGGACATAATGCCA : 279
FWD : CGAGAAGACTGTTCCCTATGGTCTTGGAGGCCCTTCTCGGTCCAGAAGATCACTGAAGGACATAATGCCA : 172

EDN1 : GAGATGCTTGCTGGAGCCAGCAGCAGATGCCGATGTGCCAGCCAGAGAGACAAGAAATGTCTGAACTTCT : 349
FWD : GAGATGCTTGCTGGAGCCAGCAGCAGATGCCGATGTGCCAGCCAGAGAGACAAGAAATGTCTGAACTTCT : 242

EDN1 : GCCAGACGGGAAAAGATCTCTGGGCTCAGTCCACAGCAGAGAAAACCTCACGTCACCGCAACAAAGCTGG : 419
FWD : GCCAGACGGGAAAAGATCCCTGGGCTCAGTCCACAGCAGAGAAAACCTCACGTCACCGCAACAAAGCTGG : 312

EDN1 : TGGTTGCATTGGACCTAAATGCATGAACCAACAGTTTGTTGACAGCAGGAAAATGAAGAGGCTGGAGGCT : 489
FWD : TGGTTGCATTGGACCTAAATGCATGAACCAACAGTTTGTTGACAGCAGGAAAATGAAGAGGCTGGAGGCT : 382

EDN1 : GTTGGTAACAGTATCAAAGCTTCCTTCAGTATTGCAAAGCTGAAAGCTGAGCTCCAGAAAGGACGGAAGC : 559
FWD : GTTGGTAACAGTATCAAAGCTTCCTTCAGTATTGCAAAGCTGAAAGCTGAGCTCCAGAAAGGACGGAAGC : 452

EDN1 : TGAAACATAACAGGGCAAGCAAAAGGCAAAGCATCTGGAAAAGCCTGAAAACATTCTAG : 618
FWD : TGAAACATAACAGGGCAAGCAAAAGGCAAAGCATCTGGAAAAGCCTGAAAACATTCTAG : 511

EGR1
EGR1 : ATGGCTGCGGCCAAGGCAGAGATGCAGCTCCTGCCTTCGCTGCAGATCTCCGACCCCTTCGGCGTCTTTC : 70
FWD : ATGGCTGCGGCCAAGGCAGAGATGCAGCTCCTGCCTTCGCTGCAGATCTCCGACCCCTTCGGCGTCTTTC : 70

EGR1 : CGCACTCGCCTCCCGCCATGGACGGCCACTATCCCAAACTGGAGGAGATGATGCTGCTCAGTGGCGGGGG : 140
FWD : CGCACTCGCCTCCCGCCATGGACGGCCACTATCCCAAACTGGAGGAGATGATGCTGCTCAGTGGCGGGGG : 140

EGR1 : ACAGCAGTTTCTTGCGCCTTCCGGGGCGCCTGAGAGTGCGGGCTTCGGCGCGGCCGGGGAGCCCAGCGAG : 210
FWD : ACAGCAGTTTCTTGCGCCTTCCGGGGCGCCTGAGAGTGCGGGCTTCGGCGCGGCCGGGGAGCCCAGCGAG : 210

EGR1 : CAACATTTTGAGCACCTTGCGGCAGACACTTTTCCTGAGATCTCCCTGAACAACGAGAAAACCATGCCAG : 280
FWD : CAACATTTTGAGCATCTTGCGGCAGACACTTTTCCTGAGATCTCCCTGAACAACGAGAAAACCATGCCAG : 280

EGR1 : AAACCAGCTATCCCAACCAAACAACACGGCTACCGCCAATTACCTACACGGGGCGCTTCTCCCTAGAGCC : 350
FWD : AAACCAGCTATCCCAACCAAACAACACGGCTACCGCCAATTACCTACACGGGGCGCTTTTCCCTAGAGCC : 350

EGR1 : AGCCCCAAATGGTAGCAACACCTTATGGCCAGAGCCTCTCTTCAGCCTTGTCAGTGGGCTGGTGAGCATG : 420
FWD : AGCCCCAAATGGTAGCAACACCTTATGGCCAGAGCCTCTCTTCAGCCTTGTCAGTGGGCTGGTGAGCATG : 420

EGR1 : GCTAATGCACCTCCCACCTCTACACCCTCTTCATCATCGCCCTCCTCTTCACAGAGCACCCCCCTGAGCT : 490
FWD : GCTAATGCACCTCCCACCTCTACACCCTCTTCATCATCGCCCTCCTCTTCACAGAGCACCCCCCTGAGCT : 490

EGR1 : GTTCTGTCCAAGCCAGTGAGAACAGTCCGATTTATTCGGCTGCACCCACTTTCCCCACTTCCAGCTCTGA : 560
FWD : GTTCTGTCCAAGCCAGTGAGAACAGTCCGATTTATTCGGCTGCACCCACTTTCCCCACTTCCAGCTCTAA : 560

EGR1 : TATTTTCCCTGAACCACAGACCCAGCCGTTTCCCAACCCCTCGGGAGTCCCCATCCAGTATCCACCTCCA : 630
FWD : TATTTTCCCTGAACCACAGACCCAGCCCTTTCCCAACCCCTCGGGAGTCCCCATCCAGTATCCACCTCCA : 630

EGR1 : GCTTATCCGACTGCTAAAACCAACTTCCAGGTGCCAATGATCCCGGATTACCTGTTCCCTCAGCAACAGA : 700
FWD : GCTTATCCGACTGCTAAAACCAACTTCCAGGTGCCAATGATCCCGGATTACCTGTTCCCTCAGCAACAGA : 700

EGR1 : GTGAGCTCAACCTTATCTCAGCTGATCAGAAGCCCTTCTCAGCCCTTGAGACCAGAGCACAGCAGCCTTC : 770
FWD : GTGAGCTCAACCTTATCTCAGCTGATCAGAAGCCCTTCTCAGCCCTTGAGACCAGAGCACAGCAGCCTTC : 770

EGR1 : CCTCACGCCGCTGTCCACTATCAAGGCATTTGCCACACAGACTGGCTCTCAGGAGTTGAAGACCCTCAAC : 840
FWD : CCTCACGCCGCTATCCACTATA................................................ : 792

EGR1 : CCTCACGCCGCTGTCCACTATCAAGGCATTTGCCACACAGACTGGCTCTCAGGAGTTGAAGACCCTCAAC : 840
REV : ...........................ATTTGCCACACAGACTGGCTCTCAGGAGTTGAAGACCCTCAAC : 43

EGR1 : GCTAACTATCAGTCCCAGCTGATCAAGCCCAGCAGGATGAGGAAATACCCTAATCGTCCCAGCAAGACAC : 910
REV : ACTAACTATCAGTCCCAGCTGATCAAGCCCAGCAGGATGAGGAAATACCCTAATCGTCCCAGCAAGACAC : 113

EGR1 : CTCCTCATGAACGGCCCTATGCTTGCCCAGTGGAGTCCTGTGACCGGCGATTTTCACGGTCCGATGAACT : 980
REV : CTCCTCATGAACGGCCCTATGCTTGCCCAGTGGAGTCCTGTGACCGGCGATTTTCACGGTCCGATGAACT : 183

EGR1 : AACTCGTCACATTCGCATCCACACTGGACAGAAACCTTTCCAATGCCGCATTTGCATGCGGAACTTCAGC : 1050
REV : AACTCGTCACATTCGCATCCACACTGGACAGAAACCTTTCCAATGCCGCATTTGCATGCGGAACTTCAGC : 253

EGR1 : AGGAGTGACCACTTGACCACGCACATCCGCACACATACAGGAGAGAAGCCATTTGCATGTGACATTTGTG : 1120
REV : AGGAGTGACCACTTGACCACGCACATCCGCACACATACAGGAGAGAAGCCATTTGCATGTGACATTTGTG : 323

EGR1 : GCAGAAAGTTTGCCAGAAGTGATGAGAGGAAGAGACACACTAAAATCCACCTTAGGCAGAAGGACAAGAA : 1190
REV : GCAGAAAGTTTGCCAGAAGTGATGAGAGGAAGAGACACACTAAA.TCCACCTTAGGCAGAAGGACAAGAA : 392

EGR1 : AGTGGAAAAGGCAGCTTCGGTCTCAGCAACTTCTTCCTCTGTTGCTGCCTACTCATCCTCTGTGGCTACA : 1260
REV : AGTGGAAAAGGCAGCTTCGGTCTCAGCAACTTCTTCCTCTGTTGCTGCCTACTCATCCTCTGTGGCTACA : 462

EGR1 : TCCTACTCTTCGTCCATAGCCACCACTTACCCCTCACCAGTGCGCACAGTGTATTCCTCCCCTGCTTCCT : 1330
REV : TCCTACTCTTCGTCCATAGCCACCACTTACCCCTCATCAGTGCGCACAGTGTATTCCTCCCCTGCTTCCT : 532

EGR1 : CTTCCTATCCCTCCCCTGCACACACGACATTCCCATCCCCATCTATAGCAACCACTTACTCCTCTGGCAC : 1400
REV : CTTCCTATCCCTCCCCTGCACACACGACATTCCCATCCCCATCTATAGCAACCACTTACTCCTCTGGCAC : 602

EGR1 : TGCCACTTTTCAGACCCAAGTGGCCACTTCCTTCCCATCTCCAGGGGTTACCAATAATTTCAGCTCACAG : 1470
REV : TGCCACTTTTCAGACCCAAGTGGCCACTTCCTTCCCATCTCCAGGGGTTACCAATAATTTCAGCTCACAG : 672

EGR1 : GTGACTTCAGCACTTTCAGACATGACATCAACCTTTTCTCCAAGGACAATTGAGATTTGCTAA : 1533
REV : GTGACTTCAGCACTTTCAGACATGACATCAACCTTTTCTCCAAGGACAATTGAGATTTGCTAA : 735

ENSGALG0000002955

ENS2955 : ATGAAGATGTTCAGGTGGAGGTGGAGCAGCATCTCTGTCAGTGTGCGGTCCCGGCTGAGGGCTGCCCTGG : 70
FWD : ATGAAGATGTTCAGGTGGAGGTGGAGCAGCATCTCTGTCAGTGTGCGGTCCCGGCTGAGGGCTGCCCTGG : 70

ENS2955 : CCCTTCTTGTCGTGGGCAATGTGGCCTTTGTGATAGCTCAGGACCTGCCTCAGACCTCCAACAGCCTGGA : 140
FWD : CCCTTCTTGTCGTGGGCAATGTGGCCTTTGTGATAGCTCAGGACCTGCCTCAGACCTCCAACAGCCTGGA : 140

ENS2955 : GGAGGGTGCAGATGTCACTGCATACTGGTGGGGTGAGTGGACCAAGTGGACGGCATGCACGAGGAGCTGT : 210
FWD : GGAGGGTGCAGATGTCACTGCATACTGGTGGGGTGAGTGGACCAAGTGGACGGCGTGCACGAGGAGCTGT : 210

ENS2955 : GGTGGAGGTGTGAAGTCCCAGGAGAGGCACTGCCTGCGGCAGAGACGAAAGTCGGTGAGTGGGCTGGCTA : 280
FWD : GGTGGAGGTGTGAAGTCCCAGGAGAGGCACTGCCTGCGGCAGAGACGAAAGTCGGTGAGTGGGCTGGCTA : 280

ENS2955 : ATAAAACTTGCACTGGAACATCCAAAAGATATCAGCTCTGCAGAGTACAAGAGTGCCCCGCAAACGGAAG : 350
FWD : ATAAAACTTGCACTGGAACATCCAAAAGATATCAGCTCTGCAAAGTACAAGAGTGCCCCGCAAACGGAAG : 350

ENS2955 : GAGCTTTCGAGAGGAGCAGTGTTCATCCTTTAACTCCCATGTGTATAATGGAAGAACGTATCAATGGAAA : 420
FWD : GAGCTTTCGAGAGGAGCAGTGTTCATCCTTTAACTCCCATGTGTATAATGGAAGAACGTATCAATGGAAA : 420

ENS2955 : CCTTTATACCCTGATGACTATGTTCACATTTCTAGCAAGCCCTGTGACCTCCATTGCACCACTGTGGATG : 490
FWD : CCTTTATACCCTGATGACTATGTTCACATTTCTAGCAAGCCCTGTGACCTCCATTGCACCACTGTGGATG : 490

ENS2955 : GTCAGAGACAATTAATGGTTCAGGCTCGGGATGGAACATCCTGCAAATACACTGATTTCCGAGGGGTTTG : 560
FWD : GTCAGAGACAATTAATGGTTCAGGCTCGGGATGGAACATCCTGCAAATACACTGATTTCCGAGGGGTTTG : 560

ENS2955 : CGTGTCTGGAAAATGTGAGCCAATTGGCTGCGATGGCATTCTCTTTTCCACCCACACCTTGGATAAATGT : 630
FWD : CGTGTCTGGAAAAT.....CCAATTGGCTGCGATGGCATTCTCTTTTCCACCCACACCTTGGATAAATGT : 625

ENS2955 : CGTGTCTGGAAAATGTGAGCCAATTGGCTGCGATGGCATTCTCTTTTCCACCCACACCTTGGATAAATGT : 630
REV : CGTGTCTGGAAAAT.....CCAATTGGCTGCGATGGCATTCTCTTTTCCACCCACACCTTGGATAAATGT : 184

ENS2955 : GGTGTTTGCCAAGGAGATGGGAGCAGCTGCACTCATGTGACGGGGAGTTACCGGAAAGGAAACGCTCATC : 700
REV : GGTGTTTGCCAAGGAGATGGGAGCAGCTGCACTCATGTGACGGGGAGTTACCGGAAAGGAAACGCTCATC : 254

ENS2955 : TGGGCTATTCCCTGGTAACGCACATTCCTGCTGGAGCAAGGGATATCCAGATAGTGGAACGGAAAAAATC : 770
REV : TGGGCTATTCCCTGGTAACGCACATTCCTGCTGGAGCAAGGGATATCCAGATAGTGGAACGGAAAAAATC : 324

ENS2955 : TGCAGATGTTCTGGCTGTGGCTGATGAAGCTGGGTACTATTTCTTCAACGGGAATTACAAAGTTGACAGC : 840
REV : TGCAGATGTTCTGGCTGTGGCTGATGAAGCTGGGTACTATTTCTTCAACGGGAATTACAAAGTTGACAGC : 394

ENS2955 : CCAAAGAACTTCAACATTGCAGGCACGGTGTTCAAGTACCGCTGGCCCATGGATGTCTATGAGACTGGCA : 910
REV : CCAAAGAACTTCAACATTGCAGGCACGGTGTTCAAGTACCGCCGGCCCATGGACGTCTATGAGACTGGCA : 464

ENS2955 : TAGAGTATATTGTTGCACAAGGACCAACAAACCAAGGATTGAACATAATGGTCTGGAATCAAAATGGCAA : 980
REV : TAGAGTATATTGTTGCACAAGGACCAACAAACCAAGGATTGAACATAATGGTCTGGAATCAAAATGGCAA : 534

ENS2955 : AAATCCATCCATCACGTTTGAATACACTCTCTTGAGGAAGCCACACTCAAATCTCCAGCCCATCTACTAC : 1050
REV : AAATCCATCCATCACGTTTGAATACACTCTCTTGAGGAAGCCACACTCAAATCTCCAGCCCATCTACTAC : 604

ENS2955 : ACCTTTTCTGAGTCAGATAGCGAAGAAAGCAGGGAGTTTGATGGAGACGTGCCATTGGGTTTTATCCAAC : 1120
REV : ACCTTTTCTGAGTCAGATAGCGAAGAAAGCAGGGAGTTTGATGGAGACGTGCCATTGGGTTTTATCCAAC : 674

ENS2955 : ACAACGCAACCTATTTTGGGAAGATCTCCAGGGAAAGGATAGACTTG : 1167
REV : ACAACGCAACCTATTTTGGGAAGATCTCCAGGGAAAGGATAGACTTG : 721

ENSGALG0000005747

ENSGAL5747 : ATGACTCGCTGCCAGGTGGTTGCCATCCTTCTGCTTTCCTTCCTTCCCAGCACTGCAGCTGGGACTTCGC : 70
FWD : ATGACTCGCTGCCAGGTGGTTGCCATCCTTCTGCTTTCCTTCCTTCCCAGCACTGCAGCTGGGACTTCGC : 70
REV : ...................................................................... : -

ENSGAL5747 : TTCCCACCCTCGTTCCCCGGTCCACGCTGCAGCTGGGCAGAGTGCGGTTCTCGGCACGGCCCGTGCTGGA : 140
FWD : TTCCCACCCTCGTTCCCCGGTCCACGCTGCAGCTGGGCAGAGTGCGGTTCTCGGCACGGCCCGTGCTGGA : 140
REV : ...................................................................... : -

ENSGAL5747 : GTTGGCTACGCGCTGTGACGAGGCGTGTGTCCGGAGGGAGGCCGCCCTGCTCCCCCAGGACTTCCAGGAG : 210
FWD : GTTGGCTACGCGCTGTGACGAGGTGTGTGTCCGGAGGGAGGCCGCCCTGCTCCCCCAGGACTTCCAGGAG : 210
REV : ...................................................................... : -

ENSGAL5747 : TACCTCTTCTATGAGACACTGTATGCCAACGGCACCCGCACCCTCACGACAGTGGAGCTGAGCCCCGGTG : 280
FWD : TACCTCTTCTATGAGACACTGTATGCCAACGGCACCCGCACCCTCACGACAGTGGAGCTGAGCCCCGGTG : 280
REV : ...................................................................... : -

ENSGAL5747 : AGGGCGGCACGGAGAGGAGGTGGCCGAAGAGGCGCACGCGGCGCAAGCGACAGATTTACGGCACGGATGG : 350
FWD : AGGGCGGCACGGAGAGGAGGTGGCCGAAGAGGCGCACGCGGCGCAAGCGACAGATTTACGGCACGGATGG : 350
REV : ...................................................................... : -

ENSGAL5747 : GCGGTTTTCCATCAGCGGGGATCACTTCCTGATGGATTACCCCTTCTCCACCACAGTGAAGATCTCCACG : 420
FWD : GCGGTTTTCCATCAGCGGGGATCACTTCCTGATGGATTACCCCTTCTCCACCACGGTGAAGATCTCCACG : 420
REV : GCGGTTTTCCATCAGCGGGGATCACTTCCTGATGGATTACCCCTTCTCCACCACGGTGAAGATCTCCACG : 70

ENSGAL5747 : GGGTGCACGGGGGTGCTGGTGTCCGAGCAGCACGTCCTCACGGCTGCCCACTGCGTCCACGATGGCCAAG : 490
FWD : GGGTGCACGGGGGTGCTGGTGTCCGAGCAGCACGTCCTCACGGCTGCCCACTGCGTCCACGATGGCCAAG : 490
REV : GGGTGCACGGGGGTGCTGGTGTCCGAGCAGCACGTCCTCACGGCTGCCCACTGCGTCCACGATGGCCAAG : 140

ENSGAL5747 : ACTACGTGAAGGGTGCCAGGAAGATCAAGGTGGGCTTCCTGACGCCGGCCAATGGCACGGGGCGGCCGGT : 560
FWD : ACTACGTGAAGGGTGCCAGGAAGATCAAGGTGGGCTTCCTGACGCCGGCCAATGGCACGGGGCGGCCGGT : 560
REV : ACTACGTGAAGGGTGCCAGGAAGATCAAGGTGGGCTTCCTGACGCCGGCCAATGGCACGGGGCGGCCGGT : 210

ENSGAL5747 : GATGCGCTGGGCTCGTGTGCGGCGCACGCAGGTGCCCAAGGGTTGGATCCGGAGCCTCAACTCCATCAGC : 630
FWD : GATGCGCTGGGCTCGTGTGCGGCGCACGCAGGTGCCCAAGGGTTGGATCCGGAGCCTCAACTCCATCAGC : 630
REV : GATGCGCTGGGCTCGTGTGCGGCGCACGCAGGTGCCCAAGGGTTGGATCCGGAGCCTCAACTCCATCAGC : 280

ENSGAL5747 : ATGGACTACGATTACGCCCTTTTGGAGCTGCGCAGACCACACCGGCGCCCGCACATGGAGCTGGCGGTGG : 700
FWD : ATGGACTACGATTACGCCCTTTTGGAGCTGCGCAGACCACACCGGCGCCCGCACATGGAGCTGGCGGTGG : 700
REV : ATGGACTACGATTACGCCCTTTTGGAGCTGCGCAGACCACACCGGCGCCCGCACATGGAGCTGGCGGTGG : 350

ENSGAL5747 : CTCCGGCGGCGGAGGAAATGGCTGGGAAGAGGATTCATTTCTCAGGGTTTGACAGTGACCGGCCGGGAGA : 770
FWD : CTCCGGCGGCGGAGG....................................................... : 715
REV : CTCCGGCGGCGGAGGAAATGGCTGGGAAGAGGATTCATTTCTCAGGGTTTGACAGTGACCGGCCGGGAGA : 420

ENSGAL5747 : GCTGGTATACCGCTTCTGTGGGGTGGAGGATGAGACGGCACACCTCATCTACCAGCACTGTGATGCCAGG : 840
FWD : ...................................................................... : -
REV : GCTGGTATACCGCTTCTGTGGGGTGGAGGATGAGACGGCACACCTCATCTACCAGCACTGTGATGCCAGG : 490

ENSGAL5747 : CCTGGCGCATCTGGCTCTGGGGTGTATGGCAAGGTGTGGGATCCCGTGCGGCACAAGTGGGAGAGGAAGG : 910
FWD : ...................................................................... : -
REV : CCTGGCGCATCTGGCTCTGGGGTGTATGGCAAGGTGTGGGATCCCGTGCGGCACAAGTGGGAGAGGAAGG : 560

ENSGAL5747 : TGATTGGCATCTTCTCTGGGCATCAGTGGCTGGAAGTGGGGGGGGAGCAGCACGACTATAACGTTGCTGT : 980
FWD : ...................................................................... : -
REV : TGATTGGCATCTTCTCTGGGCATCAGCGGCTGGAAGTGGGGGGGGAGCAGCACGACTATAACGTTGCTGT : 630

ENSGAL5747 : TCGTCTGACTGCTCCCAAGTTTGCTCAGATTTGTTATTGGATCAAAGGGGACTATAAAAGCTGCCAGAAT : 1050
FWD : ...................................................................... : -
REV : TCGTCTGACTGCTCCCAAGTTTGCTCAGATTTGTTATTGGATCAAAGGGGACTATAAAAGCTGCCAGAAT : 700

ENSGAL5747 : GAATGA : 1056
FWD : ...... : -
REV : GAATGA : 706

ENSGALG00000011172

ENS11172 : ATGCACCAGAGCAGCATCAATGGGGTAGAGGACATGTCCATGCTGGGAGACCTGCACGAAGCTGCCATCC : 70
FWD : ATGCACCAGAGCAGCATCAATGGGGTAGAGGACATGTCCATGCTGGGAGACCTGCACGAAGCTGCCATCC : 70

ENS11172 : TACTCAACCTGCACCAGCGCTACCAGCAGGGTAACATCTACACCAACATTGGCTCCATCCTGGCGTCGGT : 140
FWD : TACTCAACCTGCACCAGCGCTACCAGCAGGGTAACATCTACACCAACATTGGCTCCATCCTGGCGTCGGT : 140

ENS11172 : GAACCCCTACAAGCCCATCCCTGGTCTGTACAGCATGGATGCCATAGAGCTGTACAGGCAGCACCGCCTG : 210
FWD : GAACCCCTACAAGCCCATCCCTGGTCTGTACAGCGTGGATGCCATAGAGCTGTACAGGCAGCACCGCCTG : 210

ENS11172 : GGTGAGCTGCCTCCCCATATCTTCGCCACCGCTAACGAGTGCTACTGCTGCTTGTGGAAGCGTCACGACA : 280
FWD : GGTGAGCTGCCTCCCCATATCTTCGCCACCGCTAACGAGTGCTACTGCTGCTTGTGGAAGCGTCACGACA : 280

ENS11172 : GCCAGTGTGTTCTGATAAGCGGAGAAAGCGGAGCTGGAAAGACTGAGAGCACCAAGCTGCTGCTGAAGTT : 350
FWD : GCCAGTGTGTTCTGATAAGCGGAGAAAGCGGAGCTGGAAAGACTGAGAGCACCAAGCTGCTGCTGAAGTT : 350

ENS11172 : CCTGTCCGCCATGAGCCAGACCTCCCTTGGGGCCCCGGCCTCTGAGAAGAGCACACACGTGGAGGAAGCC : 420
FWD : CCTGTCCGCCATGAGCCAGACCTCCCTTGGGGCCCCGGCCTCCGAGAAGAGCACACACGTGGAGGAAGCC : 420

ENS11172 : ATCCTGGAGAGCAGCCCAATTCTGGAGGCCTTTGGAAACGCCAAGACGGTTTATAACAACAACTCTAGCC : 490
FWD : ATCCTGGAGAGCAGCCCAATTCTGGAGGCCTTTGGAAACGCCAAGACGGTTTATAACAACAACTCTAGCC : 490

ENS11172 : GCTTTGGCAAGTTCATCCAGCTGCACTTCTCTCAGCATGGGCACATCCAGGGCGGACGAGTCACTGACTA : 560
FWD : GCTTCGGCAAGTTTATCCAGCTGCACTTCTCTCAGCATGGGCACATCCAGGGCGGACGAGTCACTGACTA : 560

ENS11172 : TTTACTGGAAAAGAACAGAGTGGTACACCAGAACCCTGGAGAGAGGAATTACCACATCTTCTATGCTCTG : 630
FWD : TTTACTGGAAAAGAACAGAGTGGTACACCAGAACCCTGGAGAGAGGAATTACCACATCTTCTATGCTCTG : 630

ENS11172 : CTGGCTGGTGTGAGCGGGGAGCTGAAAGAGAGCCTCTCCCTCGCTGAGCCGGAGACTTACCGGTACTTGA : 700
FWD : CTGGCTGGTGTGACCGG..................................................... : 647

ENS11172 : CTGGCTGGTGTGAGCGGGGAGCTGAAAGAGAGCCTCTCCCTCGCTGAGCCGGAGACTTACCGGTACTTGA : 700
REV : ..............CGGGGAGCTGAAAGAGAGCCTCTCCCTCGCTGAGCCGGAGACTTACCGGTACTTGA : 56

ENS11172 : GCCAGTCCGGCTGTGTGAGCGATGAGAACCTGAATGATGGAGAAATGTTCACTAAGGTCATGACTGCCAT : 770
REV : GCCAGTCCGGCTGTGTGAGCGATGAGAACCTGAATGATGGAGAAATGTTCACTAAGGTCATGACTGCCAT : 126

ENS11172 : GAAGGTGGTGGACTTCAGCAGTGAAGAGATCCGAGACATCTTCAAACTTCTTTCTGGCACTCTTCACCTG : 840
REV : GAAGGTGGTGGACTTCAGCAGTGAAGAGATCCGAGACATCTTCAAACTTCTTTCTGGCACTCTTCACCTG : 196

ENS11172 : GGAAACGTTGAGTTCATGACAGCTGGTGGAGCCCAGGTTACAACCAAAGCAGTGCTGAACATTGCCAGCG : 910
REV : GGAAACGTTGAGTTCATGACAGCTGGTGGAGCCCAGGTTACAACCAAAGCAGTGCTGAACATTGCCAGCG : 266

ENS11172 : ACCTCCTGGGCTTAGATGCCTTCCAGCTTTCCGAAGTCCTGACTCAGAGATCCATGATTCTGCGTGGGGA : 980
REV : ACCTCCTGGGCTTAGATGCCTTCCAGCTTTCCGAAGTCCTGACTCAGAGATCCATGATTCTGCGTGGGGA : 336

ENS11172 : AGAGATCAGCTCCCCCCTCACTGTGGAGCAGGCAGCAGACTCCCGGGACTCCCTCTCCATGGCGCTGTAT : 1050
REV : AGAGATCAGCTCCCCCCTCACTGTGGAGCAGGCAGCAGACTCCCGGGACTCCCTCTCCATGGCGCTGTAT : 406

ENS11172 : TCCCAGTGCTTCTCGTGGCTCATTAGCAAGATTAATACGAAGATCAAAGGCAAGGAAAACTTTAAGTCTG : 1120
REV : TCCCAGTGCTTCTCGTGGCTCATTAGCAAGATTAATACGAAGATCAAAGGCAAGGAAAACTTTAAGTCTG : 476

ENS11172 : TGGGCATCCTGGATATCTTTGGCTTCGAGAACTTCCAGGTGAATCGTTTTGAGCAGTTTAACATTAACTA : 1190
REV : TGGGCATCCTGGATATCTTTGGCTTCGAGAACTTCCAGGTGAATCGTTTTGAGCAGTTTAACATTAACTA : 546

ENS11172 : TGCAAATGAGAAGCTCCAGGAATATTTCAACAAGCATATCTTCTCCTTGGAGCAGCTGGAGTACAACAGG : 1260
REV : TGCAAATGAGAAGCTCCAGGAATATTTCAACAAGCATATCTTCTCCTTGGAGCAGCTGGAGTACAACAGG : 616

ENS11172 : GAAGGGATAAATTGGGAAGCTATTGACTGGATGGATAATGCGGAGTGCCTGGACC : 1315
REV : GAAGGGATAAATTGGGAAGCTATTGACTGGATGGATAATGCGGAGTGCCTGGACC : 671

ENSGALG0000005395

ENS15395 : ATGCTTCTGCTTCAGGCCTGCCGGGCTGCGCTCTGCCTGCTGCTGCTCTGCCTAAGCTGCTGTCTGGAGG : 70
FWD : ATGCTTCTGCTTCAGGCCTGCCGGGCTGCGCTCTGCCTGCTGCTGCTCTGCCTAAGCTGCTGTCTGGAGG : 70
REV : ...................................................................... : -
REV2 : ...................................................................... :

ENS15395 : CTGCCAGGGGTTTGAAGGAAGCAGTGAAGTGTGAAAGCGTTACTGAAGCAACCCTCGGTGAAGAGGCAAA : 140
FWD : CTGCCAGGGGTTTGAAGGAAGCTGTGAAGTGTGAGAGCGTTACTGAAGCAACCCTCGGTGAAGAGGCAAA : 140
REV : ...................................................................... : -
REV2 : ...................................................................... : -

ENS15395 : TTTCTCTTGTGACTTCTTGCTCGCAATGGATGTCTTTCAAGTCACTTGGCAGAAGATAAATGGATCTTCC : 210
FWD : TTTCTCTTGTGACTTCTTGCTCGCAATGGATGTCTTTCAAGTCACTTGGCAGAAGATAAATGGATCTTCC : 210
REV : ...................................................................... : -
REV2 : ...................................................................... : -

ENS15395 : TTCCAGAACATAGCCACTTACAGCCAAACCCGTGGGCTGCGACTGATAGGATCATTTCGGAGGAAGGCAC : 280
FWD : TTCCAGAACATAGCCACTTACAGCCAAACCCGTGGGCTGCGACTGATAGGATCATTTCGGAGGAAGGCAC : 280
REV : ...................................................................... : -
REV2 : ...................................................................... : -

ENS15395 : GTTTTGCTAGAGCAGCCCTGAACACCTCAGTCATCACTCTGAAAAATCTCACATTTGAGGATGTGTCCTG : 350
FWD : GTTTTGCTAGAGCAGCCCTGAACACCTCAGTCATCACTCTGAAAAATCTCACATTTGAGGATGTGTCCTG : 350
REV : .................CTGAACACCTCAGTCATCACTCTGAAAAATCTCACATTTGAGGATGTGTCCTG : 53
REV2 : ..................................................ACATTTGAGGATGTGTCCTG : 20

ENS15395 : TTACAGATGCATCTTCAATGTGTTCCCTCATGGCTCTTTCAGCAGCAAAGCTATGTGCCTCAACATCCAA : 420
FWD : TTACAGATGCATCTTCAATGTGTTCCCTCATGGCTCTTTCAGCAGCAAAGCTATGTGCCTCAACATCCAA : 420
REV : TTACAGATGCATCTTCAATGTGTTCCCTCATGGCTCTTTCAGCAGCAAAGCTATGTGCCTCAACATCCAA : 123
REV2 : TTACAGATGCATCTTCAATGTGTTCCCTCATGGCTCTTTCAGCAGCAAAGCTATGTGCCTCAACATCCAA : 90

ENS15395 : AAAAGTGGAAACACAAAAAAACCAGAAGTCAAAACGTTGGATGTGGGTTCCCCCAGTACAAGAGGTATTC : 490
FWD : AAAAGTGGAAACACGAACAAATCAGAAGTCAAAACGTTGGATATGGGTTCCCCCAGTACAAGAGGTATTC : 490
REV : AAAAGTGGAAACACGAACAAATCAGAAGTCAAAACGTTGGATATGGGTTCCCCCAGTACAAGAGGTATTC : 193
REV2 : AAAAGTGGAAACACGAACAAATCAGAAGTCAAAACGTTGGATATGGGTTCCCCCAGTACAAGAGGTATTC : 160

ENS15395 : AGAAGAGAATTGGCCTTGTGGTGGTCTTCATCGGTGCTGTCTTAGCAGTCTTGACACTTCTCATCATGGG : 560
FWD : AGAAGAGAATTGGCCTTGTGGTGGTCTTCATCGGTGCTGTCTTAGCAGTCTTGACACTTCTCATCATGGG : 560
REV : AGAAGAGAATTGGCCTTGTGGTGGTCTTCATCGGTGCTGTCTTAGCAGTCTTGACACTTCTCATCATGGG : 263
REV2 : AGAAGAGAATTGGCCTTGTGGTGGTCTTCATCGGTGCTGTCTTAGCAGTCTTGACACTTCTCATCATGGG : 230

ENS15395 : GCTAACCAACAGAAAAAGGAGACAACTGCAGAAACACAGAGCACGTAGCACACCTGAAATGGAGAAAGGC : 630
FWD : GCTAACCAACAGAAAAAGGAGACAACTGCAGAAACACAGAGCACGTAGCACACCTGAAAAGGAGAAAGGC : 630
REV : GCTAACCAACAGAAAAAGGAGACAACTGCAGAAACACAGAGCACGTAGCACACCTGAAAAGGAGAAAGGC : 333
REV2 : GCTAACCAACAGAAAAAGGAGACAACTGCAGAAACACAGAGCACGTAGCACACCTGAAAAGGAGAAAGGC : 300

ENS15395 : TTACAGCAGGATGTAAGTGAGCAATCCGAAAGCCTGAACACACTGAAGGACCAAGACAGCACTTATCAAA : 700
FWD : TTACAGCAGGATGTAAGTGAGCAATCCGAAAGCCTGAACACACTGAAGGACCAAGACAGCACTTATCAAA : 700
REV : TTACAGCAGGATGTAAGTGAGCAATCCGAAAGCCTGAACACACTGAAGGACCAAGACAGCACTTATCAAA : 403
REV2 : TTACAGCAGGATGTAAGTGAGCAATCCGAAAGCCTGAACACACTGAAGGACCAAGACAGCACTTATCAAA : 370

ENS15395 : ATGAGAGGCAGACACCAGGATCTTCACTTCATAAAAGGCTACTAAATCAGAAGAGAAATATGGAAGAGAT : 770
FWD : ATGAGAGGCAGACACCAGGATCTTCACTTCACAAAAGG................................ : 738
REV : ATGAGAGGCAGACACCAGGATCTTCACTTCACAAAAGGCTACTAAATCAGAGGAGAAATACGGAAGAGAT : 473
REV2 : ATGAGAGGCAGACACCAGGATCTTCACTTCACAAAAGGCTACTAAATCAGAGGAGAAATACGGAAGAGAT : 440

ENS15395 : TGAAGGAAGAGAAACCTGGAAGAGAAACAAGAGGCTTGTGTTTTCAGAGGAAGCTGACAGCCAAGACAGT : 840
FWD : ...................................................................... : -
REV : TGAAGGAAGAGGCACCTGGAAGAGAAACAAGAGGCTTGTGTTTTCAGAGGAAGCTGACAGCCAAGACAGT : 543
REV2 : TGAAGGAAGAGGCACCTGGAAGAGAAACAAGAGGCTTGTGTTTTCAGAGGAAGCTGACAGCCAAGACAGT : 510

ENS15395 : ACCAGCCCCAACATACCCCAGAGGGAGCTCACTGAGTTGAGCAATAATGAGCTGGGCTGCACACCCATCA : 910
FWD : ...................................................................... : -
REV : ACCAGCCCCAGCATACCCCAGAGGGAGCTAACAGAGTTGAGCAGTAATGAGCTGGGCTGCACACCCATCA : 613
REV2 : ACCAGCCCCAGCATACCCCAGAGGGAGCTAACAGAGTTGAGCAGTAATGAGCTGGGCTGCACACCCATCA : 580

ENS15395 : AGAACAACAGTGAGACACAGGCATGTGGGGAGTCTGAATTATGTACAGTAATAGTATGTACAGTATGTAC : 980
FWD : ...................................................................... : -
REV : AGAACAACAGTGAGACAGAGGCATGTGGGGAGTCTGAATTATGCCCTGCCATGGC...TACTCCTTGG.. : 678
REV2 : AGAACAACAGTGAGACAGAGGCATGTGGGGAGTCTGAATTATGCCCTGCCATGGC...TACTCCTTGG.. : 645

ENS15395 : ATACAGTACGATCTCCAGTACAGGAGAGAAATCAGCCCACCAAAGCCCTGTCTCTACAAGTCCAGAGGAA : 1050
FWD : ...................................................................... : -
REV : .............CCTAGTACAGGAGAGCAATCAGCCCACCAAAGCCCTGTCTCTACAAGTCCAGAGGAA : 735
REV2 : .............CCCAGTACAGGAGAGCAATCAGCCCACCAAAGCCCTGTCTCTACAAGTCCAGAGGAA : 702

ENS15395 : CACTGA : 1056
FWD : ...... : -
REV : CACTGA : 741
REV2 : CACTGA : 708

ENSGALG00000027419

ENS27419 : ATGATGGTTACTTGCCTGCTCCTCTCCAGCCTCTGGGCCCTTGCATCAGGCTCTGTGCAGGTGCTGCACA : 70
FWD : ATGATGGTTACTTGCCTGCTCCTCTCCAGCCTCTGGGCCCTTGCATCAGGCTCTGTGCAGGTGCTGCACA : 70
REV : ...................................................................... : -

ENS27419 : AAAAGGTGCAGTCGGTCCAGGCAGGCGGAAATATTACTTTCTCATGCCAATCAGTCACGAACGAAGATGT : 140
FWD : AAAAGGTGCAGTCGGTCCAGGCAGGCGGAAATATTACTTTCTCATGCCAATCAGTCACGAACGAAGATGT : 140
REV : ...................................................................... : -

ENS27419 : AATACAAGTGACCTGGCAGAAGGAGATGGATGGGGCTGAAGACAACATAGCAACTTATAGTACAATGAAC : 210
FWD : AATACAAGTGACCTGGCAGAAGGAGATGGATGGGGCTGAAGACAACATAGCAACTTATAGTACAATGAAC : 210
REV : ...................................................................... : -

ENS27419 : GGCGAAAAGATAGCAAAAGCCTATGATGGCCACGTGAGCTTTGCCCATAGCGGGCTGGAAGCCTCGTCCA : 280
FWD : GGCGAAAAGATAGCAAAAGCCTATGATGGCCACGTGAGCTTTGCCCATAGCGGGCTGGAAGCCTCGTCCA : 280
REV : ..........................................................AAGCCTCGTCCA : 12

ENS27419 : TCTCCCTTCACCGAGTCACCCTGCAAGATGAAGGATGCTACAAGTGCATCTTCAACACCTTTCCCTCGGG : 350
FWD : TCTCCCTTCACCGAGTCACCCTGCAAGATGAAGGATGCTACAAGTGCATCTTCAACACCTTTCCCTCGGG : 350
REV : TCTCCCTTCACCGAGTCACCCTGCAAGATGAAGGATGCTACAAGTGCATCTTCAACACCTTTCCCTCGGG : 82

ENS27419 : AGCTGTCACTGGCAGGATGTGCCTCAAGGTTTACGCCATCTCAGACCCCAAAGTGGAAGCCAAGCTTATA : 420
FWD : AGCTGTCACTGGCAGGATGTGCCTCAAGGTTTACGCCATCTCAGACCCCAAAGTGGAAGCCAAGCTTATA : 420
REV : AGCTGTCACTGGCAGGATGTGCCTCAAGGTTTACGCCATCTCAGACCCCAAAGTGGAAGCCAAGCTTATA : 152

ENS27419 : CCCAGTCCTGACAACGCTGAAGTCTCTGAGGAAGTGGTGGGGATGAGCTGCTCAGCAACAGGGAAGCCAG : 490
FWD : CCCAGTCCTGACAACGCTGAAGTCTCTGAGGAAGTGGTGGGGATGAGCTGCTCAGCAACAGGGAAGCCAG : 490
REV : CCCAGTCCTGACAACGCTGAAGTCTCTGAGGAAGTGGTGGGGATGAGCTGCTCAGCAACAGGGAAGCCAG : 222

ENS27419 : CTCCAAAAATCACCTGGCACCTTCCAAGCACGCTGCTGCAGAAACCAAAGGAGTACCACATCAAGCTCAC : 560
FWD : CTCCAAAAATCACCTGGCACCTTCCAAGCACGCTGCTGCAGAAACCAA...................... : 538
REV : CTCCAAAAATCACCTGGCACCTTCCAAGCACGCTGCTGCAGAAACCAAAGGAGTACCACATCAAGCTCAC : 292

ENS27419 : CAACCAGACCATCACTGTCATCAGCAACTTCACCCATGCCCACTCCAAAATCCTCAAGGAGTACCCCATT : 630
FWD : ...................................................................... : -
REV : CAACCAGACCATCACTGTCATCAGCAACTTCACCCATGCCCACTCCAAAATCCTCAAGGAGTACCCCATT : 362

ENS27419 : GCCTGTGTGATCCAGCACCCATCTCTAAATGTGACCCTGCCCCTGCCCATGGACAATCTGACACAAGGTC : 700
FWD : ...................................................................... : -
REV : GCCTGTGTGATCCAGCACCCATCTCTAAATGTGACCCTGCCCCTGCCCATGGACAATCTGACACAAGGTC : 432

ENS27419 : AGGACAATATCATGGCACCAACCATGGCCATTATTGTGGGGGTGCTGGTCCCTCTGATATTCCTCTTCGC : 770
FWD : ...................................................................... : -
REV : AGGACAATATCATGGCACCAACCATGGCCATTATTGTGGGGGTGCTGGTCCCTCTGATATTCCTCTTCGC : 502

ENS27419 : GTTGCTCCTTTGCCTGAGACACCTTCGTGACCCAGAGAGAAACACAGCCTGGCCTCGCTGGGTCCTTGCT : 840
FWD : ...................................................................... : -
REV : GTTGCTCCTTTGCCTGAGACACCTTCGTGACCCAGAGAGAAACACAGCCTGGCCTCGCTGGGTCCTTGCT : 572

ENS27419 : GTATGTGCCAAGGAGAGAGCATGTGGAAGGTACATGCTTACTGGCAAGCGGGCTACTGCTGCAGTGCCCA : 910
FWD : ...................................................................... : -
REV : GTATGTGCCAAGGAGAGAGCATGTGGAAGGTACATGCTTACTGGCAAGCGGGCTGCTGCTGCAGTGCCCA : 642

ENS27419 : ACACGTGA : 918
FWD : ........ : -
REV : ACACGTGA : 650

ENSGALG00000022324
ENSGAL22324 : ATGAGGATGCCGCTTCCCACTGCAGGAAGAGTCAGCCCTGCTTGCTGGAGACAGCCCGGCATGATGGCTT : 70
FWD : ATGAGGATGCCGCTTCCCACTGCAGGAAGAGTCAGCCCTGCTTGCTGGAGACAGCCCGGCATGATGGCTT : 70

ENSGAL22324 : ACGGCACAGCTTGGCACCATGGGCTGCTGAGGCAAGCTGGCTCCTATAAGAATGACTATGACTGGACCCA : 140
FWD : ACGGCACAGCTTGGCACCATGGGCTGCTGAGGCAAGCTGGCTCCTATAAGAATGACTATGACTGGACCCA : 140

ENSGAL22324 : CTGTGAAGCCAATGCTTTGAGCGAAGCCCCCATCACAGTGCTCATCTGCCTCTGTGGGCTGGTGGGCAAT : 210
FWD : CTGTGAAGCCAATGCTTTGAGCGAAGCCCCCATCACAGTGCTCATCTGCCTCTGTGGGCTGGTGGGCAAT : 210

ENSGAL22324 : GCGGCCGCCCTCTGGCTACTCAGACCCCACATCCGCAGGAACTTCATCACCATCTACATCTTCAACCTGG : 280
FWD : GCGGCCGCCCTCTGGCTACTCAGACCCCACATCCGCAGGAACTTCATCACCATCTACATCTTCAACCTGG : 280
REV : ................................................................ACCTGG : 6

ENSGAL22324 : CCGTGGCTGACTTCATCTTCCTCTTCTCTGTTGTCATTGCCCTCGTGATATTTTACGGCCCACAGAGCTT : 350
FWD : CCGTGGCTGACTTCATCTTCCTCTTCTCTGTCGTCATTGCCCTCGTGATATTTTACGGCCCACAGAGCTT : 350
REV : CCGTGGCTGACTTCATCTTCCTCTTCTCTGTCGTCATTGCCCTCGTGATATTTTACGGCCCACAGAGCTT : 76

ENSGAL22324 : TTGTCACAGTCTGGGCTCGCAGGACATGATGACTGTGTTGAGCGTGGTCATCACCTTTGCTTTCATTGCT : 420
FWD : TTGTCACAGTCTGGGCTCGCAGGACATGATGACTGTGTTGAGCGTGGTCATCACCTTTGCTTTCATTGCT : 420
REV : TTGTCACAGTCTGGGCTCGCAGGACATGATGACTGTGTTGAGCGTGGTCATCACCTTTGCTTTCATTGCT : 146

ENSGAL22324 : GGTGTCTACCTCATGGCAGCCCTGGGGGCCAGGACCTGCCTGCCTGCCATCCCACGGCCCCTCTGCCCTT : 490
FWD : GGTGTCTACCTCATGGCAGCCCTGGGGGCCAGGACCTGCCTGCCTGCCATCCCACGGCCCCTCTGCCCTT : 490
REV : GGTGTCTACCTCATGGCAGCCCTGGGGGCCAGGACCTGCCTGCCTGCCATCCCACGGCCCCTCTGCCCTT : 216

ENSGAL22324 : GCCAGAACTCCTGGTGCTTGCCAGCACTCCTGTGTGCCCTGCTCTGGGCCCTTGCCCTCTTGCTCACTCT : 560
FWD : GCCAGAACTCCTGGTGCTTGCCAGCACTCCTGTGTGCCCTGCTCTGGGCCCTTGCCCTCTTGCTCACTCT : 560
REV : GCCAGAACTCCTGGTGCTTGCCAGCACTCCTGTGTGCCCTGCTCTGGGCCCTTGCCCTCTTGCTCACTCT : 286

ENSGAL22324 : GACCCTCTATTTCTCCCCACCGATGCTAGTGGCCTTTGTCCTCAGCTACCTCTTGTCGGTGCTTATCCTC : 630
FWD : GACCCTCTATTTCTCCCCACCGATGCTAGTGGCCTTTGTCCTCAGCTACCTCTTGTCGGTGCTTATCCTC : 630
REV : GACCCTCTATTTCTCCCCACCGATGCTAGTGGCCTTTGTCCTCAGCTACCTCTTGTCGGTGCTTATCCTC : 356

ENSGAL22324 : ATTCTCTCTGGTTTAGCCCTCTTTGCCAAGCTCTTATGCTGCTCATGGCAATATCTCCCAAGGAAGTTCT : 700
FWD : ATTCTCTCTGGTTTAGCCCTCTTTGCCAAGCTCTTATGCTGCTCATGGCAATATCTCCCAAGGAAGTTCT : 700
REV : ATTCTCTCTGGTTTAGCCCTCTTTGCCAAGCTCTTATGCTGCTCATGGCAATATCTCCCAAGGAAGTTCT : 426

ENSGAL22324 : ACATTGCAGTCTTGCTTGCTATCATCTCCTTCCCGTTCTTCACTGCTGACTTTGCCTACTGGCTCTTGCT : 770
FWD : ACATTGCAGTCTTGCTTGCTATCATCTCCTTCCCGTTCTTCGCTGCTGACTTTGCC.............. : 756
REV : ACATTGCAGTCTTGCTTGCTATCATCTCCTTCCCGTTCTTCGCTGCTGACTTTGCCTACTGGCTCTTGCT : 496

ENSGAL22324 : AAGGCTGTTTGATTTTTCGGTTCTTGCTTTTGACACCTCTCTCCTATTTGCCTGTGTGAACAGCAGTATT : 840
REV : AAGGCTGTTTGATTTTTCGGTTCTTGCTTTTGACACCTCTCTCCTATTTGCCTGTGTGAACAGCAGTATT : 566

ENSGAL22324 : AAGCCTGTCCTTTACTTCCTTGCTGGGTGCTGCATGAAGAAGTTCACGTTCTCTATCAGGGTTGCTTGCC : 910
REV : AAGCCTGTCCTTTACTTCCTTGCTGGGTGCTGCATGAAGAAGTTCACGTTCTCTATCAGGGTTGCTTGCC : 636

ENSGAL22324 : ATAGGGCTTTTGAAGGGGTAATGGAGCCAGATATCGTAGGCGAAACCCTACAAGAAAGCACAGTGGAAAC : 980
REV : ATAGGGCTTTTGAAGGGGTAATGGAGCCAGATGTCGTAGGCGAAACCCTACAAGAAAGCACAGTGGAAAC : 706

ENSGAL22324 : CCCACAAGAAAGCACAGTGAATGTTTAA : 1008
REV : CCCACAAGAAAGCACAGTGAATGTTTAA : 734

ENSGALG0000002590

ENSGAL2590 : ATGAAGATGGAGACAGGGGATGAAATTGCTATTGTGGGAATAGCATGCAACTTTCCTGGAGGTGAAGGAA : 70
FWD : ATGAAGATGGAGACAGGGGATGAAATTGCTATTGTGGGAATAGCATGCAACTTTCCTGGAGGTGAAGGAA : 70
REV : ...................................................................... : -

ENSGAL2590 : TTGACAATTTCTGGAGAGTGCTGGAGGAAGGCAAAAACTGCACCGTAGAAATTCCCCCAGAGAGATTTAA : 140
FWD : TTGACAATTTCTGGAGAGTGCTGGAGGAAGGCAAAAACTGCACCGTAGAAATTCCCCCAGAGAGATTTAA : 140
REV : ...................................................................... : -

ENSGAL2590 : TACCGAAGACTGGTATGATCCAGACAGCAACAAGCCAGGGAAGATGTATACAACACGAGCTGCTCTTCTC : 210
FWD : TACCGAAGACTGGTATGATCCAGACAGCAATAAGCCAGGGAAGATGTATACAACACGAGCTGCTCTTCTC : 210
REV : ...................................................................... : -

ENSGAL2590 : AATGAATTTAACACATTTGACAACCACCTGTTTGGAATTAATAGTGTGGAAGTTGAAAGCATGGATCCAC : 280
FWD : AATGAATTTAACACATTTGACAACCACCTGTTTGGAATTAATAGTGTGGAAGTTGAAAGCATGGATCCAC : 280
REV : .........................................TAGTGTGGAAGTTGAAAGCATGGATCCAC : 29

ENSGAL2590 : AACAGAAATTACTTTTAGAATGCACATACAAAGTGCTAGAGGACGCAGGAGTCCCTGTAGAAGCCATCAG : 350
FWD : AGCAGAAATTACTTTTAGAATGCACATACAAAGTGCTAGAGGACGCAGGAGTCCCTGTAGAAGCCATCAG : 350
REV : AGCAGAAATTACTTTTAGAATGCACATACAAAGTGCTAGAGGACGCAGGAGTCCCTGTAGAAGCCATCAG : 99

ENSGAL2590 : TGGCACCAAAACAGGTGTTTTTGTTGGACTTATGAATCGAGACTTTGAAATCGTAAGAAGCAAAGCAGTA : 420
FWD : TGGCACCAAAACAGGTGTTTTTGTTGGACTTATGAATCGAGACTTTGAAATCGTAAGAAGCAAAGCAGTA : 420
REV : TGGCACCAAAACAGGTGTTTTTGTTGGACTTATGAATCGAGACTTTGAAATCGTAAGAAGCAAAGCAGTA : 169

ENSGAL2590 : AGTGAAATAAATCATTATGCTGGTACAGGATCAGCAATGAGCATAGCTGCTAACAGGGTCTCCTTCACAT : 490
FWD : AGTGAAATAAATCATTATGCTGGTACAGGATCAGCAATGAGCATAGCTGCTAACAGGGTCTCCTTCACAT : 490
REV : AGTGAAATAAATCATTATGCTGGTACAGGATCAGCAATGAGCATAGCTGCTAACAGGGTCTCCTTCACAT : 239

ENSGAL2590 : TTAATCTGACTGGACCATCGCTGACAGTTGACACTGCTTGTTCATCATTTCTTTTTGCTCTGCACTGTGC : 560
FWD : TTAATCTGACTGGACCATCGCTGACAGTTGACACTGCTTGTTCATCATTTCTTTTTGCTCTGCACTGTGC : 560
REV : TTAATCTGACTGGACCATCGCTGACAGTTGACACTGCTTGTTCATCATTTCTTTTTGCTCTGCACTGTGC : 309

ENSGAL2590 : CTTGCGAGCAATTAAATCAGGAGACTGTGAGGCAGCTATTTGCGGTGGTGTGAACAGCATAATAGATCCC : 630
FWD : CTTGCGAGCAATTAAATCAGGAGACTGTGAGGCAGCTATTTGCGGTGGTGCGAACAGCATAATAGATCCC : 630
REV : CTTGCGAGCAATTAAATCAGGAGACTGTGAGGCAGCTATTTGCGGTGGTGCGAACAGCATAATAGATCCC : 379

ENSGAL2590 : CGCAACTTTGTGTCTCTCAGTAAAGCAAAAATGATCTCTCCAGATGGAATAAGTAAACCCTTTTCCAAAA : 700
FWD : CGCAACTTTGTGTCTCTCAGTAAAGCAAAAATGATCTCTCCAGATGGAATAAGTAAACCCTTTTCCAAAA : 700
REV : CGCAACTTTGTGTCTCTCAGTAAAGCAAAAATGATCTCTCCAGATGGAATAAGTAAACCCTTTTCCAAAA : 449

ENSGAL2590 : AGGCAGATGGCTATGGAAGGGGAGAAGGCTGTGGTGTTGTATTCCTCAAACCACTGGAAAAGGTAAGATT : 770
FWD : AGGCAGATGGCTATGGAAGGGGAGAAGGCTGTGGTGTTGTATTCCTCAAACCA................. : 753
REV : AGGCAGATGGCTATGGAAGGGGAGAAGGCTGTGGTGTTGTATTCCTCAAACCACTGGAAAAGGTAAGATT : 519

ENSGAL2590 : GTTTGTGAAGAAGCCTGAAATAAGCCTGGCTTCTTACTTGAATTTTGTATTTCCAATTCCCATAATTACT : 840
FWD : ...................................................................... : -
REV : GTTTGTGAAGAAGCCTGAAATAAGCCTGGCTTCTTACTTGAATTTTGTATTTCCAATTCCCATAATTACT : 589

ENSGAL2590 : GTAGCAAAAAGCTTTTCTAATTCAAGCTTACTGTTAGCATTAAGAAAACATTTATGCTCCTCTCCTGCAC : 910
FWD : ...................................................................... : -
REV : GTAGCAAAAAGCTTTTCTAATTCAAGCTTACTGTTAGCATTAAGAAAACATTTATGCTCCTCTCCTGCAC : 659

ENSGAL2590 : TGAGAATGATATCACAAATTTAA : 933
FWD : ....................... : -
REV : TGAGAATGATATCACAAATTTAA : 682

ETS2

ETS2 : ATGAGTGAATTTGCGATCAGAAACATGGATCAAGTAGCACCTGTCTCCAATATGTACAGAGGAATGCTCA : 70
FWD : ATGAGTGAATTTGCGATCAGAAACATGGATCAAGTAGCACCTGTCTCCAATATGTACAGAGGAATGCTCA : 70

ETS2 : AGCGGCAACCAGCGTTTGACACCTTTGATAGCTCCAACTCCCTGTTTGCTGGCTATTTTTTATCGCTAAA : 140
FWD : AGCGGCAACCAGCGTTTGACACCTTTGATAGCTCCAACTCCCTGTTTGCTGGCTACTTTTTATCGCCAAA : 140

ETS2 : TGAAGATCAAACGCTCCAGGAAGTGCCAACGGGATTTGATTCTACTTCTTACGAGTCCAACAACTGTGAA : 210
FWD : TGAAGATCAAACGCTCCAGGAAGTGCCAACAGGATTTGATTCTACTTCTTACGAGTCCAACAACTGTGAA : 210

ETS2 : TTGCCTCTGTTAACCCCGTGCAGTAAGGCTGTGATGAGTCAAGCCCTGAAAGATACTTTCAGTGGCTTCA : 280
FWD : TTGCCTCTGTTAACCCCGTGCAGTAAGGCTGTGATGAGTCAAGCCCTGAAAGATACTTTCAGTGGCTTCA : 280

ETS2 : CAAAGGAACAGTGTCGGCTGGGTATACCAAATAATCCCTGGTTGTGGACTGAACAGCATGTTTGCCAGTG : 350
FWD : CAAAGGAACAGTGTCGGCTGGGTATACCAAATAATCCCTGGTTGTGGACTGAACAGCATGTTTGCCAGTG : 350

ETS2 : GCTTGCATGGGCTACCAATGAATTTAGCTTGGCAAATGTGAACATCCATCAGTTTCTTATGAGTGGCCAA : 420
FWD : GCTTGCATGGGCTACCAATGAATTTAGCTTGGCAAATGTGAACATCCATCAGTTTCTTATGAGTGGCCAA : 420

ETS2 : GACCTCTGCAACCTGGGCAAGGAACGCTTCCTGGAACTTGCACCTGACTATGTGGGTGACATTCTGTGGG : 490
FWD : GACCTCTGCAACCTGGGCAAGGAACGCTTCCTGGAACTTGCACCTGACTATGTGGGTGACATTCTGTGGG : 490

ETS2 : AACACCTGGAGCAGATGATAAAAGACAGCCAAGAAAAAACACAGGATCAATATGTGGAGAGCTCTCATCT : 560
FWD : AACACCTGGAGCAGATGATAAAAGACAGCCAAGAAAAAACACAGGATCAATATGTGGAGAGCTCTCATCT : 560

ETS2 : CACCTCAGTTCCTCACTGGGTCAATAATAACTCCTTAACTGTTAATGTGGATCAGACTCCCTATGGTATA : 630
FWD : CACCTCAGTTCCTCACTGGGTCAATAATAACTCCTTAACTGTTAATGTGGATCAGACTCCCTATGGTATA : 630

ETS2 : CAGATGCCTGGGTACCCTAAAGCCCTCAGTTATCCCAAGCCCAATCTCCTGAGTGATATCTGCCAGACTT : 700
FWD : CAGATGCCTGGGTACCCTAAAGCCCTCAGTTATCCCAAGCCCAATCTCCTGAGTGATATCTGCCAGACTT : 700

ETS2 : CTACAGGACCAAATCTCCTTAGTCCAGAACAAGACTTTTCATTGTTCCCTAAAACCCAAGTAGATGCAGT : 770
FWD : CTACAGGACCAAATCTCCTTAGTCCAGAACA....................................... : 731

ETS2 : CTACAGGACCAAATCTCCTTAGTCCAGAACAAGACTTTTCATTGTTCCCTAAAACCCAAGTAGATGCAGT : 770
REV : .............................CAAGACTTTTCATTGTTCCCTAAAACCCAAGTAGATGCAGT : 41

ETS2 : TAGTGTGAACTACTGTACTGTAAATCAAGATTTCACAAGAAGTAATCTGAACTTGCTGATAGATAATTCT : 840
REV : TAGTGTGAACTACTGTACTGTAAATCAAGATTTCACAAGAAGTAATCTGAACTTGCTGATAGATAATTCT : 111

ETS2 : GGCAAACTTAGAGAACACGAATCCAGTGAAAGTGGTGCAGAAAGCTATGAAAGCTCAGATTCAATGCTGC : 910
REV : GGCAAACTTAGAGAACACGAATCCAGTGAAAGTGGTGCAGAAAGCTATGAAAGCTCAGATTCAATGCTGC : 181

ETS2 : AGTCCTGGAACAGCCAGTCGTCGCTAGTGGATTTACAGCGTGTGCCATCCTATGAGAGTTTTGAAGATGA : 980
REV : AGTCCTGGAACAGCCAGTCGTCGCTAGTGGATTTACAGCGTGTGCCATCCTATGAGAGTTTTGAAGATGA : 251

ETS2 : CTGTAGCCAGTCCTTGTGTATGAGCAAACCTACGATGTCTTTCAAAGACTATATTCAAGATCGAAGCGAT : 1050
REV : CTGTAGCCAGTCCTTGTGTATGAGCAAACCTACGATGTCTTTCAAAGACTATATTCAAGATCGAAGCGAT : 321

ETS2 : CCTGTAGAGCAAGGGAAACCAGTTATACCAGCAGCAATTCTAGCTGGCTTTACTGGCAGTGGACCTATAC : 1120
REV : CCTGTAGAGCAAGGGAAACCAGTTATACCAGCAGCAATTCTAGCTGGCTTTACTGGCAGTGGACCTATAC : 391

ETS2 : AGCTATGGCAATTCCTGCTGGAGTTACTGACTGACAAGTCCTGCCAGTCATTTATTAGTTGGACTGGAGA : 1190
REV : AGCTATGGCAATTCCTGCTGGAGTTACTGACTGACAAGTCCTGCCAGTCATTTATTAGTTGGACTGGAGA : 461

ETS2 : CGGATGGGAGTTCAAACTTGCTGACCCAGATGAGGTGGCACGGAGGTGGGGAAGGAGGAAAAACAAGCCA : 1260
REV : CGGATGGGAGTTCAAACTTGCTGACCCAGATGAGGTGGCACGGAGGTGGGGAAGGAGGAAAAACAAGCCA : 531

ETS2 : AAAATGAACTATGAGAAACTCAGCCGAGGGCTGCGCTACTATTACGACAAGAACATCATCCACAAGACCT : 1330
REV : AAAATGAACTATGAGAAACTCAGCCGAGGGCTGCGCTACTATTACGACCAGAACATCATTCACAAGACCT : 601

ETS2 : CGGGGAAGCGCTACGTTTATCGCTTTGTGTGCGACCTGCAGAACTTGCTGGGGTACACAGCGGAGGAGCT : 1400
REV : CGGGGAAGCGCTACGTTTATCGCTTTGTGTGCGACCTGCAGAACTTGCTGGGGTACACAGCGGAGGAGCT : 671

ETS2 : GCATGCCATGCTGGGGGTGCAGCCTGACACCGAGGACTGA : 1440
REV : GCATGCCATGCTGGGGGTGCAGCCTGACACCGAGGACTGA : 711

G0S2
G0S2 : ATGGAAACCATGCACGAGCTGATCCCCTTCGCCAAAGAGATGCTCAGCCAGAAGCCCAACAGGAAGATGG : 70
FWD : ATGGAAACCATGCACGAGCTGATCCCCTTCGCCAAAGAGATGCTCAGCCAGAAGCCCAACAGGAAGATGG : 70

G0S2 : TGAAGCTGTACGTGCTGGGCAGCGTGCTGGCGTTCTTCGGCGTGGTCATCGGTCTGGTGGAGGCAGTGTG : 140
FWD : TGAAGCTGTACGTGCTGGGCAGCGTGCTGGCATTCTTCGGCGTGGTCATCGGTCTGGTGGAGGCAGTGTG : 140

G0S2 : CAGCCCTTTCACCTCCGAAGGGAATATAGAGGAGGAGAAGAGACCGAGCCCATCTCGAGAGCCAGCGCTT : 210
FWD : CAGCCCTTTCACCTCCGAAGGGAATATAGAGGAGGAGAAGAGACCGAGCCCATCTCGAGAGCCAGCGCTT : 210

G0S2 : CCTCGGAAGCGGGAGGATTTGGTGTTGGAGCAGAGCAAGAAGTCGTCAGCGGTGCAGCGGGGGGTGGTGA : 280
FWD : CCTCGGAAGCGGGAGGATTTGGTGTTGGAGCAGAGCAAGAAGTCATCAGCGGTGCAGCGGGGGGTGGTGA : 280

G0S2 : CCAGGCAGCATGCATCCTAA : 300
FWD : CCAGGCAGCATGCATCCTAA : 300

GCH1

REV : TACGCACGGCGGGAGGGACCGCCCTCCCCCAAGCTGGGCACCGAGAAGCCGCGTGTATCCGCCGGTAGCG : 70
GCH1 : TACGCACGGCGGGAGGGACCGCCCTCCCCCAAGCTGGGCACCGAGAAGCCGCGTGTATCCGCCGGTAGCG : 70
FWD : TACGCACGGCGGGAGGGACCGCCCTCCCCCAAGCTGGGCACCGAGAAGCCGCGTGTATCCGCCGGTAGCG : 70

REV : GCGGTAGCGGGGACGGGTGGCGGGGAGAGCGGCCCCGCAGCGAGGAGGACAACGAGCTGAGCCTGCCCAG : 140
GCH1 : GCGGTAGCGGGGACGGGTGGCGGGGAGAGCGGCCCCGCAGCGAGGAGGACAACGAGCTGAGCCTGCCCAG : 140
FWD : GCGGTAGCGGGGACGGGTGGCGGGGAGAGCGGCCCCGCAGCGAGGAGGACAACGAGCTGAGCCTGCCCAG : 140

REV : CCTGGCGGCCGCGTACACCACCATCCTGAGGGCGCTGGGCGAGGACCCCGAGCGGCAGGGGCTGCTGAAG : 210
GCH1 : CCTGGCGGCCGCGTACACCACCATCCTGAGGGCGCTGGGCGAGGACCCCGAGCGGCAGGGGCTGCTGAAG : 210
FWD : CCTGGCGGCCGCGTACACCACCATCCTGAGGGCGCTGGGCGAGGACCCCGAGCGGCAGGGGCTGCTGAAG : 210

REV : ACGCCCTGGAGGGCGGCCACCGCCATGCAGTTCTTCACCAAGGGCTACCAGGAGACCATCGCGGATGTGC : 280
GCH1 : ACGCCCTGGAGGGCGGCCACCGCCATGCAGTTCTTCACCAAGGGCTACCAGGAGACCATCGCGGATGTGC : 280
FWD : ACGCCCTGGAGGGCGGCCACCGCCATGCAGTTCTTCACCAAGGGCTACCAGGAGACCATCGCGGATGTGC : 280

REV : TGAATGATGCCATCTTTGACGAAGACCACGATGAGATGGTAATTGTGAAGGACATAGACATGTTCTCATT : 350
GCH1 : TGAATGATGCCATCTTTGACGAAGACCACGATGAGATGGTAATTGTGAAGAACATAGACATGTTCTCATT : 350
FWD : TGAATGATGCCATCTTTGACGAAGACCACGATGAGATGGTAATTGTGAAGGACATAGACATGTTCTCATT : 350

REV : GTGTGAGCATCACCTCGTTCCATTTGTTGGAAAGGTACATATTGGCTATCTTCCTAACAAACAAGTACTT : 420
GCH1 : GTGTGAGCATCACCTCGTTCCATTTGTTGGAAAGGTACATATTGGCTATCTTCCTAACAAACAAGTACTT : 420
FWD : GTGTGAGCATCACCTCGTTCCATTTGTTGGAAAGGTACATATTGGCTATCTTCCTAACAAACAAGTACTT : 420

REV : GGCCTCAGCAAGCTCGCGAGGATTGTGGAAATATACAGTAGAAGACTACAAGTCCAGGAACGCCTTACCA : 490
GCH1 : GGCCTCAGCAAGCTCGCGAGGATTGTGGAAATATACAGTAGAAGACTACAAGTCCAGGAACGCCTTACCA : 490
FWD : GGCCTCAGCAAGCTCGCGAGGATTGTGGAAATATACAGTAGAAGACTACAAGTCCAGGAACGCCTTACCA : 490

REV : AACAAATTGCAATAGCCATCACAGAAGCCTTACAGCCCGCTGGAGTTGGAGTGGTGATCGAAGCTACGCA : 560
GCH1 : AACAAATTGCAATAGCCATCACAGAAGCCTTACAGCCCGCTGGAGTTGGAGTGGTGATCGAAGCTACGCA : 560
FWD : AACAAATTGCAATAGCCATCACAGAAGCCTTACAGCCCGCTGGAGTTGGAGTGGTGATCGAAGCTACGCA : 560

REV : TATGTGTATGGTAATGCGTGGGGTACAGAAAATGAACAGTAAAACGGTAACCAGCACAATGTTGGGGGTA : 630
GCH1 : TATGTGTATGGTAATGCGTGGGGTACAGAAAATGAACAGTAAAACGGCAACCAGCACAATGTTGGGGGTA : 630
FWD : TATGTGTATGGTAATGCGTGGGGTACAGAAAATGAACAGTAAAACGGTAACCAGCACAATGTTGGGGGTA : 630

REV : TTCCGGGAAGACCCAAAGACCCGTGAAGAGTTCTTGACGCTCATCAGGAGCTGA : 684
GCH1 : TTCCGGGAAGACCCAAAGACCCGTGAAGAGTTCTTGACGCTCATCAGGAGCTGA : 684
FWD : TTCCGGGAAGACCCAAAGACCCGTGAAGAGTTCTTGACGCTCATCAGGAGCTGA : 684

GLUL
GLUL : ATGGCCACCTCGGCGAGCTCCCACCTGAGCAAAGCCATCAAGCACATGTACATGAAGCTGCCGCAGGGTG : 70
FWD : ATGGCCACCTCGGCAAGCTCCCACCTGAGCAAAGCCATCAAGCACATGTACATGAAGCTGCCGCAGGGGG : 70

GLUL : AGAAGGTCCAAGCCATGTACATCTGGATCGACGGGACTGGGGAGCACCTCCGCTGCAAAACCCGCACTCT : 140
FWD : AGAAGGTCCAAGCCATGTACATCTGGATCGACGGGACTGGGGAGCACCTCCGCTGCAAAACCCGCACGCT : 140

GLUL : GGACCACGAACCCAAGAGCCTGGAAGATCTCCCCGAGTGGAACTTTGATGGCTCCAGCACCTTCCAAGCC : 210
FWD : GGACCACGAACCCAAGAGCCTGGAAGATCTCCCCGAGTGGAACTTTGATGGCTCCAGCACCTTCCAAGCC : 210

GLUL : GAAGGCTCCAACAGCGACATGTACCTGCGACCTGCTGCCATGTTCCGGGACCCTTTTCGCAAGGATCCCA : 280
FWD : GAAGGCTCCAACAGCGACATGTACCTGCGACCTGCTGCCATGTTCCGGGACCCTTTTCGCAAGGATCCCA : 280

GLUL : ACAAATTAGTTCTCTGTGAGGTCTTCAAATACAACCGCCAGTCTGCAGACACAAATCTTCGGCACACCTG : 350
FWD : ACAAATTAGTTCTCTGTGAGGTCTTCAAATACAACCGCCAGTCTGCAGACACAAATCTTCGGCACACCTG : 350

GLUL : TAGGCGGATTATGGATATGGTGTCCAACCAGCACCCCTGGTTTGGGATGGAGCAGGAGTACACCCTTCTG : 420
FWD : TAGGCGGATTATGGATATGGTGTCCAACCAGCACCCCTGGTTTGGGATGGAGCAGGAGTACACCCTTCTG : 420

GLUL : GGAACAGATGGTCATCCGTTTGGCTGGCCTTCCAATTGCTTCCCTGGACCCCAAGGTCCGTACTACTGCG : 490
FWD : GGAACAGATGGTCATCCGTTTGGCTGGCCTTCCAATGGCTTCCCTGGACCCCAAGGTCCGTACTACTGCG : 490

GLUL : GTGTAGGAGCTGACAAAGCCTATGGCAGAGACATTGTGGAGGCCCACTACCGAGCGTGCCTGTATGCTGG : 560
FWD : GTGTAGGAGCTGACAAAGCCTATGGCAGAGACATTGTGGAGGCCCACTACCGAGCGTGCCTGTATGCTGG : 560

GLUL : TGTGAAAATTGGAGGAACCAACGCAGAAGTGATGCCAGCCCAGTGGGAGTTCCAGGTGGGACCGTGCGAA : 630
FWD : TGTGAAAATTGGAGGAACCAACGCAGAAGTGATGCCAGCCCAGTGGGAGTTCCAGGTGGGACCGTGCGAA : 630

GLUL : GGGATTGAGATGGGGGATCACCTCTGGATAGCACGTTTCATCCTCCACCGGGTGTGCGAAGACTTTGGTG : 700
REV : GGGATTGAGATGGGGGATCACCTCTGGATAGCACGCTTCATCCTCCACCGAGTGTGCGAAGACTTTGGTG : 205

GLUL : TCATTGTGTCCTTCGATCCCAAACCCATCCCTGGGAACTGGAACGGTGCTGGCTGTCACACCAACTTCAG : 770
REV : TCATTGTGTCCTTCGATCCCAAACCTATCCCTGGGAACTGGAACGGTGCTGGCTGTCACACCAACTTCAG : 275

GLUL : CACCAAGAACATGAGGGAAGATGGAGGTCTCAAGCACATCGAGGAGGCCATCGAGAAGCTGAGCAAGCGC : 840
REV : CACCAAGAACATGAGGGAAGATGGAGGTCTCAAGCACATCGAGGAGGCCATCGAGAAGCTGAGCAAGCGC : 345

GLUL : CACCAGTACCACATCCGTGCCTACGACCCCAAAGGAGGGCTGGACAACGCCCGGCGCCTGACGGGCTTCC : 910
REV : CACCAGTACCACATCCGTGCCTACGACCCCAAAGGAGGGCTGGACAACGCCCGGCGCCTGACGGGCTTCC : 415

GLUL : ACGAGACGTCCAGCATCCACGAGTTCTCCGCCGGCGTGGCCAACCGCGGCGCCAGCATCCGCATCCCACG : 980
REV : ACGAGACGTCCAGCATCCACGAGTTCTCCGCCGGCGTGGCCAACCGCGGCGCCAGCATCCGCATCCCACG : 485

GLUL : CAACGTGGGCCATGAGAAGAAAGGCTACTTCGAGGACCGCGGGCCTTCAGCCAACTGCGATCCCTACGCC : 1050
REV : CAACGTGGGCCATGAGAAGAAAGGCTACTTCGAGGACCGCCGGCCTTCAGCCAACTGCGATCCCTACGCC : 555

GLUL : GTGACGGAGGCCCTGGTCCGTACGTGTCTCCTCAACGAAACCGGGGACGAGCCTTTTGAGTACAAGAACT : 1120
REV : GTGACGGAGGCCCTGGTCCGTACGTGTCTCCTCAACGAAACCGGGGACGAGCCTTTTGAGTACAAGAACT : 625

GLUL : AA : 1122
REV : AA : 627

HPS5
HPS5 : ATGAGGCTCTGTATCTGCTTCGTGTTGCTCTCCATTGTTCTGTATGCAAGTGCAGACAACCCATTCTCCA : 70
FWD : ATGAGGCTCTGTATCTGCTTCGTGTTGCTCTCCATTGTTCTGTATGCAAGTGCAGACAACCCATTCTCCA : 70
REV : ATGAGGCTCTGTATCTGCTTCGAGTTGCTCTCCATTGTTCTGTATGCAAGTGCAGACAACCCATTCTCCA : 70

HPS5 : GCGGTATCAAGTTTGTCAGGGATGCAGCTGGAGGCGCACGGGATATGTGGAGAGCATACCGGGACATGCG : 140
FWD : GCGGTATCAAGTTTGTCAGGGATGCAGCTGGAGGCGCACGGGATATGTGGAGAGCATACCGGGACATGCG : 140
REV : GCGGTATCAAGTTTGTCAGGGATGCATCTGGAGGCGCCCGGGATATGTGGAGAGCATACCGGGACATGCG : 140

HPS5 : TGAGGCAAACTACATTGGTGCTGACAAGTATTTCCATGCTCGTGGCAATTACGATGCTGCTCGGAGAGGA : 210
FWD : TGAGGCAAACTACATTGGTGCTGACAAGTATTTCCATGCTCGTGGCAATTACGATGCTGCTCGGAGAGGA : 210
REV : TGAGGCAAACTACATTGGTGCTGACAAGTATTTCCTTGCTCGTGGCAATTACGATGCTGCTCGGACAGGA : 210

HPS5 : CCTGGAGGTGCTTGGGCAGCCAAAGTGATCAGCGATGCCCGGGAGGGCTGGCAGAGCAGGGTGAGCGGCA : 280
FWD : CCTGGAGGTGCTTGGGCAGCCAAAGTGATCAGCGATGCCCGGGAGGGCTGGCAGAGCAGGGTGAGCGGCA : 280
REV : CCTGGAGGTGCTTGGGCACCCAAAGTGATCAGCGATGCCCGGGAGGGCTGGAAGAGCAGGGTGAGCGGTA : 280

HPS5 : GAGGCGCGGAGGACACCCGGCTCGACCAGGAGGCCAACGAGTGGGGCAGGAGAGGCGGCGATCCCAACCG : 350
FWD : GAGGCGCGGAGGACACCCGGCTCGACCAGGAGGCCAACGAGTGGGGCAGGAGAGGCGGCGATCCCAACCG : 350
REV : TAGGCGCGGAGGACACCCGGCTCAACCAGAAGGCCAACAAGTGGGGCAGGAAAGGCGGCGATCCCAACCG : 350

HPS5 : CTTCAGGCCCGCAGGACTTCCTAACAAATACTGA : 384
FWD : CTTCAGGCCCGCAGGGCTTCCTAACAAATACTGA : 384
REV : CTTCAGGCCCGCAGGGCTTCCTAACAAATACTGA : 384

IFIT5
IFIT5 : ATGAGTACCATTTCCAAGAATTCCTTGAAGAACTCCCTGCTGCAGCTAGAATGTTATTTTACATGGACTT : 70
FWD : ATGAGCCCCATTTCCAAGAATTCCTTGAAGAACTCCCTGCTGCAGCTAGAATGTTATTTTACATGGACTT : 70

IFIT5 : TGCTGAAGGAGGATGTAGATCTTGACAGTCTGGAAGAATCAATAGAGGATCAGATTGAGTTTTTCATAAA : 140
FWD : TGCTGAAGGAGGATGTAGATCTTGACAGTCTGGAAGAATCAATAGAGGATCAGATTGAGTTTTTCATAAA : 140

IFIT5 : ACCCAACATTTCAAATTACAATCTACTATCTTATGTATACCACCTAAAGCTCTCAGATGAAGCAGCTCTG : 210
FWD : ACCCAACATTTCAAATTACAATCTACTATCTTATGTATACCACCTAAAGCTCTCAGATGAAGAAGCTCTG : 210

IFIT5 : GAATATCTCCAAAAAGCTGAAGAAGAAATTAAAAAATACTATCCAGGTGAAATTGACAGGAGAAGTCTCG : 280
FWD : GAATATCTCCAAAAAGCTGAAGAAGAAATTAAAAAATACTATCCAGGTGAAATTGACAGGAGAAGTCTCG : 280

IFIT5 : TTACCTGGGGGAACTACGCTTGGATCTACTACCACATGGGCAGATATGAAGAAGCTCAAGTGTATATAAA : 350
FWD : TTACCTGGGGGAACTACGCTTGGATCTACTACCACATGGGCAGATATGAAGAAGCTCAAGTGTATATAAA : 350

IFIT5 : TAAAGTGGAAAACAGCTGCAAAAAGCTTTCAAATACTGCTCATTTGAAGATTCAACTTCCAGAGATCTAT : 420
FWD : TAAAGTGGAAAACAGCTGCAAAAAGCTTTCAAATACTGCTCATTTGAAGATTCAGCTTCCAGAGATCTAT : 420

IFIT5 : GCTGAGCAAGGATTTGCACTATTAAAATTTGGAGGAAAGTACTATAACAGAGCGAAAGAGTGCTTCAAAA : 490
FWD : GCTGAGCAAGGATTTGCACTATTAAAATTTGGAGGAAAGTACTATAACAGAGCAAAAGAGTGCTTCAAAA : 490

IFIT5 : ATGCTCTGAGGGAAGAACCCAACAACCCAGAATTTAATGCCGGCTATGCAATAGCAGTGTATCGTTTGGA : 560
FWD : ATGCTCTGAGGGAAGAACCCAACAACCCAGAATTTAATGCCGGTTATGCAATAGCAGTGTATCGTTTGGA : 560

IFIT5 : AGAATTTTCTT.ACAGAAGATGTGAA.GAAGTGGACTCATCCCTGGAGCCTCTGAAGCGTGCACTGAAAC : 628
FWD : AGAATTTTCTTTACAGAAGATGTGAAAGAAGTGGACT................................. : 597

IFIT5 : AATCCAATGGACACTTATCTTTTGGCTTTACTTGCATTGAAACTTCAGGATTCAGATCAAGTTGATGAAG : 700
REV : ................................................GATTCAGATCAAGTTGATGAAG : 22

IFIT5 : CAGAGAAA.TGCATCGAAGAAGGAATGAAGAAAACCCCTTATCTTCCCTACTTCCTGAGATATGCTGCTA : 769
REV : CAGAGAAAATGCAT.GAAGAAGGAATGAAGAAAACCCCTTATCTTCCCTACTTCCTGAGATATGCTGCTA : 91

IFIT5 : AATTCTACAGAAGGAAAAAAGAACTGGACAA.GGCACAAGAGGTTTTGGAGAGAGCCCTAGAAATATCAC : 838
REV : AATTCTACAGAAGGAAAAAAAAACTGGACAAAGGCACAAGAGGTTTTGGAGAGAGCCCTAGAAATATCAC : 161

IFIT5 : CAAAATCTACCTTTTTGCTTCACCAGCTAGGACTCTGCTATCGAGCAAAGCTGTATGAGTTGAAAAACAG : 908
REV : CAAAATCTACCTTTTTGCTTCACCAGCTAGGACTCTGCTACCGAGCAAAGCTGTATGAGTTGAAAAACAG : 231

IFIT5 : TACAAGATATCCACCTCAAGATCAAATAGAAGAGCTCATTCAAATTTGCATTTCTCATTTCAAAGTGGTG : 978
REV : TACAAGATATCCACCTCAAGATCAAATAGAAGAGCTCATTCAAATTTGCATTTCTCATTTCAAAGTGGTG : 301

IFIT5 : ACAGAGCAAAAGCCAAAATTTTTTAGTGCCCTAATTGACTTGGCAAGGATGTATGCGGAGGCAAATATGT : 1048
REV : ACAGAGCAAAAGCCAAAATTTTTTAGTGCCCTAATTGACTTGGCAAGGATGTATGCGGAGGCAAATATGT : 371

IFIT5 : ATCAAAAAGCAGAAGAGACGTTTCAGAAAGCCCTGAATGTAAATATTCTGACTTGCTCCGATAAACAAGA : 1118
REV : ATCGAAAAGCAGAAGAGACGTTTCAGAAAGCCCTGAATGTAAATATTCTGACTTGCTCCAATAAACAAGA : 441

IFIT5 : AATATACTATTTTTATGGAAATTTTCTGCAATATAAAAAGAAATCAGAATCTGAAGCAATTAAATATTAC : 1188
REV : AATATGCTATTTTTATGGAAATTTTCTGCAATATAAAAAGAAATCAGAATCTGAAGCAATTAAATATTAC : 511

IFIT5 : AAAGAGGGGCTAAAAAATGGTAACTACTGTTTTGCAGAGAAGATCAGACAATACCTAAAGAGACTGTTGG : 1258
REV : AAAGAGGGGCTAAAAAATGGTAACTACTGTTTTGCAGAGAAGATCAGACAATACCTAAAGAGACTGTTGG : 581

IFIT5 : AAAAGAGAATTCAAGGAGGATTAGGAGGTGAAGATGATTTCAGTACACTTGGACTCATTCATAAACTAGA : 1328
REV : AAAAGAGAATTCAAAGAGGATTAGGAGGTGAAGATGATTTCAGTACACTTGGACTCATTCATAAACTAGA : 651

IFIT5 : TGGTGAGAAGCTTGAAGCAATTGAATGTTATGAGAAAGCCAATGAATATAATCCAGACAATGAAGAATAT : 1398
REV : TGGTGAGAAGCTTGAAGCAATTGAATGTTATGAGAAAGCCAATGAATATAATCCAGACAATGAAGAATAT : 721

IFIT5 : CTGAGTGTGTTATTGGAGCTACGACTTTCCCTCTCAAGCTGA : 1440
REV : CTGAGTGTGTTATTGGAGCTACGACTTTCCCTCTCAAGCTGA : 763

IL1R2
IL1R2 : ATTTCAAGACACTTTCCCTCGCTCTTCTTCCCATTTCCTCTGGGAAAGAATGCATGGGTTCTTCTTTGCC : 70
FWD : ATTTCAAGACACTTTCCCTCGCTCTTCTTCCCATTTCCTCTGGGAAAGAATGCATGGGTTCTTCTTTGCC : 70

IL1R2 : CTGGTTACATGCACGATGTGTGCCTCTGCTTTCAAGCTCCAGCAAGGAAAAAGCACAGAAAACTGCCCAG : 140
FWD : CTGGTTACATGCACGATGTGTGCCTCTGCTTTCAAGCTCCAGCAAGGAAAAAGCACAGAAAACTGCCCAG : 140

IL1R2 : ATCACACCATGTTTTTCCAATACTACTATGAACTGCACGGAGAACCTGTGGTTCTGAAATGCCCTTCCCC : 210
FWD : ATCACACCATGTTTTTCCAATACTACTATGAACTGCACGGAGAACCTGTGGTTCTGAAATGCCCTTCCCC : 210

IL1R2 : CAGATACAAACATGTGGATTTTTCTGCCTTGACACATAATATCACATGGTACAAAAATGATTCAAAAACC : 280
FWD : CAGATACAAACATGTGGATTTTTCTGCCTTGACACATAATATCACATGGTACAAAAATGATTCAAAAACC : 280

IL1R2 : ATGATATTAGGAAGAGATGAAGATCCAAGAATCTGGGCAAAAGGAGACGCGCTCTGGTTTTTGCCAGCAA : 350
FWD : ATGATATTAGGAAGAGATGAAGATCCAAGAATCTGGGCAAAAGGAGACGCGCTCTGGTTTTTGCCAGCAA : 350

IL1R2 : GGCAAGAAGACTCAGGAGTGTATATCTGTACCAGAAGGAACAACTCCTACTGTGCCGAGGTGTCCATCCA : 420
FWD : GGCAAGAAGACTCAGGAGTGTATATCTGTACCAGAAGGAACAACTCCTACTGTGCCGAGGTGTCCATCCA : 420

IL1R2 : GCTCACGGTCGTGCAGAAGGCAGCTGCTCGGGACATCGCCTATCCCCAGATCCTCTTCACTTTCACTTCT : 490
FWD : GCTCACGGTTGTGCAGAAGGCAGCTGCTCGGGACATCGCCTATCCCCAGATCCTCTTCACTTTCACTTCT : 490

IL1R2 : GGAAAGATCGTTTGCCCCAATCTGTGGGAGTTTACAATAAACAGGACAAACATGGAGCTCCAGTGGTACA : 560
FWD : GGAAAGATCGTTTGCCCCAATCTGTGGGAGTTTACAATAAACAGGACAAACATGGAGCTCCAGTGGTACA : 560

IL1R2 : AGGATGCTCTGCTTTTGGAAGATGACAGTGAAAAATTTGTATTTCTGAAAGAGTCGTCTTCCCTGTTCTT : 630
FWD : AGGATGCTCTGCTTTTGGAAGACGACAGTGAAAAATTTGTATTTCTGAAAGAGTCTTCTTCCCTGTTCTT : 630
REV : AGGATGCTCTGCTTTTGGAAGACGACAGTGAAAAATTTGTATTTCTGAAAGAGTCTTCTTCCCTGTTCTT : 178

IL1R2 : CACTTCTGTATTACCGACAGACTCCGGCTACTACACCTGCAAGATGGCTTTTCCATATGAAGGTGTCACA : 700
REV : CACTTCTGTATTACCGACAGACTCCGGCTACTACACCTGCAAGATGGCTTTTCCATATGAAGGTGTCACA : 248

IL1R2 : TATGAAATCACCAGAACAATTAAACTGCAGACTGTTGAACAAGAAAAGAGAATTACCCCCATAATTGTAT : 770
REV : TATGAAATCACCAGAACAATTAAACTGCAGACTGTTGAACAAGAAAAGAGAATTACCCCCATAATTGTAT : 318

IL1R2 : ATCCAACTCAGAAGACAACAACAGCTGCTCTTGGCTCCAAGATGACTCTCCCATGTAAAGTATTTGCTGG : 840
REV : ATCCAACTCAGAAGACAACAACAGCTGCTCTTGGCTCCAAGATGACTCTCCCATGTAAAGTATTTGCTGG : 388

IL1R2 : ACCGAACAGCAAGGTCTATACTGACGTGGAATGGTTAGCGAATGATACCACGATCGATGTGGTTTACAAG : 910
REV : ACCGAACAGCAAGGTCTATACTGACGTGGAATGGTTAGCGAATGATACCACGATCGATGTGGTTTACAAG : 458

IL1R2 : CAAAACAGAGTTATGGAGGGGGAGCGACAAGAAACCACAGAAAATGGTGAGAACTTCATTGAAGTGCCAC : 980
REV : CAAAACAGAGTAATGGAGGGGGAGCGACAAGAAACCACAGAAAATGGTGAGAACTTCATTGAAGTGCCAC : 528

IL1R2 : TGATTTTTAAATCCGTGGAAGAAGTGGATTTTTACACAGATTTTAAGTGTCTGGCCCAGAACAGATATGG : 1050
REV : TGATTTTTAAATCCGTGGAAGAAGTGGATTTTTACACAGATTTTAAGTGTCTGGCCCAGAACAGATATGG : 598

IL1R2 : ATCTCAAGTACTGCCAACACGGGTCAAGCAGGAAGCTGTCGGCTTGCCCTGGTACATTGCAACAATTCCA : 1120
REV : ATCTCAAGTACTGCCAACACGGGTCAAGCAGGAAGCTGTCGGCTTGCCCTGGTACATTGCAACAATTCCA : 668

IL1R2 : GTCGCATTGGCCTGTGTCATCCTGGCAGG : 1149
REV : GTCGCATTGGCCTGTGTCATCCTGGCAGG : 697

IL4I1
IL4I1 : ATGGCTGCGATGGTTCTCTTCCAAATGCTCCTCCTACTGGGACTCGCCAGCGCCAAGAGGTTCTCGTGCT : 70
FWD : ATGGCTGCGATGGTTCTCTTCCAAATGCTCCTCCTACTGGGACTCGCCAGCGCCAAGAGGTTCTCGTGCT : 70

IL4I1 : TCCCTGAGTATTGCCTCCATGACAAAGACTACGAAGAGCTGCTGAGTATTGTGAAATATGGGCTGGAGCC : 140
FWD : TCCCTGAGTATTGCCTCCATGACAAAGACTACGAAGAGCTGCTGAGTATTGTGAAATATGGGCTGGAGCC : 140

IL4I1 : CACGACGCGTCCATCGAATGTGGTCATCGTTGGGGCTGGGATCAGCGGGCTGACAGCAGCCAAACTGCTC : 210
FWD : CACGATGCGTCCATCGAATGTGGTCATCGTTGGGGCTGGGATCAGCGGGCTGACAGCAGCCAAACTGCTC : 210

IL4I1 : CGAGACGCCGGCCATAAGGTCACGATTCTGGAAATCAGCAATAGGGTTGGTGGGAGGATCAGGACCTACC : 280
FWD : CGAGACGCCGGCCATAAGGTCACCATTCTGGAAATCAGCAATAGGGTTGGTGGGAGGATCAGGACCTACC : 280

IL4I1 : GGGAGAAGGACTGGTATGTGGAGCTGGGAGCCATGCGCCTGCCGAGCAAGCACAGGCTGGTGCGTGAGTT : 350
FWD : GGGAGAAGGACTGGTATGTGGAGCTGGGAGCCATGCGCCTGCCGAGCAAGCACAGGCTGGTGCGTGAGTT : 350

IL4I1 : TATAAGGCAGTTTGACCTGAAGCTCAACCCGTTCCGCCAGACAGATGACAATGCCTGGTACTTCGTGAAC : 420
FWD : TATAAGGCAGTTTGACCTGAAGCTCAACCCGTTCCGCCAGACGGATGACAACGCCTGGTACTTCGTGAAC : 420

IL4I1 : GGCATCCGAGCGAGAGTGGCAGAAGTGAACAGAAACCCCGACATCCTGAATTACACCCTCAGGCCGTCAG : 490
FWD : GGCATCCGAGCGAGAGTGGCAGAAGTGAACAGAAACCCCGACATCCTGAATTACACCCTCAGGCCGTCAG : 490

IL4I1 : AGAGGGGCAAAAGTGCCAGCCAGCTCTACAGGGAAGCCCTGCAGTCGGCTGTCAAGGAGTTCCAGACCAT : 560
FWD : AGAGGGGCAAAAGTGCCAGCCAGCTCTACAGGGAAGCCCTGCAGTCGGCTGTCAAGGAGTTCCAGACCAT : 560

IL4I1 : GAACTGCAAGGAGTATCTGGCTAAACACGACTCCTT.TCCACCAAGGAATATCTGATTAAAGTCGGGAAT : 629
FWD : GAACTGCAAGGAGTATCTGGCTAAACACGACTCCTTCTCCACCAAGGAATATCTGATTAAAGTCGGGAAT : 630

IL4I1 : TGAGCCGAGGAGCTGTGCAGATGATCGGAGATTTGCTGAACGAGGACTCGGGGTTTTACCTGTCCTTCCT : 700
REV : TGAGC.GAGGAGCTGTGCAGATGATCGGAGATT.GCTGAACGAGGACTCGGGGTTTTACCTGTCCTTCCT : 117

IL4I1 : TGCCTCGCTGTGGGATTTTGATATCTTCTCTAACGGGGAGAGCTTTGACGAAATCACGGGAGGTTTCGAC : 770
REV : TGCCTCGCTGTGGGATTTTGATATCTTCTCTAACGGGGAGAGCTTTGACGAAATCACGGGAGGTTTCGAC : 187

IL4I1 : CAGCTGCCCAAAGCCTTCCACAAAGCACTGCCGGGCGTCATCAAATTCAATTGCACGGTGGAGAAAATCA : 840
REV : CAGCTGCCCAAAGCCTTCCACAAAGCACTGCCGGGCGTCATCAAATTCAATTGCACGGTGGAGAAAATCA : 257

IL4I1 : TGACGAAGGGAGGGAAGGTCCGGGTGTTCTACCGCGCTCCGGACACCCTCGCCCCCAGCATAGTGACCGC : 910
REV : TGACGAAGGGAGGGAAGGTCCGGGTGTTCTACCGCGCTCCGGACACCCTCGCCCCCAGCATAGTGACCGC : 327

IL4I1 : AGATTACGTCCTGGTCACCTCCTCGGCCAAAGCCACGAGGCACATCCAGTTCTTCCCACCTCTCTCCCCT : 980
REV : AGATTACGTCCTGGTCACCTCCTCGGCCAAAGCCACGAGGCACATCCAGTTCTTCCCACCTCTCTCCCCT : 397

IL4I1 : GCCAAAAACGAGGCTCTCCGCTCCATCCACTACGCGAGCGCTTCCAAAATCGCCTTGGCATGCACGGAGA : 1050
REV : GCCAAAAACGAGGCTCTCCGCTCCATCCACTACGCGAGCGCTTCCAAAATCGCCTTGGCATGCACGGAGA : 467

IL4I1 : AGTTCTGGGAGAAGGACGGCATTCAAGGAGGGCAATCCATAACCGACCGCCCCTCCAGGTTTATCTACTA : 1120
REV : AGTTCTGGGAGAAGGACGGCATTCAAGGAGGGCAATCCATAACCGACCGCCCCTCCAGGTTTATCTACTA : 537

IL4I1 : CCCCAGCCACAACTTCTCCAGTGGGGTCGGGGTCATCTTGGCCTCCTACACCTGGAACGACGACGCTGAG : 1190
REV : CCCCAGCCACAACTTCTCCAGTGGGGTCGGGGTCATCTTGGCCTCCTACACCTGGAACGACGACGCTGAG : 607

IL4I1 : TTCCTCATACCCTTAGCGGATGAGAAATACTTGGACGTGGTTCTCCAAGACTTGTCGGACATCCACCGGG : 1260
REV : TTCCTCATACCCTTAGCGGATGAGAAATACTTGGACGTGGTTCTCCAAGACTTGTCGGACATCCACCGGG : 677

IL4I1 : TGAGCAAGAGTTACCTGCAGTACACCTGCGACCGCTACG.GATACAGAAGTGGCAGCTGGACAGGCATTC : 1329
REV : TGAGCAAGAGTTACCTGCAGTACACCTGCGACCGCTACGTGATACAGAAGTGGCAGCTGGACAGGCATTC : 747

IL4I1 : CCTGGGAGCCTTCGCTGCCTTCACGCCGTATCAGTTCACCGATTACTCCCAGGCGCTCTTTGAGCACGAG : 1399
REV : CCTGGGAGCCTTCGCTGCCTTCACGCCGTATCAGTTCACCGATTACTCCCAGGCGCTCTTTGAGCACGAG : 817

IL4I1 : GGGAGGGTGCACTTTGCAGGGGAGCACACCGCCCAGCCTCACGCATGGATCGACACCTCCATGAAGGC.G : 1468
REV : GGGAGGGTGCACTTTGCAGGGGAGCACACCGCCCAGCCTCACGCATGGATCGACACCTCCATGAAGGCTG : 887

IL4I1 : CGGTGAGGGCAGCGAGCAACATCCACGACGACAGCGAAACCCGAAATCTCAGCCCTAACCACGTACACCT : 1538
REV : CGGTGAGGGCAGCGAGCAACATCCACGACGACAGCGAAACCCGAAATCTCAGCCCTAACCACGTACACCT : 957

IL4I1 : GGAGAGCTTCGTGGAGAAGGGAGAGCTCTGA : 1569
REV : GGAGAGCTTCGTGGAGAAGGGAGAGCTCTGA : 988

IL10RA
IL10RA : ATGGCCCTCTGCGCTGCCCTGGCCCTGTGCGTGACTCTGCTGCTCACTCAACCCGCACATGGTGAGCTGC : 70
REV : ...................................................................... : -
FWD : ATGGCCCTCTGCGCTGCCCTGGCCCTGTGCGTGACTCTGCTGCTCACTCAACCTGCACATGGTGAGCTGC : 70

IL10RA : GGTTAAAGCCCACGCGCGTGCGCTTCGTGGCGGAGATGGTGTACCACCTGCTGCAGTGGGAGCCAGGCCG : 140
REV : ...................................................................... : -
FWD : GGTTAAAGCCCACGCGCGTGCGCTTCGTGGCGGAGATGGTGTACCACCTGCTGCAGTGGGAGCCAGGCCG : 140

IL10RA : TGATGCTCCCAGCGACACCCGCTACGACGTGGAGCACAAAATCTATGGCACCAACAGCCCCTGGACCGCC : 210
REV : ...................................................................... : -
FWD : TGATGCTCCCAGCGACACCCGCTACGACGTGGAGCACAAAATCTATGGCACCAACAGCCCCTGGACCGCC : 210

IL10RA : ATCCCGAACTGCATGAAGATCCACGGGCACAGCTGTGACCTCACCTACTACACCCTGGATCCCTCCCTGC : 280
REV : ...................TCCACGGGCACAGCTGTGACCTCACCTACTACACCCTGGATCCCTCCCTGC : 51
FWD : ATCCCGAACTGCATGAAGATCCACGGGCACAGCTGTGACCTCACCTACTACACCCTGGATCCCTCCCTGC : 280

IL10RA : GCTATTACGCCCGGGTGCGAGCTGTGGTTGGGAACCACACATCTGACTGGAAAAGAACCAATGCCTTCTC : 350
REV : GCTATTACGCCCGGGTGCGAGCTGTGGTTGGGAACCACACATCTGACTGGAAAAGAACCAATGCCTTCTC : 121
FWD : GCTATTACGCCCGGGTGCGAGCTGTGGTTGGGAACCACACATCTGACTGGAAAAGAACCAATGCCTTCTC : 350

IL10RA : CCCGCAGGAAGCCAGCCTGCGCCTGTCTGGCCACAGCCTGGCCGTGACGGACAACAGCATCCATGTGCAG : 420
REV : CCCGCAGGAAGCCAGCCTGCGCCTGTCTGGCCACAGCCTGGCCGTGACGGACAACAGCATCCATGTGCAG : 191
FWD : CCCGCAGGAAGCCAGCCTGCGCCTGTCTGGCCACAGCCTGGCCGTGACGGACAACAGCATCCATGTGCAG : 420

IL10RA : CTGCAGCTGCTGCTCAGGGCAGGGAACCGCACTGTGAAGTACGACGACATCCAGAAGCATGCGAGGCGGT : 490
REV : CTGCAGCTGCTGCTCAGGGCAGGGAACCGCACTGTGAAGTACGACGACATCCAGAAGCATGCGAGGCGGT : 261
FWD : CTGCAGCTGCTGCTCAGGGCAGGGAACCGCACTGTGAAGTACGACGACATCCAGAAGCATGCGAGGCGGT : 490

IL10RA : ACCGGGTGTACATCCGGCGGGCAAGGGACAACCAGACGTATGAAGTGTGGGAGACCGCCTCAGAGTTCTA : 560
REV : ACCGGGTGTACATCCGGCGGGCAAGGGACAACCAGACGTATGAAGTGTGGGAGACCGCCTCAGAGTTCTA : 331
FWD : ACCGGGTGTACATCCGGCGGGCAAGGGACAACCAGACGTATGAAGTGTGGGAGACCGCCTCAGAGTTCTA : 560

IL10RA : CATCCGCAACCTCTTCTGGAACACTGAGTACTGCATCAGTGTGGAGCCCGACGTGGCCAGCCGGCACATC : 630
REV : CATCCGCAACCTCTTCTGGAACACTGAGTACTGCATCAGTGTGGAGCCCGACGTGGCCAGCCGGCACATC : 401
FWD : CATCCGCAACCTCTTCTGGAACACTGAGTACTGCATCAGTGTGGAGCCCGACGTGGCCAGCCGGCACATC : 630

IL10RA : CCTGCCATGCGCACCGCTGAGCAGTGCGTCACCATTGGCCACAGAGACGAAAGTGCAGAACTCATCCCGA : 700
REV : CCTGCCATGCGCACCGCTGAGCAGTGCGTCACCATTGGCCACAGAGACGAAAGTGCAGAACTCATCCCGA : 471
FWD : CCTGCCATGCGCACCGCTGAGCAGTGCGTCACCATTGGCCACAGAGACGAAAGTGCAGAACTCATCCCGA : 700

IL10RA : TCACTGTCAGCTCCTTCGCCATTGTCCTCTTCCTTCTGGGTATCCTTGGGGCTCTGCTGGTCTGCACGTA : 770
REV : TCACTGTCAGCTCCTTCGCCATTGTCCTCTTCCTTCTGGGTATCCTTGGGGCTCTGCTGGTCTGCACGTA : 541
FWD : TCACTGTCAGCTCCTTCGCCATTGTCCTCTTCCTTCTGGGTATCCTTGGGGCTCTGCTGGTCTGC..... : 765

IL10RA : CCTAAAGAAACCCATGAGGCCACCATCCGTCCTGAAGTCATTCATAAAGCAGAGCTCACTCTGGATGGAA : 840
REV : CCTAAAGAAACCCATGAGGCCACCATCCGTCCTGAAGTCATTCATAAAGCAGAGCTCACTCTGGATGGAA : 611
FWD : ...................................................................... : -

IL10RA : CAGGAGATCTCATCCTCAGGGAGCACGGATGCAGTGCAGCAGCTGTTCCTGTGCCAGAAG : 900
REV : CAGGAGATCTCATCCTCAGGGAGCACGGATGCAGTGCAGCAGCTGTTCCTGTGCCAGAAG : 671
FWD : ............................................................ : -

IL17REL
IL17REL : ATGATAAGTGTTCATGTTCTGATTCTTCTTATAATTCTATTTTGGAATTATGATTGTCATATTATTCCCA : 70
FWD : ATGATAAGTGTTCATGTTCTGATTCTTCTTATAATTCTATTTTGGAATTATGATTGTCATATTATTCCCA : 70

IL17REL : GGATTGAAGAATGTGGACTTTCATGTTCTCAGGGGATTCATTGTAAAAGCAAACCTTCCAGTGGCATTTT : 140
FWD : GGATTGAAGAATGTGGACTTTCATGTTCTCAGGGGATTCATTGTAAAAGCAAACCTTCCAGTGGCATTTT : 140

IL17REL : TAACAGCTTCTGCCACAGTGCTCCTGCATCTTTGTCTTCTATGGTACTGAAGAGCATGAAGATCTCAACA : 210
FWD : TAACAGCTTCTGCCACAGTGCTCCTGCATCTTTGTCTTCTATGGTACTGAAGAGCATGAAGATCTCAACA : 210

IL17REL : GTTATTAAATGTCCTCAAGGAAGTCCATGCTCTCTTCATTTAAACATTAAAGGAACTCTGAGTCTGGATG : 280
FWD : GTTATTAAATGTCCTCAAGGAAGTCCATGCTCTCTTCATTTAAACATTAAAGGAACTCTGAGTCTGGATG : 280

IL17REL : AAAATATTCGTGGGCTGGAAATATGTTCTTTTTCACTGGACACACAGCAATCTCAGTGCACAAATGTGAG : 350
FWD : AAAATATTCGTGGGCTGGAAATATGTTCTTTTTCACTGGACACACAGCAATCTCAGTGCACAAATGTGAG : 350

IL17REL : ATTTGCCAGGAAAAAAAACAAGATACTTAATGGAAAGAAGGTGCAGGTTCAATTCAATTGCTTTGAAGTC : 420
FWD : ATTTGCCAGGAAAAAAAACAAGATACTTAATGGAAAGAAGGTGCAGGTTCAATTCAATTGCTTTGAAGTC : 420

IL17REL : AACGTAGCACAACACATCTATGTGACCGTGAAAACAGTACCAAATTACTGTGAAGTTAAGATGAGTCAGG : 490
FWD : AACGTAGCACAACACATCTATGTGACCGTGAAAACAGTACCAAATTACTGTGAAGTTAAGATGAGTCAGG : 490

IL17REL : AATACTATGTTGAAGATTGCAGAAACAGTGATGTGGGAAAGCATATTCCAGCTTGTTTGGCTGGGAAGTT : 560
FWD : AATACTATGTTGAAGATTGCAGAAACAGTGATGTGGGAAAGCATATTCCAGCTTGTTTGGCTGGGAAGTT : 560

IL17REL : AGACTATAATGTAGACAGGACGAGAAAAATTATATCAGTGAATGTATCCAACTTTCTTCGAGATCAAGAT : 630
FWD : AGACTATAATGTAGACAGGACGAGAAAAATTATATCAGTGAATGTATCCAACTTTCTTCGAGATCAAGAT : 630

IL17REL : TATTACATTCGTTTGTGTCACAAATGGTTTACCTGTGAAGATGTGGGACAATTTGCTGTGATAAAAGGGA : 700
REV : TATTACATTCGTTTGTGTCACAAATGGTTTACCTGTGAAGATGTGGGACAATTTGCTGTGATAAAAGGGA : 76

IL17REL : AGGATTCTTTAAAATCAGTTTCTCTGAAGTACTCTCAGCTACTTCCTTGTCTTTGCATTGAGGGTTGGCT : 770
REV : AGGATTCTTTAAAATCAGTTTCTCTGAAGTACTCTCAGCTACTTCCTTGTCTTTGCATTGAGGGTTGGCT : 146

IL17REL : GGCAATTCCAGATGCCAGGAGGTTACAACTTTGTCCCTTTGAAAATGATACAAAGGCATTATGGGATAAT : 840
REV : GGCAATTCCAGATGCCAGGAGGTTACAACTTTGTCCCTTTGAAAATGATACGAAGGCATTATGGGATAAT : 216

IL17REL : ATTGTTTATAATCCAGTGACACAAACCCTAGCTTGGGAACCATCCTGCCCTGTGCTTGTCATTGTTAACC : 910
REV : ATTGTTTATAATCCAGTGACACAAACCCTAGCTTGGGAACCAGCCTGCCCTGTGCTTGTCATTGTTAACC : 286

IL17REL : TGTGCAGATTAATGAAATCTAACGACCACTGTGAAGATATACAAGATTCTTCCAAACGTTTTCCCGAGAA : 980
REV : TGTGCAGATTAATGAAGTCTAACGACCACTGTGAAGATATATAAGATTCTTCCAAACGTTTTCCCGAGAA : 356

IL17REL : GCAGGTTAAGTATTCTCGTGTGGACACTCACCCAAGACTTTGCATGAAGTTTACAACCAAGCAAGGCTCT : 1050
REV : G...GTTAAGTATTCTCGTGTGGACACTCACCCAAGACTTTGCATGAAGTTTACAACCAAGCAAGGCTCT : 423

IL17REL : TGGGTTAAATGTCCATTTGCTCATGGAGAATTTCCAGCTTGGAAGATGAGAACAGCTGCAGTTGCAGATC : 1120
REV : TGGGTTAAATGTCCATTTGCTCATGGAGAATTTCCAGCTTGGAAGATGAGAACAGCTGCAGTTGCAGATC : 493

IL17REL : GAATACAAGTTTTCTTCACATCCCAAACCAAAGCACACTTCTCAGTGCTGGTGTGTAACAGGACACAGCT : 1190
REV : GAATACAAGTTTTCTTCACATCCCAAACCAAAGCACACTTCTCAGTGCTGGTGTGTAACAGGACACAGCT : 563

IL17REL : GGCCTCATGTGAATCTGTTGGGATGCGCCATTCAGTCTCTGTGGGCTGGAGGACAGATGTAGATTATTCA : 1260
REV : GGCCTCATGTGAATCTGTTGGGATGCGCCATTCAGTCTCTGTG....GGAGATTCTGTGTCAATCATGAT : 629

IL17REL : GTTCCAC............................................................... : 1267
REV : GTCACGGGAAATGTGTGGGTCAACAATTTGCATTCAGGGCTGGAGGACAGATGTAGATTATTCAGTTCCA : 699

IL17REL : . : -
REV : C : 700

iNOS
iNOS : ATGCTGTGCCCATGGCAGTTTGCATTCAAACCTCATGCTGTTAAGAACCAGTCCTCTGAAGAGAAGGATA : 70
FWD : ATGCTGTGCCCATGGCAGTTTGCATTCAAACCTCATGCTGTTAAGAACCAGTCCTCTGAAGAGAAGGATA : 70

iNOS : TCAATAATAACGTGGAGAAAGATGTGAAGGTCCACAGCTTTGTGAAAGATGATGCCAAATTACACAGTCT : 140
FWD : TCAATAATAACGTGGAGAAAGATGTGAAGGTCCACAGCTTTGTGAAAGATGATGCCAAATTACACAGTCT : 140

iNOS : AAGCAAGAAGCAGATGAAGATGTCACCTATAATCACTTCAGCAGAGAAACACCCACAAAATGGTATCAAA : 210
FWD : AAGCAAGAAGCAGATGAAGATGTCACCTATAATCACTTCAGCAGAGAAACACCCACAAAATGGTATCAAA : 210

iNOS : GCTTCTAACCAAATATCAAGATGTCCTAGACATGTAAAAGTAAGGAACATGGAAAATGGATCCAGCCTTC : 280
FWD : GCTTCTAACCAAATATCAAGATGTCCTAGACATGTAAAAGTAAGGAACATGGAAAATGGATCCAGCCTTC : 280

iNOS : TAGACACATTACACCTGACAGCAAAGGAGGTTATCAATTGTCGGACCAGAGCTTGCCAAGGAGCACTCAT : 350
FWD : TAGACACATTACACCTGACAGCAAAGGAGGTTATCAATTGTCGGACCAGAGCTTGCCAAGGAGCACTCAT : 350

iNOS : GACCCCAAAGGGCCTGGTGAGAAGCACTAGAGATGGGCCAGTTCCTCCAGCAGAGCTTCTACCTCAAGCA : 420
FWD : GACCCCAAAGGGCCTGGTGAGAAGCACTAGAGATGGGCCAGTTCCTCCAGCAGAGCTTCTACCTCAAGCA : 420

iNOS : GTAGACTTTGTCAAGCAGTACTACAGTTCATTCAAAGAATTAAAAATAGAAGAGCACCTGGCCCGACTAG : 490
FWD : GTAGACTTTGTCTAGCAGTACTACAGTTCATTCTAAGAATTAAAAATAG..................... : 469

iNOS : AAACAGTGACCAAAGAGATAGAAACAACAGGAACCTACCATCTGACAAAGGATGAACTGATCTTTGCTGC : 560
FWD : ...................................................................... : -

iNOS : GCTCGTGATTGTAAAACAGCCAAGGAAATGTTTGAATATATCTGTCGCCATATTCAGTATGCCACAAACA : 700
FWD1 : ............................TGTTTGA.TATATCTGTCGC.ATATTCAGTATGCCACAAACA : 40

iNOS : ATGGAAATATAAGATCAGCCATCACCATTTTCCCTCAGAGGACTGATGGGAAACATGATTTCCGTGTTTG : 770
FWD1 : ATGGAAATATAAGATCAGCCATCACCATTTTCCCTCAGAGGACTGATGGGAAACATGATTTCCGTGTTTG : 110

iNOS : GAACAGCCAGCTCATCCGATATGCTGGATATCAAATGCCAGATGGGTCTGTCATAGGAGACCCTGCAAGT : 840
FWD1 : GAACAGCCAGCTCATCCGATATGCTGGATATCAAATGCCAGATGGGTCTGTCATAGGAGACCCTGCAAGT : 180

iNOS : GTGGAGTTCACAAAGTTGTGCATTGAGCTTGGGTGGAAGCCGAAATATGGCCGCTTTGATGTAGTTCCAC : 910
FWD1 : GTGGAGTTCACAAAGTTGTGCATTGAGCTTGGGTGGAAGCCGAAATATGGCCGCTTTGATGTAGTTCCAC : 250

iNOS : TCATTCTCCAAGCAAACGGCCAAGATCCAGAAATATTTGAATACCCGCCAGAGATTATCCTTGAAGTGCC : 980
FWD1 : TCATTCTCCAAGCAAACGGCCAAGATCCAGAAATATTTGAATACCCGCCAGAGATTATCCTTGAAGTGCC : 320

iNOS : AATGGAGCATCCAAAATATGAGTGGTTTAAGGAGTTGGATCTGAAGTGGTATGCTCTGCCTGCTGTTGCC : 1050
FWD1 : AATGGAGCATCCAAAATATGAGTGGTTTAAGGAGTTGGATCTGAAGTGGTATGCTCTGCCTGCTGTTGCC : 390

iNOS : AACATGCTCCTTGAGGTGGGAGGTCTGGAATTTACTGCGTGTCCTTTCAACGGCTGGTACATGGGAACAG : 1120
FWD1 : AACATGCTCCTTGAGGTGGGAGGTCTGGAATTTACTGCGTGTCCTTTCAACGGCTGGTACATGGGAACAG : 460

iNOS : AGATTGGAGTGCGAGACTTCTGTGATGTACAGCGGTACAATATCCTGAAGGAGGTTGGAAGAAGAATGGG : 1190
FWD1 : AGATTGGAGTGCGAGACTTCTGTGATGTACAGCGGTACAATATCCTGAAGGAGGTTGGAAGAAGAATGGG : 530

iNOS : ACTGGAAAGCAACAAACTTGCATCATTGTGGAAGGACCGAGCTGTTGTAGAGATAAATGTGGCTGTGCTT : 1260
FWD1 : ACTGGAAAGCAACAAACTTGCATCATTGTGTAAGGACCGAGCTGTTGTATAGATAAATGTGGCTGTGCTT : 600

iNOS : CATAGCTTCCAGAAACAAAATGTTACTATAATGGATCATCACTCAGCTGCTGAGTCCTTCATGAAATACA : 1330
FWD1 : CATAGCTTCCAGAAACAAAATGTTACTATAATGGATCATCACTCAGCTGCTG.................. : 652


iNOS : CATAGCTTCCAGAAACAAAATGTTACTATAATGGATCATCACTCAGCTGCTGAGTCCTTCATGAAATACA : 1330
FWD2 : C.TAGCTTC...AGAACAAATGTTACTATAATGGATCATCACTCAGCTGGTGCGTCCATCATGAAATACA : 74


iNOS : TGCAGAATGAGTACCGTGTGCGAGGAGGCTGCCCAGCTGATTGGGTGTGGATTGTACCTCCTATGTCGGG : 1400
FWD2 : TGCAGAATGAGTACCGTGTGCGAGGAGGCTGCCCAGCTGATTGCTTGTGCATTGTACCTCCTATGTCGGG : 144

iNOS : GAGCATAACTCCCGTGTTCCACCAGGAGATGTTGAACTATGTCCTCACTCCCTTCTTTTACTACCAGGTG : 1470
FWD2 : GAGCATAACTCCCGTGTTCCACCAGGAGATGTTGAACTATGTCCTCACTCCCTTCTTTTACTACCAGGTG : 214

iNOS : GATGCATGGAAAACACACATCTGGCATGATGAGACCCGTAGGCCAAAGAAAAGAGAAATAAAGTTGAGCA : 1540
FWD2 : GATGCATGGAAAACACACATCTGGCATGATGAGACCCGTAGGCCAAAGAAAAGAGAAATAAAGTTGAGCA : 284

iNOS : TTTTGGCCAAGGCTGTACTCTTGGCGTCATTACTCCTGCAAAAAACAATGGCAGCCAGGCCCAAGGTGAC : 1610
FWD2 : TTTTGGCCAAGGCTGTACTCTTTGCGTCATTACTCCTGCAAAAAACAATGGCAGCCAGGCCCAAGGTGAC : 354

iNOS : TGTGATCTATGCAACCGAGACTGGGAAATCAGAAACATTAGCCAACAGTCTGTGCAGTTTGTTTAGCTGT : 1680
FWD2 : TGTGATCTATGCAACCGAGACTGGGAAATCAGAAACATTAGCCTACAGTCTGTGTAGTTTGTTTAGCTGT : 424

iNOS : GCCTTCAACACTAAGATTCTGTGCATGGATGAGTACAACATCAGTGACTTGGAAAAAGAAACACTTCTTT : 1750
FWD2 : GCCTTCAACACTAAGATTCTGTGCATGGATGATTACAACATCAGTGACTTGTATAAAGAAACACTTCTTT : 494

iNOS : TAGTGGTTACTAGCACTTTTGGAAATGGAGATTCTCCAAATAATGGAAAGACGTTGAAGAACTCCTTGCT : 1820
FWD2 : TAGTGGTTACTAGCACTTTTGGAAATTGAGATTCTCCAATTAATGGAAAGATT................. : 547

iNOS : CACCCTGAAATTACTGAGAAAAAATATTAGATATGCTGTGTTTGGTTTGGGGTCCACCATGTATCCTGAG : 1890
FWD3 : ...............................GCTGTTGTTTTGGTTTTGGGGTCC.CCATGTATCCTGAG : 38

iNOS : TTCTGTGCCTTTGCTCATGCCATTGACCAGAAACTGTCCCAACTGGGGGCTTTGCAGCTCACTCCAGTGG : 1960
FWD3 : TTCTGTGCCTTTGCTCATGCCATTGACCAGAAACTGTCCCAACTGGGGGCTTTGCAGCTCACTCCAGTGG : 108

iNOS : GTGAAGGAGATGAACTCAATGGTCAAGAAGAAGCCTTTCGCACATGGGCAGTCACTGCATTCAAGACTGC : 2030
FWD3 : GTGAAGGAGATGAACTCAATGGTCAAGAAGAAGCCTTTCGCACATGGGCAGTCACTGCATTCAAGACTGC : 178

iNOS : CTGTGATATTTTTGACATCCGTGGGAAAAACAGTATTCAGTTACCTGAGATATACACATCAGATGACAGC : 2100
FWD3 : CTGTGATATTTTTGACATCCGTGGGAAAAACAGTATTCAGTTACCTGAGATATACACATCAGATGACAGC : 248

iNOS : TGGAATCCTAAGAAACATAGGATAGTATATGACTCTCAAACAATGGACTTGACTAAAGCACTGTCAGACA : 2170
FWD3 : TGGAATCCTAAGAAACATAGGATAGTATATGACTCTCAAACAATGGACTTGACTAAAGCACTGTCAGACA : 318

iNOS : TTCATGGAAAGAACGTAATTCCCATGAAGCTGAAATTCAGACAGAATCTTCAGAGTTTGAAATCCAGTCG : 2240
FWD3 : TTCATGGAAAGAACGTAATTCCCATGAAGCTGAAATTCAGACAGAATCTTCAGAGTTTGAAATCCAGTCG : 388

iNOS : TGTTACCATTCTAGTTAAGCTTTCCTGTGAGACTAATCAGGAAGTGCACTACCTGCCTGGAGAACATATT : 2310
FWD3 : TGTTACCATTCTAGTTAAGCTTTCCTGTGAGACTAATCAGGAAGTGCACTACCTGCCTGGAGAACATATT : 458

iNOS : GGGATTTCCCCAGGCAACCAGCCAGAATTAGTCCACGGCCTCATTGCACGTGTTAAGGATGCCCCTCCAG : 2380
FWD3 : GGGATTTCCCCAGGCAACCAGCCAGAATTAGTCCACGGCCTCATTGCACGTGTTAAGGATGCCCCTCCTG : 528

iNOS : CTGATCAGACTATCAGACTTGAAACCTGTACTGAAGGTGGCTATTGGGCAAGCGAGAAAAAGATTCCGGC : 2450
FWD3 : CTGATCAGACTATCAGACTTGAAACCTGTACTGAAGGTGGCTATTGGGCAAGCGAGAAAAAGATTCCGGC : 598

iNOS : TTGCACACTCTCACAGGCCTTGACATATTTACTTGATATCACTACCCCACCCACCCAACAACTGCTAAAA : 2520
FWD3 : TTGCACACTCTCACAGGCCTTGACATATTTACTTG................................... : 633

iNOS : TTGCACACTCTCACAGGCCTTGACATATTTACTTGATATCACTACCCCACCCACCCAACAACTGCTAAAA : 2520
FWD4 : .....................GACATATTTACTTGATATCACTACCCCACCCACCCAACAACTGCTAAAA : 49

iNOS : AAACTCTCCCAGCTGGTAACAGCGGAAGGAGACAAACAGAGACTGGAAGTTTTATGTCATAGCACAGAAG : 2590
FWD4 : AA.CTCTCCCAGCTGGTAACAGCGGAAGGAGACAAACAGAGACTGGAAGTTTTATGTCATAGCACAGAAG : 118

iNOS : AATACAATAAATGGAAGTTTTACAACAGGCCAAACATCCTGGAGGTCCTGGAAGAGTTTCCTTCTGCTGA : 2660
FWD4 : AATACAATAAATGGAAGTTTTACAACAGCCCAAACATCCTGGAGGTCCCGGAAGAGTTTCCTTCTGCTGA : 188

iNOS : AGTCTCAACAGCTTTCTTACTGACTCAGCTGCCACTTCTGAAACCCAGGTACTACTCTGTCAGTTCCTCC : 2730
FWD4 : AGTCTCAACAGCTTTCTTACTGACTCAGCTGCCACTTCTGAAACCCAGGTACTACTCTGTCAGTTCCTCC : 258

iNOS : TGTGACATGACACCCAGAGAGATTCATCTGACCGTTGCAGTGGTAAACTACAGGACAAGAGATGGACAAG : 2800
FWD4 : TGTGACATGACACCCAGAGAGATTCATCTGACCGTTGCAGTGGTAAACTACAGGACAAGAGATGGACAAG : 328

iNOS : GGCCATTGCACCATGGAGTCTGCAGCACGTGGCTGAACAAAATAGCTCTCAATGAAACAGTCCCGTGCTT : 2870
FWD4 : GGCCATTGCACCATGGAGTCTGCAGCACGTGGCTGAACAAAATAGCTCTCAATGAAACAGTCCCGTGCTT : 398

iNOS : TGTGCGCAGCGCTGATGGATTCCGGCTCCCAAAGGAGCCAGCCAAGCCGTGCATTCTTATTGGCCCAGGA : 2940
FWD4 : TGTGCGCAGCGCTGATGGATTCCGGCTCCCAAAGGAGCCAGCCAAGCCGTGCATTCTTATTGGCCCAGGA : 468

iNOS : ACAGGAATTGCTCCATTCAGAAGTTTCTGGCAGCAGCGTCTCTATGACTTGGAAAAGAAAGGGATCAAAG : 3010
FWD4 : ACAGGAATTGCTCCATTCAGAAGTTTCTGGCAGCAGCGTCTCTATGACTTGGAAAAGAAAGGGATCAAAG : 538

iNOS : GTGGTGACATGATATTACTGTTTGGCTGCCGGCATCCAGATATGGATCATATCTACAAAGAAGAAGTTGA : 3080
FWD4 : GTGGTGATATGATATTACTGTTTGGCTGCCGGCATCCATATATGGATCATATCTACAAAGAAGAAGTTGA : 608

iNOS : AGAAATGAAGAGGAAAGGAGTTCTGAAAGAAGTTTTTACAGCTTACTCCAGGCAACCTGGGCAGCCTAAA : 3150
FWD4 : AGAAATGAAGAGGAAAGGAGTTCTGAAAGAAGTTTTTACAGCTTACTCCTG................... : 659

iNOS : AGAAATGAAGAGGAAAGGAGTTCTGAAAGAAGTTTTTACAGCTTACTCCAGGCAACCTGGGCAGCCTAAA : 3150
FWD5 : .................GAGTTCTGAAAGAAGTTTTTACAGCTTACTCCAGGCAACCTGGGCAGCCTAAA : 53

iNOS : GTCTATGTTCAAGATATTCTTCAAAATGAGTTGGAAACCAAAGTGTGTAATATCTTGCATAAAGAGGAAG : 3220
FWD5 : GTCTATGTTCAAGATATTCTTCAAAATGAGTTGGAAACCAAAGTGTGTAATATCTTGCATAAAGAGGAAG : 123

iNOS : GGCATCTGTATGTCTGTGGAGATGTACGCATGGCCAGGGATGTTGCTCAGACTTTGAAGAGGATGCTTGT : 3290
FWD5 : GGCATCTGTATGTCTGTGGAGATGTACGCATGGCCAGGGATGTTGCTCAGACTTTGAAGAGGATGCTTGT : 193

iNOS : AAAAAAACTGAACCACACAGAGCAGCAGGCAGAAGAGTATTTCTTCCAGCTAAAGAGCCAAAAGCGATAC : 3360
FWD5 : AAAAAAACTGAACCACACAGAGCAGCAGGCAGAAGAGTATTTCTTCCAGCTAAAGAGCCAAAAGCGATAC : 263

iNOS : CATGAAGACATATTTGGGGCTGTGTTCCCACATGAAGTCAAAAGAATATAA : 3411
FWD5 : CATGAAGACATATTTGGGGCTGTGTTCCCACATGAAGTCAAAAGAATATAA : 314

IRAK2
FWD : ...........TTGTGTGGTCTTAGCAGAGATACTGACGGGGATTAAGGCACTGGATGAAGGAAGGAGCC : 59
IRAK2 : GTATTTGGCTGTGGTGTGGTCTTAGCAGAGATACTGACGGGGATTAAGGCACTGGATGAAGGAAGGAGCC : 1330

FWD : CTATTTATCTGAAAGATATGATTGCTGATGAAATCCAAACAGCAAAAGAAACCTCACACTCCAAAGACAA : 129
IRAK2 : CTATTTATCTGAAAGATATGATTGCTGATGAAATCCAAACAGCAAAAGAGACCTCACACTCCAAAGACAA : 1400

FWD : GAATTTGGAAAAGCTAGCTGCCAAGGAAATATGCTGTAAATACCTGGACAAGAAAGCAGGACATTTGCTG : 199
IRAK2 : GAATTTGGAAAAGCTAGCTGCCAAGGAAATATGCTGTAAATACCTGGACAAGAAAGCAGGACATTTGCTG : 1470

FWD : GAAGAGGTTGCCATTGATTTTGCCTCAGCCACCTGCCTTTGTCTGAAAAAGAAGAATTCTAACATAACAG : 269
IRAK2 : GAAGAGGTTGCCATTGATTTTGCCTCAGCCACCTGCCTTTGTCTGAAAAAGAAGAATTCTAACATAACAG : 1540

FWD : AGGTACTGGAAATTATGGAAACAGCTGAAAATAAATTGAGAGAGCATTACATCTGTGGAGGAGGCAGCAC : 339
IRAK2 : AGGTACTGGAAATTATGGAAACAGCTGAAAATAAATTGAGAGAGCATTACATCTGTGGAGGAGGCAGCAC : 1610

FWD : TTCTGGGTTTTCCATGAATACTCCGGAAGAAACTGACGATGAGACAACCAACCTCAGTGTGGACGCTCCT : 409
IRAK2 : TTCTGGGTTTTCCATGAATACTCCGGAAGAAACTGACGATGAGACAACCAACCTCAGTGTGGACGCTCCT : 1680

FWD : TCTGCAGGGCAGAATAAAGACGATAGCACACAGCCAGTACTCCTGGCAAGTGGCGGTCAGTGCACACCTT : 479
IRAK2 : TCTGCAGGGCAGAATAAAGACGATAGCACACAGCCAGTACTCCTGGCAAGTGGCGGTCAGTGCACACCTT : 1750

FWD : CAGTAGGAGTTGTGCCACCTGACACGTACTGTGAACAGATGTCTCGGGTGCCTTGTGAATCAGATGAATC : 549
IRAK2 : CAGTAGGAGTTGTGCCACCTGACACGTACTGTGAACAGATGTCTCGGGTGCCTTGTGAATCAGATGAATC : 1820

FWD : AACGAGTTTCATCTGGAACCCTGAAGAAAAATCTACAGATGAGCTATTCAGCAACAACTATAACAATTCA : 619
IRAK2 : AACGAGTTTCATCTGGAACCCTGAAGAAAAATCTACAGATGAGCTATTCAGCAACAACTATAACAATTCA : 1890

FWD : GAAAATATTACGAGCTCTGACTGCAGGATGAAGGAGAGAACAAAAGCAAATGGACTCCGGGAAAGAAACG : 689
IRAK2 : GAAAATATTACGAGCTCTGACTGCAGGATGAAGGAGAGAACAAAAGCAAATGGACTCCGGGAAAGAAACG : 1960

FWD : TAGTATGCATCAGAAGTGATGACAACTTGGAAATGGCTGCTACTGCACACAGCACATTTCAACCAAAAAA : 759
IRAK2 : TAGTATGCATCAGAAGTGATGACAACTTGGAAATGGCTGCTACTGCACACAGCACATTTCAACCAAAAAA : 2030

FWD : TGCAGACCTTGACTCCGCCAGTTCATCCCAAGGTAAATGTAGATAAAATCACTAGTGAATTCGCGGCCGC : 829
IRAK2 : TGCAGACCTTGACTCCGCCAGTTCATCCCAAGGTAAATGTAGATAA........................ : 2076

IRF1
IRF1 : ATGCCCGTCTCAAGGATGCGCATGAGACCCTGGTTGGAAATGCAGATTAATTCCAATCAAATACCGGGGC : 70
REV : ---------------------------------------------------------------------- : -
FWD : ATGCCCGTCTCAAGGATGCGCATGAGACCCTGGTTGGAAATGCAGATTAATTCCAATCAAATACCGGGGC : 70

IRF1 : TGATCTGGATCAACAAGGATAAGATGATATTTCAGATCCCATGGAAACATGCAGCTAAGCATGGCTGGGA : 140
REV : ---------------------------------------------------------------------- : -
FWD : TGATCTGGATCAACAAGGATAAGATGATATTTCAGATCCCATGGAAACATGCAGCTAAGCATGGCTGGGA : 140

IRF1 : CATGGAGAAGGACGCCTGCCTCTTCCGGAGCTGGGCCATCCATACAGGAAGATATAAAGTAGGCGAGAAG : 210
REV : ---------------------------------------------------------------------- : -
FWD : CATGGAGAAGGACGCCTGCCTCTTCCGGAGCTGGGCCATCCATACAGGAAGATATAAAGTAGGCGAGAAG : 210

IRF1 : GACCCCGACCCAAAAACCTGGAAGGCTAACTTCCGCTGCGCCATGAATTCACTGCCTGACATTGAAGAAG : 280
REV : -------------AAACCTGGAAGGCGAACTTCCGCTGCGCCATGAATTCACTGCCCGACATTGAAGAAG : 57
FWD : GACCCCGACCCAAAAACCTGGAAGGCGAACTTCCGCTGCGCCATGAATTCACTGCCCGACATTGAAGAAG : 280

IRF1 : TGAAGGATAAAAGCATCAACAAAGGCTCCAGCGCTGTCCGAGTGTACAGGATGCTCCCACCTCTGACAAA : 350
REV : TGAAGGATAAAAGCATCAACAAAGGCTCCAGCGCTGTCCGAGTGTACAGGATGCTCCCACCTCTGACAAA : 127
FWD : TGAAGGATAAAAGCATCAACAAAGGCTCCAGCGCTGTCCGAGTGTACAGGATGCTCCCACCTCTGACAAA : 350

IRF1 : GGACCAGAAGAAGGAAAGGAAGTCAAAGTCTTCAAGGGAAGCAAGAAACAAGAGCAAGAGAAAGTTGTAT : 420
REV : GGACCAGAAGAAGGAAAGGAAGTCAAAGTCTTCAAGGGAAGCAAGAAACAAGAGCAAGAGAAAGTTGTAT : 197
FWD : GGACCAGAAGAAGGAAAGGAAGTCAAAGTCTTCAAGGGAAGCAAGAAACAAGAGCAAGAGAAAGTTGTAT : 420

IRF1 : GAGGATATGAGGATGGAGGAGTCAGCAGAACGACTAACCAGCACTCCTCTGCCCGATGACCACAGCAGCT : 490
REV : GAGGATATGAGGATGGAGGAGTCAGCAGAACGACTAACCAGCACTCCTCTGCCCGATGACCACAGCAGCT : 267
FWD : GAGGATATGAGGATGGAGGAGTCAGCAGAACGACTAACCAGCACTCCTCTGCCCGATGACCACAGCAGCT : 490

IRF1 : ACACCGCTCACGACTACACGGGGCAGGAAGTGGAGGTGGAGAACACGTCCATCACCTTAGACCTCTCGTC : 560
REV : ACACCGCTCACGACTACACGGGGCAGGAAGTGGAGGTGGAGAACACGTCCATCACCTTAGACCTCTCGTC : 337
FWD : ACACCGCTCACGACTACACGGGGCAGGAAGTGGAGGTGGAGAACACGTCCATCACCTTAGA--------- : 551

IRF1 : CTGCGAGGTGAGCGGCTCCCTGACCGACTGGAGGATGCCGATGGAAATCGCCATGGCTGACAGCACCAAC : 630
REV : CTGCGAGGTGAGCGGCTCCCTGACCGACTGGAGGATGCCGATGGAAATCGCCATGGCTGACAGCACCAAC : 407
FWD : ---------------------------------------------------------------------- : -

IRF1 : GACATCTACCAGCTGCAGGTGTCCCCTCTGGGGTCGTCCTCAGAAGATGAGGATGAAATGAAATCAAACA : 700
REV : GACATCTACCAGCTGCAGGTGTCCCCTCTGGGGTCGTCCTCAGAAGATGAGGATGAAATGAAATCAAACA : 477
FWD : ---------------------------------------------------------------------- : -

IRF1 : TTATAAAGCTGCTTGAACCCACCCAGGACTGGCACACAACCAGCGTTGAGGGGAAAGGCTTCTTCACCAA : 770
REV : TTATAAAGCTGCTCGAACCCACCCAGGACTGGCACACAACCAGCGTTGAGGGGAAAGGCTTCTTCACCAA : 547
FWD : ---------------------------------------------------------------------- : -

IRF1 : CGAGCCGGGCACGCAGACCATGTGCAGCACGTTTGGCTACAAGGAGCAGGACGGCGAGATCGACACGTCT : 840
REV : CGAGCCGGGCACGCAGACCATGTGCAGCACGTTTGGCTACAAGGAGCAGGACGGCGAGATCGACACGTCT : 617
FWD : ---------------------------------------------------------------------- : -

IRF1 : TCGGCAGAGTTGGAGTTCCGCATGATGGACCAGAAGAGCAGCCTGGACTTCTCCTGGCTGGACACCGTCA : 910
REV : TCAGCAGAGTTGGAGTTCCGCATGATGGACCAGAAGAGCAGCCTGGACTTCTCCTGGCTGGACACCGTCA : 687
FWD : ---------------------------------------------------------------------- : -

IRF1 : GACCCATGCAGGCCATCTCCTGCAGCTTGTAA : 942
REV : GACCCATGCAGGCCATCTCCTGCAGCTTGTAA : 719
FWD : -------------------------------- : -

IRF10
IRF10 : ATGGCGGAGCCGGGGTCTCCCATGCGGCTGAAGGAGTGGCTGATCGCGCAGATCGACAGCGGCCGCTACC : 70
FWD : ATGGCGGAGCCGGGGTCTCCCATGCGGCTGAAGGAGTGGCTGATCGCGCAGATCGACAGCGGCCGCTACC : 70

IRF10 : CGGGGCTGCGCTGGGAGAACCGCGAGCGGACGCTCTTCCGCATCCCCTGGAAGCACGCGGCCAAGCAGGA : 140
FWD : CGGGGCTGCGCTGGGAGAACCGCGAGCGGACGCTCTTCCGCATCCCCTGGAAGCACGCGGCCAAGCAGGA : 140

IRF10 : CTACCGGCAGCAGCAGGACGCCGCTCTCTTCAGGGCTTGGGCCGTCTACAAGGGCAAATACCACGAGGGC : 210
FWD : CTACCGGCAGCAGCAGGACGCCGCTCTCTTCAGGGCTTGGGCCGTCTACAAGGGCAAATACCACGAGGGC : 210

IRF10 : ACGGACAAGGCAGACCCATCCACCTGGAAAACGCGACTGCGCTGCGCCCTCAACAAAAGCACGGACTTCC : 280
FWD : ACGGACAAGGCAGACCCATCCACCTGGAAAACGCGACTGCGCTGCGCCCTCAACAAAAGCACGGACTTCC : 280

IRF10 : AGGAGGTGCCCGAGCGGAGCCAGCTGGACATCTCCGAGCCTTACAAGGTGTACCAGATCGTGTGCGACGG : 350
FWD : AGGAGGTGCCCGAGCGGAGCCAGCTGGACATCTCCGAGCCTTACAAGGTGTACCAGATCGTGTGCGACGG : 350

IRF10 : CACTCGGGATGCAGAGAAGGATGAGAAGGAGGGCAGAATGCAGCCCACAAGCAGTAAGGACCCACAGGGC : 420
FWD : CACTCGGGATGCAGAGAAGGATGAGAAGGAGGGCAGAATGCAGCCCACAAGCAGTAAGGACCCACAGGGC : 420

IRF10 : CATGTGGCCGAGGAGAGCCACCGAGGCACAGCGGGAACATGCCACATGCCCACGTTACCCCTGCTAACTG : 490
FWD : CATGTGGCCGAGGAGAGCCACCGAGGCACAGCAGGAACATGCCACATGCCCACGTTACCCCTGCTAACTG : 490

IRF10 : CCCATCCAGCAGAGCGAGGGTACCACGTGCGGGGAATCTTCTATGGCTGGAGCCCGACCCGCAGCCACCT : 560
FWD : CCCATCCAGCAGAGCGAGGGTACCACGTGCGGGGAATCTTCTATGGCTGGAGCCCGACCCGCAGCCACCT : 560

IRF10 : GCTCCCCAGGGCCCCATCCTTTCTCCCTGCTGAGGATGTCAATCACTCAGACTGCTGGC : 619
FWD : GCTCCCCAGGGCCCCATCCTTTCTCCCTGCTGAGGATGTCAATCACTCAGACTGCTGGC : 619

JUN
JUN : ATGGAGCCTACTTTCTACGAGGATGCCCTGAACGCCAGCTTCGCGCCGCCGGAGAGCGGCGGCTATGGAT : 70
FWD : ATGGAGCCTACTTTCTACGAGGATGCCCTGAACGCCAGCTTCGCGCCGCCGGAGAGCGGCGGCTATGGAT : 70

JUN : ATAATAACGCCAAGGTGCTGAAGCAGAGCATGACGCTGAACCTGTCCGACGCGGCCAGCAGCCTGAAGCC : 140
FWD : ATAATAACGCCAAGGTGCTGAAGCAGAGCATGACGCTGAACCTGTCCGACCCGGCCAGCAGCCTGAAGCC : 140

JUN : GCACCTGAGGAACAAGAACGCCGACATCCTCACCTCCCCCGACGTGGGGCTGCTGAAGCTGGCCTCCCCG : 210
FWD : GCACCTGAGGAACAAGAACGCCGACATCCTCACCTCCCCCGACGTGGGGCTGCTGAAGCTGGCCTCCCCG : 210

JUN : GAGCTGGAACGGCTCATCATCCAGTCCAGCAACGGGTTAATCACCACCACGCCGACCCCGACGCAGTTCC : 280
FWD : GAGCTGGAGCGGCTCATCATCCAGTCCAGCAACGGGTTAATCACCACCACGCCGACCCCGACGCAGTTCC : 280

JUN : TCTGCCCCAAGAACGTTACCGACGAGCAAGAGGGGTTCGCCGAAGGCTTCGTGAGAGCGCTGGCGGAACT : 350
FWD : TCTGCCCCAAGAACGTTACCGACGAGCAAGAGGGGTTCGCCGAAGGCTTCGTGAGAGCGCTGGCGGAACT : 350

JUN : GCACAACCAGAACACGCTGCCCAGCGTCACCTCAGCCGCACAACCTGTTAGCGGCGGCATGGCACCTGTG : 420
FWD : GCACAACCAGAACACGCTGCCTAGCGTCACCTCATCCGCACAACCTGATAGCGGCGGCATGGCACCTGTG : 420

JUN : TCCTCCATGGCCGGCGGCGGCAGCTTCAACACGAGTTTGCACAGCGAGCCCCCGGTGTATGCCAATCTCA : 490
FWD : TCCTCCATGGCCGGCGGCGGCAGCTTCAACACGAGTTTGCACAGCGAGCCCTCGGTGTATGCCAATCTCA : 490

JUN : GCAACTTCAACCCCAACGCGCTCAACTCTGCACCCAACTACAACGCCAACGGCATGGGCTACGCGCCGCA : 560
FWD : GCAACTTCAACCCCAACGTGCTCAACTCCGCACCCAACTACATCGTC....................... : 537

JUN : GCAACTTCAACCCCAACGCGCTCAACTCTGCACCCAACTACAACGCCAACGGCATGGGCTACGCGCCGCA : 560
REV : ...............ACGCGCTCAACTCCGCACCCAACTACAACGCCAACGGCATGGGCTACGCGCCGCA : 55

JUN : GCATCACATAAACCCCCAGATGCCCGTGCAGCATCCCAGGCTTCAGGCTCTGAAAGAAGAGCCTCAGACT : 630
REV : GCATCACATAAACCCCCAGATGCCCGTGCAGCATCCCAGGCTTCAGGCTCTGAAAGAAGAGCCTCAGACT : 125

JUN : GTACCTGAAATGCCGGGGGAAACCCCTCCCCTGTCCCCTATTGACATGGAGTCGCAGGAGAGAATCAAAG : 700
REV : GTACCTGAAATGCCGGGGGAAACCCCTCCCCTGTCCCCTATTGACATGGAGTCGCAGGAGAGAATCAAAG : 195

JUN : CCGAGAGAAAACGCATGAGAAACAGAATTGCGGCGTCCAAATGCCGGAAAAGGAAGTTGGAAAGGATTGC : 770
REV : CCGAGAGAAAACGCATGAGAAACAGAATTGCGGCGTCCAAATGCCGGAAAAGGAAGTTGGAAAGGATTGC : 265

JUN : CAGGTTGGAAGAAAAAGTGAAAACTTTGAAAGCCCAGAACTCAGAGCTGGCATCCACGGCCAACATGCTC : 840
REV : CAGGTTGGAAGAAAAAGTGAAAACTTTGAAAGCCCAGAACTCAGAGCTGGCATCCACGGCCAACATGCTC : 335

JUN : AGAGAACAGGTTGCACAGCTTAAGCAGAAGGTCATGAACCATGTCAACAGCGGGTGCCAGCTAATGCTAA : 910
REV : AGAGAACAGGTTGCACAGCTTAAGCAGAAGGTCATGAACCATGTCAACAGCGGGTGCCAGCTAATGCTAA : 405

JUN : CACAACAGTTGCAAACGTTTTGA : 933
REV : CACAACAGTTGCAAACGTTTTGA : 428

LYG2
LYG2 : GAACATAGCAAATGTTGAAACAACTGGTGCATCACAGAGAACTGCGAAGCCGGAAGGTCTGAGCTATGCA : 70
FWD : GAACATAGCAAATGTTGAAACAACTGGTGCATCACAGAGAACTGCGAAGCCGGAAGGTCTGAGCTATGCA : 70

LYG2 : GGAGTTGCGGCTTCAGAGAAGATTGCTGAAAGAGATTTGAAGAATATGGACAAATATAAAGAAACTATTA : 140
FWD : GGAGTTGCGGCTTCAGAGAAGATTGCTGAAAGAGATTTGAAGAATATGGACAAATATAAAGAAACTATTA : 140

LYG2 : CAAAAGTGGCCAACAGCAAGTGCATTCCACCATCTTTGGTTGCTGCTGTTATCTCTCGAGAGTCACACGC : 210
FWD : CAAAAGTGGCCAACAGCAAGTGCATTCCACCATCTTTGGTTGCTGCTGTTATCTCTCGAGAGTCACACGC : 210

LYG2 : TGGGACGGCACTGAAGGATGGCTGGGGTGACCACGGTAATGCATTTGGTTTAATGCAGGTTGACAAACGG : 280
FWD : TGGGACGGCACTGAAGGATGGCTGGGGTGACCACGGTAATGCATTTGGTTTAATGCAGGTTGACAAACGG : 280

LYG2 : TACCATAAACCTCATGGGGCATGGGACAGTGAAGAGCACATAAAACAAGGCACAGACATTTTGTGTCAGT : 350
FWD : TACCATAAACCTCATGGGGCATGGGACAGTGAAGAGCACATAAAACAAGGCACAGACATTTTGTGTCAGT : 350

LYG2 : CAATAACCGATATTCAGAAAAAATTCCCAACATGGAGTAAGGAACAGCAGCTCAAAGGTGGTATTTCAGC : 420
FWD : CAATAACCGACATTCAGAAAAAATTCCCAACATGGAGTAAGGAACAGCAGCTCAAAGGTGGTATTTCAGC : 420

LYG2 : CTATAATGCAGGAACAAGAAATGTCCGGACCTATGAAGGAATGGATGTTGGCACAACACACGACGACTAT : 490
FWD : CTATAATGCAGGAACGAGAAATGTCCGGACCTATGAAGGAATGGATGTTGGCACAACACACGACGACTAT : 490

LYG2 : GCCAACGATGTGGTTGCAAGAGCCAAGTTCTTTCAGAGAAATGGATACTGA : 541
FWD : GCCAACGATGTGGTTGCAAGAGCCAAGTTCTTTCAGAGAAATGGATACTGA : 541

LYZ
REV : ATG.GGTCTTTGCTAATCTTGGTGCTTTGCTTCCTGCCCCTGGCTGCTCTGGGGAAAGTCTTTGGACGAT : 69
LYZ : ATGAGGTCTTTGCTAATCTTGGTGCTTTGCTTCCTGCCCCTGGCTGCTCTGGGGAAAGTCTTTGGACGAT : 70
FWD : ATG.GGTCTTTGCTAATCTTGGTGCTTTGCTTCCTGCCCCTGGCTGCTCTGGGGAAAGTCTTTGGACGAT : 69

REV : GTGAGCTGGCAGCGGCTATGAAGCGTCACGGACTTGATAACTATCGGGGATACAGCCTGGGAAACTGGGT : 139
LYZ : GTGAGCTGGCAGCGGCTATGAAGCGTCACGGACTTGATAACTATCGGGGATACAGCCTGGGAAACTGGGT : 140
FWD : GTGAGCTGGCAGCGGCTATGAAGCGTCACGGACTTGATAACTATCGGGGATACAGCCTGGGAAACTGGGT : 139

REV : GTGTGCCGCAAAATTCGAGAGTAACTTCAACACCCAGGCTACAAACCGTAACACCGATGGGAGTACCGAC : 209
LYZ : GTGTGTTGCAAAATTCGAGAGTAACTTCAACACCCAGGCTACAAACCGTAACACCGATGGGAGTACCGAC : 210
FWD : GTGTGCCGCAAAATTCGAGAGTAACTTCAACACCCAGGCTACAAACCGTAACACCGATGGGAGTACCGAC : 209

REV : TACGGAATCCTACAGATCAACAGCCGCTGGTGGTGCAACGATGGCAGGACCCCAGGCTCCAGGAACCTGT : 279
LYZ : TACGGAATCCTACAGATCAACAGCCGCTGGTGGTGCAACGATGGCAGGACCCCAGGCTCCAGGAACCTGT : 280
FWD : TACGGAATCCTACAGATCAACAGCCGCTGGTGGTGCAACGATGGCAGGACCCCAGGCTCCAGGAACCTGT : 279

REV : GCAACATCCCGTGCTCAGCCCTGCTGAGCTCAGACATAACAGCGAGCGTGAACTGCGCGAAGAAGATCGT : 349
LYZ : GCAACATCCCGTGCTCAGCCCTGCTGAGCTCAGACATAACAGCGAGCGTGAACTGCGCGAAGAAGATCGT : 350
FWD : GCAACATCCCGTGCTCAGCCCTGCTGAGCTCAGACATAACAGCGAGCGTGAACTGCGCGAAGAAGATCGT : 349

REV : CAGCGATGGAAACGGCATGAACGCGTGGGTCGCCTGGCGCAACCGCTGCAAGGGCACCGACGTCCAGGCG : 419
LYZ : CAGCGATGGAAACGGCATGAGCGCGTGGGTCGCCTGGCGCAACCGCTGCAAGGGTACCGACGTCCAGGCG : 420
FWD : CAGCGATGGAAACGGCATGAACGCGTGGGTCGCCTGGCGCAACCGCTGCAAGGGCACCGACGTCCAGGCG : 419

REV : TGGATCAGAGGCTGCCGGCTGTGA : 443
LYZ : TGGATCAGAGGCTGCCGGCTGTGA : 444
FWD : TGGATCAGAGGCTGCCGGCTGTGA : 443

MADPRT
MADPRT : ATGGAGCACGCCATTCTGGGCTTGGTGCTGCTGCTCAGCACCAGGACTGATGCATCGGCTGCCAGGAGCA : 70
FWD : ATGGAGCACGCCATTCTGGGCTTGGTGCTGCTGCTCAGCACCAGGACTGATGCATCGGCTGCCAGGAGCA : 70

MADPRT : AGAAAGGCCCCATAAAGGAGGTGGCGATGGACATGGCCCCCCACTCCTTTGATGACCAGTACCAGGGCTG : 140
FWD : AGAAAGGCCCCATAAAGGAGGTGGCGATGGACATGGCCCCCCACTCCTTTGATGACCAGTACCAGGGCTG : 140

MADPRT : CATCCACTTGATGGAGGCTGAGCTGGAGGAGCTGAACCGCACCGAGTTCGCCAACGAAACCTTTGCTGAG : 210
FWD : CATCGACTTGATGGAGGCTGAGCTGGAGGAGCTGAACCGCACCGAGTTCGCCAACGAAACCTTTGCTGAG : 210

MADPRT : GAATGGAGAAATGCCACAGAGAAGTGGCAGCGCCGATGGGGTCGGGTCTCCAGCCCAATGGTGCTGCGAC : 280
FWD : GGCTGGAGGAGCGCCACAAAGGAGTGGCAGCGCCGATGGGGTCGGGTCTCCAGCCCAATGGTGCTGCGAC : 280

MADPRT : AGGATCAAGCCATAGCCATGCTGGCGTATACAATGGAGGGAGAACTGTACCGTGTGTTCAACAACGCCAC : 350
FWD : AGGATCAAGCCATAGCCATGCTGGCGTATACAATGGAGGGAGAACTGTACCGTGTGTTCAACAACGCCAC : 350

MADPRT : GCTCACGGCTGGGCGCTCCCGGCAGCACTACCTGAGCTCCTACCCCTTCAAGGCACTGCATTTCCTACTG : 420
FWD : GCTCACGGCTGGGCGCTCCCGGCAGCACTACCTGAGCTCCTACCCCTTCAAGACACTGCATTTCCTACTG : 420

MADPRT : AGCAGAGCCCTGCACACCCTGCGGGAATCCCAAACCCAGCGATGCCACAATGTCTTCCGTG : 481
FWD : AGCAGAGCCCTGCAGACCCTGCGGGAATCCCAAACCCAGCGATGCCACAATGTCTTCCGTG : 481

MAFA
MAFA : ATGGCCTCGGAGTTGGCCATGACCGCGGAGCTGCCCACCAGCCCCCTCGCCATCGAATACGTCAACGATT : 70
FWD : ...................................................................... : -
REV : ATGGCCTCGGAGTTGGCCATGACCGCGGAGCTGCCCACCAGCCCCCTCGCCATCGAATACGTCAACGATT : 70

MAFA : TTGACCTGATGAAGTTTGAGGTGAAGAAAGAACCGGCGGAGGCCGAGAGGCTGTGCCACCGCCTGCCTGC : 140
FWD : ...................................................................... : -
REV : TTGACCTGATGAAGTTTGAGGTGAAGAAAGAACCGGCGGAGGCCGAGAGGCTGTGCCACCGCCTGCCTGC : 140

MAFA : CGGTTCTCTATCTTCCACCCCTCTCAGCACCCCGTGCTCCTCCGTGCCTTCCTCGCCCAGTTTCTGCGCT : 210
FWD : ...................................................................... : -
REV : CGGTTCTCTATCTTCCACCCCTCTCAGCACCCCGTGCTCCTCCGTGCCTTCCTCGCCCAGTTTCTGCGCT : 210

MAFA : CCCAGCCCCGGTGGGCAACCGAGTGCTGGTCCCACCGCCGCTCCCCTGGGCTCCAAACCGCAGCTGGAGG : 280
FWD : ...................................................................... : -
REV : CCCAGCCCCGGTGGGCAACCGAGTGCTGGTCCCACCGCCGCTCCCCTGGGCTCCAAACCGCAGCTGGAGG : 280

MAFA : AGCTGTATTGGATGTCGGGCTACCAACACCACCTCAACCCCGAAGCGCTCAACTTGACCCCGGAGGATGC : 350
FWD : ...................................................................... : -
REV : AGCTGTATTGGATGTCGGGCTACCAACACCACCTCAACCCCGAAGCGCTCAACTTGACCCCGGAGGATGC : 350

MAFA : TGTGGAAGCGTTGATCGGCGCTCCGCACCACCATCATCACCACCATCAGAGCTATGAGTCGTTCCGGCCT : 420
FWD : ...................................................................... : -
REV : TGTGGAAGCGTTGATCGGCGCTCCGCACCACCATCATCACCACCATCAGAGCTATGAGTCGTTCCGGCCT : 420

MAFA : CAGCCCTTTGGGGGCGAGGAGCTGCCCCCGGCCGCCCACCACCACAACGCCCACCACCACCATCACCACC : 490
FWD : ...............................................CGCCCACCACCACCATCACCACC : 23
REV : CAGCCCTTTGGGGGCGAGGAGCTGCCCCCGGCCGCCCACCACCACAACGCCCACCACCACCATCACCACC : 490

MAFA : ACCACCTGCGCTTGGAGGAGAGGTTTTCCGACGACCAGTTGGTGAGTATGTCGGTGCGGGAGCTCAACCG : 560
FWD : ACCACCTGCGCTTGGAGGAGAGGTTTTCCGACGACCAGTTGGTGAGTATGTCGGTGCGGGAGCTCAACCG : 93
REV : ACCACCTGCGCTTGGAGGAGAGGTTTTCCGACGACCAGTTGGTGAGTATGTCGGTGCGGGAGCTCAACCG : 560

MAFA : GCAGCTGCGGGGATTCAGCAAGGAGGAGGTGATTCGCCTCAAGCAGAACAGGAGGACCTTGAAGAACCGC : 630
FWD : GCAGCTGCGGGGATTCAGCAAGGAGGAGGTGATTCGCCTCAAGCAGAAGAGGAGGACCTTGAAGAACCGC : 163
REV : GCAGCTGCGGGGATTCAGCAAGGAGGAGGTGATTCGCCTCAAGCAGAAGAGGAGGACCTTGAAGAACCGC : 630

MAFA : GGCTACGCGCAGTCCTGTCGCTACAAGCGGGTCCAGCAGAGGCACATCCTGGAGAACGAGAAGTGTCAGC : 700
FWD : GGCTACGCGCAGTCCTGTCGCTACAAGCGGGTCCAGCAGAGGCACATCCTGGAGAACGAGAAGTGTCAGC : 233
REV : GGCTACGCGCAGTCCTGTCGCTACAAGCGGGTCCAGCAGAGGCACATCCTGGAGAACGAGAAGTGTCAGC : 700

MAFA : TCCAGAGCCAAGTGGAGCAACTCAAGCAAGAAGTTTCCCGTTTGGCCAAGGAGCGGGATCTGTACAAAGA : 770
FWD : TCCAGAGCCAAGTGGAGCAACTCAAGCAAGAAGTTTCCCGTTTGGCCAAGGAGCGGGATCTGTACAAAGA : 303
REV : TCCAGAGCCAAGTGGAGCA.CTCAAGCAAGAAGTTTCCCGTTTGGCCAAGGAGCGGGATCT......... : 760

MAFA : GAAGTACGAGAAGTTGGCGGCGCGGGGCTTCCCCCGGGAGCCATCCCCTCCCGCAGCCCCAAAAACTACT : 840
FWD : GAAGTACGAGAAGTTGGCGGCGCGGGGCTTCCCCCGGGAGCCATCCCCTCCCGCAGCCCCAAAAACTACT : 373
REV : ...................................................................... : -

MAFA : GCCGCTGACTTCTTCATGTGA : 861
FWD : GCCGCTGACTTCTTCATGTGA : 394
REV : ..................... : -

MAFF
REV : ATGGCTGCGGATGGGCTGTCCAGCAAAGCATTAAAGGTGAAGCGGGAGCTGGGAGAGAACACGCCGCTGC : 70
MAFF : ATGGCTGCGGATGGGCTGTCCAGCAAAGCATTAAAGGTGAAGCGGGAGCTGGGAGAGAACACGCCGCTGC : 70
FWD : ATGGCTGCGGATGGGCTGTCCAGCAAAGCATTAAAGGTGAAGCGGGAGCTGGGAGAGAACACGCCGCTGC : 70

REV : TGTCGGATGAGGAGCTGATGGGGCTGTCGGTGCGGGAGCTCAACCACCACCTGCGGGGCCTTTCCAAGGA : 140
MAFF : TGTCGGATGAGGAGCTGATGGGGCTGTCGGTGCGGGAGCTCAACCACCACCTGCGGGGCCTTTCCAAGGA : 140
FWD : TGTCGGATGAGGAGCTGATGGGGCTGTCGGTGCGGGAGCTCAACCACCACCTGCGGGGCCTTTCCAAGGA : 140

REV : GGAGGTGGCGAGGCTGAAGCAGCGACGACGGACGCTGAAGAACCGGGGCTACGCTGCCAGCTGTCGAGTG : 210
MAFF : GGAGGTGGCGAGGCTGAAGCAGCGACGACGGACGCTGAAGAATCGGGGCTACGCTGCCAGCTGTCGAGTG : 210
FWD : GGAGGTGGCGAGGCTGAAGCAGCGACGACGGACGCTGAAGAACCGGGGCTACGCTGCCAGCTGTCGAGTG : 210

REV : AAGCGTGTCTGCCAGAAGGAAGAGCTGCAGAAGCGGAAGATGGAGCTGGAATGGGAGGTGGACAAGCTGG : 280
MAFF : AAGCGTGTCTGCCAGAAGGAAGAGCTGCAGAAGCAGAAGATGGAGCTGGAATGGGAGGTGGACAAGCTGG : 280
FWD : AAGCGTGTCTGCCAGAAGGAAGAGCTGCAGAAGCGGAAGATGGAGCTGGAATGGGAGGTGGACAAGCTGG : 280

REV : CCCGGGAGAATGCTGCCATGCGCCTGGAGCTTGACACTCTCCGTGGCAAGTACGAGGCCCTGCAGGGCTT : 350
MAFF : CCCGGGAGAATGCTGCCATGCGCCTGGAGCTTGACACTCTCCGTGGCAAGTACGAGGCCCTGCAGGGCTT : 350
FWD : CCCGGGAGAATGCTGCCATGCGCCTGGAGCTTGACACTCTCCGTGGCAAGTACGAGGCCCTGCAGGGCTT : 350

REV : TGCCCGCACTGTGGCTGCCCACGGGCCACCTGCCAAAGTGGCCACTGCCAGCGTCATCACCATCGTCAAG : 420
MAFF : TGCCCGCACTGTGGCTGCCCACGGGCCACCTGCCAAAGTGGCCACTGCCAGCGTCATCACCATCGTCAAG : 420
FWD : TGCCCGCACTGTGGCTGCCCACGGGCCACCTGCCAAAGTGGCCACTGCCAGCGTCATCACCATCGTCAAG : 420

REV : TCTGGTGCCAACCAGGCCGCCTACTCCTAG : 450
MAFF : TCTGGTGCCAACCAGGCCGCCTACTCCTAG : 450
FWD : TCTGGTGCCAACCAGGCCGCCTACTCCTAG : 450

MYD88
MYD88 : ATGGCTACGGTACCCGTGGGTGCGGGCTCCGCTCCCGGCCCCGAGCCGGCGGATCTGCACTCCGTGCCTA : 70
REV : ...................................................................... : -

MYD88 : TGGTGGCCCTCAACTACGGCGTGCGGCGCCGCCTCGGCCTTTACCTCAACCCGCGGGCGGCGACCGCCGC : 140
REV : ...................................................................... : -

MYD88 : CGACTGGACGGCGCTGGCGGAGAAGTTGGGCCACGACTACCTGGAGATCCGGCGGCTGGAGGCGCTGCCC : 210
REV : ......................................ACCTGCAG.....GCGGCCGCGAAT.TCACTA : 26

MYD88 : GACCCCACGGCGGCGCTGCTGGAAGAGTGGCAGAGCCGCTGTCCCGGCGGAGCCACCGTGGGTCAACTGC : 280
REV : GTGATTATGGCTACGGTACCCGT.GGGTGCGGGCTCCGCT..CCCGGCCCCGAGCCGGCGGATCTGC... : 90

MYD88 : TGGAGCTGCTCCGGCAGCTGGGCCGCCACGATGTCCTGCTGGAGCTGGGCGGCAGCGTGGAGGAGGACTG : 350
REV : ..ACGCCGTGCCTATGG.TGGCCCTCAACTACGGCGTGC...........GGCAGCGTGGAGGAGGACTG : 146

MYD88 : CAAGAAGTACTTACGAAGGAAGCAGCAGGAGGCCGAGCAGCCGCTGCAGGTCCCGGCGGTAGACAGCAGC : 420
REV : CAAGAAGTACTTACGAAGGAAGCAGCAGGAGGCCGAGCAGCCGCTGCAGGTCCCGGCGGTAGACAGCAGC : 216

MYD88 : GTGCCAAAGACTTCAGAGCTGATGGGCATCACCACGAGGGATGATCCGTATGGGCATGGAACAGAGATGT : 490
REV : GTGCCAAAGACTTCAGAGCTGATGGGCATCACCACGAGGGATGATCCGTATGGGCATGGAACAGAGATGT : 286

MYD88 : TTGATGCCTTCATCTGCTACTGTCAGAAAGACCTTCAGTTTGTCCAGGAGATGATCAGAGAGTTGGAGCA : 560
REV : TTGATGCCTTCATCTGCTACTGTCAGAAAGACCTTCAGTTTGTCCAGGAGATGATCAGAGAGTTGGAGCA : 356

MYD88 : AACGGAGTTCAAACTGAAGCTGTGTGTGTTTGATCGGGATGTCTTGCCAGGAACGTGTGTGTGGTCCATT : 630
REV : AACGGAGTTCAAACTGAAGCTGTGTGTGTTTGATCGGGATGTCTTGCCAGGAACGTGTGTGTGGTCCATT : 426

MYD88 : AGTGGAGAACTCATAGAAAGAAGGTGTCGGAGGATGGTGGTCGTCATTTCAGATGATTACCTGGAAAGTG : 700
REV : AGTGGAGAACTCATAGAAAGAAGGTGTCGGAGGATGGTGGTCGTCATTTCAGATGATTACCTGGAAAGTG : 496

MYD88 : ATGAATGTGATTTTCAGACCAAATTTGCTCTTAGTCTTTCCCCAGGGGCTCGTCTCAAGCGGCTGATTCC : 770
REV : ATGAATGTGATTTTCAGACCAAATTTGCTCTTAGTCTTTCCCCAGGGGCTCGTCTCAAGCGGCTGATTCC : 566

MYD88 : GGTCAAGTGCAAGACCATGAAGAACGAGTTTCCAAGTATCTTGCGGTTCATTACAATTTGTGATTACACC : 840
FWD : GGTCAAGTGCAAGACCATGAAGAACGAGTTTCCAAGTATCTTGCGGTTCATTACAATTTGTGATTACACC : 139

MYD88 : AATCCTTGCACCAAAAAAATGGTTCTGGACAAGACTGGCAAAATCTCTCTTGCTGCCGTGATGCAAAGTT : 910
FWD : AATCCTTGCACCAAAAAA.TGGTTCTGGACAAGACTGGCAAAATCTCTCTTGCTGCCGTGATGCAAAGTT : 208

MYD88 : TTGAGAGCTTTTTCTCCATCTGTCCTGGCTGTGCCTTCGGACCACGGTGTTCCTACAGCATCTCACTGCA : 980
FWD : TTGAGAGCCTTTTCTCCATCTGTCCTGGCTGTGCCTTCGGACCACGGTGTTCCTACAGCATCTCACTGCA : 278

MYD88 : CAACTTGGATCCTCGCAGTCAGCATTTGGAGCCCGCAGCTCGGAGCAAAGAATTTTCTGTAGCACTTTTT : 1050
FWD : CAACTTGGATCCTTGCAGTCAGCATTTGGAGCCCGCAGCTCGGAGCAAAGAATTTTCTGTAGCACTTTTT : 348

MYD88 : CATAACTTGGAAGGGGAAATAGAGCAACTGTGTGGGTGTTCCTACTTAATTCAGCGAGCAATAGCATCCA : 1120
FWD : CATAACTTGGAAGGGGAAATAGAGCAACTGTGTGGGTGTTCCTACTTAATTCAGTGAGCAATAGCATCCA : 418

MYD88 : GCACTTGGTGA : 1131
FWD : GCACTTGGTGA : 429

NDNF
NDNF : ATGCAGAAGCTGCCTACCAGAGATGAGGAGCTATTCCAGATGCAGATCCGGGACAAAGCGTTTTTTCATG : 70
FWD : ATGCAGAAGCTGCCTACCAGAGATGAGGAGCTATTCCAGATGCAGATCCGGGACAAAGCGTTTTTTCATG : 70

NDNF : ATTCATCAGTCATCCCAGATGGAGCCGAAATTAGCAGCTACCTCTTCCGAGACACACCTAAAAGGTACTT : 140
FWD : ATTCATCAGTCATCCCAGATGGAGCCGAAATTAGCAGCTACCTCTTCCGAGACACACCTAAAAGGTACTT : 140

NDNF : TTTTGTGGTGGAAGAGGACAACACACCCCTAGCAGTAACAGTGACACCCTGTGATGCTCCCCTGGAGTGG : 210
FWD : TTTTGTGGTGGAAGAGGACAACACACCCCTAGCAGTAACAGTGACACCCTGTGATGCTCCCCTGGAGTGG : 210

NDNF : AAACTGAGTGTACAAGAGCTCCCAGAGGAAGCCAGTGGAGAAGGTTCAGGAGAACCAGAACCTCTTGAGC : 280
FWD : AAACTGAGTGTACAAGAGCTCCCAGAGGAAGCCAGTGGAGAAGGTTCAGGAGAACCAGAACCTCTTGAGC : 280

NDNF : AACAGAAACAGCAGATTACTAATGAAGAAGGCACAGAGCTGTTCTCTTACAAAGGCAACGACGTTGAGTA : 350
FWD : AACAGAAACAGCAGATTACTAATGAAGAAGGCACAGAGCTGTTCTCTTACAAAGGCAACGACGTTGAGTA : 350

NDNF : TTTTGTGTCCTCTAGTTCCCCTTCTGGTTTGTACCAACTAGATTTGCTGTCAACAGAGAAGGATACACAT : 420
FWD : TTTTGTGTCCTCTAGTTCCCCTTCTGGTTTGTACCAACTAGATTTGCTGTCAACAGAGAAGGATACACAT : 420

NDNF : TTTAAAGTGTATGCAACTACTACTCCAGAATCTGACCAACCTTACCCTGAATTACCTTATGATCCAAGAA : 490
FWD : TTTAAAGTGTATGCAACTACTACTCCAGAATCTGACCAACCTTACCCTGAATTACCTTATGATCCAAGAA : 490

NDNF : TTGATGTTACTTCTCTGGGACGTACGACAGTAACGCTGGCATGGAAGCCAAGTCCCACGGCCTCATTGCT : 560
FWD : TTGATGTTACTTCTCTGGGACGTACGACAGTAACGCTGGCATGGAAGCCAAGTCCCACGGCCTCATTGCT : 560

NDNF : GAAACAGCCAATTCAGTATTGCATAGTCATCAATAAAGAGCACAATTTCAAAAGCCTCTGTGCTGTTGAA : 630
FWD : GAAACAGCCAATTCAGTATTGCATAGTCATCAATAAAGAGCACAATTTCAAAAGCCTCTGTGCTGTTGAA : 630

NDNF : GCCAAGCTGAGTTCTGATGATGCCTTCATGATGGCTCCAAAACCAGGTCTGGATTTCAGTCCATTTGACT : 700
FWD : GCCAAGCTGAGTTCTGATGATGCCTTCATGATGGCTCCAAAACCAGGTCTGGATTTCAGTCCGTTTGACT : 700

NDNF : TTGCCCATTTTGGCTTCCCCTCAGACAGCAATGCTGGCAAAGAACGTGGTTTCCTAAAATCACCATCAAA : 770
FWD : TTGCCCATTTTGGCTTCCCCTCAGACAGCAATGCTGGCAAAGAACGTGGTTTCCTAAAATCACCATCAAA : 770

NDNF : ATTTGGGCGTCAAACATCCTCAAAGCCAAGAGTTGACCTGCATAAAGTTTGTATTGGGAACAAGAACATC : 840
FWD : ATTTGGGCGTCAAACATCCTCAAAGCCAAGAGTTGACCTGCATAAAGTTTGTATTGGGAACAAGAACA.. : 838

NDNF : ATTTGGGCGTCAAACATCCTCAAAGCCAAGAGTTGACCTGCATAAAGTTTGTATTGGGAACAAGAACATC : 840
REV : ..................................................................CATC : 4

NDNF : TTCACGGTGTCTGATCTGAAGCCCGACACACAGTACTACTTTGACATGTTTGCAGTAAACACTAACACAA : 910
REV : TTCCCCGTGTCTGATCTGAAGCCCGACACACAGTACTACTTTGACATGTTTGCAGTAAACACTAACACAA : 74

NDNF : ACCTGAGCACCGCATATGTTGGCACCTTTGCCAGAACGAAGGAGGAGGCCAAGCAGAAAACAGTTGAGCT : 980
REV : ACCTGAGCACCGCATATGTTGGCACCTTTGCCAGAACGAAGGAGGAGGCCAAGCAGAAAACAGTTGAGCT : 144

NDNF : GAAGGATGGCAAAGTTACAGATGTGTTCATCAAGAGGAAGGGAGCCAAATTTCTGCGGTTTGCTCCTGTT : 1050
REV : GAAGGATGGCAAAGTTACAGATGTGTTCATCAAGAGGAAGGGAGCCAAATTTCTGCGGTTTGCTCCTGTT : 214

NDNF : TCATCTCACCAGAAGGTCACTTTTTCTGTTCATTCATGCCTAGATGCTGTTCAGATCCAAGTTAGAAGAG : 1120
REV : TCATCTCACCAGAAGGTCACCTTTTCTGTTCATTCATGCCTAGATGCTGTTCAGATCCAAGTTAGAAGAG : 284

NDNF : ATGGCAAACTACTCTTGTCTCAAAACGTGGAAGGGGTACGGCAGTTCCAGCTGAGAGGGAAAGCAAAAGC : 1190
REV : ATGGCAAACTACTCTTGTCTCAAAACGTGGAAGGGGTACGGCAGTTCCAGCTGAGAGGGAAAGCAAAAGC : 354

NDNF : TAAGTATCTAATTAGGCTGAAAGGAAACAAAAAAGGTGCTTCTATGCTGAAGATTCTGGCAACGACAAGG : 1260
REV : TAAGTATCTAATTAGGCTGAAAGGAAACAAAAAAGGTGCTTCTATGCTGAAGATTCTGGCAACGACAAGG : 424

NDNF : CCTAACAAGCAGTTATTTCCTTCTCTTCCTGAAGATACAAGAATCAAAGCCTTTGACAAACTCCGCACGT : 1330
REV : CCTAACAAGCAGTTATTTCCTTCTCTTCCTGAAGATACAAGAATCAAAGCCTTTGACAAACTCCGCACGT : 494

NDNF : GTTCCTCAGTCACAGTGGCATGGCTTGGCACACAGGAGAGAAACAAATTCTGCATCTACAAAAAGGAGGT : 1400
REV : GTTCCTCAGTCACAGTGGCATGGCTTGGCACACAGGAGAGAAACAAATTCTGCATCTACAAAAAGGAGGT : 564

NDNF : GGATGACAACTACAACGAGGAGCAGAAGAAAAGAGAACAGAACCAGTGCTTGGGTCCAGATACAAGGAAG : 1470
REV : GGATGACAACTACAACGAGGAGCAGAAGAAAAGAGAACAGAACCAGTGCTTGGGTCCAGATACAAGGAAG : 634

NDNF : AAGTCGGAAAAGGTTCTCTGTAAATACTTTCACAGCCAGAATATACAGAAAGCAGTTACCACAGAGACAA : 1540
REV : AAGTCGGAAAAGGTTCTCTGTAAATACTTTCACAGCCAGAATATACAGAAAGCAGTTACCACAGAGACAA : 704

NDNF : TCCGGGGCCTGCAGTCTGGCAAGTCCTACTTGCTGGACGTGTATGTCATTGGGCATGGGGGGCACTCTGT : 1610
REV : TCCGGGGCCTGCAGTCTGGCAAGTCCTACTTGCTGGACGTGTATGTCATTGGGCATGGGGGGCACTCTGT : 774

NDNF : CAAATACCAGAGCAAATTGGTGAAAACGAGGAAGTTCTGTTAG : 1653
REV : CAAATACCAGAGCAAATTGGTGAAAACGAGGAAGTTCTGTTAG : 817

NFKB2
FWD : ATGGTGGAGCAGAAGGAGCCCTTGATGGAGACAGCAGTCGGGCCCTACCTGGTCATCATCGAGCAGCCGA : 70
NFKB2 : ATGGTGGAGCAGAAGGAGCCCTTGATGGAGACAGCAGTCGGGCCCTACCTGGTCATCATCGAGCAGCCGA : 70

FWD : AGCAGCGGGGCTTCCGATTTCGGTATGGCTGCGAGGGCCCTTCTCACGGGGGGCTGCCAGGCGCCTCCAG : 140
NFKB2 : AGCAGCGGGGCTTCCGATTTCGGTATGGCTGCGAGGGCCCTTCTCACGGGGGGCTGCCAGGCGCCTCCAG : 140

FWD : CGAGAAGGGGCACAAGACCTATCCCACCGTCAAGATCTGCAACTACGAGGGGATGGCGCGCATCGAGGTG : 210
NFKB2 : CGAGAAGGGGCACAAGACCTATCCCACCGTCAAGATCTGCAACTACGAGGGGATGGCGCGCATCGAGGTG : 210

FWD : GACCTGGTGACGCACAGCGACCCTCCGCGTGTGCGCGCGCACAGCCTGGTGGGCAAGCAGTGCAACGAGG : 280
NFKB2 : GACCTGGTGACGCACAGCGACCCTCCGCGTGTGCATGCGCACAGCCTGGTGGGCAAGCAGTGCAACGAGG : 280

FWD : CCGGCAACTGCGTCGCCATCGTGGGGCCCAAAGACATGACGGCACAGTTCAGCAACCTGGGTGTGCTCCA : 350
NFKB2 : CCGGCAACTGCGTCGCCATCGTGGGGCCCAAAGACATGACGGCACAGTTCAGCAACCTGGGTGTGCTCCA : 350

FWD : CGTCACCAAGAAGAACATGATGGAGATCATGAAGGAGAAGCTGAAGAAGCAGAAGACGCGCAACACAAAT : 420
NFKB2 : CGTCACCAAGAAGAACATGATGGAGATCATGAAGGAGAAGCTGAAGAAGCAGAAGACGCGCAACACAAAT : 420

FWD : GGGCTGCTGACAGAAGCTGAGCTGCGTGAGATCGAGCTGGAGGCCAAGGAGCTGAAGAAGGTGATGGACC : 490
NFKB2 : GGGCTGCTGACAGAAGCTGAGCTGCGTGAGATCGAGCTGGAGGCCAAGGAGCTGAAGAAGGTGATGGACC : 490

FWD : TGAGCATCGTGCGGCTGCGCTTCACCGCTTACCTCCGTGACAGCAGTGGGAACTTCACTCTGGCACTACA : 560
NFKB2 : TGAGCATCGTGCGGCTGCGCTTCACCGCTTACCTCCGTGACAGCAGTGGGAACTTCACTCTGGCACTACA : 560

FWD : GCCCGTCATCTCTGACCCCATCCATGACAGCAAGTCCCCCGGCGCTTCCAACCTGAAGATCTCGCGGATG : 630
NFKB2 : GCCCGTCATCTCTGACCCCATCCATGACAGCAAGTCCCCCGGCGCTTCCAACCTGAAGATCTCGCGGATG : 630

FWD : GACAAGACTGCGGGCTCAGTGCGGGGTGGGGACGAGGTGTACCTGCTGTGCGACAAGGTGCAGAAAGATG : 700
NFKB2 : GACAAGACTGCGGGCTCAGTGCGGGGTGGGGACGAGGTGTACCTGCTGTGCGACAAGGTGCAGAAAGATG : 700

FWD : ACATTGAGGTGCGGTTCTATGAGGATGACGAGAACGGCTGGCAGGCCTTCGGGGACTTCTCCCCCACGGA : 770
NFKB2 : ACATTGAGGTGCGGTTCTATGAGGATGACGAGAACGGCTGGCAGGCCTTCGGGGACTTCTCCCCCACGGA : 770

FWD : CGTACACAAGCAGTACGCCATCGTCTTCCGCACGCCCCCCTACCACAAGCCCAAAATTGACCGTCCTGTC : 840
NFKB2 : CGTACACAAGCAGTACGCCATCGTCTTCCGCACGCCCCCCTACCACAAGCCCAAAATTGACCGTCCTGTC : 840

FWD : ACCGTGTTCCTGCAACTGAAGCGGAAGCGCGGTGGGGACGTCAGCGAC...AAGCA.............. : 893
NFKB2 : ACCGTGTTCCTGCAACTGAAGCGGAAGCGCGGTGGGGACGTCAGCGACTCCAAGCAGTTCACCTATTACC : 910

NFKBIZ
NFKBIZ : ATGGGGGGAGGAAAGCAGCACAGAGGGCCGTTCCAGGGGGACCGCGTGAAGAACTCGGTGAAGGAGCTCC : 70
FWD : ATGGGGGGAGGAAAGCAGCACAGAGGGCCGTTCCAGGGGGTCCGCGTGAAGAACTCGGTGAAGGAGCTCC : 70

NFKBIZ : TGCTGCACTTCAGGAGCAGCAAGCAGATGTCCTCGGGCCCCGCTGCGGATGAAGGCAAGGCACAGGGAGG : 140
FWD : TGCTGCACTTCAGGAGCAGCAAGCAGATGTCCTCGGGCCCCGCTGCGGATGAAGGCAAGGCACAGGGAGG : 140

NFKBIZ : ACTGGTGAACTACGAACCATACACAGCAGAGTTGAAGAGCGTTCTAGGTCAGGGTGGCAAAAGAAAGGCT : 210
FWD : ACTGGTGAACTACGAACCATACACAG...AGTTGAAGAGCGTTCTAGGTCAGGGTGGCAAAAGAAAGGCT : 207

NFKBIZ : CCTGAGCTCCTTTCTGATGGACCTCCTTTCAAACGCCAAGTTAATGTTCACCCACACCTCCTGACGCCAC : 280
FWD : CCTGAGCTCCTTCCTGATGGACCTCCTTTCAAACGCCAAGTTAATGTTCACCCACTCCTCCTGACGCCAC : 277

NFKBIZ : CCCAGACCCCAACTTCAATGGATAACATGGAAGAAACGCATAAAAATGAACCAAAGCATGAGAGCAATTC : 350
FWD : CCCAGACCCCAACTTCAATGGATAACATGGAAGGAACGCATAAAAATGAACCAAAGCATGAGAGCAATTC : 347

NFKBIZ : TGATCTGCTTCAGAACATCATAAACATTAAGAACGAGTCCAGCCCTGTTTCCCTGAATACAGTGCAGGTT : 420
FWD : TGATCTGCTTCAGAACATCATAAACATTAAGAACGAGTCCAGCCCTGTTTCCCTGAATACAGTGCAGGTT : 417

NFKBIZ : AGCTGGTTGCACGG.GTCTCCAGCCACAGCTCACCCGGCGAGCAATACCACGACAGTCCGGGAACACAGG : 489
FWD : AGCTGGTTGCACGGTGTCTCCAGCCACAGCTCACCCGGCGAGCAATACCACGACAGTCCGGGAACACAGG : 487

NFKBIZ : CTTTTTCCCCACCCCAGAAGTACCAAGCATTCCAAGATCACACCTCCCAGAGTATGCTTGATCCTCCTCA : 559
FWD : CTTTTTCCCCACCCCAGAAGTACCAAGCATTCCAAGATCACACCTCCCAGAGTATGCTTGATCCTCCTCA : 557

NFKBIZ : GCATTACCAGTTTCCTTCATCGCAGAACCAGGATTTGTTACAGAACTACACTTCGGATGCATCTCTGGAG : 629
FWD : GCATTACCAGTTTCCTTCATCGCAGAACCAGGATTTGTTACAGAACTACACTTCGGATGCATCTCTGGAG : 627

NFKBIZ : TACAGGCCGTTTTCTGCCAATGACCAGTCTCCGGGCTACCACCAGCACGCCTTTGAGAGCCATGAACTGC : 699
FWD : TACAGGCCGTTTTCTGCCAATGACCAGTCTCCGGGCTACCACCAGCACGCCTTTGAGAGCCATGAACTGC : 697

NFKBIZ : AGTATTGCCC.GTCGCAAAGTTTCTCCTCCCTCCTCAATGATTCTGAAGGCTCAGAGACCATCTCTGCTC : 768
FWD : AGTATTGCCCCGTCGCAAAGTTTCTCCTCCCTCCTCAATGATTCTGAAGGCTCAGAGACCATCTCTGCTC : 767

NFKBIZ : CTCCAGTCCTTGCCCAGTGCCCACCCGCAGGCGAACGTCAGCCCCCACGCTCAGAACTTCAGCCTGGCTC : 840
FWD2 : CTCCAGTCCTTGCCCAGTGCCCACCCGCAGGCGAACGTCAGCCCCCACGCTCAGAACTTCAGCCTGGCTC : 135

NFKBIZ : CCAATAATGCCTGCAGTAGTCTTGAACGCAGCATCTCTTTGGCCACTTTGAATGTCTCTCTACCATCCCA : 910
FWD2 : CCAATAATGCCTGCAGTAGTCTTGAACGCAGCATCTCTTTGGCCACTTTGAATGTCTCTCTACCATCCCA : 205

NFKBIZ : AAGTGTTGCCAGGAACACGACGCAGCTGGGCAAGTCGTTTTTCCAGTGGCAAGTGGAGCAGGAGGAAAAC : 980
FWD2 : AAGTGTTGCCAGGAACACGACGCAGCTGGGCAAGTCGTTTTTCCAGTGGCAAGTGGAGCAGGAGGAAAAC : 275

NFKBIZ : AAATTGGCTAACATCTCTCAAGACC.AGTTCCTCGCGAAAGACTCGGATGGAGACACCTTCCTTCACATT : 1049
REV : AAATTGGCTAACATCTCTCAAGACCCAGTTCCTCGCGAAAGACTCGGATGGAGACACCTTCCTTCACATT : 80

NFKBIZ : GCTGTTGCCCAGGGCCGACGAGCTCTCTCCTACGTTCTTGCAAGGAAAA.TGGCTGCTCTGCATATGTTG : 1118
REV : GCTGTTGCCCAGGGCCGACGAGCTCTCTCCTACGATCTTGCAAGGAAAAATGGTTGCTCTGCATATGTTG : 150

NFKBIZ : GATATTAAAGAGCACAATGGCCAGAGTGCTTTCCAGGTTGCTGTGGCTGCCAACCAGCATCTCATTGTGC : 1188
REV : GATATTAAAGAGCACAATGGCCAGAGTGCTTTCCAGGTTGCTGTGGCTGCCAACCAGCATCTCATTGTGC : 220

NFKBIZ : AGGATTTGGTTAGCCTGGGGG.CTCAAGTCAACACCACAGACTGCTGGGGTAGAACGCCATTGCATGTTT : 1257
REV : AGGATTTGGTTAGCCTGGGGGGCTCAAGTCAACACCACAGACTGCTGGGGTAGAACGCCAT.GCATGTTT : 289

NFKBIZ : GCGCTGAGAAGGGGCATGCCCAGGTCCTCCAGGCAATCCAAAAGGGGGCCATGGGAAGCAATCAATATGT : 1327
REV : GCGCTGAGAAGGGGCATGCCCAGGTCCTCCAGGCAATCCAAAAGGGGGCCATGGGAAGCAATCAATATGT : 359

NFKBIZ : GGACCTTGAGGCAACGAACTACGATGGTTTGACAGCCCTGCACTGTGCTGTTTTGGCCCATAACGCTGTG : 1397
REV : GGACCTTGAGGCAACGAACTACGATGGTTTGACAGCCCTGCACTGTGCTGTTTTGGCCCATAACGCTGTG : 429

NFKBIZ : CTGCATGAGCTACAAAACTGTCAACCACCTCACTCCCCTGAGGTTCAGGAACTTCTGCTGAGAAACAAGA : 1467
REV : CTGCATGAGCTACAAAACTGTCAACCACCTCACTCCCCTGAGGTTCAGGAACTTCTGCTGAGAAACAAGA : 499

NFKBIZ : GCCTGGTGGAAACCATCAAGACTCTGATACAAATGGGAGCTTCTGTTGAAGCAAAAGATCGCAAGAGCGG : 1537
REV : GCCTGGTGGAAACCATCAAGACTCTGATACAAATGGGAGCTTCTGTTGAAGCAAAAGATCGCAAGAGCGG : 569

NFKBIZ : TCGCTCTGCTTTACATTTGGCAGCGGAGGAAGCCAATCTGGAGCTCATCCGTCTCTTCTTGGAGCTGCCC : 1607
REV : TCGCTCTGCTTTACATTTGGCAGCGGAGGAAGCCAATCTGGAGCTCATCCGTCTCTTCTTGGAGCTGCCC : 639

NFKBIZ : AACTGCCTCTCTTTTGTGAATGCAAAGGCTTACAATGGCAACACGGCGCTCCATGTGGCTGCCAGCCTGC : 1677
REV : AACTGCCTCTCTTTTGTGAATGCAAAGGCTTACAATGGCAACACGGCGCTCCATGTGGCTGCCAGCCTGC : 709

NFKBIZ : AGTATCGGGTGAGTCAGTTGGATGCAGTTCGCCTGCTAATGAGAAAGGGAGCTGATCCGAGTGCCAGAAA : 1747
REV : AGTATCGGGTGAGTCAGTTGGACGCAGTTCGCCTGCTAATGAGAAAGGGAGCTGATCCGAGTGCCAGAAA : 779

NFKBIZ : CTTGGAAAATGAACAGCCAGTTCACTTGGTTCCTGATGGCCTCGTAGGAGAACAGATAAGACGTATCCTA : 1817
REV : CTTGGAAAATGAACAGCCAGTTCACTTGGTTCCTGATGGCCTCGTAGGAGAACAGATAAGACGTATCCTA : 849

NFKBIZ : AAGGGGAAGACAGTTCAGCAGAGAGCGTCGCTGTTTTGA : 1856
REV : AAGGGGAAGACAGTTCAGCAGAGAGCGTCGCTGTTTTGA : 888

NLRC5
NLRC5 : GAGTGCATATCCTCCAACAACAACGTGACAGTCGATGCTGCAGGGGGGCTTTTACGTTCACTGAGTAAAA : 1120
REV : ..................................ATGCTGCAGGGGGGCTTTTACGTTCACTGAGTAAAA : 36

NLRC5 : CTCCAGGTCCTCTAAAAATCAGCATAGAAGAGCCCTGGGTATGTAAGTTGAGTGTCACGAGCCTTCTTGA : 1190
REV : CTCCAGGTCCTCTAAAAATCAGCATAGAAGAGCCCTGGGTATGTAAGTTGAGTGTCACGAGCCTTCTTGA : 106

NLRC5 : GCTGGCTGTCCAGGCCCATGGAAACATCACTGCGATCATGATATGCAAAGAGAAGAACCTCTTCCAA.CT : 1259
REV : GCTGGCTGTCCAGGCCCATGGAAACATCACTGCGATCATGATATGCAAAGAGAAGAACCTCTTCCAAACT : 176

NLRC5 : AGGTGTAAGGCTTCCACACTGCCTGGAGAAGGTGGGATCAGTCGTTAGCAGATTGAATTTGAACGAGCCA : 1329
REV : AGGTGTAAGGCTTCCACACTGCCTGGAGAAGGTGGGATCAGTCGTTAGCAGATTGAATTTGAACGAGCCA : 246

NLRC5 : GAAATCAAACAGGCTTGCTTCTACCAAAGGGTTCATGACAAATGCACCCAGCTTCAGGAACTGAGATGGT : 1399
REV : GAAATCAAACAGGCTTGCTTCTACCAAAGGGTTCATGACAAATGCACCCAGCTTCAGGAACTGAGATGGT : 316

NLRC5 : CTCACGTTGAACTCCACGATGACACAGAAATGCTCGTCAGTATTTTGCTACCTCTTCCAGATCTGAAGAA : 1469
REV : CTCACGTTGAACTCCACGATGACACAGAAATGCTCGTCAGTATTTTGCTACCTCTTCCAGATCTGAAGAA : 386

NLRC5 : GTTTGAGCTGACCTCTTGCAGCTTTACACCAACTGGAATTGATTGCTTGATCACAGGCTTGCAGAGGTGC : 1539
REV : GTTTGAGCTGACCTCTTGCAGCTTTACACCAACTGGAATTGATTGCTTGATCACAGGCTTGCAGAGGTGC : 456

NLRC5 : CAGGCCATTGAAGAGCTCAACCTGGGCCACATGAAGCTCGGCGATGCTGCCATTCCTAAGCTAGTGTTTG : 1609
REV : CAGGCCATTGAAGAGCTCAACCTGGGCCACATGAAGCTCGGCGATGCTGCCATTCCTAAGCTAGTGTTTG : 526

NLRC5 : GACTCTGTGAAATGCCGTCTCTGAAAAGGCTGATCTTGAACCATAACAGTATTGGTGACGATGGCTGCTC : 1679
REV : GACTCTGTGAAATGCCGTCTCTGAAAAGGCTGATCTTGAACCATAACAGTATTGGTGACGATGGCTGCTC : 596

NLRC5 : CAGACTCGCAGAAGCCCTGAGCAGCATGCACTGCATGGAAGAAATCAATTTAGGCCACAACAAGATTGGA : 1749
REV : CAGACTCGCAGAAGCCCTGAGCAGCATGCACTGCATGGAAGAAATCAATTTAGGCCACAACAAGATTGGA : 666

NLRC5 : GATCTAGGCCTGATAAATATAGCTGCTGTCCTGCTGGAAATGCAAAACTTGAAGAGAATCGACCTCTCAG : 1819
REV : GATCTAGGCCTGATAAATATAGCTGCTGTCCTGCTGGAAATGCAAAACTTGAAGAGAATCGACCTCTCAG : 736

NLRC5 : GGAACTGTCCCAGTCCTGCTGGAGGAGAAAAGCTGATGGAAGCTCTTGCCAATTGCAAGCACCTCGAGGA : 1889
REV : GGAACTGTCCCAGTCCTGCTGGAGGAGAAAAGCTGATGGAAGCTCTTGCCAATTGCAAGCACCTCGAGGA : 806

NLRC5 : GCTAATTTTATCTGAAAACAACCTTGGGGAAGGA........GGTATCCGTGCCCTGTCCG......... : 1942
REV : GCTAATACTGTCAAGGAATGATTTTGGAGATGGGACAGCAGTGAAACTTGCCCTCTGTCTGCCTCACATG : 876

NLRC5 : .AGGGGCTGCCGCGTTTTGA...GCACCTCCGGAAGATTGAGTGA......................... : 1983
REV : AACAGGCTGAAGATCCTGCACTTGCAGCACAATAACATCGGGCCAGCAGGAGGAACGGAGCTGGCCAGAG : 946


NR4A3
NR4A3 : ATGCCCTGTGTGCAAGCGCAGTATAGCCCTTCGCCGCCCGGTTCGAGTTATGCAGCTCAGACCTACGCGT : 70
FWD : ATGCCCTGTGTGCAAGCGCAGTATAGCCCTTCGCCGCCCGGTTCGAGTTATGCAGCTCAGACCTACGCGT : 70

NR4A3 : ATGGCTCGGAGTACAGCTCGGAGATCATGAACCCGGACTATGGCAAGCTGAGCATGGAGCTGAGCGGCAC : 140
FWD : ATGGCTCGGAGTACAGCTCGGAGATCATGAACCCGGACTATGGCAAGCTGAGCATGGAGCTGAGCGGCAC : 140

NR4A3 : CGAGATCACCGCCACCGCCACCACCTCCCTCCCCAGCTTCAGCACCTTCATGGAGGGTTACTCCGGCAGC : 210
FWD : CGAGATCACCGCCACCGCCACCACCTCCCTCCCCAGCTTCAGCACCTTCATGGAGGGTTACTCCGGCAGC : 210

NR4A3 : TACGAGCTCAAGCCTTCCTGCCTCTACCAAATGCAATCCGCCTCCTCCGGCCAGAGGCCCCTCATCAAGA : 280
FWD : TACGAGCTCAAGCCTTCCTGCCTCTACCAAATGCAATCCGCCTCCTCCGGCCAGAGGCCCCTCATCAAGA : 280

NR4A3 : TGGAAGACGCTCGGCTCTCCACCTACCAGCCCTCGCTGCCCCCCTCGGTGGACGAGAGCATGCCCAGCAC : 350
FWD : TGGAAGACGCTCGGCTCTCCACCTACCAGCCCTCGCTGCCCCCCTCGGTGGACGAGAGCATGCCCAGCAC : 350

NR4A3 : CTCCATGTACTTCAAGCAGTCGCCTCCCTCCACGCCCACCACGCCCGGCTTCCCCCCACACCAGAGCCTG : 420
FWD : CTCCATGTACTTCAAGCAGTCGCCTCCCTCCACGCCCACCACGCCCGGCTTCCCCCCACACCAGAGCCTG : 420

NR4A3 : TGGGACGAGCCCCCGCTGCCTCCCACGCAGACCTGCCTGCCCCCCGGCCACCTGATGGAGGCCGCTCCCA : 490
FWD : TGGGACGAGCCCCCGCTGCCTCCCACGCAGACCTGCCTGTCGCCCGGCCACCTGATGGAGGCCGCTCCCA : 490

NR4A3 : TGAAGACTGCGCCTCCGCGCTTCCCCCTCTTCCACTTCAAGCACTCTCCTCCCCACACGCCGCCGGCGGG : 560
FWD : TGAAGACTGCGCCTCCGCGCTTCCCCCTCTTCCACTTCAAGCACTCTCCTCCCCGCCTTCTCCT...... : 554

NR4A3 : CCCCCACATGTGCTACGACCCGGCCTCCCTGAGCCTGCCGCTGGGCTCTGACAGACCGCCGGCCGGCCAG : 630
FWD : ..CCCATCAGCATGATGAAC..GCCCTCGTACGAGCTTTAACCGACTCC.ACGCCCAGGGAGCTCGACTA : 619

NR4A3 : GCGCCCATGGAGAGCCACTCCTACGGGCTGCCCCTGGCCAAGCGGCCGGCCACCTTAGCCTTCTCACCGC : 700
FWD : TTCACGATACTGTTCCACCGAT.CAGGCTGCTGCAGGCACAGATGCAGAACATGTA.......CAACAGT : 681

NR4A3 : TCGGCCTCAACACGGCTGCCTCCGGCCTCATGGGCGAGGCCAGCGGTGGTGGCGGTGGCGGGCTGCCCTC : 770
FWD : TCTATAATCTTCTGACTGCCTCCATTGACATATCCAGAGGCTG.GGCAGAAAAAATTCCAGGATTTACTG : 750

NR4A3 : CCCGCCCAGCAGGAGTTCCTCGTCCGGGGAGGGCACGTGCGCCGTCTGTGGGGACAACGCTGCCTGCCAG : 840
FWD : ACCTCCCGAAAGAAGATC.........AGACATTACTCATAGAATCAGCTTTTTTGGAGCTGTTTG.... : 807

NR4A3 : CACTATGGTGTGCGGACCTGCGAGGGCTGCAAGGGCTTCTTCAAGAGGACGGTTCAGAAAAATGCAAAAT : 910
FWD : ...................................................................... : -

NR4A3 : ATGTTTGCCTGGCAAACAAGAACTGTCCGGTGGACAAGAGACGCCGTAACAGATGTCAGTACTGCCGCTT : 980
FWD : ...................................................................... : -

NR4A3 : TCAGAAGTGTCTCAGCGTCGGCATGGTGAAAGAAGTTGTCCGCACGGACAGCCTGAAAGGGAGAAGAGGT : 1050
REV : ...................................................................... : -

NR4A3 : CGGCTGCCTTCCAAACCAAAGAGCCCCCTGCAGCAGGAACCTTCGCAGCCCTCCCCGCCTTCTCCTCCCA : 1120
REV : ..................................................CTCCCCGCCTTCTCCTCCCA : 20

NR4A3 : TCAGCATGATGAACGCCCTCGTACGAGCTTTAACCGACTCCACGCCCAGGGAGCTCGACTATTCACGATA : 1190
REV : TCAGCATGATGAACGCCCTCGTACGAGCTTTAACCGACTCCACGCCCAGGGAGCTCGACTATTCACGATA : 90

NR4A3 : CTGTTCCACCGATCAGGCTGCTGCAGGCACAGATGCAGAACATGTACAACAGTTCTATAATCTTCTGACT : 1260
REV : CTGTTCCACCGATCAGGCTGCTGCAGGCACAGATGCAGAACATGTACAACAGTTCTATAATCTTCTGACT : 160

NR4A3 : GCCTCCATTGACATATCCAGAGGCTGGGCAGAAAAAATTCCAGGATTTACTGACCTCCCGAAAGAAGATC : 1330
REV : GCCTCCATTGACATATCCAGAGGCTGGGCAGAAAAAATTCCAGGATTTACTGACCTCCCGAAAGAAGATC : 230

NR4A3 : AGACATTACTCATAGAATCAGCTTTTTTGGAGCTGTTTGTACTAAGACTCTCCATCAGGTCTGATACTGC : 1400
REV : AGACATTACTCATAGAATCAGCTTTTTTGGAGCTGTTTGTACTAAGACTCTCCATCAGGTCTGATACTGC : 300

NR4A3 : TGAGGATAAGTTTGTATTCTGCAATGGACTTGTGCTTCATAGACTTCAGTGCCTTCGTGGATTTGGGGAG : 1470
REV : TGAGGATAAGTTTGTATTCTGCAATGGACTTGTGCTTCATAGACTTCAGTGCCTTCGTGGATTTGGGGAG : 370

NR4A3 : TGGCTCGACTCTATTAAAGACTTTTCCTTAAACTTAAAGAGCCTTAACCTTGATATCCCAGCCTTAGCAA : 1540
REV : TGGCTCGACTCTATTAAAGACTTTTCCTTAAACTTAAAGAGCCTTAACCTTGATATCCCAGCCTTAGCAA : 440

NR4A3 : GTTTATCAGCTCTAACTATGATTACAGAACGACATGGCTTAAAAGAACCAAAGAAAGTGGAAGAGCTATG : 1610
REV : GTTTATCAGCTCTAACTATGATTACAGAACGACATGGCTTAAAAGAACCAAAGAAAGTGGAAGAGCTATG : 510

NR4A3 : CAACAAGATCACAAGCAGTTTGAAAGATCACTTAACTTTCAGTTGCCAAAACAAAGGACAGCCGCTCGAG : 1680
REV : CAACAAGATCACAAGCAGTTTGAAAGATCACTTAACTTTCAGTTGCCAAAACAAAGGACAGCCGCTCGAG : 580

NR4A3 : TCCGCAGAGCCTAAGGTACTGGGTGTTCTTGCTGACTTGCGTTCTCTCTGCACACTGGGACTGCAGCGCA : 1750
REV : TCCGCAGAGCCTAAGGTACTGGGTGTTCTTGCTGACTTGCGTTCTCTCTGCACACTGGGACTGCAGCGCA : 650

NR4A3 : TCTTTTACCTGAAACTGGAAGATTTGGTGCCAGCCCCTTCCATTATCGACAGGCTGTTTCTGGACACCTT : 1820
REV : TCTTTTACCTGAAACTGGAAGATTTGGTGCCAGCCCCTTCCATTATCGACAGGCTGTTTCTGGACACCTT : 720

NR4A3 : GCCCTTCTGA : 1830
REV : GCCCTTCTGA : 730

PFKFB3
PFKFB3 : GCCAATTCCCCCACTGTGATAGTGATGGTTGGCCTCCCAGCCCGGGGGAAGACTTACATCTCCAAGAAGC : 70
FWD : GCCAATTCCCCCACTGTGATAGTGATGGTTGGCCTCCCAGCCCGGGGGAAGACTTACATCTCCAAGAAGC : 70

PFKFB3 : TGACTCGCTATCTCAACTGGATCGGTGTCCCAACAAAAGTTTTCAATGTAGGGGAGTATCGCCGTGAGGC : 140
FWD : TGACTCGCTATCTCAACTGGATCGGTGTCCCAACAAAAGTTTTCAATGTGGGGGAGTATCGCCGTGAGGC : 140

PFKFB3 : AGTGAAGCATTACAGCTCCTATGACTTCTTTCGCCCCGACAACGAAGAGGCTATGAAAGTCAGGAGGCAG : 210
FWD : AGTGAAGCATTACAGCTCCTATGACTTCTTTCGCCCCGACAACGAAGAGGCTATGAAAGTCAGGAGGCAG : 210

PFKFB3 : TGTGCTCTGGCTGCCCTGAGGGATGTCAAGCTGTACCTGACAGAGGAGGCTGGTCAGATTGCGGTTTTTG : 280
FWD : TGTGCTCTGGCTGCCCTGAGGGATGTCAAGCTGTACCTGACGGAGGAGGCTGGTCAGATCGCGGTTTTTG : 280

PFKFB3 : ATGCCACAAATACCACACGGGAGAGGAGAGGGATGATCCTAAATTTTGCCAAAGAAAATGGGTTCAAGGT : 350
FWD : ATGCCACAAATACCACACGGGAGAGGAGAGGGATGATCCTAAATTTTGCCAAAGAAAATGGGTTCAAGGT : 350

PFKFB3 : GTTCTTCATTGAATCTGTCTGCAACGATCCCAATGTTGTTGCCACCAATGTCATGGAAGTAAAACTGTCC : 420
FWD : GTTCTTCATTGAATCTGTCTGCAACGATCCCAATGTTGTTGCCACCAATGTCATGGAAGTAAAACTGTCC : 420

PFKFB3 : AGTCCTGATTACCGGGACTGTAATTCGACCGATGCCATGGAGGACTTCATGAAGAGAATCAATTGTTACC : 490
FWD : AGTCCTGATTACCGGGACTGTAATTCGACCGATGCCATGGAGGACTTCATGAAGAGAATCAATTGTTACC : 490

PFKFB3 : AGGCCAGCTACCAGCCACTTGACCCTGATGACTATGACCGGGAGCTTTCTCTCATCAAAGTCATCGATGT : 560
FWD : AGGCCAGCTACCAGCCACTTGACCCTGATGACTATGACCGGGAGCTTTCTCTCATCAAAGTCATCGATGT : 560

PFKFB3 : TGGCCGGCGGTTCCTGGTCAACAGGGTTCAGGATCACATCCAAAGCAGGATTGTTTACTACCTAATGAAC : 630
FWD : TGGCCGGCGGTTCCTGGTCAACAGGGTTCAGGATCACATCCAAAGCAGGATTGTTTACTACCTAATGAAC : 630

PFKFB3 : ATCCATGTCCAGCCCCGCACCATTTATCTCTGTCGGCACGGTGAGAGCGAGTTCAACCTCAAGGGGAAGA : 700
REV : ATCCATGTCCAGCCCCGCACCATTTATCTTTGTCGGCACGGTGAGAGCGAGTTCAACCTCAAGGGGAAGA : 167

PFKFB3 : TTGGAGGTGACTCAGGCCTCTCCAATAGGGGCAAGAAGTTTGCACTGGCACTGAACAAGTTTGTTGAGGA : 770
REV : TTGGAGGTGACTCAGGCCTCTCCAACAGGGGCAAGAAGTTTGCACTGGCACTGAACAAGTTTGTTGAGGA : 237

PFKFB3 : GCAGAACCTGAAGGACCTCAAAGTCTGGACCAGCCAACTGAAGAGAACAATCCAAACAGCAGAAGCACTC : 840
REV : GCAGAACCTGAAGGACCTCAAAGTCTGGACCAGCCAACTGAAGAGAACAATCCAAACAGCAGAAGCACTC : 307

PFKFB3 : CAGCTGCCCTACGAGCAGTGGAAGGCCCTCAATGAAATAGATGCTGGTGTGTGTGAAGAAATGACTTATG : 910
REV : CAGCTGCCCTATGAGCAGTGGAAGGCCCTCAATGAAATAGATGCTGGTGTGTGTGAAGAAATGACTTATG : 377

PFKFB3 : AAGAAATCAGGGACCAGCATCCAGAGGAATTTGCTTTACGTGATCAGGATAAATATTACTACCGCTATCC : 980
REV : AAGAAATCAGGGACCAGCATCCAGAGGAATTTGCTTTACGTGATCAGGATAAATATTACTACCGCTATCC : 447

PFKFB3 : TTCTGGAGAGTCCTACCAAGACCTGGTGCAGCGCCTGGAGCCAGTCATCATGGAGCTGGAGAGACAAGAG : 1050
REV : TTCTGGAGAGTCCTACCAAGACCTGGTGCAGCGCCTGGAGCCAGTCATCATGGAGCTGGAGAGACAAGAG : 517

PFKFB3 : AATGTCCTTGTGATCTGTCACCAGGCTGTCATGCGCTGTCTCCTTGCTTACTTCTTGGACAAGAGTGCAG : 1120
REV : AATGTCCTTGTGATCTGTCACCAGGCTGTCATGCGCTGTCTCCTTGCTTACTTCTTGGACAAGAGTGCAG : 587

PFKFB3 : ATGAAATGCCCTACCTGAAATGTCCTCTTCACACGGTATTGAAGTTGACCCCAGTGGCTTATGGCTGCCG : 1190
REV : ATGAAATGCCCTACCTGAAATGTCCTCTTCACACGGTATTGAAGTTGACCCCAGTGGCTTATGGCTGCCG : 657

PKD2L1
PKD2L1 : ATGGAGGGGAAGTGCTGCTTCTACATCTTCAGAGGCATCCGAGGTCCCAAAGGTCTGTGGGGCACTACGC : 70
FWD : ATGGAGGGGAAGTGCTGCTTCTACATCTTCAGAGGCATCCGAGGTC.........TGTGGGGCACTACGC : 61

PKD2L1 : TAACTGAGAACACAGCTGAAAACAGGGAACTTTATGTGAAGACTACTCTGAGAGAGCTCCTAGTCTACAT : 140
FWD : TAACTGAGAACACAGCTGAAAACAGGGAACTTTATGTGAAGACTACTCTGAGAGAGCTTCTAGTCTACAT : 131

PKD2L1 : TGTGTTCCTGGTGGACATCTGTCTGCTGACCTATGGTATGACAAGTTCCAGTGCCTATTACTACACTAAA : 210
FWD : TGTGTTCCTGGTGGACATCTGTCTGCTGACCTATGGTATGACAAGTTCCAATGCCTATTACTACACTAAA : 201

PKD2L1 : GTGATGTCCGAGCTATTCCTGCAGACCTCTTCGGACAGCCGTGTCTCCTTCCAGTCCATCGGCAGCATGG : 280
FWD : GTGATGTCCGAGCTATTCCTGCAGACCTCTTCGGACAGCCGTGTCTCCTTCCAGTCCATTGGCAGCATGG : 271

PKD2L1 : CTGACTTCTGGGTGTATGCACAAGGTCCTCTTCTGGATAATCTTTACTGGACAAAATGGTATAACAATGA : 350
FWD : CTGACTTCTGGGTGTATGCACAAGGTCCTCTTCTGGATAATCTTTACTGGACAAAATGGTATAACAATGA : 341
REV : .............................................ACTGGACAAAAATGGTATAACAATG : 25

PKD2L1 : GTCCCTAGCAGCGCACAACACTCAGTCATACATCTATTATGAGAACCTGTTACTGGGTGTCCCACGCATG : 420
FWD : GTCCCTAGCAGCACACAACACTCAGTCATACATCTATTATGAGAACCTGTTACTGGGTGTCCCACGCATG : 411
REV : AGTCCCTAGCAGCACACAACACTCAGTCATACATCTATTATGAGAACCTGTTACTGGGTGTCCCACGCAT : 95


PKD2L1 : CGACAGCTGAAGGTGAAGAACAATTCCTGTGTGGTTCATGATGACTTCAAAGAGGAGATCTCAGGCTGCT : 490
FWD : CGACAGCTGAAGGTGAAGAACAATTCCTGTGTGGTTCATGATGACTTCAAAGAGGAGATCTCAGGCTGCT : 481
REV : GCGACAGCTGAAGGTGAAGAACAATTCCTGTGTGGTTCATGATGACTTCAAAGAGGAGATCTCAGGCTGC : 165

PKD2L1 : ACGACGTATACTCAGAAGACAAAGAGGAAAAGGTCTCCTTTGGACTCATCAATGGAACAGCGTGGAGGTA : 560
FWD : ACGACGTATACTCAGAAGACAAAGAGGAAAAGGTCTCCTTTGGACTCATCAATGGAACAGCGTGGAGGTA : 551
REV : TACGACGTATACTCAGAAGACAAAGAGGAAAAGGTCTCCTTTGGACTCATCAATGGAACAGCGTGGAGGT : 235

PKD2L1 : CCATTCTGAGGAAGAGCTGGGTGGCTCATCTCACTGGGGAAGACTAACCAGTTACAGTGGGGGAGGATAC : 630
FWD : CCATTCTGAGGAAGAGCTGGGTGGCTCATCTCACTGGGGAAGACTAACCAGTTACAGTGGGGGAGGATAC : 621
REV : ACCATTCTGAGGAAGAGCTGGGTGGCTCATCTCACTGGGGAAGACTAACCAGTTACAGTGGGGGAGGATA : 305

PKD2L1 : TACATAGACCTCAAGTTGACCAGGGAAGAGAGTGCTGAAGCCCTGCAAATCTTGAAGGAGAAGTTATGGT : 700
FWD : TACATAGACCTCAAGTTGACCAGGGAAGAGAGTGCTGAAGCCCTGCAAATCTTGAAGGAGAAGTTATG.. : 689
REV : CTACATAGACCTCAAGTTGACCAGGGAAGAGAGTGCTGAAGCCCTGCAAATCTTGAAGGAGAAGTTATG. : 374

PKD2L1 : TTGGATCGGGGGACACGGGTTGTCTTCATTGATTTCTCAGTGTATAATGCAAATATCAATCTGTTCTGCG : 769
REV : ...................................................................... : -

PKD2L1 : TTCTGAGGTTAGTGGTTGAGTTTCCCGCCACTGGTGGTGCCATTCCCTCCTGGCAAATCCGGACAGTCAA : 839
REV : ...................................................................... : -

PKD2L1 : GCTTATACGATATGTCAGCGCATGGGATTTTTTCATTGTTGCCTGTGAAATTGTTTTCTGTGTCTTCATC : 909
REV : ...................................................................... : -

PKD2L1 : TTCTACTATGTGGTGGAGGAGATTTTGGAGCTGCGTATCCATAGGCTTCAGTACTTTACCAGCATATGGA : 979
REV : ...................................................................... : -

PKD2L1 : ACATCTTGGATGTGGTTGTCATTCTGCTCTCCATTGTTGCTATTGGATTTCACATCTTTCGTACCATTGA : 1049
REV : ..........................CTCTCCATTGTTGCTATTGGATTTCACATCTTTCGTACCATTGA : 418

PKD2L1 : GGTGAACAGACTGTTGGGGGAGCTGCTGAAACATCCTGAAACCTACGCAGACTTTGAATTCCTAGCGTTC : 1119
REV : GGTGAACAGACTGTTGGGGGAGCTGCTGAAACATCCTGAAACCTACGCAGACTTTGAATTCCTAGCGTTC : 488

PKD2L1 : TGGCAGACTCAGTACAATAATATGAATGCAGTCAACTTATTCTTTGCTTGGATCAAGATATTCAAGTATA : 1189
REV : TGGCAGACTCAGTACAATAATATGAATGCAGTCAACTTATTCTTTGCTTGGATCAAGATATTCAAGTATA : 558


PKD2L1 : TTAGCTTTAACAAAACAATGACTCAGCTGTCCTCCACGTTGGCTCGTTGTGCCAAGGACATCCTGGGCTT : 1259
REV : TTAGCTTTAACAAAACAATGACTCAGCTGTCCTCCACGTTGGCTCGTTGTGCCAAGGACATCCTGGGCTT : 628

PKD2L1 : TGCCATTATGTTCTTTATTGTCTTCTTTGCCTATGCCCAGTTGGGTTACCTTCTTTTTGGGACACAAGTG : 1329
REV : TGCCATTATGTTCTTTATCGTCTTCTTTGCCTATGCCCAGTTGGGTTACCTTCTTTTTGGGACACAAGTG : 698

PKD2L1 : GAAAACTTCAGTACCTTTGTTAAATGCATCTTCACCCAATTTCGGATCATTCTTGGTGACTTTGACTACA : 1399
REV : GAAAACTTCAGTACCTTTGTTAAATGCATCTTCACCCAATTTCGGATCATTCTTGGTGATTTTGACTACA : 768

PKD2L1 : ATTCCATTGACAATGCCAACAGGGTCCTTGGGCCTGTTTATTTCGTCACCTACGTGTTCTTTGTTTTCTT : 1469
REV : ATTCCATTGACAATGCCAACAGGGTCCTTGGGCCTGTTTATTTCGTCACCTACGTGTTCTTTGTTTTCTT : 838

PKD2L1 : TGTGCTCCTGAACATGTTTCTGGCCATCATCAATGACACCTACTCAGAAGTCAAGGAGGAACTTTCAAGC : 1539
REV : TGTGCTCCTGAACATGTTTCTGGCCATCATCAATGACACCTACTCAGAAGTCAAGGAGGAACTTTCAAGC : 908

PKD2L1 : CAGAAGGATGAGCTGCAGC : 1558
REV : CAGAAGGATGAGCTGCAGC : 927

PLA2G5
REV : ATGAATGCTCTCCTTGCATTGGCCATACTGTTTGCTTGGGGCTCATCACTGGCTGGCGGGAGCCTCTGGC : 70
PLA2G5 : ATGAATGCTCTCCTTGCATTGGCCATACTGTTTGCTTGGGGCTCATCACTGGCTGGCGGGAGCCTCTGGC : 70
FWD : ATGAATGCTCTCCTTGCATTGGCCATACTGTTTGCTTGGGGCTCATCACTGGCTGGCGGGAGCCTCTGGC : 70

REV : AGCTGCAGGAGGTGGTCACAAAGATGACGGGGAAAAACGCCGTGCTGAATTACTCCTCCTATGGCTGCTA : 140
PLA2G5 : AGCTGCAGGAGGTGGTCACAAAGATGACGGGGAAAAACGCCGTGCTGAATTACTCCTCCTATGGCTGCTA : 140
FWD : AGCTGCAGGAGGTGGTCACAAAGATGACGGGGAAAAACGCCGTGCTGAATTACTCCTCCTATGGCTGCTA : 140

REV : CTGCGGCGTGGGGGGCCACGGGCAGCCAAAGGATGCCACAGACAGGTGCTGCCAGCTGCACGACACCTGC : 210
PLA2G5 : CTGCGGCGTGGGGGGCCACGGGCAGCCAAAGGATGCCACAGACAGGTGCTGCCAGCTGCACGACACCTGC : 210
FWD : CTGCGGCGTGGGGGGCCACGGGCAGCCAAAGGATGCCACAGACAGGTGCTGCCAGCTGCACGACACCTGC : 210

REV : TATGACAGCCTCCAGCGGTACCACTGCAACGCCAAGAAGCAGCGCTATAAGTACAGCTGGCACAGCGGCC : 280
PLA2G5 : TATGACAACCTCCAGCGGTACCACTGCAACGCCAAGAAGCAGCGCTATAAGTACAGCTGGCACAGCGGCC : 280
FWD : TATGACAGCCTCCAGCGGTACCACTGCAACGCCAAGAAGCAGCGCTATAAGTACAGCTGGCACAGCGGCC : 280

REV : GCCTCACCTGCAACAGGGACTCGTGGTGCGCCCAGCTGTCCTGCGAGTGTGACCGCAGCCTGGGGCTGTG : 350
PLA2G5 : GCCTCACCTGCAACAGGGACTCGTGGTGCGCCCAGCTGTCCTGCGAGTGTGACCGCAGCCTGGGGCTGTG : 350
FWD : GCCTCACCTGCAACAGGGACTCGTGGTGCGCCCAGCTGTCCTGCGAGTGTGACCGCAGCCTGGGGCTGTG : 350

REV : CCTGCAGAGGAACAGAGGGAGCTACAACTGGCGCTACGTCCTGTACCCCAGGTGCAAGTGCAGGTGA : 417
PLA2G5 : CCTGCAGAGGAACAGAAGGAGCTACAACTGGCGCTACGTCCTGTACCCCAGGTGCAAGTGCAGGTGA : 417
FWD : CCTGCAGAGGAACAGAGGGAGCTACAACTGGCGCTACGTCCTGTACCCCAGGTGCAAGTGCAGGTGA : 417

PLK3
PLK3 : ATGACGGACCTCTCCAGCAACAAAACCTATGCCGTGAAGGTCATCCCTCACAGCCGGGTGGCTAAACCCC : 70
FWD : ATGACGGACCTCTCCAGCAACAAAACCTATGCCGTGAAGGTCATCCCTCACAGCCGGGTGGCTAAACCCC : 70

PLK3 : ACCAACGGGAGAAGATCACCAATGAGATCGAGCTGCACCGTGACCTGCACCACAAGCACATCGTCAAGTT : 140
FWD : ACCAACGGGAGAAGATCACCAATGAGATCGAGCTGCACCGTGACCTGCACCACAAGCACATCGTCAAGTT : 140

PLK3 : CTCCCACTATTTTGAGGATGCGGAGAGCATCTACATCTTCTTGGAGCACTGCAGTCGGAAGTCACTGGCC : 210
FWD : CTCCCACTATTTTGAGGATGCGGAGAGCATCTACATCTTCTTGGAGCACTGCAGTCGGAAGTCACTGGCC : 210

PLK3 : CACATCTGGAAGGCCCGCCACACTCTGCTGGAGCCCGAAGTGCGCTATTACCTCAAACAAATCATCTCAG : 280
FWD : CACATCTGGAAGGCCCGCCACACTCTGCTGGAGCCCGAAGTGCGCTATTACCTCAAACAAATCATCTCAG : 280

PLK3 : GCCTCAAATACCTCCACCTCAAGGGCATCCTGCACCGGGACCTCAAGTTGGGCAACTTCTTCATCAATGA : 350
FWD : GCCTCAAATACCTCCACCTCAAGGGCATCCTGCACCGGGACCTCAAGTTGGGCAACTTCTTCATCAATGA : 350

PLK3 : GAACATGGAGCTGAAAGTGGGGGACTTTGGACTGGCTGCCTACCAGGATGCCTCTGACCAGAAGAAGAAG : 420
FWD : GAACATGGAGCTGAAAGTGGGGGACTTTGGGCTGGCTGCCTACCAGGATGCCTCTGACCAGAAGAAGAAG : 420

PLK3 : ACAATATGTGGGACCCCCAACTACCTGGCCCCTGAGGTGCTGTTGCGGCAGGGGCACGGCCCCGAGTCGG : 490
FWD : ACAATATGTGGGACCCCCAACTACCTGGCCCCTGAGGTGCTGTTGCGGCAGGGGCACGGCCCTGAGTCGG : 490

PLK3 : ACGTGTGGTCGCTGGGCTGTGTTATGTACACCTTGCTGTGTGGGAACCCTCCCTTTGAGACCCTGGACCT : 560
FWD : ACGTGTGGTCGCTGGGCTGTGTTATGTACACCTTGCTGTGTGGGAACCCTCCCTTTGAGACCCTGGACCT : 560

PLK3 : GAAGGAGACCTATAGGTACATCAAGCAAGTGGACTACATCCTGCCCACCTTCCTCTCGCTGCCTGCCAGG : 630
FWD : GAAGGAGACCTATAGGTACATCAAGCAAGTGGACTACATCCTGCCCACCTTCCTCTCGCTGCCTGCCAGG : 630

PLK3 : CACCTCATTGCTGGCATTCTCAAGCGCAACCCCCAGGACCGCCTCACACTCGATGAGATTTTGGACCATG : 700
FWD : CACCTCATTGCTGGCATTCTCAAGCGCAACCCCCAGGACCGCCTCACACTCGATGAGATTTTGGACCATG : 700

PLK3 : AGTTCTTCAAGGGTTACACACCCGAGAAGCTCCCTCCCAGCAGCTGCGTGATGGCTCCAGAGCTGTGTGC : 770
FWD : AGTTCTTCAAGGGTTACACACCCGAGAAGCTCCCTCCCAGCAGCTGCGTGATGGCTCCAGAGCTGTGTGC : 770

PLK3 : CCCCAACCCAGCCAAGAGTCTGTTTGCTAAAGTCACCAAGACGCTCTTTAGGAAGAAGAAACCTAAGGCC : 840
FWD : CCCCAACCCAGCCAAGAGTCTGTTTGCTAAAGTCACCAAGACGCTCTTTAG................... : 821

PLK3 : AAGAAGGGCTCTCTGGAAGACAAGGATGACATCTCCAAGCTGGTCACTGGGCTGGTGAAGACGTCCATCT : 910
REV : ...................................................................... : -

PLK3 : GCCGGCAGATGAGCTGCAAGGCGGCCACCCCTGTGTCCTGCTGCAGTGCCGGCTCCAGCCCTGTGGAGAC : 980
REV : .GCGGTAGAGGGG....AATGAGGCCACCCCTGTGTCCTGCTGCAGTGCCGGCTCCAGCCCTGTGGAGAC : 65

PLK3 : GCTGGTAGAGGAGACGTCCCGCAAGTCCACGTCCCCCTCTGTCCGTGGGACCATGGCCAGCAGCTGTGAG : 1050
REV : GCTGGTAGAGGAGACGTCCCGCAAGTCCACGTCCCCCTCTGTCCGTGGGACCATGGCCAGCAGCTGTGAG : 135

PLK3 : GCCTTTGAAGACTGCATCACCACCTCCGCCATCATTGAGTCTGCTGTGCAGCTCCTGCGCACCTGCCTCT : 1120
REV : GCCTTTGAAGACTGCATCACCACCTCTGCCATCATTGAGTCTGCTGTGCAGCTCCTGCGCACCTGCCTCT : 205

PLK3 : CCTGCATGCCCCCAGCGGAGAGAAACCCGGCTTCCCTGGCCCGACATGAGCAGTTTGTTTGGGTGAGCAA : 1190
REV : CCTGCATGCCCCCAGCGGAGAGAAACCCGGCTTCCCTGGCCCGACATGAGCAGTTTGTTTGGGTGAGCAA : 275

PLK3 : GTGGGTGGACTACTCCAACAAGTATGGCTTTGGCTACCAGCTCTCCAACCACAGCATTGGGGTCCTCTTC : 1260
REV : GTGGGTGGACTACTCCAACAAGTATGGCTTTGGCTACCAGCTCTCCAACCACAGCATTGGGGTCCTCTTC : 345

PLK3 : AACAACGGCACGCACATGATGCTGTCCCCCAACCACAAGACAGTGCATTACAACCCAACCAACAGCAAGC : 1330
REV : AACAACGGCACCCACATGATGCTGTCCCCCAACCACAAGACAGTGCATTACAACCCAACCAACAGCAAGC : 415

PLK3 : ACTTTGCATTCCCTGTATCCGCTGTCCCTGAGCAGCTGCGTGGGCAGATGAGTGTCTTGCGCTATTTTGC : 1400
REV : ACTTTGCATTCCCTGTATCCGCTGTCCCTGAGCAGCTGCGTGGGCAGATGAGTGTCTTGCGCTATTTTGC : 485

PLK3 : ATCCTATATGGAGCAGCATCTCATGAAGGGAGGTGACTTGCCTAGCATAGATGACCTTGGACAGCCAGCC : 1470
REV : ATCCTATATGGAGCAGCATCTCATGAAGGGAGGTGACTTGCCTAGCATAGATGACCTTGGACAGCCAGCC : 555

PLK3 : CTACTCCTCCTGCAGTGGGTGAAGACTGACCAGGCTTTGCTCATGCTTTTTAGCAATGGCACCCTTCAGG : 1540
REV : CTACTCCTCCTGCAGTGGGTGAAGACTGACCAGGCTTTGCTCATGCTTTTTAGCAATGGCACCCTTCAGG : 625

PLK3 : TGAACTTCTACAATGACCACACCAAGGTGATCATCAGCAAGCCTGACCATTCATGCCTCGTCACCTACAT : 1610
REV : TGAACTTCTACAATGACCACACCAAGGTGATCATCAGCAAGCCTGACCATTCATGCCTCGTCACCTACAT : 695

PLK3 : CAACCGGGAGCGTAACTCCTACACCTACAAGCTGTGCAGCATCCAGGAGCTGGGCTGCTCACCTGAGCTC : 1680
REV : CAACCGGGAGCGTAACTCCTACACCTACAAGCTGTGCAGCATCCAGGAGCTGGGCTGCTCACCTGAGCTC : 765

PLK3 : CAGCACTGCCTCCGATACATCCTCAAGCTCCTCCAGGAGTGGGCTGATGCCTAG : 1734
REV : CAGCACTGCCTCCGATACATCCTCAAGCTCCTCCAGGAGTGGGCTGATGCCTAG : 819

PPARG
PPARG : ATGGTTGACACAGAAATGCCGTTTTGGCCCGTTAATTTTGGAATTAGCCCAGTGGATCTGTCTGCGATGG : 70
REV : ATGGTTGACACAGAAATGCCGTTTTGGCCCGTTAATTTTGGAATTAGCCCAGTGGATCTGTCTGCGATGG : 70

PPARG : ATGATCATATGCATTCCTTTGACATAAAGCCATTTACCACTGTTGATTTTTCAAGCATTTCTTCACCACA : 140
REV : ATGATCATATGCATTCCTTTGACATAAAGCCATTTACCACTGTTGATTTTTCAAGCATTTCTTCACCACA : 140

PPARG : CTATGAAGATATACCTCTTGGAAGAGCTGATCAAACAAGCATTGATTATAAATATGATATCAAGCTCCAG : 210
REV : CTATGAAGATATACCTCTTGGAAGAGCTGATCAAACAAGCATTGATTATAAATATGATATCAAGCTCCAG : 210

PPARG : GATTGCCAAAGTGCAATCAAAATGGAGCCTCCTTCTCCTCCCTATTTTTCTGAAAAAGTGCAGTTATACA : 280
REV : GATTGCCAAAGTGCAATCAAAATGGAGCCTCCTTCTCCTCCCTATTTTTCTGAAAAAGTGCAGTTATACA : 280

PPARG : ATAAACCTCACGAGGAGTCTTCCAACTCACTTATGGCTATTGAATGTCGTGTGTGTGGGGACAAGGCCTC : 350
REV : ATAAACCTCATGAGGAGTCTTCCAACTCACTTATGGCTATTGAATGTCGTGTGTGTGGGGACAAGGCCTC : 350

PPARG : TGGATTTCATTATGGAGTGCATGCATGTGAAGGTTGTAAGGGCTTTTTTCGAAGAACAATCAGATTAAAG : 420
REV : TGGATTTCATTATGGAGTGCATGCATGTGAAGGTTGTAAGGGCTTTTTTCGAAGAACAATCAGATTAAAG : 420

PPARG : CTTATTTATGATAGGTGTGACCTTAATTGTCGCATCCATAAGAAAAGCAGAAATAAGTGTCAATACTGCA : 490
REV : CTTATTTATGATAGGTGTGACCTTAATTGTCGCATCCATAAGAAAAGCAGAAATAAGTGTCAATACTGCA : 490

PPARG : GATTTCAGAAATGTCTTGCAGTTGGAATGTCACATAATGCCATCAGGTTTGGGCGAATGCCACAAGCGGA : 560
FWD : GATTTCAGAAATGTCTTGCAGTTGGAATGTCACATAATGCCATCAGGTTTGGGCGAATGCCACAAGCGGA : 111

PPARG : GAAGGAGAAGCTCTTGGCAGAGATTTCCAGCGACATCGACCAGTTAAATCCTGAATCTGCTGATCTGCGA : 630
FWD : GAAGGAGAAGCTCTTGGCAGAGATTTCCAGCGACATCGACCAGTTAAATCCTGAATCTGCTGATCTGCGA : 181

PPARG : GCGCTTGCCAAGCATTTGTATGACTCATACATAAAGTCCTTCCCGCTGACCAAAGCCAAGGCAAGGGCGA : 700
FWD : GCGCTTGCCAAGCATTTGTATGACTCATACATAAAGTCCTTCCCTCTGACCAAAGCCAAGGCAAGGGCGA : 251

PPARG : TCTTGACAGGAAAGACGACAGACAAATCACCATTTGTTATTTATGACATGAACTCTTTAAGGATGGGAGA : 770
FWD : TCTTGACAGGAAAGACGACAGACAAATCACCATTTGTTATTTATGACATGAACTCTTTAAGGATGGGAGA : 321

PPARG : AGATCAGATCAAGTGTAAGCATGCATCACCACTGCAGGAACAGAACAAAGAAGTAGCAATTCGCATTTT. : 839
FWD2 : AGATCAGATCAAGTGTAAGCATGCATCACCACTGCAGGAACAGAACAAAGAAGTAGCAATTCGCATTTTT : 86

PPARG : CCAGCGATGTCAGTTTCGCTCTGTGGAGGCAGTGCAGGAGATTACAGAATTTGCGAAGAACATTCCAGGT : 909
FWD2 : CCAGCGATGTCAGTTTCGCTCTGTGGAGGCAGTGCAGGAGATTACAGAATTTGCGAAGAACATTCCAGGT : 156

PPARG : TTTGTGAATCTTGACCTGAATGATCAGGTAA.CCCTCCTGAAATATGGTGTCCATGAGATCATATATACT : 978
FWD2 : TTTGTGAATCTTGACCTGAATGATCAGGTAAACTCTCCTGAAATATGGTGTCCATGAGATCATATATACT : 226

PPARG : CTCCTGGCTTCTCTCATGAATAAAGATGGAGTTCTTATATCTGATGGACAAGGATTCATGACACGGGAGT : 1048
FWD2 : CTCCTGGCTTCTCTCATGAATAAAGATGGAGTTCTTATATCTGATGGACAAGGATTCATGACACGGGAGT : 296

PPARG : TTCTGAAGAGTCTGAGAAAACCTTTTTGTGACTTTATGGAGCCCAAGTTTGAGTTTGCTGTGAAGTTCAA : 1118
FWD2 : TTCTGAAGAGTCTGAGAAAACCTTTTTGTGACTTTATGGAGCCCAAGTTTGAGTTTGCTGTGAAGTTCAA : 366

PPARG : CGCACTGGAATTAGATGACAGTGACCTGGCAATATTTATAGCTGTCATTATACTAAGTGGAGATCGCCCA : 1188
FWD2 : TGCACTGGAATTAGATGACAGTGACCTGGCAATATTTATAGCTGTCATTATACTAAGTGGAGATCGCCCA : 436

PPARG : GGTTTGTTAAATGTGAAGCCCATTGAAGATATACAAGATAATCTGTTGCAAGCTTTGGAGCTCCAGCTAA : 1258
FWD2 : GGTTTGTTAAATGTGAAGCCCATTGAAGATATACAAGATAATCTGTTGCAAGCTTTGGAGCTCCAGCTAA : 506

PPARG : AGCTGAATCATCCAGAGTCATCACAGCTGTTTGCAAAATTGCTTCAGAAAATGACGGACCTCAGACAAAT : 1328
FWD2 : AGCTGAATCATCCAGAGTCATCACAGCTGTTTGCAAAATTGCTTCAGAAAATGACGGACCTCAGACAAAT : 576

PPARG : TGTAACGGAACACGTGCAGCTGTTGCAAATAATAAAGAAAACGGAAACAGATATGAGTCTTCATCCACTC : 1398
FWD2 : TGTAACGGAACACGTGCAGCTGTTGCAAATAATAAAGAAAACGGAAACAGATATGAGTCTTCATCCACTC : 646

PPARG : CTACAAGAAATCTATAAAGACTTATATTAA : 1433
FWD2 : CTACAAGAAATCTATAAAGACTTATATTAA : 676

PTGS2
PTG2 : ATGACAACAGGATTTGATCGGTATGAATGTGACTGCACGAGGACGGGCTATTATGGGGAAAACTGTACAA : 70
FWD : ATGACAACAGGATTTGATCGGTATGAATGTGACTGTACGAGGACGGGCTATTATGGGGAAAACTGTACAA : 70

PTG2 : CACCGGAATTCTTCACGTGGCTGAAACTAATATTGAAACCTACACCAAATACTGTCCACTACATTCTCAC : 140
FWD : CACCGGAATTCTTCACGTGGCTGAAACTAATATTGAAACCTACACCAAATACTGTCCACTACATTCTCAC : 140

PTG2 : CCACTTCAAAGGAGTCTGGAACATCATCAACAACATTTCCTTCTTACGAGATACTATTATGAGATACGTG : 210
FWD : CCACTTCAAAGGAGTCTGGAACATCATCAACAACATTTCCTTCTTACGAGATACTATTATGAGATACGTG : 210

PTG2 : TTGACGTCGAGATCACACTTGATTGACAGCCCACCAACATACAATAGTGACTACAGTTACAAATCCTGGG : 280
FWD : TTGACGTCGAGATCACACTTGATTGACAGCCCACCAACATACAATAGTGACTACAGTTACAAATCCTGGG : 280

PTG2 : AAGCTTATTCCAATCTTTCCTATTACACAAGAAGCCTTCCACCAGTAGGACATGACTGTCCAACACCAAT : 350
FWD : AAGCTTATTCCAATCTTTCCTATTACACAAGAAGCCTTCCACCGGTAGGACATGACTGTCCAACACCAAT : 350

PTG2 : GGGTGTTAAAGGTAAGAAAGAGCTCCCAGATTCAAAGCTGATTGTGGAGAAATTCTTGCTGCGGAGAAAA : 420
FWD : GGGTGTTAAAGGTAAGAAAGAGCTCCCAGATTCAAAGCTGATTGTGGAGAAATTCTTGCTGCGGAGAAAA : 420

PTG2 : TTTATTCCTGACCCACAAGGCACAAATGTGATGTTCACATTCTTTGCCCAGCACTTCACTCATCAATTCT : 490
FWD : TTTATTCCTGACCCACAAGGCACAAATGTGATGTTCACATTCTTTGCCCAGCACTTCACTCATCAATTCT : 490

PTG2 : TTAAGACAGACCATAAGAAAGGGCCTGGCTTCACTAAAGCTTACGGCCATGGGGTTGACTTGAACCACAT : 560
FWD : TTAAGACAGACCATAAGAAAGGGCCTGGCTTCACTAAAGCTTACGGCCATGGGGTTGACTTGAACCACAT : 560

PTG2 : TTATGGAGAGACTCTGGAGAGGCAACTTAAATTGAGACTTCGCAAGGATGGAAAACTAAAGTACCAGATG : 630
FWD : TTATGGTGAGACTCTGGAGAGGCAACTTAAATTGAGACTTCGCAAGGATGGAAAACTAAAGTACCAGATG : 630

PTG2 : ATTGATGGAGAAATGTATCCACCAACAGTGAAGGACACTCAAGCAGAGATGATCTACCCTCCTCATGTTC : 700
FWD : ATTGATGGAGAAATGTATCCACCAACAGTGAAGGACACTCAAGCAGAGATGATCTACCCTCCTCATGTTC : 700

PTG2 : CTGAGCATCTGCAGTTTTCTGTTGGGCAGGAGGTGTTTGGCTTGGTCCCAGGCCTAATGATGTATGCAAC : 770
FWD : CTGAGCATCTGCAGTTTTCTGTTGGGCAGGAGGTGTTTGGCTTGGTCCCAGGCCTAATGATGTATGCAAC : 770

PTG2 : GATATGGCTGAGAGAACACAATAGAGTCTGTGACGTCTTGAAACAGGAGCATCCAGAGTGGGATGATGAG : 840
REV : GATATGGCTGAGAGAACACAATAGAGTCTGTGACGTCTTGAAACAGGAGCATCCAGAGTGGGATGATGAG : 178

PTG2 : CAGCTATTTCAGACTACAAGACTCATATTGATAGGAGAAACGATCAAGATAGTTATTGAAGATTATGTGC : 910
REV : CAGCTATTTCAGACTACAAGACTCATATTGATAGGAGAAACGATCAAGATAGTTATTGAAGATTATGTGC : 248

PTG2 : AGCACTTAAGTGGCTACCATTTCAAATTGAAGTTTGACCCTGAGCTTCTGTTCAACCAGCGTTTTCAATA : 980
REV : AGCACTTAAGTGGCTACCATTTCAAATTGAAGTTTGACCCTGAGCTTCTGTTCAACCAGCGTTTTCAATA : 318

PTG2 : CCAGAACCGAATCGCAGCTGAATTCAATACTCTGTACCACTGGCACCCCCTTCTGCCTGACACTTTTCAG : 1050
REV : CCAGAACCGAATCGCAGCTGAATTCAATACTCTGTACCACTGGCACCCCCTTCTGCCTGACACTTTTCAG : 388

PTG2 : ATACATAACCAGGAGTACACATTCCAGCAGTTCCTCTACAACAACTCCATAATGCTGGAACATGGCCTTT : 1120
REV : ATACATAACCAGGAGTACACATTCCAGCAGTTCCTCTACAACAACTCCATAATGCTGGAACATGGCCTTT : 458

PTG2 : CCCATATGGTGAAATCTTTTTCCAAGCAAAGTGCTGGTAGGGTTGCTGGTGGGAAAAATGTTCCAGCTGC : 1190
REV : CCCATATGGTGAAATCTTTTTCCAAGCAAAGTGCTGGTAGGGTTGCTGGTGGGAAAAATGTTCCAGCTGC : 528

PTG2 : AGTACAGAAAGTAGCAAAGGCTTCAATTGACCAAAGCAGACAAATGAGATACCAGTCTTTGAATGAGTAC : 1260
REV : AGTACAGAAAGTAGCAAAGGCTTCAATTGACCAAAGCAGACAAATGAGATACCAGTCTTTGAATGAGTAC : 598

PTG2 : AGGAAACGCTTCATGTTGAAACCATTCAAATCATTTGAAGAACTTACAGGAGAAAAAGAAATGGCTGCTG : 1330
REV : AGGAAACGCTTCATGTTGAAACCATTCAAATCATTTGAAGAACTTACAGGAGAAAAAGAAATGGCTGCTG : 668

PTG2 : AACTAGAAGAGCTTTATGGAGACATAGATGCTATGGAACTGTATCCAGG : 1379
REV : AACTAGAAGAGCTTTATGGGGACATAGATGCTATGGAACTGTATCCAGG : 717

RASD1
RASD1 : ATGAAACTGGCAGCGATGATCAAGAAGATGTGTCCCAGCGAGGCTGAGCTGAGCATCCCCGCCAAGAACT : 70
FWD : ATGAAACTGGCAGCGATGATCAAGAAGATGTGTCCCAGCGAGGCTGAGCTGAGCATCCCCGCCAAGAACT : 70
REV : ...................................................................... : -

RASD1 : GCTACCGCATGGTCATCCTGGGCTCCTCCAAGGTGGGCAAGACGGCCATCGTCTCGCGCTTCCTCACCGG : 140
FWD : GCTACCGCATGGTCATCCTGGGCTCCTCCAAGGTGGGCAAGACGGCCATCGTCTCGCGCTTCCTCACCGG : 140
REV : ....................................................................GG : 2

RASD1 : CCGCTTCGAGGAGCAGTACACGCCCACCATCGAGGACTTCCACCGCAAGTTCTACAGCATCCGCGGTGAG : 210
FWD : CCGCTTTGAGGAGCAGTACACGCCCACCATCGAGGACTTCCACCGCAAGTTCTACAGCATCCGCGGTGAG : 210
REV : CCGCTTTGAGGAGCAGTACACGCCCACCATCGAGGACTTCCACCGCAAGTTCTACAGCATCCGCGGTGAG : 72

RASD1 : GTCTACCAGCTCGACATCCTGGACACGTCGGGCAACCACCCCTTCCCCGCCATGCGGCGCCTGTCCATCC : 280
FWD : GTCTGCCAGCTCGACATCCTGGACACGTCGGGCAACCACCCCTTCCCCGCCATGCGGCGCCTGTCCATCC : 280
REV : GTCTGCCAGCTCGACATCCTGGACACGTCGGGCAACCACCCCTTCCCCGCCATGCGGCGCCTGTCCATCC : 142

RASD1 : TCACAGGTGACGTTTTCATCCTCGTGTTCAGCCTGGACAACCGGGACTCCTTCGAGGAGGTGCAGCGCCT : 350
FWD : TCACAGGTGACGTTTTCATCCTCGTGTTCAGCCTGGACAACCGGGACTCCTTCGAGGAGGTGCAGCGCCT : 350
REV : TCACAGGTGACGTTTTCATCCTCGTGTTCAGCCTGGACAACCGGGACTCCTTCGAGGAGGTGCAGCGCCT : 212

RASD1 : GAAGCAGCAGATCCTGGAGACCAAGTCGTGCCTGAAGAACAAAACCAAGGAGAACATCGAGGTGCCGCTG : 420
FWD : GAAGCAGCAGATCCTGGAGACCAAGTCGTGCCTGAAGAACAAAACCAAGGAGAACATCGAGGTGCCGCTG : 420
REV : GAAGCAGCAGATCCTGGAGACCAAGTCGTGCCTGAAGAACAAAACCAAGGAGAACATCGAGGTGCCGCTG : 282

RASD1 : GTCATCTGCGGCAACAAGGGCGACCGGGACTTTTACCGGGAGGTGCAGCCCCGAGAGATCGAGCAGCTGG : 490
FWD : GTCATCTGCGGCAACAAGGGCGACCGGGACTTTTACCGGGAGGTGCAGCCCCGAGAGATCGAGCAGCTGG : 490
REV : GTCATCTGCGGCAACAAGGGCGACCGGGACTTTTACCGGGAGGTGCAGCCCCGAGAGATCGAGCAGCTGG : 352

RASD1 : TGGGCGCAGACCCCAAGAAATGCGCCTACTTCGAGATCTCGGCCAAGAAGAACAGCAGCCTGGATCAGAT : 560
FWD : TGGGCGCAGACCCCAAGAAATGCGCCTACTTCGAGATCTCGGCCAAGAAGAACAGCAGCCTGGATCAGAT : 560
REV : TGGGCGCAGACCCCAAGAAATGCGCCTACTTCGAGATCTCGGCCAAGAAGAACAGCAGCCTGGATCAGAT : 422

RASD1 : GTTCCAGGCGCTCTTCGCCATGGCCAAACTGCCCAGCGAGATGAGCCCCGACCTGCACCGCAAGGTCTCG : 630
FWD : GTTCCAGGCGCTCTTCGCCATGGCCAAACTGCCCAGCGAGATGAGCCCCGACCTGCACCGCAAGGTCTCG : 630
REV : GTTCCAGGCGCTCTTCGCCATGGCCAAACTGCCCAGCGAGATGAGCCCCGACCTGCACCGCAAGGTCTCG : 492

RASD1 : GTCCAGTACTGCGACATCCTGCACAAGAAGGCTCTGAAAGGCAAAAAGCTGCTCAAAGAGGGCGGCCGGG : 700
FWD : GTCCAGTACTGCGACATCCTGCACAAGAAGGCTCTGAAAGGCAAAAAGCTGCTCAAAGAGGGCGGCCGGG : 700
REV : GTCCAGTACTGCGACATCCTGCACAAGAAGGCTCTGAAAGGCAAAAAGCTGCTCAAAGAGGGCGGCCGGG : 562

RASD1 : GCGGCACGGAGGAGGCGTACGGCGTCGTGGCTCCCTTCGCCCGCCGGCCCAGCGTCCACAGCGACCTCAT : 770
FWD : GCGGCACGGAGGAGGCGTACGGCGTCGTGGCTCCCTTCGCCCGCCGGCCCAGCGT............... : 755
REV : GCGGCACGGAGGAGGCGTACGGCGTCGTGGCTCCCTTCGCCCGCCGGCCCAGCGTCCACAGCGACCTCAT : 632

RASD1 : GTACATCCGTGAGAAAGCCATCGGCGGAGGGCACGGCAAGGAGAAGGACCGCTGTGTGATCAGCTAG : 837
FWD : ................................................................... : -
REV : GTACATCCGTGAGAAAGCCATCGGCGGAGGGCACGGCAAGGAGAAGGACCGCTGTGTGATCAGCTAG : 699

SAAL1
SAAL1 : ATGGATCGCAACCCCTCGCCGCCCTCCAGCGAGGCGGAGGAGGAGAGCGACGCGGTGGGCAGCACGGTGT : 70
FWD : ATGGATCGCAACCCCTCGCCGCCCTCCAGCGAGGCGGAGGAGGAGAGCGACGCGGTGGGCAGCACGGTGT : 70

SAAL1 : ACAGCAAGCACTGGCTCTTCAGCATCCTCACGCGCCTCATCGAGCTCATCAGCCCCGAGAAGTCCGAGCC : 140
FWD : ACAGCAAGCATTGGCTCTTCAGCATCCTTACGCGCCTCATCGAGCTCATCAGCCCCGAGAAGTCCGAGCC : 140

SAAL1 : CAGCGGGAACCCCGAGGGGATCCAGACGGACCTGGACGAAGAGATGGAGAACGACATCTGCAAGGTGTGG : 210
FWD : CAGCGGGAACCCCGAGGGGATCCAGACGGAGCTGGACGAAGAGATGGAGAACGACATCTGCAAGGTGTGG : 210

SAAL1 : GACATGTCCATGGATGAGGATGTTGCTTTGTTTCTTCAGGAGTTCAATGCCCCTGATATATTCATGGGAG : 280
FWD : GACATGTCCATGGATGAGGATGTTGCTTTGTTTCTTCAGGAGTTCAATGCCCCTGATATATTCATGGGAG : 280

SAAL1 : TTTTTGCCAAATCCAAGTGTCCTCGTCTGACTGAGATCTGTGTGGGAATACTGGGAAATATGGCCTGTTT : 350
FWD : TTTTTGCCAAATCCAAGTGTCCTCGTCTGACCGAGATCTGTGTGGGAATACTGGGAAATATGGCCTGTTT : 350

SAAL1 : CCAAGACATTTGCCTGTCCATTAGTAAAGATGAGAATCTCGGCCAAGTGTTACTGCAGCGTTTGTGTGAT : 420
FWD : CCAAGACATTTGCCTGTCCATTAGTAAAGATGAGAATCTCGGCCAAGTGTTACTGCAGCGTTTGTGTGAT : 420

SAAL1 : TCGGACTCTCCAACTCTCCTGGAAACAAGCAGGTTGTTGCTGACTTGCCTCTCCCAACCTGAGGTGGCCA : 490
FWD : TCGGACTCTCCAACTCTCCTGGAAACAAGCAGGTTGTTGCTGACTTGCCTCTCCCAACCTGAGGTGGCCA : 490

SAAL1 : ACATTTGGGTTGAGAGGATCCGAGACAGCCCTTCAGTGTATGACTGTCTTTGCTTTATCATGTCAAGCTC : 560
FWD : ACATTTGGGTTGAGAGGATCCGAGACAGCCCTTCAGTGTATGACTGTGTTTGCTTTATCATGTCAAGCTC : 560

SAAL1 : TACAAATGTTGAATTGCTGGTAAAAGTGGGTGAAGTGGTGGACAAACTCTTTGATCTGGATGAAGAGCTG : 630
FWD : TACAAATGTTGAATTGCTGGTAAAAGTGGGTGAAGTGGTGGACAAACTCTTTGATCTGGATGAAGAGCTG : 630

SAAL1 : ATGTTAAACTGGATTAAAAGTGGCACTTGTCAGTCTGTGGGACCCTCTACAGATGACTCCCCTGAAGAGC : 700
FWD : ATGTTAAACTGGATTAAAAGTGGCACTTGTCAGTCTGTGGGACCCTCTACAGATGACTCCCCTGAAGAGC : 700

SAAL1 : TTCCAGATTTTAAAATTGTGCCTTGTATACTTGAAGCAGCCAAACAAGTCCGCTCAGATAGTCCAGAAGG : 770
REV : TTCCAGATTTTAAAATTGTGCCTTGTATACTTGAAGCAGCCAAACAAGTCCGCTCAGATAGTCCAGAAGG : 120

SAAL1 : ACTTGATGTTTATATGCATATCTTGCAGCTCCTCACTACGGTGGACGAGGGCATACAGGCTATTGTGCAG : 840
REV : ACTTGATGTTTATATGCATATCTTGCAGCTCCTCACTACGGTGGACGAGGGCATACAGGCTATTGTGCAG : 190

SAAL1 : GCTCCTGATGGAGGAAAAGAGACCTGGGCTTTACTTTATGACCTGATTTGCCATGAGCTCTGCCAGCCGG : 910
REV : GCTCCTGATGGAGGAAAAGAGACCTGGGCTTTACTTTATGACCTGATTTGCCATGAGCTCTGCCAGCCGG : 260

SAAL1 : ATGATCCACCAATCATTGTGCAGGAGCAGAAGACGGTATTGGCCTCTATTTTGTCCGTGCTGTCTGCCAT : 980
REV : ATGATCCACCAATCATTGTGCAGGAGCAGAAGACGGTATTGGCCTCTATTTTGTCCGTGCTGTCTGCCAT : 330

SAAL1 : ATTTGCTTCACAGACAGAACAAGAATACATCAAGATGAGGAAAAATATGCCTCTGATTGGGAGCTTGATT : 1050
REV : ATTTGCTTCACAGACAGAACAAGAATACATCAAGATGAGGAAAAATATGCCTCTGATTGGGAGCTTGATT : 400

SAAL1 : CGTATCTTACAATACATGGAGGGCTGTGGGAAGAGATCTGTTGATAACTCAAAGGAATCTGAACAAGAAG : 1120
REV : CGTATCTTACAATACATGGAGGGCTGTGGGAAGAGATCTGTTGATAACTCAAAGGAATCTGAACAAGAAG : 470

SAAL1 : AAAGCGGAAAGGCCGAACTAAAGGAGGAAGATTTTCATTTGAAAATTTTGAAGGATATTTGTTGTGAATT : 1190
REV : AAAGCGGAAAGGCCGAACTGAAGGAGGAAGATTTTCATTTGAAAATTTTGAAGGATATTTGTTGTGAATT : 540

SAAL1 : ACTTTCCAATATGTTTCAAGAACTGACCAAGGAAAATACATTAGAAGGACTAAACCAGGGACATCTAAAT : 1260
REV : ACTTTCCAATATGTTTCAAGAACTGACCAAGGAAAATACATTAGAAGGACTAAACCAGGGACATCTAAAT : 610

SAAL1 : GAACAGACGTGTTCCTGCGCGTTCCAGAACCTCCTGCCCCTGTATTTCACATCAGTGGAGAGTTTCCTTG : 1330
REV : GAACAGACGTGTTCCTGCGCGTTCCAGAACCTCCTGCCCCTGTATTTCACATCAGTGGAGAGTTTCCTTG : 680

SAAL1 : AAGTTCTGCGCGAGGCTGATGGAACACTTGCTGAGAATCTTGAAAAACGTTTCCCGAGCCTGAAGGTCCC : 1400
REV : AAGTTCTGCGTGAGGCTGATGGAACACTTGCTGAGAATCTTGAAAAACGTTTCCCGAGCCTGAAGGTCCC : 750

SAAL1 : TACCTAA : 1407
REV : TACCTAA : 757

SDC4
REV : ATGCCGCTGCCCCGCGCCGCGTTCCTGCTCGGCCTCCTGCTGGCCGCTGCCGCCGCCGAGTCGGTGAGAG : 70
SDC4 : ATGCCGCTGCCCCGCGCCGCGTTCCTGCTCGGCCTCCTGCTGGCCGCTGCCGCCGCCGAGTCGGTGAGAG : 70
FWD : ATGCCGCTGCCCCGCGCCGCGTTCCTGCTCGGCCTCCTGCTGGCCGCTGCCGCCGCCGAGTCGGTGAGAG : 70

REV : AAACAGAGACCATGGATGCCCGATGGCTTGACAACATGGGCTCTGGAGACCTGCCAGATGATGAAGACAT : 140
SDC4 : AAACAGAGACCATGGATGCCCGATGGCTTGACAACGTGGGCTCTGGAGACCTGCCAGATGATGAAGACAT : 140
FWD : AAACAGAGACCATGGATGCCCGATGGCTTGACAACATGGGCTCTGGAGACCTGCCAGATGATGAAGACAT : 140

REV : TGGTGAATTCACACCTCACTTAACTTCTGACGAGTTTGATATAGATGACACATCTGGCTCCGGAGACTAC : 210
SDC4 : TGGTGAATTCACACCTCACTTAACTTCTGACGAGTTTGATATAGATGACACATCTGGCTCCGGAGACTAC : 210
FWD : TGGTGAATTCACACCTCACTTAACTTCTGACGAGTTTGATATAGATGACACATCTGGCTCCGGAGACTAC : 210

REV : TCAGACTATGTTGATGCCATATACCTGACCACTGTGGATACTCCTGCAATATCTGACAACTATATCCCTG : 280
SDC4 : TCAGATTATGATGATGCCATATACCTGACCACTGTGGATACTCCTGCAATATCTGACAACTATATCCCTG : 280
FWD : TCAGACTATGTTGATGCCATATACCTGACCACTGTGGATACTCCTGCAATATCTGACAACTATATCCCTG : 280

REV : GAGATACAGAGAGAAAGATGGAAGGTGAGAAGAAAAACACGATGCTGGACAATGAAATCATTCCAGACAA : 350
SDC4 : GAGATACAGAGAGAAAGATGGAAGGTGAGAAGAAAAACACCATGCTGGACAATGAAATCATTCCAGACAA : 350
FWD : GAGATACAGAGAGAAAGATGGAAGGTGAGAAGAAAAACACGATGCTGGACAATGAAATCATTCCAGACAA : 350

REV : AGCTTCACCTGTTGAAGCAAACCTGTCCAACAAGATCTCCATGGCAAGCACAGCCAACAGCAGCATCTTT : 420
SDC4 : AGCTTCACCTGTTGAAGCAAACCTGTCCAACAAGATCTCCATGGCAAGCACAGCCAACAGCAGCATCTTT : 420
FWD : AGCTTCACCTGTTGAAGCAAACCTGTCCAACAAGATCTCCATGGCAAGCACAGCCAACAGCAGCATCTTT : 420

REV : GAAAGAACAGAAGTTCTTACAGCTCTCATTGCAGGAGGAGCAGTTGGCCTCCTGTTTGCTGTCTTCCTGA : 490
SDC4 : GAAAGAACAGAAGTTCTTACAGCTCTCATTGCAGGAGGAGCAGTTGGCCTCCTGTTTGCTGTCTTCCTGA : 490
FWD : GAAAGAACAGAAGTTCTTACAGCTCTCATTGCAGGAGGAGCAGTTGGCCTCCTGTTTGCTGTCTTCCTGA : 490

REV : TCCTCCTCTTAGTCTATCGCATGAAGAAAAAGGACGAGGGCAGCTACGACCTTGG : 545
SDC4 : TCCTCCTCTTAGTCTATCGCATGAAGAAAAAGGACGAGGGCAGCTACGACCTTGG : 545
FWD : TCCTCCTCTTAGTCTATCGCATGAAGAAAAAGGACGAGGGCAGCTACGACCTTGG : 545

SELE
SELE : ATGGTGAATTGTTGGACATACCATTATTCAGACACAAATATGACCTACAAAGAGGCAGAGTTGTGGTGCA : 70
FWD : ATGGTGAATTGTTGGACATACCATTATTCAGACACAAACATGACCTACGAAGAGGCAGAGTTGTGGTGCA : 70

SELE : AAAAGAGGTATACTAACATGGTTGCCATTCAAAACAAGGATGAAATCAACCATCTCAATGACTTTTTACC : 140
FWD : AAAAGAGGTATACTAACATGGTTGCCATTCAAAACAAGGATGAAATCAACCATCTCAATGACTTCTTACC : 140

SELE : CTTCAATCCGAGTTACTACTGGATTGGAATCAGAAAAATTAATGGTACATGGACCTGGGTTGGAACAAAC : 210
FWD : CTTCAATCCGAGTTACTACTGGATTGGAATCAGAAAAATTAATGGTACATGGACCTGGGTTGGAACAAAC : 210

SELE : AAGGAGCTGACAAAAGAAGCAGAAAACTGGGCTTCAGGTGAACCAAATGGCAAAGGGAACAATGAGGACT : 280
FWD : AAGGAGCTGACAGAAGAAGCAGAAAACTGGGCTTCAGGTGAACCAAATGGCAAAGGGAACAATGAGGACT : 280

SELE : GTGTTGAAATCTACATCAAAAGAGGGAAGGATGACGGCAAATGGAATGATGAGAAGTGTGAGAAGAAGAA : 350
FWD : GTGTTGAAATCTACATCAAAAGAGGGAAGGATGACGGCAAATGGAATGATGAGAAGTGTGAGAAGAAAAA : 350

SELE : GGTTGCCTTGTGCTATACAGCTTCTTGCAACTCATCTCTCTGCAGTGGCCGTGGAAAATGCATAGAGACT : 420
FWD : GGTTGCCTTGTGCTATACAGCTTCTTGCAACTCATCTCTCTGCAGTGGCCGTGGAGAATGCATAGAGACT : 420

SELE : ATTAACAATTACACCTGCCATTGTAACCCTGGATTCTATGGGCCGGGTTGTGAATTTGTTGAGACTTGTG : 490
FWD : ATTAACAATTACACCTGCCATTGTAACCCTGGATTCTATGGGCCGGGTTGTGAATTTGTTGAGACTTGTG : 490

SELE : ATCCACTTAAAAAACCTGATCACGGAAGCCTTGAGTGCAACCATCCGTTGAAGGACTTCAGCTACAACTC : 560
FWD : ATCCACTGAAGAAACCTGATCACGGAAGCCTTGAGTGCAACCATCCGTTGAAGGACTTCAGCTACAACTC : 560

SELE : GTCATGCACAGCTCAATGTGAAGAAGGCTATGAGCTGAATGGATTGGAGTCAGTATATTGTACGTCTCAT : 630
FWD : GTCATGCACAGCTCAATGTGAAGAAGGCTATGAGCTGAATGGATTGGAGTCAGTATATTGTACGTCTCAT : 630

SELE : GGAAAATGGTCTGGCCCACTTGCATCATGCACAGCTGTGAGGTGTGATGCTGTAACCTGGCCAGAAGAAG : 700
FWD : GGAAACTGGTCTGGCCCACTTGCATCATGCACAGCTGTGAGGTGTGATGCTGTAACCTGGCCAGAAGAAG : 700

SELE : GTTCTGTGAACTGTTCTTATGCACATCCCACCTATGGCTCACGTTGTGATTTCCGTTGCCGAGAAGGCTA : 770
FWD : GTTCTGTGAACTGTTCTTATGCAGATCCCACCTATGGCTCACGCTGTGATTTCCGT.............. : 756

SELE : GTTCTGTGAACTGTTCTTATGCACATCCCACCTATGGCTCACGTTGTGATTTCCGTTGCCGAGAAGGCTA : 770
REV : ................TTATGCAGATCCCACCTATGGCTCACGCTGTGATTTCCGTTGCCGAGAAGGCTA : 54

SELE : TGTTCTGGAGGGCCCATCCAGCACTGAGTGCATGGCACAAGGACAGTGGTCAGAGCCATTCCCAAAATGC : 840
REV : TGTCCTGGAGGGCCCATCCAGCACTGAGTGCATGGCACAAGGACAGTGGTCAGAGCCATTCCCAAAATGC : 124

SELE : AAAGCTGTGACCTGCACTGTCTTAAAAGCACCTGCTAATGGCTCTCTGAACTGCTCCCGTTCCTCTGAGT : 910
REV : AAAGCTGTGACCTGCACTGTCTTAAAAGCACCTGCTAATGGCTCTCTGAACTGCTCCCGTTCCTCTGAGT : 194

SELE : TTACATGGAATACCACCTGTGAGTTCACTTGTGAGGAAGGATTTGCTCTGACAGGACAATCCACGCTGCA : 980
REV : TTACATGGAATACCACCTGTGAGTTCACTTGTGAGGAAGGATTTGCTCTGACAGGACAATCCACGCTGCA : 264

SELE : CTGTGGCTCTTCTGGGGCCTGGGACAAGCAGCAGCCATCCTGTGCAGCTGTGAGGTGTGATGCTGTAACC : 1050
REV : CTGTGGCTCTTCTGGGGCCTGGGACAAGCAGCAGCCATCCTGTGCAGCTGTGAGGTGTGATGCTGTAACC : 334

SELE : TGGCCAGAAGAAGGTTCTGTGAGCTGTTCTTATGCAGATCCCACCTATGGCTCACGTTGTGATTTCCGTT : 1120
REV : TGGCCAGAAGAAGGTTCTGTGAGCTGTTCTTATGCAGATCCCACCTATGGCTCACGTTGTGATTTCCGTT : 404

SELE : GCCGAGAAGGCTATGTCCTGGAGGGCCCATCCAGCACTGAGTGCATGGCACAGGGACAGTGGTCAGAGCC : 1190
REV : GCCGAGAAGGCTATGTTCTGGAGGGCCCATCCAGCACTGAGTGCATGGCACAGGGACAGTGGTCAGAGCC : 474

SELE : ATTCCCAAAATGCAAAGTTGTACAGTGTGAACCGCTGAAATCTCCTGAGAAAGGCTCTATGGATTGCATA : 1260
REV : ATTCCCAAAATGCAAAGTTGTACAGTGTGAACCGCTGAAGTCTCCTGAGAAAGGCTCTATGGATTGCATA : 544

SELE : CATGGTGCTGGGAACTTCACATATAACACGGCCTGCCACTTCAGCTGCCTAGAAGGATGGAATCTCAATG : 1330
REV : CATGGTGCTGGGAACTTCACATATAACACGGCCTGCCACTTCAGCTGCCTAGAAGGATGGAATCTCAATG : 614

SELE : GATCTCGTGTTCTTGAGTGCAGTCATTCAGGAAACTGGAGTGCCAGTCTGCCCACATGTGAAGCTTCTCA : 1400
REV : GATCTCGTGTTCTTGAGTGCAGTCATTCAGGAAACTGGAGTGCCAGTCTGCCCACATGTGAAGCTTCTCA : 684

SELE : ACAAGTTGCCTATGTCACTGTAGGCATAGCAGCCACCACTACCTCTCTGCTTGCCACAGCATCATTCCTC : 1470
REV : ACAAGTTGCCTATGTCACTGTGGGCATAGCAGCCACCACTACCTCTCTGCTTGCCACAGCATCATTCCTC : 754

SELE : TTTTGGCTTGCAAAACGGTTACGGGGAAAAGCAAAGAAATTCACTCCTGCCAGCAGCTGTCAGCACCTCA : 1540
REV : TTTTGGCTTGCAAAACGGTTACGGGGAAAAGCAAAGAAATTCACTCCTGCCAGCAGCTGTCAGCACCTCA : 824

SELE : CTACTGAATGCCAGAATGTCTAA : 1563
REV : CTACTGAATGCC.GAATGTCTAA : 846

SERPINE2
SERPINE2 : ATGAACTGGCACTTCTCGCTTCTCTTTCTTCTTGGGACTTTAGCATCTGTGTGTTCCCAGTTCAATTTTT : 70
FWD : ATGAACTGGCACTTCTCGCTTCTCTTTCTTCTTGGGACTTTAGCATCTGTGTGTTCCCAGTTCAATTTTT : 70

SERPINE2 : ATCCTCTGGAGGAGCTTAGCTCTGATGTTGGAATCCAAGTCTTCAATCAGATAGTGAAAGCTAAGCCTCA : 140
FWD : ATCCTCTGGAGGAGCTTAGCTCTGATGTTGGAATCCAAGTCTTCAATCAGATAGTGAAAGCTAAGCCTCA : 140

SERPINE2 : GGACAATGTAGTAGTTTCTCCTCATGGGATTGCATCAGTGTTGGGGGTGTTACAGCTGGGTGCTGATGGC : 210
FWD : GGACAATGTAGTAGTTTCTCCTCATGGGATTGCATCAGTGTTGGGGGTGTTACAGCTGGGTGCTGATGGC : 210

SERPINE2 : AAGACAAAGAAGCAGCTGACAACTATGATGAGATATAGTGTAAATGGAGCAGAAAACCCTCCAAGAGCAG : 280
FWD : AAGACAAAGAAGCAGCTGACAACTATGATGAGATATAGTGTAAATGGAGCAGAAAACCCTCCAAGAG... : 277

SERPINE2 : CTGGAAGCAAGGAGGAGAGCCAGCATCATGAGACCAGCCAGCTCTCCCCGGACCACCAGCAAGTGCTCCG : 350
FWD : ...................................................................... : -

SERPINE2 : TGGACCACCAGTGGTCCACAGAATACAGTTTGAGAACCTCTGTTATGGAGTTGGTAAAGCGTTAAAGAAG : 420
FWD : ...............................................GAGTTGGTAAAGCGTTAAAGAAG : 300

SERPINE2 : ATAAACAGGCTCATAGTCTCAAAAAAGAATAAAGACATTGTTACAATTGCTAATGCAGTATTTGCAAAGA : 490
FWD : ATAAACAGGCTCATAGTCTCAAAAAAGAATAAAGACATTGTTACAATTGCTAATGCAGTATTTGCAAAGA : 370

SERPINE2 : GTGGCTTTAAAATGGAAGTGCCTTTTGTTACAAGGAACAAAGAGGTGTTTCAGTGCAGTGTCAAGAGCGT : 560
FWD : GTGGCTTTAAAATGGAAGTGCCTTTTGTTACAAGGAACAAAGAGGTGTTTCAGTGCAGTGTCAAGAGCGT : 440

SERPINE2 : GGACTTTGAGGACCCAAATACAGCCTGTGATTCCATCAACCAGTGGGTGAAAAATGAAACAAGGGGTATG : 630
FWD : GGACTTTGAGGACCCAAATACAGCCTGTGATTCCATCAACCAGTGGGTGAAAAATGAAACAAGGGGTATG : 510

SERPINE2 : ATTGATCAAGTTGTAGCTCCAGATGATATTGACAGTTTAACCAGACTGGTTCTAGTAAATGCTGTGTATT : 700
FWD : ATTGATCAAGTTGTAGCTCCAGATGATATTGACAGTTTAACCAGACTGGTTCTAGTAAATGCTGTGTATT : 580

SERPINE2 : TCAA.GGGCTTGTGGAAA.TCACGATTTCGACCTGAAAACACAAAGAAACGGCCATTTTATGGAGCTGAT : 768
REV : TCAAAGGGCTTGTGGAAAATCACGATTTCGACCTGAAAACACAAAGAAACGGCCATTTTATGGAGCTGAT : 136

SERPINE2 : GGGAAGACCTACCAAGTTCCCATGTTGTCCCAGTTATCAATCTTCCGCTGTGGTACTACAAGTACTCCAA : 838
REV : GGGAAGACCTACCAAGTTCCCATGTTGTCCCAGTTATCAATCTTCCGCTGTGGTACTACAAGTACTCCAA : 206

SERPINE2 : ATGAGCTGTGGTATAACATAATTGAATTGCCATACCATGGAGAAATGATAAGCATGTTGATTGCTTTGCC : 908
REV : ATGAGCTTTGGTATAACATAATTGAATTGCCATACCATGGAGAAATGATAAGCATGTTGATTGCTTTGCC : 276

SERPINE2 : TACGGAGAACACAACACCGCTCTCTGCTATCATTCCACACATCAGCACAAAGACAATAGGAAGCTGGATG : 978
REV : TACGGAGAACACAACACCGCTCTCTGCTATCATTCCATACATCAGCACAAAGACAATAGGAAGCTGGATG : 346

SERPINE2 : ACAACCATGGTAGCCAAAAGAGTGCAGGTTATTTTGCCCAAATTTACAGCAGTAGCAGAAACAGACTTAA : 1048
REV : ACAACCATGGTAGCCAAAAGAGTGCAGGTTATTTTGCCCAAATTTACAGCAGTAGCAGAAACAGACTTAA : 416

SERPINE2 : AGGACCCTCTGAAAGCCCTTGGTATTACAGATATGTTTGATGAGTCAAAATCAAACTTTGCAAAAATAAC : 1118
REV : AGGACCCTCTGAAAGCCCTTGGTATTACAGATATGTTTGATGAGTCAAAATCAAACTTTGCAAAAATAAC : 486

SERPINE2 : AAGAACAGAAGGTCTACACGTATCTCATGTTTTGCAGAAGACGAAAATTGAAGTTAGTGAAGATGGAACC : 1188
REV : AAGAACAGAAGGTCTACACGTATCTCATGTTTTGCAGAAGACGAAAATTGAAGTTAGTGAAGATGGAACC : 556

SERPINE2 : AAAGCTTCTGCAGCAACAACTGCGATTTTAATAGCCAGATCATCCCCTCCTTGGTTCATAGTAGACAGAC : 1258
REV : AAAGCTTCTGCAGCAACAACTGCGATTTTAATAGCCAGATCATCCCCTCCTTGGTTCATAGTAGACAGAC : 626

SERPINE2 : CATTTGTATTCTTCATCCGGCACAATCCTACAGGTACAATCTTGTTTATGGGACAAATAAACAAACCTTG : 1328
REV : CATTTGTATTCTTCATCCGGCACAATCCTACAGGTACAATCTTGTTTATGGGACAAATAAACAAACCTTG : 696

SERPINE2 : A : 1329
REV : A : 697

SLCOGA1
SLCOGA1 : ATGAAGGGCAACGACGGTATCGAGAACCCGGCGTTCGAGCCCCCTACCCCTGTCTTCGGCGGTCGACGCC : 70
FWD : ATGAAGGGCAACGACGGTATCGAGAACCCGGCGTTCGAGCCCCCTACCCCTGTCTTCGGCGGTCGACGCC : 70

SLCOGA1 : GTGCGAGCCCCGCCGGGGCAGGTGTCGCTGCCGAGGCCCCGCCGGTGGAGGAGGCCGAGGAGGGGCCCTG : 140
FWD : GTGCGAGCCCCGCCGGGGCAGGTGTCGCTGCCGAGGCCCCGCCGGTGGAGGAGGCCGAGGAGGGGCCCTG : 140

SLCOGA1 : CGGCTGGGGCCGCTGCACCCCTAAGGTTCTGCAGCTCTGCAACACCCCGGAGGGCTACCTGGCTGCCTAC : 210
FWD : CGGCTGGGGCCGCTGCACCCCTAAGGTTCTGCAGCTCTGCAACACCCCGGAGGGCTACCTGGCTGCCTAC : 210

SLCOGA1 : AGCCTGCTGGCCATCTTCCAAGGCATTGTGGTAAATGGCCTAATTAACATCAGTATATCAACAATTGAGA : 280
FWD : AGCCTGCTGGCCATCTTCCAAGGCATTGTGGTAAATGGCCTAATTAACATCAGTATATCAACAATTGAGA : 280

SLCOGA1 : AGCGCTATGAGTTGAACAGCTCCCTTACTGGCTTGATATCTGCAAGCTATGACATTGCTTTTTGTGTATT : 350
FWD : AGCGCTATGAGTTGAACAGCTCCCTTACTGGCTTGATATCTGCAAGCTATGACATTGCTTTTTGTGTATT : 350

SLCOGA1 : GTCATTGTTTGTATCTTTCTTTGGAGAAAGAGGGCACAAACCACGATGGCTGGCTTTCTCAGCTTTTATG : 420
FWD : GTCATTGTTTGTATCTTTCTTTGGAGAAAGAGGGCACAAACCACGATGGCTGGCTTTCTCAGCTTTTATG : 420

SLCOGA1 : CTAGGATTGGGCTCACTTGTATTTTCCTTACCACACTTCAGTAGTGGAAAATATCACTATGGAGCTAAAC : 490
FWD : CTAGGATTGGGCTCACTTGTATTTTCCTTACCACACTTCAGTAGTGGAAAATATCACTATGGAGCTAAAC : 490

SLCOGA1 : TTGAAGATACATGTCAAATTTCAGGAGCAAGCTCTGCTAATTTCACTTGCAGTGCCAGCACAAAGTCTTC : 560
FWD : TTGAAGATACATGTCAAATTTCAGGAGCAAGCTCTGCTAATTTCACTTGCAGTGCCAGCACAAAGTCTTC : 560

SLCOGA1 : ACTTTCCAATTATCTGTATGTCTTTATACTGGGACAGCTGCTGCTGGGAGTTGGAGGAACTCCACTATAT : 630
FWD : ACTTTCCAATTATCTGTATGTCTTTATACTGGGACAGCTGCTGCTGGGAGTTGGAGGAACTCCACTGTAT : 630

SLCOGA1 : ACTTTGGGGACAGCTTTTATTGATGATAGTGTTCCAAAGCACACATCTTCCCTTTATATAGGAATTGGCT : 700
FWD : ACTTTGGGGACAGCTTTTATTGATGATAGTGTTCCAAAGCACACATCTTCCCTTTATATAGGAATTGGCT : 700

SLCOGA1 : ATGCCATGTCATTGCTTGGGCCTGCTATTGGCTATGTCTTAGGAGGACAGCTGCTTAGTATTTACATTGA : 770
FWD : ATGCCATGTCATTGCTTGGGCCTGCTATTGGCTATGTCTTAGGAGGACAGCTGCTTAGTATTTACATTGA : 770

SLCOGA1 : TATCAAGATCCCAGAAAGTACACAGATGGACCAAGATGACCCACGCTGGCTTGGAGCATGGTGGATAGGA : 840
FWD : TATC.................................................................. : 774

SLCOGA1 : TTCCTTGCATGCTTTTTTGCAATGTGGTTTCTGATAATACCATTTTCTTGCTTTCCGAAATACTTGCCAG : 910
FWD : ...................................................................... : -

SLCOGA1 : GAACAGCAAAAATACAGGCAGAAAAAATCTCTGAAACCCATAATGATGGAAGTGAGGCACTTGTTGAAGC : 980
FWD : ...................................................................... : -

SLCOGA1 : AAAGAATATTGGAAAAAATTTCAGAGACTTTCCTATTGCTCTCCTGATTCTGTTGAAGAATCCAGTGCTT : 1050
FWD : ...................................................................... : -
SLCOGA1 : ATGAGTCTAATATTAGCCAGTACCTCAGAAGCTCTAGTTGCCACTGGCTTTGCCACATTCCTACCAAAGT : 1120
FWD : ...................................................................... : -
SLCOGA1 : TCATAGAAAATCAGTTTGGAATGACATCAAGTTTTTCAGCAACTCTTGGAGGGCTTGTATTAGTTCCAGC : 1190
FWD : ...................................................................... : -
SLCOGA1 : AGCAGCACTAGGCCAAGTCATAAGTGGCATCTTGGTATCCAAGTATAAAATGGATTGCAAAAACATGATC : 1260
FWD : ...................................................................... : -
SLCOGA1 : AGCAGCACTAGGCCAAGTCATAAGTGGCATCTTGGTATCCAAGTATAAAATGGATTGCAAAAACATGATC : 1260
REV : ................................................................ATGATC : 6
SLCOGA1 : AAGTTCATGATAGGCACTTGCTCAGTTGCCTTCCTGTTAAACACAGTCTTTTTAGTTGCTAAATGTGGAA : 1330
REV : AAGTTCATGATAGGCACTTGCTCAGTTGCCTTCCTGTTAAACACAGTCTTTTTAGTTGCTAAATGTGGAA : 76

SLCOGA1 : ATGAACCTTTTGCTGGTGTTTCTGAAACATACAATGGAACAGGCATGCTACATAACTTAACTGCACCATG : 1400
REV : ATGAACCTTTTGCTGGTGTTTCTGAAACATACAATGGGACAGGCATGCTACATAACTTAACTGCACCATG : 146

SLCOGA1 : CAATGCTAACTGCAGATGCTCCAGCTCCAGATACTACCCTGTTTGTGGCAGAGATGAAGTCCAGTACTTT : 1470
REV : CAATGCTAACTGCAGATGCTCCAGCTCCAGATACTACCCTGTTTGTGGCAGAGATGAAGTCCAGTACTTT : 216

SLCOGA1 : TCTCCATGTTTTGCAGGATGTGCATCGTATAATCTTAATAACAAGAAAAAGACTTACTACAACTGTTCCT : 1540
REV : TCTCCATGTTTTGCAGGATGTGCATCGTATAATCTTAATAACAAGAAAAAGACTTACTACGACTGTTCCT : 286

SLCOGA1 : GTATTGGGAAGCTGAAGAGAGAAAATATTTCAGAAGACTTTCTCTCTGAAGCTGTACCTGGAAAATGTCC : 1610
REV : GTATTGGGAAGCTGAAGAGAGAAAATATTTCAGAAGACTTTCTCTCTGAAGCTGTACCTGGAAAATGTCC : 356

SLCOGA1 : AACACGATGCAAACATTTACCTTTATTTCTGATCTATTTCTTTTTTGCTGTTGTTTTTACATTTATGGCT : 1680
REV : AACACGATGCAAACATTTACCTTTATTTCTGATCTATTTCTTTTTTGCTGTTGTTTTTACATTTATGGCT : 426

SLCOGA1 : GTTACTCCAACTACTGTGGCAGTTCTCAGATGTGTGCCAGATAAGCAGCGCTCATTTGCTCTTGGAGTGC : 1750
REV : GTTACTCCAACTACTGTGGCAGTTCTCAGATGTGTGCCAGATAAGCAGCGCTCATTTGCTCTTGGAGTGC : 496

SLCOGA1 : AGTCAGTGTTTCTACGACTACTAGGTACTGTTCCTGGACCAATTTTGTTTGGTTTTGCAATAGATAATAG : 1820
REV : AGTCAGTGTTTCTACGACTACTAGGTACTGTTCCTGGACCAATTTTGTTTGGTTTTGCAATAGATAATAG : 566

SLCOGA1 : TTGTTCTCTGTGGGATGTCAATGAATGTAGAACTAAAGGAGCCTGTTGGGTTTATGACAATGAAAGAATG : 1890
REV : TTGTTCTCTGTGGGATGTCAATGAATGTAGAACTAAAGGAGCCTGTTGGGTTTATGACAATGAAAGAATG : 636

SLCOGA1 : GCATATCTTTTGATGAGCATAAGCGCTGCTTGCAAAATCATCACGATCATCTTTGTGGTTGTAGCAGTGT : 1960
REV : GCATATCTTTTGATGAGCATAAGCGCTGCTTGCAAAATCATCACGATCATCTTTGTGGTTGTAGCAGTGT : 706

SLCOGA1 : TTTTGTATAAGCCTCCACAACCAAGACCAGCTCTGCCGCAAAAGGATTCAGAAATGGTATCTGCTATACA : 2030
REV : GTTTGTATAAGCCTCCACAACCAAGACCAGCTCTGCCGCAAAAGGATTCAGAAATGGTATCTGCTATACA : 776

SLCOGA1 : TGCGTAA : 2037
REV : TGCGTAA : 783

SNX10
REV : ATGACACCAAAACACGAAAAACAAGAATTTGTTACCGTCTTGGTACGAGACCCCAGAACACAGAAAGAAG : 70
SNX10 : ATGACACCAAAACACGAAAAACAAGAATTTGTTACCGTCTTGGTACGAGACCCCAGAACACAGAAAGAAG : 70
FWD : ATGACACCAAAACACGAAAAACAAGAATTTGTTACCGTCTTGGTACGAGACCCCAGAACACAGAAAGAAG : 70

REV : ACTCATGGCATTCTTACATAGACTATGAGATATTTATTCACACAAACAGTATGTGCTTCACGAGGAAAAC : 140
SNX10 : ACTCATGGCATTCTTACATAGACTACGAGATATTTATTCACACAAACAGTATGTGCTTCACGAGGAAAAC : 140
FWD : ACTCATGGCATTCTTACATAGACTATGAGATATTTATTCACACAAACAGTATGTGCTTCACGAGGAAAAC : 140

REV : TTCCTGTGTCAGACGACGCTTCCGAGAATTTGTTTGGCTTCGGCAGAGGCTTCAGAGCAATGCTGTGCTC : 210
SNX10 : TTCCTGTGTCAGACGACGCTTCCGAGAATTTGTTTGGCTTCGGCAGAGGCTTCAGAGCAATGCTGTGCTC : 210
FWD : TTCCTGTGTCAGACGACGCTTCCGAGAATTTGTTTGGCTTCGGCAGAGGCTTCAGAGCAATGCTGTGCTC : 210

REV : ATACAGTTACCTGAACTGCCATCTAAAACTCCCTTTTTCAATATGAACAATCCCCATCATGTGGACCATC : 280
SNX10 : ATACAGTTACCTGAACTGCCATCTAAAACTCCCTTTTTCAATATGAACAATCCCCATCATGTGGACCATC : 280
FWD : ATACAGTTACCTGAACTGCCATCTAAAACTCCCTTTTTCAATATGAACAATCCCCATCATGTGGACCATC : 280

REV : GTCGGCAGGGGCTGCAGGAGTTCCTGGAGAAGATTCTACAGAATGCATTATTGCTGTCAGACAGCAGGCT : 350
SNX10 : GTCGGCAGGGGCTGCAGGAGTTCCTGGAGAAGATTCTACAGAATGCATTATTGCTGTCAGACAGCAGGCT : 350
FWD : GTCGGCAGGGGCTGCAGGAGTTCCTGGAGAAGATTCTACAGAATGCATTATTGCTGTCAGACAGCAGGCT : 350

REV : TCACCTCTTCCTACAGACTCAGCTAAGTCCGGAAGATATGGAGGCTTGTGTATGTGGGCAGACCAAATAT : 420
SNX10 : TCACCTCTTCCTACAGACTCAGCTAAGTCCGGAAGATATGGAGGCTTGTGTATGTGGGCAGACCAAATAT : 420
FWD : TCACCTCTTCCTACAGACTCAGCTAAGTCCGGAAGATATGGAGGCTTGTGTATGTGGGCAGACCAAATAT : 420

REV : TCTGTCGCTGATGCGATTCAGAAGTTTGCCTCTTTGAACAGGCGCTTTCCTATAGAAGATGAAGAGAGGA : 490
SNX10 : TCTGTCGCTGATGCGATTCAGAAGTTTGCCTCTTTGAACAGGCGCTTTCCTATAGAAGATGAAGAGAGGA : 490
FWD : TCTGTCGCTGATGCGATTCAGAAGTTTGCCTCTTTGAACAGGCGCTTTCCTATAGAAGATGAAGAGAGGA : 490

REV : AAAAAGGACGAAACGATGCAGACTCTGATTCAGAGAGTTCATCCTCCGGGCTTGGACCTAGTGATGACAG : 560
SNX10 : AAAAAGGACGAAACGATGCAGACTCTGATTCAGAGAGTTCATCCTCCGGGCTTGGACCTAGTGATGACAG : 560
FWD : AAAAAGGACGAAACGATGCAGACTCTGATTCAGAGAGTTCATCCTCCGGGCTTGGACCTAGTGATGACAG : 560

REV : CATTTCATGTGGATGTAAAGCAAGCCTAGCTTCTGAAGAATCGTGA : 606
SNX10 : CATTTCATGTGGATGTAAAGCAAGCCTAGCTTCTGAAGAATCGTGA : 606
FWD : CATTTCATGTGGATGTAAAGCAAGCCTAGCTTCTGAAGAATCGTGA : 606

SOCS1
SOCS1 : ATGGTAGCGCACAGCAAGGTGTCAGCAGACAATGCAGTTGCAGCAGACCCGCGATGTCTACTTGACCCTC : 70
FWD : ..........................................................TACTTGACCCTC : 12
REV : ATGGTAGCGCACAGCAAGGTGTCAGCAGACAATGCAGTTGCAGCAGACCCGCGATGTCTACTTGACCCTC : 70

SOCS1 : CGGCACGGGATCGCTCTCAGGCTCGAGGTTACCGCGGCACAGGCCGGCCTGGCGCTGCGCAGGCACCGAG : 140
FWD : CGGCACGGGATCGCTCTCAGGCTCGAGGTTACCGCGGCACAGGCCGGCCTGGCGCTGCGCAGGCACCGAG : 82
REV : CGGCACGGGATCGCTCTCAGGCTCGAGGTTACCGCGGCACAGGCCGGCCTGGCGCTGCGCAGGCACCGAG : 140

SOCS1 : CAGCACGCACTTCCGAACCTTTCGCTCGCAAGCGGATTTCAGTAGCATCACTCGAGCAAGCAGCCTGCTG : 210
FWD : CAGCACGCACTTCCGAACCTTTCGCTCGCAAGCGGATTTCAGTAGCATCACTCGAGCAAGCAGCCTGCTG : 152
REV : CAGCACGCACTTCCGAACCTTTCGCTCGCAAGCGGATTTCAGTAGCATCACTCGAGCAAGCAGCCTGCTG : 210

SOCS1 : GATGCCTGCGGCTTCTACTGGGGACCGCTGACCGTCAGCGCCGCCCATGAGAAGCTGAAGTCTGAGCCCG : 280
FWD : GATGCCTGCGGCTTCTACTGGGGACCGCTGACCGTCAGCGCCGCCCATGAGAAGCTGAAGTCTGAGCCCG : 222
REV : GATGCCTGCGGCTTCTACTGGGGACCGCTGACCGTCAGCGCCGCCCATGAGAAGCTGAAGTCTGAGCCCG : 280

SOCS1 : AGGGCACCTTCCTCATCAGGGACAGCACGCAGAAGAATTGTTTCTTTGCCATCAGTGTTAAAACCGCGAC : 350
FWD : AGGGCACCTTCCTCATCAGGGACAGCACGCAGAAGAATTGTTTCTTTGCCATCAGTGTTAAAACCGCGAC : 292
REV : AGGGCACCTTCCTCATCAGGGACAGCACGCAGAAGAATTGTTTCTTTGCCATCAGTGTTAAAACCGCGAC : 350

SOCS1 : CGGGCCCACCAGCATCCGGATAAACTTTCAGACGGGGCGTTTCAGCCTGGATGGCAGCAAGGAGACCTTT : 420
FWD : CGGGCCCACCTGCATCCGGATAAACTTTCAGACGGGGCGTTTCAGCCTGGATGGCAGCAAGGAGACCTTT : 362
REV : CGGGCCCACCTGCATCCGGATAAACTTTCAGACGGGGCGTTTCAGCCTGGATGGCAGCAAGGAGACCTTT : 420

SOCS1 : GATTGTCTTTTTAAGCTGCTGGAACATTACTTAAGCTCCCCGAGGAAGGTATTGGTCACCCCCCTGCGCA : 490
FWD : GATTGTCTTTTTAAGCTGCTGGAACATTACTTAAGCTCCCCGAGGAAGGTATTGGTCACCCCCCTGCGCA : 432
REV : GATTGTCTTTTTAAGCTGCTGGAACATTACTTAAGCTCCCCGAGGAAGGTATTGGTCACCCCCCTGCGCA : 490

SOCS1 : AAGTCCGTGTGCAGCCCCTGCAGGAGCTCTGCCGGAAGAGCATCGTGAAGACTTTTGGAAGAGAGAACTT : 560
FWD : AAGTCCGTGTGCAGCCCCTGCAGGAGCTCTGCCGGAAGAGCATCGTGAAGACTTTTGGAAGAGAGAACTT : 502
REV : AAGTCCGTGTGCAGCCCCTGCAGGAGCTCTGCCGGAAGAGCATCGTGAAGACTTTTGGAAGAGAGAACTT : 560

SOCS1 : GAACCAGATCCCTCTCAATCCTGTTCTAAAGGACTATCTGAAATCCTTCCCGTTTCAGATCTAA : 624
FWD : GAACCAGATCCCTCTCAATCCTGTTCTAAAGGACTATCTGAAATCCTTCCCGTTCAGATCTAA. : 565
REV : GAACCAGATCCCTCTCAATCCTGTTCTAAAGGACTA............................ : 596

SOCS3
REV : ATGGTCACCCACAGCAAGTTCCCCGCCGCCGGGATGAGCCGCCCCCTGGACACCAGCCTGCGCCTCAAGA : 70
FWD : ATGGTCACCCACAGCAAGTTCCCCGCCGCCGGGATGAGCCGCCCCCTGGACACCAGCCTGCGCCTCAAGA : 70
SOCS3 : ATGGTCACCCACAGCAAGTTCCCCGCCGCCGGGATGAGCCGCCCCCTGGACACCAGCCTGCGCCTCAAGA : 70

REV : CGTTCAGCTCCAAGAGCGAGTACCAGCTGGTGGTGAACACCGTGCGCAAGCTGCAGGAGAGCGGCTTCTA : 140
FWD : CGTTCAGCTCCAAGAGCGAGTACCAGCTGGTGGTGAACACCGTGCGCAAGCTGCAGGAGAGCGGCTTCTA : 140
SOCS3 : CGTTCAGCTCTAAGAGCGAGTACCAGCTGGTGGTGAACACCGTGCGCAAGCTGCAGGAGAGCGGCTTCTA : 140

REV : CTGGAGCACGGTGACGGGCGGCGAGGCCAACGTGCTGCTGAGCGCCGAGCCCGCCGGCACCTTCCTCATC : 210
FWD : CTGGAGCACGGTGACGGGCGGCGAGGCCAACGTGCTGCTGAGCGCCGAGCCCGCCGGCACCTTCCTCATC : 210
SOCS3 : CTGGAGCACGGTGACGGGCGGCGAGGCCAACGTGCTGCTCAGCGCCGAGCCCGCCGGCACCTTCCTCATC : 210

REV : AGGGACAGCTCGGACCAGCGGCACTTCTTCACCCTCAGCGTCAAGACGGAGTCGGGCACCAAGAACCTGC : 280
FWD : AGGGACAGCTCGGACCAGCGGCACTTCTTCACCCTCAGCGTCAAGACGGAGTCGGGCACCAAGAACCTGC : 280
SOCS3 : AGGGACAGCTCGGACCAGCGGCACTTCTTCACCCTCAGCGTCAAGACGGAGTCGGGCACCAAGAACCTGC : 280

REV : GCATCCAGTGCGAGGGCGGCAGCTTCTCTCTGCAGAGCGACCCGCGCAGCAGCCAACCCGTGCCCCGCTT : 350
FWD : GCATCCAGTGCGAGGGCGGCAGCTTCTCTCTGCAGAGCGACCCGCGCAGCAGCCAACCCGTGCCCCGCTT : 350
SOCS3 : GCATCCAGTGCGAGGGCGGCAGCTTCTCTCTGCAGAGCGACCCGCGCAGCAGCCAACCCGTGCCCCGCTT : 350

REV : CGACTGCGTGCTGAAGCTGGTGCACCACTACATGCCTCCCACCCCCTGCGCCGGGCCCAAGCAGCCCGGA : 420
FWD : CGACTGCGTGCTGAAGCTGGTGCACCACTACATGCCTCCCACCCCCTGCGCCGGGCCCAAGCAGCCCGGA : 420
SOCS3 : CGACTGCGTGCTGAAGCTGGTGCACCACTACATGCCTCCCACCCCCTGCGCCGGGCCCAAGCAGCCCGGA : 420

REV : GGGGCCCTGCACCCCAAACGCACCTACTACATTTACTCGGGTGGAGAGAAGATCCCGCTGGTGCTCAGCC : 490
FWD : GGGGCCCTGCACCCCAAACGCACCTACTACATTTACTCGGGTGGAGAGAAGATCCCGCTGGTGCTCAGCC : 490
SOCS3 : GGGGCCCTGCACCCCAAACGCACCTACTACATTTACTC..GTGGAGAGAAGATCCCGCTGGTGCTCAGCC : 488

REV : GCCCGCTCTCCTCCAGCGTCTCCACGCTGCAGCACCTCTGCCGTAAGACTGTCAACGGGCACCTGGACTC : 560
FWD : GCCCGCTCTCCTCCAGCGTCTCCACGCTGCAGCACCTCTGCCGTAAGACTGTCAACGGGCACCTGGACTC : 560
SOCS3 : GCCCGCTCTCCTCCAGCGT.TCCACGCTGCAGCACCTCTGCCGTAAGACTGTCAACGGGCACCTGGACTC : 557

REV : CTACGAGAAGATGACTCAGCTGCCAGCCCCTATCAAGGAGTTTTCTGGACCAGTACGATGCCCCCCTCTA : 630
FWD : CTACGAGAAGATGACTCAGCTGCCAGCCCCTATCAAGGAGTTT.CTGGACCAGTACGATGCCCCCCTCTA : 629
SOCS3 : CTACGAGAAGATGACTCAGCTGCCAGCCCCTATCAAGGAGTTT.CTGGACCAGTACGATGCCCCCCTCTA : 626

REV : A : 631
FWD : A : 630
SOCS3 : A : 627

STEAP1
REV : ATGGAGAAGAGAGAAGGTGATAGTCATGCCATTGACCAGAATGCATTTCAGAACATCATACCAAGAAGAA : 70
STEAP1 : ATGGAGAAGAGAGAAGGTGATAGTCATGCCATTGACCAGAATGCATTTCAGAACATCATACCAAGAAGAA : 70
FWD : ATGGAGAAGAGAGAAGGTGATAGTCATGCCATTGACCAGAATGCATTTCAGAACATCATACCAAGAAGAA : 70

REV : ATAGAGAAAACCTTAACGACTTGAATGTGGATATGCAAGTCGCCAATCCACCAGAAGTAGCTGCACTTTT : 140
STEAP1 : ATAGAGAAAACCTTAACGACTTGAATGTGGATATGCAAGTCGCCAATCCACCAGAAGTAGCTGCACTTTT : 140
FWD : ATAGAGAAAACCTTAACGACTTGAATGTGGATATGCAAGTCGCCAATCCACCAGAAGTAGCTGCACTTTT : 140

REV : TAACTTACATCAAGCACACCATTTTAGTGAGTTTGAGTATTCTTCAGAACAACACTACAAGCAGGATTTG : 210
STEAP1 : TAACTTACATCAAGCACACCATTTTAGTGAGTTTGAGTATTCTTCAGAACAACACTACAAGCAGGATTTG : 210
FWD : TAACTTACATCAAGCACACCATTTTAGTGAGTTTGAGTATTCTTCAGAACAACACTACAAGCAGGATTTG : 210

REV : TTCCCCAGGTGGCACATGCCACTGAAGATAGCATCTGTGATCTCATTATTGACGTTTATTTACACTTCTC : 280
STEAP1 : TTCCCCAGGTGGCACATGCCACTGAAGATAGCATCTGTGATCTCATTATTGACGTTTATTTACACTTCTC : 280
FWD : TTCCCCAGGTGGCACATGCCACTGAAGATAGCATCTGTGATCTCATTATTGACGTTTATTTACACTTCTC : 280

REV : TAAGAGATGTCATATATCCTTTTATAGCAAGAAATGAAAATGTTTTCTATAAAATTCCAATCCTTGTCAT : 350
STEAP1 : TAAGAGATGTCATATATCCTTTTATAGCAAGAAATGAAAATGTTTTCTATAAAATTCCAATCCTTGTCAT : 350
FWD : TAAGAGATGTCATATATCCTTTTATAGCAAGAAATGAAAATGTTTTCTATAAAATTCCAATCCTTGTCAT : 350

REV : AAACAAAGTCTTACCAGTGGTTTCAATTACCCTTTTAGCACTGGTATATTTGCCAGGAATATTAGCTGCT : 420
STEAP1 : AAACAAAGTCTTACCAGTGGTTTCAATTACCCTTTTAGCACTGGTATATTTGCCAGGAATATTAGCTGCT : 420
FWD : AAACAAAGTCTTACCAGTGGTTTCAATTACCCTTTTAGCACTGGTATATTTGCCAGGAATATTAGCTGCT : 420

REV : GGTTTCCAGCTGTATTTTGGCACTAAGTACAAAAGGTTTCCCCTGTGGCTGGATAGATGGATGTTGTCAA : 490
STEAP1 : GGTTTCCAGCTGTATTTTGGCACTAAGTACAAAAGGTTTCCCCTGTGGCTGGATAGATGGATGTTGTCAA : 490
FWD : GGTTTCCAGCTGTATTTTGGCACTAAGTACAAAAGGTTTCCCCTGTGGCTGGATAGATGGATGTTGTCAA : 490

REV : GAAAGCAATTTGGACTTCTCAGTTTCTTCTTCGCTGTGATGCATGCCTGCTATAGCCTATGCTATCCAAT : 560
STEAP1 : GAAAGCAATTTGGACTTCTCAGTTTCTTCTTCGCTGTGATGCATGCCTGCTATAGCCTATGCTATCCAAT : 560
FWD : GAAAGCAATTTGGACTTCTCAGTTTCTTCTTCGCTGTGATGCATGCCTGCTATAGCCTATGCTATCCAAT : 560

REV : GAGAAGATCATACAGATACAAGCTGCTGAACTGGGCATTCCAGCAGGTCAAACAGAAAAAAGAAAATGCC : 630
STEAP1 : GAGAAGATCATACAGATACAAGCTGCTGAACTGGGCATTCCAGCAGGTCAAACAGAAAAAAGAAAATGCC : 630
FWD : GAGAAGATCATACAGATACAAGCTGCTGAACTGGGCATTCCAGCAGGTCAAACAGAAAAAAGAAAATGCC : 630

REV : TGGATTGAACATGATGTTTGGAGAATGGAGATTTATGTGTCCCTAGGAATTCTGGGACTTGCTTTGCTGG : 700
STEAP1 : TGGATTGAACATGATGTTTGGAGAATGGAGATTTATGTGTCTCTAGGAATTCTGGGACTTGCTTTGCTGG : 700
FWD : TGGATTGAACATGATGTTTGGAGAATGGAGATTTATGTGTCCCTAGGAATTCTGGGACTTGCTTTGCTGG : 700

REV : CCTTATTGGCAATAGCATCAATCCCATCTGTCAGTCACTCTTTGACCTGGAGAGAGTTC : 759
STEAP1 : CCTTATTGGCAATAGCATCAATCCCATCTGTCAGTCACTCTTTGACCTGGAGAGAGTTC : 759
FWD : CCTTATTGGCAATAGCATCAATCCCATCTGTCAGTCACTCTTTGACCTGGAGAGAGTTC : 759

STEAP4
STEAP4 : ATGAATAAAAATTCTTCCAACATAATGGCTTTGGTTCCCAAAACATCTAACAAAAGAGAGACAGTGTGTA : 70
FWD : ATGAATAAAAATTCTTCCAACATAATGGCTTTGGTTCCCAAAACATCTAACAAAAGAGAGACAGTGTGTA : 70

STEAP4 : TATTTGGAACGGGAGATTTTGGAAGAGCTCTGGGCCATAAATTGATTCAGTCTGGTTACCCTGTTGTGTT : 140
FWD : TATTTGGAACGGGAGATTTTGGAAGAGCTCTGGGCCATAAACTGATTCAGTCTGGTTACCCTGTTGTGTT : 140

STEAP4 : TGGAAGCCGGAGCCCATGGCCATCCAGCCTGATTCCCAAGGATGCAGAGGTGCTGAACCATGCTGAGGCA : 210
FWD : TGGAAGCCGGAGCCCATGGCCATCCAGCCTGATTCCCAAGGATGCAGAGGTGCTGAACCATGCTGAGGCA : 210

STEAP4 : GCACAGAAAGCTGCCATCATTATTATAGCAATCCAGAGGCAACATTATGACTTCCTTGCATCACTGGAGG : 280
FWD : GCACAGAAAGCTGCCATCATTATTATAGCAATCCAGAGGCAACATTACGACTTCCTTGCATCACTGGAGG : 280

STEAP4 : AGACACTCCATGGAAAAGTGTTGGTGGACATAAGCAACAACTTAAAAATAAACCAGTATCCTGAATCCAA : 350
FWD : AGACACTCCGTGGAAAAGTGTTGGTGGACATAAGCAACAACTTAAAAATAAACCAGTATCCTGAATCCAA : 350

STEAP4 : TGCAAAGTACCTGGCTCAGCTGGTGCCCGGTGCTAGGGTTGTGAAAGCCTTTAACACCGTGTCTGCCTGG : 420
FWD : TGCAAAGTACCTGGCTCAGCTGGTGCCCGGTGCTAGGGTTGTGAAAGCCTTTAACACCGTGTCTGCCTGG : 420

STEAP4 : GCTCTGCAGTCAGGCACCCTGGATGCAAGTCGGCAGGTGTTTGTCTGTGGAGATGACACAGAAGCTAAGC : 490
FWD : GCTCTGCAGTCAGGCACCCTGGATGCAAGTCGACAGGTGTTTGTCTGTGGAGATGACACAGAAGCTAAGC : 490

STEAP4 : AAATGGTAATGGATATTATTCGTGCACTGGGACTCACACCGTTAGATCAAGGATCTCTCTTGGCTGCTCG : 560
FWD : AAATGGTAATGGATATTATTCGTGCACTGGGACTCACACCGTTAGATCAAGGATCTCTCTTGGCTGCTCG : 560

STEAP4 : GGAAATAGAAAATTACCCTCTACAGCTCTTTCCAATGTGGAAGATTCCCATCTTTTTGTCCCTTGGCCTA : 630
FWD : GGAAATAGAAAATTACCCTCTACAGCTCTTTCCAATGTGGAAGATTCCCATCTTTTTGTCCCTTGGCCTA : 630

STEAP4 : ACTGCATTCTTCTTCTTCTACTGTTTGGTTCGTGACGTAATTTACCCTTACGTTTATGAAAATAAGGATT : 700
FWD : ACTGCATTCTTCTTCTTCTACTGTTTGGTTCGTGACGTAATTTACCCTTACGTTTATGAAAATAAGGATT : 700

STEAP4 : ATTCATTTTTTCTTGCAATTTCCATTCCAAATCGGATCTGCCCTATAATTGCACTCGTCCTTCTTGCCTT : 770
FWD : ATTCATTTTTTCTTGCAATTTCCATTCCAAATCGGATCTGCCCTATAATTGCACTCGTCCTTCTTGCCTT : 770

STEAP4 : GGTTTATCTCCCTGGTATACTTGCTGCAATTATTCAGTTATACAGAGGTACCAAATACAGCCGTTTCCCA : 840
FWD : GGTTTATCTCCCTGGTATACTTGCTGCAATTATTCAGTTATACAGAGGTACCAAATACAGCCGTTTCCCA : 840

STEAP4 : GACTGGCTGGACAAGTGGATGCTGTGTAGGAAACAACTTGGACTAGTAGCCTTGGCATTTGCTTCGCTGC : 910
REV : GACTGGCTGGACAAGTGGATGCTGTGTAGGAAACAACTTGGACTAGTAGCCTTGGCATTTGCTTCGCTGC : 225

STEAP4 : ACGTTATTTATACTCTTGTTATCCCAATTCGCTACTATGTAAGATGGAGAATTGAGGACAGAACCATCTC : 980
REV : ACGTTATTTATACTCTTGTTATCCCAATTCGCTACTATGTAAGATGGAGAATTGAGGACAGAACCATCTC : 295

STEAP4 : ACAGGCACTGAACAATAAAACTACTCCATTTGACAACACAAATGGCTGGCTTAGCGACTCTTATTTGGCT : 1050
REV : ACAGGCACTGAACAATAAAACTACTCTATTTGACAACACAAATGGCTGGCTTAGCGACTCTTATTTGGCT : 365

STEAP4 : TTGGGGATTTTAGGATTCCTTTTATTTGTTCTTCTTGGAATAACTTCCTTGCCTTCCGTCAGCAACAATG : 1120
REV : TTGGGGATTTTAGGATTCCTTTTATTTGTTCTTCTTGGAATAACTTCCTTGCCTTCCGTCAGCAACAATG : 435

STEAP4 : TCAACTGGAGAGAATTTCGATTTGTACAGTCCAAACTGGGATATCTGACATTGATTTTATGCACCGCACA : 1190
REV : TCAACTGGAGAGAATTTCGATTTGTACAGTCCAAACTGGGATATCTGACATTGATTTTATGCACCGCACA : 505

STEAP4 : CACACTGGTTTATGGTGGAAAGCGGTTCCTAAGTCCATCAGCATACAGATGGTATCTTCCAAATGCCTAT : 1260
REV : CACACTGGTTTATGGTGGAAAGCGGTTCCTAAGTCCATCAGCATACAGATGGTATCTTCCAAATGCCTAT : 575

STEAP4 : ATGCTCTCTCTCATTATTCCATGCATTGTACTGGTTGTCAAATTTGTGCTCATACTTCCTTGTCTAGACA : 1330
REV : ATGCTCTCTCTCATTATTCCATGCATTGTACTGGTTGTCAAATTTGTGCTCATACTTCCTTGTCTAGACA : 645

STEAP4 : AACAACTAACACGAATTCGACAGGGCTGGGAGAGGAATCCCCAATACTCAGAACAGTCAAACTACGTTAT : 1400
REV : AACAACTAACACGAATTCGACAGGGCTGGGAGAGGAATCCCCAGTACTCAGAACAGTCAAACTACGTTAT : 715

STEAP4 : CAACAAGTCTGCTGTGTAA : 1419
REV : CAACAAGTCTGCTGTGTAA : 734

TGM4
TGM4 : ATGAGCCAAGACAGCGACCTGAAAGTTACCAAGGTTGACTTCCTCAAGAGCCAGAACTCGGTGCAGCACC : 70
FWD : ATGAGCCAAGACAGCGACCTGAAAGTTACCAAGGTTGACTTCCTCAAGAGCCAGAACTCGGTGCAGCACC : 70

TGM4 : ACACGGATGCGTACAACACCTCAAACCTGGTGGTGCGGCGTGGGCAGCCCTTCCTGCTGCAGCTGACCCT : 140
FWD : ACACGGATGCGTACAACACCTCAAACCTGGTGGTGCGGCGTGGGCAGCCCTTCCTGCTGCAGCTGACCCT : 140

TGM4 : CAGCAGGGAGCTGCGAGCCGCCGACAAGCTGTCACTGCATTTCAGCATCGGTGAAAGGCCAATGGAGCCC : 210
FWD : CAGCAGGGAGCTGCGAGCCGCCGACAAGCTGTCACTGCATTTCAGCATCGGTGAAAGGCCAATGGAGCCC : 210

TGM4 : ACAGGGACCCTGATGTCACTGAACCCCAGGAGCACGAGGAATGTCAGCGGTTGGCAAATCGCCATCATCA : 280
FWD : ACAGGGACCCTGATGTCACTGAACCCCAGGAGCACGAGGAATGTCAGCGGTTGGCAAATCGCCATCATCA : 280

TGM4 : AGTCCAGTGGCACAGAGTGCACGCTGTCTGTCACCAGCGCACCCAATGCTGCCGTGGGAATATATGGACT : 350
FWD : AGTCCAGTGGCACAGAGTGCACGCTGTCTGTCACCAGCGCACCCAATGCTGCCGTGGGAATATATGGACT : 350

TGM4 : GATGGTGAAGACTGGCCCTAATATTTACAAGCCCGAGAAGAACACTGTCTACCTTCTGTTCAACCCTTGG : 420
FWD : GATGGTGAAGACTGGCCCTAATATTTACAAGCCCGAGAAGAACACTGTCTACCTTCTGTTCAACCCTTGG : 420

TGM4 : GGTGAAGGTGATATTGTCTTCCTGTCCAACGAGGCTGAGAGAAAGGAGTACGTACTCAACGATACGGGCT : 490
FWD : TGTGAAGGTGATATTGTCTTCCTGTCCAACGAGGCTGAGAGAAAGGAGTACGTACTCAACGATACGGGCT : 490

TGM4 : ACATCTATGTTGGGTCTGCCTTCAACATACACAGCAAACCCTGGAATTTCGGGCAGTTTGAAGAATCAAT : 560
FWD : ACATCTATGTTGGGTCTGCCTTCAACATACACAGCAAACCCTGGAATTTCGGGCAGTTTGAAGAATCAAT : 560

TGM4 : CCTGGATGCCTGCATGTACCTGCTGGACAAAAGCAAACTCAAGATGAGCAGTAGAAGGGATCCTGTGGTT : 630
FWD : CCTGGATGCCTGCATGTACCTGCTGGACAAAAGCAAACTCAAGATGAGCAGTAGAAGGGATCCTGTGGTT : 630

TGM4 : GTATCCAGAGCCATGTCTGCTTTGGTGAATGCCAACGATGACAATGGTGTCGTGCTGGGAAACTGGTCAG : 700
FWD : GTATCCAGAGCCATGTCTGCTTTGGTGAATGCCAACGATGACAATGGTGTCGTGCTGGGAAACTGGTCAG : 700

TGM4 : GGAAGTACGAGAACGGGACTTCCCCCATGGCATGGATTGGGAGCGTCGCAATTTTGCAGCAGTATTACAA : 770
FWD : GGAAGTACGAGAACGGGACTTCCCCCATGGCATGGATTGGGAGCGTCGCAATTTTGCAGCAGTATTACAA : 770

TGM4 : AACCAAGAAGCCTGTCAGTTATGGTCAATGTTGGGTCTTCTCAGGAGTCCTCACTACAGTCATGCGCTGC : 840
FWD : AACCAAGAAGCCTGTCAGTTATGGTCAATGTTGGGTCTTC.............................. : 810

TGM4 : CTGGGGATCCCAGCCCGCAGTGTGAGCAACTTCAACTCTGCGCATGACACCGATGAGAACCTGAGGGTTG : 910
FWD : ...................................................................... : -

TGM4 : ATGTCTACCTGAACGAGAAAGGAGAAAAGCTGAAATGGATGTCCTCTGACTCCGTCTGGAACTTCCACGT : 980
FWD : ...................................................................... : -

TGM4 : GTGGAATGATGTCTGGATGAAGCGGAAGGACCTGCCATCAGGGTTTGATGGCTGGCAGGCAATCGACGCG : 1050
REV : ...................................................................... : -

TGM4 : ACCCCTCAGGAGCAAAGTCAAGGTACTTTCCAGTGTGGCCCGTGCCCACTGAAGGCCGTCAAAGAGGGAG : 1120
REV : ...................................................................... : -

TGM4 : ACGTGTATTTGCCCTACGACAGCAAGTTTGTGTACGCGGAAGTGAACGCCGACAAGGTCTACTGGCGCGT : 1190
REV : ...................................................................... : -

TGM4 : CAAGGAGGAGAACGGCAGGAACAAGTACACCAAGCTGGGCGTGGAGAGCCAGAGCATCGGCGCCAACATC : 1260
REV : ...................................................................... : -

TGM4 : AGCACAAAGGCCGTGGGGCAGAACAGGCGGGAGGACATCACCTGGCAGTACAAGTTCCCTGAAGGCTCTG : 1330
REV : ..........CCGTGGGGCAGAACAGGCGGGAGGACATCACCTGGCAGTACAAGTTCCCTGAAGGCTCTG : 60

TGM4 : CAGAGGAAAGGGCATCCATGAAGAGAGCTGTATCCTACCTGCAGCCCTCGGGGTTGACGCCTCGTTCACG : 1400
REV : CAGAGGAAAGGGCATCCATGAAGAGAGCTGTATCCTACCTGCAGCCCTCGGGGTTGACGCCTCGTTCACG : 130

TGM4 : CTTTGCTGCAGTCCCCATGGAAGTGAGCCTAAGAAACGTGAGTGACAAGGACACGGTTCAAAACGAGGTG : 1470
REV : CTTTGCTGCAGTCCCCATGGAAGTGAGCCTAAGAAACGTGAGTGACAAGGACACGGTTCAAAACGAGGTG : 200

TGM4 : GTGCCCAAGTCTGGGGTCCAGCTGGAGATAACCAACGAGAAGCCTCTGTGTCCTGGTAATCCCATTGAGG : 1540
REV : GTGCCCAAGTCTGGGGTCCAGCTGGAGATAACCAACGAGAAGCCTCTGTGTCCTGGTAATCCCATTGAGG : 270

TGM4 : TGACCATCACCGTGAAGAGCACTGTGGCCGGGAGCTGGACTGTCGACCTTGCCAGCTCCTGCCAGCTGCA : 1610
REV : TGACCATCACCGTGAAGAGCACTGTGGCCGGGAGCTGGACTGTCGACCTTGCCAGCTCCTGCCAGCTGCA : 340

TGM4 : GTCCTACACTGGGAAAGTCCATGCCAATCTTGGATACATCAAGCAGACTGTCAAGGTGGAAGGCCAATCT : 1680
REV : GTCCTACACTGGGAAAGTCCATGCCAATCTTGGATACGTCAAGCAGACTGTCAAGGTGGAAGGCCAATCT : 410

TGM4 : GAGGTGCACGTCCCGCTGAAGATCATGCCCGATGCTTACATGAAAGCATTGGCTACAGTGGATGATGAAG : 1750
REV : GAGGTGCACGTCCCGCTGAAGATCATGCCCGATGCTTACATGAAAGCATTGGCTACAGTGGATGATGAAG : 480

TGM4 : AGCACGTCCACGTCACTGCCATTGCTGAGATCCAGGGGACACCTGAGAAGCTCACCAAGGAGGCGTCACT : 1820
REV : AGCACGTCCACGTCACTGCCATTGCTGAGATCCAGGGGACACCTGAGAAGCTCACCAAGGAGGCGTCACT : 550

TGM4 : GAGCTTCGAGTACCCTCCCATCCAGGTCCAGATGCCAGAAACGGCAAAGGTGAACAACGACTTCACCTGC : 1890
REV : GAGCTTCGAGTACCCTCCCATCCAGGTCCAGATGCCAGAAACGGCAAAGGTGAACAACGACTTCACCTGC : 620

TGM4 : GCCTTCATCTTCAAGAACAAGCTGAACGTTCCCCTGGATAACTGCAAGCTGATGGTGGAGGGCTTGGGCA : 1960
REV : GCCTTCATCTTCAAGAACAAGCTGAACGTTCCCCTGGATAACTGCAAGCTGATGGTGGAGGGCTTGGGCA : 690

TGM4 : TATTTAAGATGGCGACGTTTGATGAAGGGGATATACAGCCGGGCAGGATTATTAAGTCTGAAGTCATATG : 2030
REV : TATTTAAGATGGCGACGTTTGATGAAGGGGATATACAGCCGGGCAGGATTATTAAGTCTGAAGTCATATG : 760

TGM4 : CACTCCAACGAGAGTAGGAGAGAAGAAGATCGTGGCCAGGCTGACCTCCAACCAGGTCAAAGACATTTCG : 2100
REV : CACTCCAACGAGAGTAGGAGAGAAGAAGATCGTGGCCAGGCTGACCTCCAACCAGGTCAAAGACATTTCG : 830

TGM4 : GTGGAGAAGGCCATCACGGTCACCCACTAG : 2130
REV : GTGGAGAAGGCCATCACGGTCACCCACTAG : 860

TIRAP
TIRAP : ATGGCCGGATGGTTTAGGCGGCTCCTGCAGAAGCCCAAGCAGAGCTCCATCCACGCATCGAGCAGCTCTC : 70
FWD : ...................................................................... : -
REV : ATGGCCGGATGGTTTAGGCGGCTCCTGCAGAAGCCCAAGCAGAGCTCCATCCACGCATCGAGCAGCTCTC : 70

TIRAP : ATAGCACCACCAGCCACTCACTTTCATCCTCACCCTCCTCCTTTTCCTCCTCCTCCTCTGCTTGGAGCTC : 140
FWD : ............GCCACTCACTTTCATCCTCACCCTCCTCCTTTTCCTCCTCCTCCTCTGCTTGGAGCTC : 58
REV : ATAGCACCACCAGCCACTCACTTTCATCCTCACCCTCCTCCTTTTCCTCCTCCTCCTCTGCTTGGAGCTC : 140

TIRAP : CAGCTCCTCCAGCAGCACCAGCACAGCTCAGCCTAGCCGCCCAGCCCCGGTGGACATCAGCAGCTCGAGC : 210
FWD : CAGCTCCTCCAGCAGCACCAGCACAGCTCAGCCTAGCCGCCCAGCCCCGGTGGACATCAGCAGCTCGAGC : 128
REV : CAGCTCCTCCAGCAGCACCAGCACAGCTCAGCCTAGCCGCCCAGCCCCGGTGGACATCAGCAGCTCGAGC : 210

TIRAP : AGCGCACGCTGGGTGAAGAGCTATGATGTGTGCATCTGCCACAGTGAGGTGGACCTGGAGTTTGTGGAGG : 280
FWD : AGCGCACGCTGGGTGAAGAGCTATGATGTGTGCATCTGCCACAGTGAGGTGGACCTGGAGTTTGTGGAGG : 198
REV : AGCGCACGCTGGGTGAAGAGCTATGATGTGTGCATCTGCCACAGTGAGGTGGACCTGGAGTTTGTGGAGG : 280

TIRAP : AGCTGGTGTCCTACCTGGAGAGCCAACCCCAGAGCCTGCGCTGTTTCCTGCAGCTGCGGGACAGCGTGGC : 350
FWD : AGCTGGTGTCCTACCTGGAGAGCCAACCCCAGAGCCTGCGCTGTTTCCTGCAGCTGCGGGACAGCGTGGC : 268
REV : AGCTGGTGTCCTACCTGGAGAGCCAACCCCAGAGCCTGCGCTGTTTCCTGCAGCTGCGGGACAGCGTGGC : 350

TIRAP : GGGCAGTGCTGTCATGACAGAGCTGTGTGAGGCTGTGCAGAACAGCCACTGCTGGGTGATGCTCATCACC : 420
FWD : GGGCAGTGCTGTCATGACAGAGCTGTGTGAGGCTGTGCAGAACAGCCACTGCTGGGTGATGCTCATCACC : 338
REV : GGGCAGTGCTGTCATGACAGAGCTGTGTGAGGCTGTGCAGAACAGCCACTGCTGGGTGATGCTCATCACC : 420

TIRAP : CCCAGCTTCCTGCAGGACCCCTGGTGCAGGTACCAGATGCACCAGGCGCTAGCAGAGGCCCCGATGGCCA : 490
FWD : CCCAGCTTCCTGCAGGACCCCTGGTGCAGGTACCAGATGCACCAGGCGCTAGCAGAGGCCCCGATGGCCA : 408
REV : CCCAGCTTCCTGCAGGACCCCTGGTGCAGGTTCCAGATGCACCAGGCGCTAGCTGAGGCCCCGATGGCCA : 490

TIRAP : ATGGGCGTACCATCCCTGTGCTGAAGGACATTGACAGGAAGGATTACCCCAGGGAGCTGAGGAACCTCTA : 560
FWD : ATGGGCGTACCATCCCTGTGCTGAAGGACATTGACAGGAAGGATTACCCCAGGGAGCTGAGGAACCTCTA : 478
REV : ATGGGCGTACCATCCCTGTGCTGAAGGACATTGACAGGAAGGATTACCCCAGGGAGCTGAGGAACCTCTA : 560

TIRAP : CTACATCTACGTGGCTCTCAAGGAGAACAGCTTCAGGCAGATCAGGGACACTGTGCTGCGCTACCTGGAA : 630
FWD : CTACATCTACGTGGCTCTCAAGGAGAACAGCTTCAGGCAGATCAGGGACACTGTGCTGCGCTACCTGGAA : 548
REV : CTACATCTACGTGGCTCTCAAGGAGAACAGCTTCAGGCATATCAGGGACACTGTGCTGCGCTACCTGGAA : 630

TIRAP : GAGCTGTGCCGGAGCTCCACGAGTGGAATGCAGTAG : 666
FWD : GAGCTGTGCCGGAGCTCCACGAGTG........... : 573
REV : TAGCTGTGCCGGATCTCCACGAGTGGAATGCAGTAG : 666

TNFAIP3
TNFAIP3 : ATGGCTGGCCAACACATCCTTCCTCAGGCTTTGTATCAGAGCAATATGCTGAAAGCGGTGAAGATTAGAG : 70
FWD : ATGGCTGGCCAACACATCCTTCCTCAGGCTTTGTATCAGAGCAATATGCTGAAAGCGGTGAAGATTAGAG : 70

TNFAIP3 : AGAGGACACCTGAAGATCTGGTCAAACCCCCCAGTGGAATAATTCACCACTTCAGGACTATGCACAGATA : 140
FWD : AGAGGACACCTGAAGATCTGGTCAAACCCCCCAGTGGAATAATTCACCACTTCAGGACTATGCACAGATA : 140

TNFAIP3 : CACTATAGAAATGTTCAGAATGTGCCAGTTTTGCCCTCAATTCCGAGAAACGCTTCAGAAGGCCCTGACT : 210
FWD : CACTATAGAAATGTTCAGAATGTGCCAGTTTTGCCCTCAATTCCGAGAAACGCTTCAGAAGGCCCTGACT : 210

TNFAIP3 : GACCAAGCCACCCAGGCTTCGCTGGAGCGCCAGAGGAAGCTGAACTGGTGCATGGAGGTCAGGAGACTTG : 280
FWD : GACCAAGCCACCCAGGCTTCGCTGGAGCGCCAGAGGAAGCTGAACTGGTGCATGGAGGTCAGGAGACTTG : 280

TNFAIP3 : TGCCTTTGAAAACTAATGGTGATGGAAATTGCCTCATGCATGCTGCATCGCAGTACATGTGGGGTATTGA : 350
FWD : TGCCTTTGAAAACTAATGGTGATGGAAATTGCCTCATGCATGCTGCATCGCAGTACATGTGGGGTATTGA : 350

TNFAIP3 : AGATGTTGACCTCGTCTTAAGGAAGACATTGTTTAGTGCGCTCAGGGAGATTGACACACGGAACTTCAAG : 420
FWD : AGATGTTGACCTCGTCTTAAGGAAGACATTGTTTAGTGCTCTCAGGGAGATTGACACACGGAACTTCAAG : 420

TNFAIP3 : CTGCGCTGGCAGCGGGAGGCTATTAAATCCCAGGAGTTTGTGCAAACGGGACTCCACTTTGACACCCGGA : 490
FWD : CTGCGCTGGCAGCGGGAGGCTATTAAATCCCAGGAGTTTGTACAAACGGGACTCCACTTTGACACCCGGA : 490

TNFAIP3 : ACTGGGAGGAGGAGTGGGAATACCTCATTGAAATGACCTCCCCAGAAACATCTGGGGCTCGAAACAGACT : 560
FWD : ACTGGGAGGAGGAGTGGGAATACCTCATTGAAATGACCTCCCCAGAAACATCTGGGGCTCGAAACAGACT : 560

TNFAIP3 : TCCTTATAATGCACTGGAAGAAATCCACATCTTCGTTCTTGCCAACATCCTCAGAAGACCAATCGTTGTC : 630
FWD : TCCTTATAATGCACTGGAAGAAATCCACATCTTCGTTCTTGCCAACATCCTCAGAAGACCAATCGTTGTC : 630

TNFAIP3 : CTTGCAGATAAAGTTGTGAGAAGTTTAGAATCTGGCTCCAGTTTTGCTCCTTTGAATGTTGGTGGTGTTT : 700
FWD : CTTGCAGATAAAGTTGTGAGAAGTTTAGAATCAGGCTCCAGTTTTGCTCCTCTGAATGTTGGTGGTGTTT : 700

TNFAIP3 : ACCTGCCACTCCTTTGGCCAGGTGAAGAATGCTACAGATATCCAATTGTGCTCGGCTACGACAATATGCA : 770
FWD1 : ACCTGCCACTCCTTTGGCCAGGTGAAGAATGCTACAGATATCCAATTGTGCTCGGCTACGACAATATGCA : 259

TNFAIP3 : TTTTACACCACTTGTGACCCTGAAGGATAGCGGGCCAGAAATCCGGGCTGTCCCTCTGGTCACCAGCGAA : 840
FWD1 : TTTTACACCACTTGTGACCCTGAAGGATAGCGGGCCAGAAATCCGGGCTGTCCCTCTGGTCACCAGCGAA : 329

TNFAIP3 : CGAGGCAGATTCGAGGATTTGAGCGTGCACTTTCTGACAGACGCAGAGGAGAGGGAGAAAGAGCAGCTGT : 910
FWD1 : CGAGGCAGATTCGAGGATTTGAGCGTGCACTTTCTGACAGACGCAGAGGAGAGGGAGAAAGAGCAGCTGT : 399

TNFAIP3 : TGAAAGACTACTTGATAGTCATAGAAATTCCAGTGCAAGGCTGGGATCATGGCACAACTCATTTGATTAA : 980
FWD1 : TGAAAGACTACTTGATAGTCATAGAAATTCCAGTGCAAGGCTGGGATCATGGCACAACTCATTTGATTAA : 469

TNFAIP3 : TGCTGCAAAGTTGGATGAAGGCAACCTGCCCAAAGAAATAAATCTTGTGGAAGATTACTTTCAACTGGTG : 1050
FWD1 : TGCTGCAAAGTTGGATGAAGGCAACCTGCCCAAAGAAATAAATCTTGTGGAAGATTACTTTCAACTGGTG : 539

TNFAIP3 : CAGCATGAGTACAAGAAATGGCAGGAGCACGCTGAACCTGCCAGAAAAGAGGCCTGCTCCAGGAACAAAC : 1120
FWD1 : CAGCATGAGTACAAGAAATGGCAGGAGCACGCTGAACCTGCCAGAAAAGAGGCCTGCTCCAGGAACAAAC : 609

TNFAIP3 : AGGACCTGTCTTTTGCCCAGCTCTCCCTCTTACAGGTGAAATGTGAAACTCCAAACTGCCCTTTCTATAT : 1190
FWD1 : AGGACCTGTCTTTTGCCCAGCTCTCCCTCTTACAGGTGAAATGTGAAACTCCAAACTGCCCTTTCTATAT : 679

TNFAIP3 : GTCTGTGAACACCCAGCCCTACTGCCACGAGTGCTTTGAGCGGAGGTCGCAGGGAAGCAAAGGAAGAAAG : 1260
FWD1 : GTCTGTGAACACCCAGCCCTACTGCCACGAGTGCTTTGAGCGGAGGTCGCAGGGAAGCAAAGGAAGAAAG : 749

TNFAIP3 : CAGGTCTCTGAAGCAGCACCCGAGAAACTGAAGGCAGCTGGGTCAGGCTCCTCCTGTGGTAAAGCTTGTG : 1330
FWD1 : CAGGTCTCTGAAGCAGCACCCGAGAAACTGAAGGCAGCTGGGTCAGGCTCCTCCTGTGGTAAGCTTTGTG : 819

TNFAIP3 : AACCTGGGGGGTGGATGTCCGAGGAGGCTGTAACAGGGCCTCGCTCTGCACCCCCAACTGCTCCCAGCCT : 1400
FWD2 : AACCTGGGGGGTGGATGTCCGAGGAGGCTGTAACAGGGCCTCGCTCTGCACCCCCAACTGCTCCCAGCCT : 420

TNFAIP3 : TTTCCTCTATAGTGAAACCACTGCCATGAAATGCCGGACCCCAGACTGCCCCTTCACCTTAAACGTGCAA : 1470
FWD2 : TTTCCTCTATAGTGAAACCACTGCCATGAAATGCCGGACCCCAGACTGCCCCTTCACCTTAAACGTGCAA : 490

TNFAIP3 : CACAACGGACTTTGTGAACGCTGCTACAATGCTAGACAGCTTGCTCCTTATAACAATTTGGATGACCAGA : 1540
FWD3 : CACAACGGACTTTGTGAACGCTGCTACAATGCTAGACAGCTTGCTCCTTATAACAATTTGGATGACCAGA : 82

TNFAIP3 : GATGTTTGGACTATGCCACGTGTAAGGTCTGCCTTCAGAAGGCCAATAGGACATTTAATGGCATATGCAG : 1610
FWD3 : GATGTTTGGACTATGCCACGTGCAAGGTCTGCCTTCAGAAGGCCAATAGGACATTTAATGGCATATGCAG : 152

TNFAIP3 : CACTTGTTTCAAAAGGACTACAGAGCGCTCCTCGAATGGCAGCCCTGGTTTTGTACCCACGTGCCATCAG : 1680
FWD3 : CACTTGTTTCAAAAGGACTACAGAGCGCTCCTCGAATGGCAGCCCTGGTTTTGTACCCACGTGCCATCAG : 222

TNFAIP3 : AGGTCTACATCTGACCCATCCCAGATCCCACAGAGCCTCCTTCAGCATTCCTGCCAGCAAGCTCCCAACA : 1750
FWD3 : AGGTCTACATCTGACCCATCCCAGATCCCACAGAGCCTCCTTCAGCATTCCTGCCAGCAAGCTCCCAACA : 292

TNFAIP3 : ACAACTGCAATGAGCCGCCGTTAGCTCATGCCCCACGCGCTTTGGAGGAGAAGAAGGGAAGTAAGCTCTG : 1820
FWD3 : ACAACTGCAATGAGCCGCCGTTAGCTCATGCCCCACGCGCTTTGGAGGAGAAGAAGGGAAGTAAGCTCTG : 362

TNFAIP3 : CAGGAAGCCTGGCTGCAAGTTTTTTGGAACAGCTCAGAATGAGGGCTTTTGCACGTTGTGCTTCTTTGAA : 1890
FWD3 : CAGGAAGCCTGGCTGCAAGTTTTTTGGAACAGCTCAGAATGAGGGCTTTTGCACGTTGTGCTTCTTTGAA : 432

TNFAIP3 : TACAGGGAAAACCATGACAGTGCTTCACTACGCCATCAGAGGAGATCTCAGAGGAATTCCTCAGCAGCTG : 1960
FWD3 : TACAGGGAAAACCATGACAGTGCTTCACTACGCCATCAGAGGAGATCTCAGAGGAATTCCTCAGCAGCTG : 502

TNFAIP3 : GTCAACAGGGGAGCTCAGCTGCCACCTTCCACAATACCATGTCTTGCCAGCAGCAGAACTGTGGCACCCT : 2030
FWD3 : GTCAACAGGGGAGCTCAGCTGCCACCTTCCACAATACCACGTCTTGCCAGCAGCAGAACTGTGGCACCCT : 572

TNFAIP3 : GGGTAGCACCATGCTGGAGGGCTACTGCCAGAACTGTTTCATTAAAGCCCAGAGCCAGCGATTTCAAGAA : 2100
FWD3 : GGGTAGCACCATGCTGGAGGGCTACTGCCAGAACTGTTTCATTAAAGCCCAGAGCCAGCGATTTCAAGAA : 642

TNFAIP3 : GCCAGAAGGACAGAAGAACAGCTAGTGAGACAGCCTGAAAGAACAGGACAGCGCAGAGATCTACAGCGAG : 2170
FWD3 : GCCAGAAGGACAGAAGAACAGCTAGTGAGACAGCCTGAAAGAACAGGACAGCGCAGAGATCTACAGCGAG : 712

TNFAIP3 : CAGCACTGACTAGCCAGAAGAGGCAA.TGTACCGTGGCTTCATGTCGAAACAACTTGGCCTGCAGAAGCA : 2239
FWD3 : CAGCACTGACTAGCCAGAAGAGGCAAATGTACCGTGGCTTCATGTCGAAACAACTTGGCCTGCAGAAGCA : 782

TNFAIP3 : GTGAGCTGTGCCAGCAGTGCCAGCGCCTTGGCCAGTTTGCACAGCTGGGGGG.TCCCAGGGAGCCAGCCA : 2308
FWD3 : GTGAGCTGTGCCAGCAGTGCCAGCGCCTTGGCCAATTTGCACAGCTGGGGGGGTCCCAGGGAGCCAGCCA : 852

TNFAIP3 : CAGATGAGCCCCC.GAAGCAACGCTGTCGAGCCCCTGCTTGTGATCACTATGGTAATAACAAGTGCAA.T : 2376
FWD3 : CAGATGAGCCCCCCGAAGCAACGCTGTCGAGCCCCTGCTTGTGATCACTATGGTAATTACAAGTGCAAAT : 922

TNFAIP3 : GGTTATTGCAATGAATGCTACCAGTTCAAACAGATCTACGGCTAG : 2421
FWD3 : GGTTATTGCAATGAATGCTACCAGTTCCCCCAGATCCACGGCTAG : 967

TNIP2
TNIP2 : ATGCACCTGGCCGGGCTACCGCTCGCCCCGGAGGACGGAGAGAAGGGGCGGCGGTGCGAGCGGAGCTCGT : 70
FWD : ...................................................................... : -

TNIP2 : CCCCCGCCATGTGCTCGGTGAGCGAGGCCGGCAGCACCGACCCGCTGGTGGCCCGCTTCAGGCAGGTGGA : 140
FWD : ...................................................................... : -

TNIP2 : GGAAACGCTGGAAAAGCTGCACCGGGAGAACAGGAGCCTGAAGAGCAAAGTGCCCCGCTACAACGCGCTC : 210
FWD : ...................................................................... : -

TNIP2 : TGCACCCTGTACCACGAGTCCGCCCAGCAGCTGAAGCACCTCCAGCTGCAGCTCGCCGCCAAGGACGCCA : 280
FWD : ...................................................................... : -

TNIP2 : CGGTGCGGGAGCTGAGGGGCAGCCTGGCCCGGCAACAACAACAACAACAGCAGCAGCAGCAGGCGGGGGT : 350
FWD : ...................................................................... : -

TNIP2 : TGGCGAGGCGGGGGCCGAACCCGCCCGCTCGCTGGTGGAGAGCCTGCTGGAGCAGCTGGGGCAGGCCCGG : 420
FWD : ...................................................................... : -

TNIP2 : GAGCGACTGCGGGACAGCGAGCGGCACTCGGCGCTCAGAGTGGAGGCGCTGAGCCAGGAAGTGCAGAAGT : 490
FWD : ...................................................................... : -

TNIP2 : TGAATCAGCAGCTAGAGGAGAAAAACACAGAGATAGAGAAGATGATAAATCAGCCTCCATATGAAAAGGA : 560
FWD : CGCCATGGCGGCCGCGGGAATTCGATTATGCACCTGGCCGGGCTACCGCTCGCCCC......GGAGGACG : 64

TNIP2 : TGAATCAGCAGCTAGAGGAGAAAAACACAGAGATAGAGAAGATGATAAATCAGCCTCCATATGAAAAGGA : 560
REV : ..........................ATATAGGCACCTG.GCCGGGCTACCGCTCGCCCCGGAGGACGGA : 43


TNIP2 : GAGAGAAATCTTAAGGCTTCAGAAGAGCTTGGCAGAGCGAGAGAAGGCTCAGGCCACCAGTGATGTCCTG : 630
FWD : GAGAGAAATCTTAAGGCTTCAGAAGAGCTTGGCAGAGCGAGAGAAGGCTCAGGCCACCAGTGATGTCCTG : 134

TNIP2 : GAGAGAAATCTTAAGGCTTCAGAA.GAGCTTGGCAGAGCGAGAGAAGGCTCAGGCCACCAGTGATGTCCT : 629
REV : GAGGAAATCTTAAAGGCTTCAGAAAGAGCTTGGCAGAGCGAGAGAAGGCTCAGGCCACCAGTGATGTCCT : 113

TNIP2 : TGCCGGTCACTCACTGATGAAACTCACCAACTTCAGCGCAAATTAGCATCCACAGCAGAAATGTGTCAAC : 700
FWD : TGCCGGTCACTCACTGATGAAACTCACCAACTTCAGCGCAAATTAGCATCCACAGCAGAAATGTGTCAAC : 204

TNIP2 : GTGCCGGTCACTCACTGATGAAACTCACCAACTTCAGCGCAAATTAGCATCCACAGCAGAAATGTGTCAA : 699
REV : GTGCCGGTCACTCACTGATGAAACTCACCAACTTCAGCGCAAATTAGCATCCACAGCAGAAATGTGTCAA : 183

TNIP2 : ATCTGGCAAAGTGTTTAGAAGAGAAGCAAAGAAAAGAGAAGGGGAATTCGGATGACCAGATTGCTCCTGA : 770
FWD : ATCTGGCAAAGTGTTTAGAAGAGAAGCAAAGAAAAGAGAAGGGGAATTCGGATGACCAGATTGCTCCTGA : 274

TNIP2 : CATCTGGCAAAGTGTTTAGAAGAGAAGCAAAGAAAAGAGAAGGGGAATTCGGATGACCAGATTGCTCCTG : 769
REV : CATCTGGCAAAATGTTTAGAAGAGAAGCAAAGAAAAGAGAAGAGGAATTCGGATGACCAGATTGCTCCTG : 253

TNIP2 : AAGGTCTAATCAGTTACAGGTATTAGACAATGAAACTTCACTTCAAGCCCTTATCTGCAAACTACAAGAT : 840
FWD : AAGGTCTAATCAGTTACAGGTATTAGACAATGAAACTTCACTTCAAGCCCTTATCTGCAAACTACAAGAT : 344

TNIP2 : AAAGGTCTAATCAGTTACAGGTATTAGACAATGAAACTTCACTTCAAGCCCTTATCTGCAAACTACAAGA : 839
REV : AAAGGTCTAATCAGTTACAGGTATTAGACAATGAAACTTCACTTCAAGCCCTTATCTGCAAACTACAAGA : 323


TNIP2 : GAAAACAGAATGTTAAAACAAAAAGTAGCTCATGTGGAAGACTTAAATGCAAAATGGCAGAAGTACGATG : 910
FWD : GAAAACAGAATGTTAAAACAAAAAGTAGCTCATGTGGAAGACTTAAATGCAAAATGGCAGAAGTACGATG : 414

TNIP2 : TGAAAACAGAATGTTAAAACAAAAAGTAGCTCATGTGGAAGACTTAAATGCAAAATGGCAGAAGTACGAT : 909
REV : TGAAAACAGAATGTTAAAACAAAAAGTAGCTCATGTGGAAGACTTAAATGCAAAATGGCAGAAGTACGAT : 393

TNIP2 : CTAGTAGAGATGAGTATGTGAAGCGACTCCGCTTGCAGCTGAAAGAGCTGAAGTCAAAGCTGGAGCAGCA : 980
FWD : CTAGTAGAGATGAGTATGTGAAGCGACTCCACTTGCAGCTGAAAGAGCTGAAGTCACAGCTGGAGCAGCA : 484

TNIP2 : GCTAGTAGAGATGAGTATGTGAAGCGACTCCGCTTGCAGCTGAAAGAGCTGAAGTCAAAGCTGGAGCAGC : 979
REV : GCTAGTAGAGATGAGTATGTGAAGCGACTCCACTTGCAGCTGAAAGAGCTGAAGTCACAGCTGGAGCAGC : 463

TNIP2 : GCACAGCATACCTTCAGCACAAACCAATTCTGAGCTGATGCAAAAGGAGATACTCCGGCTAAACAAGCTA : 1050
FWD : GCACAGCATACCTTCAGCACAAACCAATTCTGAGCTGATGCAAAAGGAGATACTCCGGCTAAACAAGCTA : 554

TNIP2 : AGCACAGCATACCTTCAGCACAAACCAATTCTGAGCTGATGCAAAAGGAGATACTCCGGCTAAACAAGCT : 1049
REV : AGCACAGCATACCTTCAGCACAAACCAATTCTGAGCTGATGCAAAAGGAGATACTCCGGCTAAACAAGCT : 533

TNIP2 : CTGGAAGAAAAAATGAATGAGTGCAAAAAATCAAGGAGAGAATTGGAAGATGTGAAGAAGGCGAGAGAAG : 1120
FWD : CTGGAAGAAAAAATGAATGAGTGCAAAAAATCAAGGAGAGAATTGGAAGATGTGAAGAAGGCGAGAGAAG : 624

TNIP2 : ACTGGAAGAAAAAATGAATGAGTGCAAAAAATCAAGGAGAGAATTGGAAGATGTGAAGAAGGCGAGAGAA : 1119
REV : ACTGGAAGAAAAAATGAATGAGTGCAAAAAATCAAGGAGAGAATTGGAAGATGTGAAGAAGGCGAGAGAA : 603

TNIP2 : GAGACAGTGAGCGCATACAAATGCTGGAGCAACAGGTCCTAGTTTATAAAGATGATTTCACATCAGAGAG : 1190
FWD : GAGACAGTGAGCGCATACAAATGCTGGAGCAACAGGTCCTAGTTTATAAAGATGATTTCACATCAGAGAG : 694

TNIP2 : GGAGACAGTGAGCGCATACAAATGCTGGAGCAACAGGTCCTAGTTTATAAAGATGATTTCACATCAGAGA : 1189
REV : GGAGACAGTGAGCGCATACAAATGCTGGAGCAACAGGTCCTAGTTTATAAAGATGATTTCACATCAGAGA : 673


TNIP2 : ATCAGACAGAGAACGAGCACAGAGTAAAATACAAGAACTTCAGCTAGAAGTTTCATGTCTGCAACACCAG : 1260
FWD : ATCAGACAGAGAACGAGCACAGAGTAAAATACAAGAACTTCAGCTAGAAGTTTCATGTCTGCAACACCA. : 763

TNIP2 : GATCAGACAGAGAACGAGCACAGAGTAAAATACAAGAACTTCAGCTAGAAGTTTCATGTCTGCAACACCA : 1259
REV : GATCAGACAGAGAACGAGCACAGAGTAAAATACAAGAACTTCAGCTAGAAGTTTCATGTCTGCAACACCA : 743

TNIP2 : CTAGCAAGAAGACAAGAAGGCAAGAAGGACTCCAGAGACACAAGTAGTCGTTTCAGAGTTCACACTGGTA : 1330
FWD : ...................................................................... : -
TNIP2 : GCTAGCAAGAAGACAAGAAGGCAAGAAGGACTCCAGAGACACAAGTAGTCGTTTCAGAGTTCACACTGGT : 1329
REV : GCTAGCAAGAAGACAAGAAGGCAAGAAGGACTCCAGAGACACAAGTAGTCGTTTCAGAGTTCACACTGGT : 813

TNIP2 : AACCAAAATCATATGCATGTACAGACAAAAGTGGAACACCTACGAGGCAGCA.CCCAGGCCAACCAGGCG : 1398
REV : AACCAAAATCATATGCATGTACAGACAAAAGTGGAACACCTACGAGGCAGCAGCCCAGGCCAACCAGGCG : 883

TNIP2 : CAAGAAGAACAGCCTCACAGTCTGAACAGACTTCCCCACCTGCAGACAATGGAAACTCAGGATCAGAGGG : 1468
REV : CAAGAAGAACAGCCTCACAGTCTGAACAGACTTCCCCACCTGCAGACAATGGAAACTCAGGATCAGAGGG : 953

TNIP2 : CAGGGCACAGGGTGAACTTAGATGTCCTCACTGTATGA : 1506
REV : CAGGGCACAGGGTGAACTTAGATGTCCTCACTGTATGA : 991

TOLLIP
TOLLIP : ATGGCGACCACCGTCAGTACCCAACGGGGCCCGGTGTATGTTGGTGAGCTTCCTCAAGACTTTCTTCGTA : 70
FWD : ATGGCGACCACCGTCAGTACCCAACGGGGCCCGGTGTATGTTGGTGAGCTTCCTCAAGACTTTCTTCGTA : 70

TOLLIP : TTACTCCAACCCAGCAGCAACAGCAGATCCAACTGGATGCCCAGGCAGCTCAGCAGCTGCAGTATGGAGG : 140
FWD : TTACTCCAACCCAGCAGCAACAGCAGATCCAACTGGATGCCCAGGCAGCTCAGCAGCTGCAGTATGGAGG : 140

TOLLIP : ACCAATGAGTACAGTAGGCAGACTGAGCATTACCGTAGTACAGGCAAAATTGGCAAAGAACTATGGAATG : 210
FWD : ACCAATGAGTACAGTAGGCAGACTGAGCATTACCGTAGTACAGGCAAAATTGGCAAAGAACTATGGAATG : 210

TOLLIP : ACCCGCATGGATCCCTACTGCCGAATACGGCTGGGGTATGCTGTGTATGAAACCCCCACAGCACATAATG : 280
FWD : ACCCGCATGGATCCCTACTGCCGAATACGGCTGGGGTATGCTGTGTATGAAACCCCCACAGCACATAATG : 280

TOLLIP : GAGCCAAGAACCCTCGCTGGAACAAAGTTATTCAGTGCACTGTTCCTCCGGGTGTGGACTCTTTTTACCT : 350
FWD : GAGCCAAGAACCCTCGCTGGAACAAAGTTATTCAGTGCACTGTTCCTCCGGGTGTGGACTCTTTTTACCT : 350

TOLLIP : AGAGATATTTGATGAGCGAGCCTTTTCAATGGATGATCGCATTGCTTGGACACACATTACAATTCCTGAA : 420
FWD : AGAGATATTTGATGAGCGAGCCTTTTCAATGGATGATCGCATTGCTTGGACACACATTACAATTCCTGAA : 420

TOLLIP : TCTCTAAAACAAGGAAATGTGGAGGATGAATGGTATAGCCTGAGTGGAAGGCAGGGTGATGACAAAGAAG : 490
REV : TCTCTAAAACAAGGAAATGTGGAGGATGAATGGAATAGCCTGAGTGGAAGGCAGGGTGATGACAAAGAAG : 113

TOLLIP : GAATGATTAACCTGGTGATGTCATACACGTCTTTGCCAGCTGCCATGATGATGCAGCCCCAGCCAGTGGT : 560
REV : GAATGATTAACCTGGTGATGTCATACACGTCTTTGCCAGCTGCCATGATGATGCAGCCCCAGCCAGTGGT : 183

TOLLIP : TCTGATGCCCACTGTATACCAGCAAGGAGTGGGATATGTGCCTATAGCAGGAATGCCTGCAGTCTGTAAT : 630
REV : TCTGATGCCCACTGTATACCAGCAAGGAGTGGGATATGTGCCTATAGCAGGAATGCCTGCAGTCTGTAAT : 253

TOLLIP : CCTGACATGGTACCTGTGGCAATACCACCTCCTGCAGTCAATCCTCAGCACCTGTGTAACGAAGAGGACC : 700
REV : CCTGACATGGTACCTGTGGCAATACCACCTCCTGCAGTCAATCCTCAGCACCTGTGTAACGAAGAGGACC : 323

TOLLIP : TGAAATCTATCCAGGACATGTTTCCTAACATGGACAGGGAAGTAATCCGCTCAGTGCTAGAAGCTCAGAG : 770
REV : TGAAATCTATCCAGGACATGTTTCCCAACATGGACAGGGAAGTAATCCGCTCAGTGCTAGAAGCTCAGAG : 393

TOLLIP : AGGGAACAAGAATGCAGCTATCAACTCCTTGCTTCAGATGACTGAAGAATCATAG : 825
REV : AGGGAGCAAGAATGCAGCTATCAACTCCTTGCTTCAGATGACTGAAGAATCATAG : 448

TRAF3IP2
TRAF3IP2 : ATGGCTTCTGTGTCAGGCACTTTTGTGAGCCGAAGCATACCGGTGGAAGTGGATGAATCCATGCTGTATT : 70
FWD : ATGGCTTCTGTGTCAGGCACTTTTGTGAGCCGAAGCATACCGGTGGAAGTGGATGAATCCATGCTGTATT : 70

TRAF3IP2 : CCCCATTTCCTGAACCTGCCTTGGAAGAGGCTTCACAGCCTTCTGTAGAGCATGTGGAGGATGAGGAGCG : 140
FWD : CCCCATTTCCTGAACCTGCCTTGGAAGAGGCTTCACAGCCTTCTGTAGAGCATGTGGAGGATGAGGAGCG : 140

TRAF3IP2 : CCAGAGACCACGTGCCAATCCAGCACATCTCTGCAGAGCGCTGGCACATCACCAGCTGGCCACGGAGCCG : 210
FWD : CCAGAGACCACGTGCCAATCCAGCACATCTCTGCAGAGCGCTGGCACATCACCAGCTGGCCACGGAGCCG : 210

TRAF3IP2 : GACAGCAGGCCGTGGTCAGAATATACCGATCCTGATGGATGTGGGTGCTTCTGTTCCCCTAGTGAATCAG : 280
FWD : GACAGCAGGCCGTGGTCAGAATATACCGATCCTGATGGATGTGAGTGCTTCTGTTCCCCTAGTGAATCAG : 280

TRAF3IP2 : TACTGGAATTAGTTCACTCCAAGATGCATCTTTTAACTATGGACAAATCAGGCATGCAGCCAGGACCACT : 350
FWD : TACTGGAATTAGTTCACTCCAAGATGCATCTTTTAACTATGGACAAATCAGGCATGCAGCCAGGACCACT : 350

TRAF3IP2 : GCTTTCAGCTGATACCAACTCCAGTAAGTCAGAGCAGAGCTTGGATGATGCTCCTGAGGACTCATTGGAG : 420
FWD : GCTTTCAGCTGATACCAACTCCAGTAAGTCAGAGCAGAGCTTGGATGATGCTCCTGAGGACTCATTGGAG : 420

TRAF3IP2 : GAGAACAACAAAGGGAGTTCATGGTCACGGCAGCCTTCAGGAAACAAACTCCCAGCGGAGCTGGCAACTG : 490
FWD : GAGAACAACAAAGGGAGTTCATGGTCACGGCAGCCTTCAGGAAACAAACTCCCAGCGGAGCTGGCAACTG : 490

TRAF3IP2 : AGGACACGGGTTACAACTCCCAGTCCCAAGACATCATGGGTGTCAGGCACCTGGAGCTCCCCTTACCACT : 560
FWD : AGGACACGGGTTACAACTCCCAGTCCCAAGACATCATGGGTGTCAGGCACCTGGAGCTCCCCTTACCACT : 560

TRAF3IP2 : TGTGTCCGTGGTGAACCCCCAGGACCTCCCAGGACCGCTGATCTCCAGAGAGTTTTTTGGACCTGAGCCT : 630
FWD : TGTGTCCGTGGTGAACCCCCAGGACCTCCCAGGATCGCTGATCTCCAGAGA................... : 611

TRAF3IP2 : CAGCAGCACCCCAGGTGCCAGCACTTGCCCCATCCCAACCCCTCTGCGCAAGCCCATGGCTTCTGTGGGC : 700
FWD : ...................................................................... : -

TRAF3IP2 : ACCACTATCTGGCAGAGCAGCACCTGCACGGCCCATATGGCAGAGCTCCTTACCAGCATTTTGCACATCC : 770
FWD2 : .................................................TTACCAGCATTTTGCACATCC : 21

TRAF3IP2 : ATCACAACCGCTCCCTCCTGTTCCCGGACCCTGCATGAGAGTTATCCGCCCAGCCCAGCAAGTCATCCCC : 840
FWD2 : ATCACAACCGCTCCCTCCTGTTCCCGGACCCTGCATGAGAGTTATCCGCCCAGCCCAGCAAGTCATCCCC : 91

TRAF3IP2 : AATTACTCCAACCTTCGTGCTCCCAAGGGCACCACCGAGCGACCTCCCCAGAGGCTGTGCTCCTCCCCTG : 910
FWD2 : AATTACTCCAACCTTCGTGCTCCCAAGGGCACCACCGAGCGACCTCCCCAGAGGCTGTGCTCCTCCCCTG : 161

TRAF3IP2 : GTCCTCCTCGATTCCCAAACCAGTTGTACAACCAGCTACCTAACGGCCAGCTGTCCCAAAAGGCATGTGG : 980
FWD2 : GTCCTCCTCGATTCCCAAACCAGTTGTACAACCAGCTACCTAACGGCCAGCTGTCCCAAAAGGCATGTGG : 231

TRAF3IP2 : CCCAGATGAAGCATGTTGTTGTCCTTCTGACAATTTTCCATCTCCAGCTGCTGTCCCAAGACCTCTCAGT : 1050
FWD2 : CCCAGATGAAGCATGTTGTTGTCCTTCTGACAATTTTCCATCTCCAGCTGCTGTCCCAAGACCTCTCAGT : 301

TRAF3IP2 : AACCCTGCAGCCAAGGGAACTCTGAGAACCAGCAATTTGCCGGAAGAGTTGCGCAAGGTCTTTATAACCT : 1120
FWD2 : AACCCTGCAGCCAAGGGAACTCTGAGAACCAGCAATTTGCCGGAAGAGTTGCGCAAGGTCTTTATAACCT : 371

TRAF3IP2 : ATTCTGTGGACACAGCGGTGGAGGTCATGAAATTTGTGAACTTCCTGCTTGTAAATGGATTTCAGACTGC : 1190
FWD2 : ATTCTGTGGACACAGCGGTGGAGGTCATGAAATTTGTGAACTTCCTGCTTGTAAATGGATTTCAGACTGC : 441

TRAF3IP2 : TATCGATATATTTGAAGACACAGTACGAGGTATTGACATCATAAAGTGGATGGAACGCTACCTAGGTGAT : 1260
FWD2 : TATCGATATATTTGAAGACACAGTACGAGGTATTGACATCATAAAGTGGATGGAACGCTACCTAGGTGAT : 511

TRAF3IP2 : AAGACGGTGATGATAATCATAGCAATCAGTCCAAAATACAAACAGGATGTGGAAGGGGCTGAGTCCCAGC : 1330
FWD2 : AAGACGGTGATGATAATCATAGCAATCAGTCCAAAATACAAACAGGATGTGGAAGGGGCTGAGTCCCAGC : 581

TRAF3IP2 : TGGACAGGGATGAACACGGCTTACATACTAAATACATCCACAGGATGATGCAGATTGAGTTCATACAACA : 1400
REV : TGGACAGGGATGAACACGGCTTACATACTAAATACATCCACAGGATGATGCAGATTGAGTTCATACAACA : 111

TRAF3IP2 : AGGGAGCATGAACTTCAGATTCATCCCCGTGCTTTTCCCAAATGCCAAAAAGGAGCACGTGCCTACCTGG : 1470
REV : AGGGAGCATGAACTTCAGATTCATCCCCGTGCTTTTCCCAAATGCCAAAAAGGAGCACGTGCCTACCTGG : 181

TRAF3IP2 : TTGCAGAACACTCACATTTATAACTGGCCAAAGAACAAGAAGAACATCCTCCTGCGGCTACTGAGGGAGG : 1540
REV : TTGCAGAACACTCACATTTATAACTGGCCAAAGAACAAGAAGAACATCCTCCTGCGGCTACTGAGGGAGG : 251

TRAF3IP2 : AGGAATATGTTGCTCCTCCAATAGGACCTTTACCAACACTCCAGGTTGTGCCGTTATGA : 1599
REV : AGGAATATGTTGCTCCTCCAATAGGACCTTAACCAACACTCCAGGTTGTGCCGTTATGA : 310

UPP1
UPP1 : ATGGCTCCTGGTGTCTCGAATGAGAAGAAAACGGAAGATGAACAGTCTTCAAGAGAAAATTCTATCCATC : 70
FWD : ATGGCTCCTGGTGTCTCGAATGAGAAGAAAAGGGAAGATGAACAGTCTTCAAGAGAAAATTCTATCCATC : 70
REV : ...................................................................... : -

UPP1 : TGTGCAACCCTCACCTGGAAAAAATGAAAGAAGACATCCTATACCATTTTGCTCTTGGGACCGGTACCCA : 140
FWD : TGTGCAACCCTCACCTGGAAAAAATGAAAGAAGACATCCTATACCATTTTGCTCTTGGGACCGGTACCCA : 140
REV : ...................................................................... : -

UPP1 : TGATTTTCCTGCACTGTTTGGAGATGTAAAGTTTGTGTGTGTAGGAGGAAGTCCTTCACGGATGAAAGCT : 210
FWD : TGATTTTCCTGCACTGTTTGGAGATGTAAAGTTTGTGTGTGTAGGAGGAAGTCCTTCACGGATGAAAGCT : 210
REV : ........................................GTAGGAGGAAGTCCTTCACGGATGAAAGCT : 30

UPP1 : TTTATTGCCTACATAGCTGAAGAACTTGGACTTGGGAGCTCTGGTGGTGACTACCCCAACATCTGTGCGG : 280
FWD : TTTATTGCCTACATAGCTGAAGAACTTGGACTTGGGAGCTCTGGTGGTGACTACCCCAACATCTGTGCGG : 280
REV : TTTATTGCCTACATAGCTGAAGAACTTGGACTTGGGAGCTCTGGTGGTGACTACCCCAACATCTGTGCGG : 100

UPP1 : GAACTGACCGTTACGCCATGTACAAAGTGGGACCCGTTCTGTCAGTCAGTCACGGTATGGGCATTCCTTC : 350
FWD : GAACTGACCGTTACGCCATGTACAAAGTGGGACCCGTTCTGTCAGTCAGTCACGGTATGGGCATTCCTTC : 350
REV : GAACTGACCGTTACGCCATGTACAAAGTGGGACCCGTTCTGTCAGTCAGTCACGGTATGGGCATTCCTTC : 170

UPP1 : TATTTCAATCATGTTGCACGAGCTGATCAAACTGTTGTATCATGCCAAGTGTTCCAACATAACCATTATT : 420
FWD : TATTTCAATCATGTTGCACGAGCTGATCAAACTGTTGCATCATGCCAAGTGTTCCAACATAACCATTATT : 420
REV : TATTTCAATCATGTTGCACGAGCTGATCAAACTGTTGCATCATGCCAAGTGTTCCAACATAACCATTATT : 240

UPP1 : CGCATTGGCACCTCGGGTGGAATAGGTCTGGAACCAGGCTCAGTGGTTATAACCAGGCAGTCTGTAGATG : 490
FWD : CGCATTGGCACCTCGGGTGGAATAGGTCTGGAACCAGGCTCAGTGGTTATAACCAGGCAGTCTGTAGATG : 490
REV : CGCATTGGCACCTCGGGTGGAATAGGTCTGGAACCAGGCTCAGTGGTTATAACCAGGCAGTCTGTAGATG : 310

UPP1 : CCACCTTCAAACCTCAGTTTGAACAGGTTGTTCTGGGAAAAACTATAATTCGCAGCACAAATCTAGATGA : 560
FWD : CCACCTTCAAACCTCAGTTTGAACAGGTTGTTCTGGGAAAAACTATAATTCGCAGCACAAATCTAGATGA : 560
REV : CCACCTTCAAACCTCAGTTTGAACAGGTTGTTCTGGGAAAAACTATAATTCGCAGCACAAATCTAGATGA : 380

UPP1 : AGAGCTGGCTAAAGAGCTGCTACAGTGCAGTAAAGAAATCAATCAGTTCAATACTGTCATTGGAAACACT : 630
FWD : AGAGCTGGCTAAAGAGCTGCTACAGTGCAGTAAAGAAATCAATCAGTTCAATACTGTCATTGGAAACACT : 630
REV : AGAGCTGGCTAAAGAGCTGCTACAGTGCAGTAAAGAAATCAATCAGTTCAATACTGTCATTGGAAACACT : 450

UPP1 : ATGTGCACTTTGGATTTTTATGAAGGACAGGGCAGGTTGGATGGTGCAATCTGCTTGTATAATGAAGAAG : 700
FWD : ATGTGCACTTTGGATTTTTATGAAGGACAGGGCAGGTTGGATGGTGCAATCTGCTTGTATAATGAAGAAG : 700
REV : ATGTGCACTTTGGATTTTTATGAAGGACAGGGCAGGTTGGATGGTGCAATCTGCTTGTATAATGAAGAAG : 520

UPP1 : AAAAACTGCAATATTTGAAGGAAGCTTACGATTCCGGTGTCAGAAACATTGAGATGGAATCTTCTGTCTT : 770
FWD : AAAAACTGCAATATTTGAAGGAAGCTTACGATTCCGGTGTCAGAAACATTGAGATGGAATCTTCTGTCTT : 770
REV : AAAAACTGCAATATTTGAAGGAAGCTTACGATTCCGGTGTCAGAAACATTGAGATGGAATCTTCTGTCTT : 590

UPP1 : TGCTGCAATGTGCAATCTCAGTGGTGTCAGAGCTGCTGTAGTGTGTGTCACTCTCCTGAATCGGCTTGAA : 840
FWD : TGCTGCAATGTG.......................................................... : 782
REV : TGCTGCAATGTGCAATCTCAGTGGTGTCAGAGCTGCTGTAGTGTGTGTCACTCTCCTGAATCGGCTTGAA : 660

UPP1 : GGGGACCAGATCAGTAGCTCACATGATATACTTGTGGAGTATCAGCAGAGACCGCAGAAATTAGTGGGAT : 910
FWD : ...................................................................... : -
REV : GGGGACCAGATCAGTAGCTCACACGATATACTTGTGGAGTATCAGCAGAGACCGCAGAAATTAGTGGGAT : 730

UPP1 : ATTTCATTAAGAAAAGTCTTGGGAAAGTATAA : 942
FWD : ................................ : -
REV : ATTTCATTAAGAAAAGTCTTGGGAAAGTATAA : 762

WDR24

WDR24 : ATGGATGAGAACCTGCTGGCCACCGCCGCCACCAATGGCGTGGTGGTCACCTGGAACCTGGGCAAGCCGT : 70
FWD : ATGGATGAGAACCTGCTGGCCACCGCCGCCACCAATGGCGTGGTGGTCACCTGGAACCTGGGCAAGCCGT : 70

WDR24 : CCCGCAATAAGCAGGACCAGCTGTTCACCGAGCACAAGCGCACTGTCAACAAGGTCTGCTTCCACCCCAC : 140
FWD : CCCGCAATAAGCAGGACCAGCTGTTCACCGAGCACAAGCGCACTGTCAACAAGGTCTGCTTCCACCCCAC : 140

WDR24 : CGAGGTGTACATGCTGCTCAGCGGCTCCCAGGATGGCTACATGAAGTGCTTTGACCTGCGCAAGAAGGAC : 210
FWD : CGAGGTGTACATGCTGCTCAGCGGCTCCCAGGATGGCTACATGAAGTGCTTTGACCTGCGCAAGAAGGAC : 210

WDR24 : TCTGTCAGCACCTTCTCTGGCCAGTCGGAGAGTGTGCGTGATGTGCAGTTCAGCATCCGTGACTACTTCA : 280
FWD : TCTGTCAGCACCTTCTCTGGCCAGTCGGAGAGTGTGCGTGATGTGCAGTTCAGCATCCGTGACTACTTCA : 280

WDR24 : CCTTCGCTGCCACCTTTGAGAATGGCAACGTGCAGCTGTGGGACATCCGCCGGCCCGACCGCTATGAGAG : 350
FWD : CCTTCGCTGCCACCTTTGAGAATGGCAACGTGCAGCTGTGGGACATCCGCCGGCCCGACCGCTATGAGAG : 350

WDR24 : GATGTTCACAGCCCACAACGGCCCCGTCTTCTGCTGTGACTGGCACCCCGAGGACCGGGGCTGGCTGGCA : 420
FWD : GATGTTCACAGCCCACAACGGCCCCGTCTTCTGCTGTGACTGGCACCCCGAGGACCGGGGCTGGCTGGCA : 420

WDR24 : ACAGGTGGCCGGGATAAGATGGTGAAGGTTTGGGACATGAACACCACGCGGGCAAAGGAAATCTACTGCG : 490
FWD : ACAGGTGGCCGGGATAAGATGGTGAAGGTTTGGGACATGAACACCACGCGGGCAAAGGAAATCTACTGCG : 490

WDR24 : TGCAGACCATCGCCTCCGTAGCCCGGGTGAAGTGGCGGCCGGAGTGCAAGCACCACATTGCCACCTGCTC : 560
FWD : TGCAGACCATCGCCTCCGTAGCCCGGGTGAAGTGGCGGCCGGAGTGCAAGCACCACATTGCCACCTGCTC : 560

WDR24 : CATGATGGTGGACCACAACATCTACGTCTGGGACGTGCGGCGGCCCTTCATCCCCTCTGCCATGTTTGAG : 630
FWD : CATGATGGTGGACCACAACATCTACGTCTGGGACGTGCGGCGGCCCTTCATCCCCTCTGCCATGTTTGAG : 630

WDR24 : GAGCACAAGGATGTCACCACGGGCATCGTGTGGCGTCACCTCCATGACCCCTACTTCCTCCTGTCCGGCT : 700
FWD : GAGCACAAGGATGTCACCACGGGCATCGTGTGGCGTCACCTCCACGACCCCTACTTCCTCCTGTCCGGCT : 700

WDR24 : CCAAGGACAGCACCCTCTACCAGCACATCTTCAAGGACGCCAGCCAGCCCATCGACCGGGCCAACCCTGA : 770
FWD : CCAAGGACAGTACCCTCTACCAGCACATCTTCAAGGACGCCAGCCAGCCCATCGACCGGGCCAACCCTGA : 770

WDR24 : GGGGCTGTGCTACAGCCTTTATGGAGACCTGGCCTTCGCAGCCAAGGAAAGTCTCATCTCCTCCGACTCC : 840
FWD : GGGGCTGTGCTACAGCCTTTATGGAGACCTGGCCTTCGCAGCCAAGGAAAGTCTCATCTCCTCCGACTCC : 840

WDR24 : AACCGCAAGCCCTACATCGGGGACCGCCGCCACCCCATCTTTTTCAAGCGCAAGCTGGACCCCACGGAGC : 910
FWD : AACCGCAAGCCCTACATCGGGGACCGCCGCCACCCCAT................................ : 878

WDR24 : AGTTTGAGTACATCTCCTCCTCCAGTGCCCTCAGTGTCTTCGAGACGGACGTGGAGAGTGGCAGCATGGA : 980
REV : ...................................................................... : -

WDR24 : CTGGTTTGTGCACACTGCCAAGCAGTATGCACTGGCTGGCAGGCCCCTGGCTGAGCTCTGCGACCACAAT : 1050
REV : ...................................................................... : -

WDR24 : GCCAAGGTGGCCAAGGGCTTGGACCGCAACCAGGTGGCTCAAACGTGGACGATGCTCCGGATCATCTACT : 1120
REV : ...................................................................... : -

WDR24 : CCAGCCTTGGCACCGTGTCATCCACCAACCTCAATCACAGCATGGGGAAGGGCAGCACCACTCTCCCGCT : 1190
REV : ...................................................................... : -

WDR24 : CATGAACAGCTTTAACCTGAAGGACATCCCCTCC.GGGCTGGGCAGCGAGTCGAGGTTGGATCGCAGCAA : 1259
REV : ...................AAGGACATCCCCTCCCGGGCTGGGCAGCGAGTCGAGGTTGGATCGCAGCAA : 51


WDR24 : AGGAGAAAGCCGCACGGAAAATATCCTCATGGATTCCTCCTCCACCCTGATCAACAACGAGGACAACGAG : 1329
REV : AGGAGAAAGCCGCACCGAAAATATCCTCATGGATTCCTCCTCCACCCTGATCAACAACGAGGACAACGAG : 121

WDR24 : GAGACAGAGGGCAGCGATGTCCCTGCAGACTACCTGCTGGGAGACGTGGAGGCGGATGAAGATGACCTGT : 1399
REV : GAGACAGAGGGCAGCGATGTCCCTGCAGACTACCTGCTGGGAGACGTGGAGGCGGATGAAGATGACCTGT : 191

WDR24 : ATATGATGGACCACGAGAACCCACACGCAGAAGAGCAGGAGTATAGCCTTCCCCAGGAAGCCTTCCCCCT : 1469
REV : ATATGATGGACCACGAGAACCCACACGCAGAAGAGCAGGAGTATAGCCTTCCCCAGGAAGCCTTCCCCCT : 261

WDR24 : GCGCCACGAAATCGTGGACAACCCGTCAGCCCTGGACCACCTGCAGGACAAGGCTGACTCCCCCCACGTC : 1539
REV : GCGCCACGAAATCGTGGACAACCCGTCAGCCCTGGACCACCTGCAGGACAAGGCTGACTCCCCCCACGTC : 331

WDR24 : AGTGGCAATGAGGCCGAGACGGTGTCCCTGACGCCCGTGGAGTCCTTCTCCCTCATCTCCATCTCCCACT : 1609
REV : AGTGGCAATGAGGCCGAGACGGTGTCCCTGACGCCCGTGGAGTCCTTCTCCCTCATCTCCATCTCCCACT : 401

WDR24 : CTCTCTATGAGAACCGCCTGCCCTCCGACTTCTTCAACCCCATTGTGCGTGACACGCTGCTCTTCTACGC : 1679
REV : CGCTCTATGAGAACCGCCTGCCCTCCGACTTCTTCAACCCCATTGTGCGTGACACGCTGCTCTTCTACGC : 471

WDR24 : CGAGCAGGGGGACGTGCAGACAGCTGTGTCTGTGCTCATTGTGCTGGGAGACCGAATCCGCAAGGAGATC : 1749
REV : CGAACAAGGGGACGTGCAGACAGCTGTGTCTGTGCTCATTGTGCTGGGAGACCGAATCCGCAAGGAGATC : 541

WDR24 : GATGAGCAGACCCAGGAGCACTGGTACACCTCCTACATCGACCTGCTGCAGCGCTTCCAGCTCTGGAACA : 1819
REV : GATGAGCAGACCCAGGAGCACTGGTACACCTCCTACATCGACCTGCTGCAGCGCTTCCAGCTCTGGAACA : 611

WDR24 : TCTCCAACGAGGTGATCAAGCTGAGCACGTGCCGCGCCATCAACTGCCTCAACCAAGCCTCCACCACCCT : 1889
REV : TCTCCAACGAGGTGATCAAGCTGAGCACGTGCCGCGCCATCAACTGCCTCAACCAAGCCTCCACCACCCT : 681

WDR24 : CCACATCAACTGCAGCAACTGCAAGCGGCCCATGAGCAACAGGGGCTGGATCTGTGACAGGTGTCGGCAG : 1959
REV : CCACATCAACTGCAGCAACTGCAAGCGGCCCATGAGCAACAGGGGCTGGATCTGTGACAGGTGTCGGCAG : 751

WDR24 : TGTGCCAGCATGTGCGCCGTGTGCCACCACGTGGTGAAGGGGCTCTTTGTCTGGTGCCAGGGCTGCAGCC : 2029
REV : TGTGCCAGCATGTGCGCCGTGTGCCACCACGTGGTGAAGGGGCTCTTTGTCTGGTGCCAGGGCTGCAGCC : 821

WDR24 : ACGGCGGCCACCTGCAGCACATCATGAAGTGGTTGGAGACCAGCTCACACTGCCCGGCGGGCTGCGGCCA : 2099
REV : ACGGCGGCCACCTGCAGCACATCATGAAGTGGTTGGAGACCAGCTCACACTGCCCGGCGGGCTGCGGCCA : 891

WDR24 : CCTCTGCGAGTACACCTGA : 2118
REV : CCTCTGCGAGTACACCTGA : 910

Sequences of genes previously cloned with highlighted qPCR primers aligning sites:

CCL19

>gi|694016491:541-840 Gallus gallus chemokine (C-C motif) ligand 19 (CCL19), mRNA

ATGCAGCGGCTGCACGTTCTCTGCCTTAGTCTCCTGGTGCTGAGATGTGTCCTGCATGTGTATGCTGGCAACAACGTCCTCGACTGCTGCCTGCGGACGAGCGAGAAGCCCATCCCATGGCGGATAGTGCAGGACTACAGGATGCAGCTGGTGCAGGATGGCTGCGACATCCCTGCCACCGTGTTCATCACTGCAAAGGGCAAGCGTCTCTGTGCCCCACCCCAAGCTCCGTGGGTGCTGCGCCTCCGAGAGAAGCTGGACACCAGCTCTGCCAGGAAGGTCCCAAATCAAGGCAATTAG

CCL20

>gi|402744368:65-367 Gallus gallus chemokine (C-C motif) ligand 20 (CCL20), mRNA

ATGCCTGGCTTGAGCACCAAGAGTTTGATTTTGGCTTCTCTGCTGGGACTGCTGTTGCTGCTGCTGTGCAGTACCTCCCAGGCACAAAGCAACCAAGATTGCTGTCTGTCTTACAGCAAAGTCCGTCTGCCTCGGAAGGTCATTAAGGGCTTTACTGAACAGCTGTCTGGTGAAGTCTGTGATATTGATGCCATCATTTTCCACACCGTCAGGGGACTGAAAGCCTGCGTAAATCCTAAGGAAGACTGGGTGAAGAAGCATCTTCTTTTCCTGAGCCAGAAGCTCAAGAGGATGTCAATGTGA

CCLi3

>gi|45382626:54-323 Gallus gallus chemokine (C-C motif) ligand 4 (CCL4), mRNA

ATGAAGCTCTCTGCAGTTGTTCTCGCTCTTCTCATCGCATCCTTCTGCTCCCGAGCCTCCTCTGCCCCAGTGGGACCCGACGTCCCGACCTGCTGCACCACTTACATAACACACAAGATCCCGCGGAACCTCATCCAGAGGCACTACAGCACCAGCACCAGCTGCTCAAAGCCTGCCATCATCTTCATCACAAAGAAGGAGCGCGAAGTCTGCGCCAACCCCAGCGACCCGTGGGTGCAGAGATACCTGCAGAGCGTCAAGCGGGACTGA

CCLi4

>gi|113951680|ref|NM_001045832.1| Gallus gallus chemokine (C-C motif) ligand 5 (CCL5), mRNA

ATGATGACTGCCGTAGCTGTGTCCCTCTCCATCCTCCTGGTTGCCGCCCTCTTCCCTCAAGCCTCTTCATCTCCGTTTGGGGCTGATACAACCGTGTGCTGCTTCAACTATAGTGTACGGAAGCTGCCCCAGAATCATGTGAAGGATTATTTCTACACCAGCAGCAAATGCCCACAGGCAGCAGTTGTGTTCATCACCAGGAAGGGGCGGCAGGTCTGCGCAAACCCTGACGCCCGGTGGGTGAAGGAATACATCAACTTCCTGGAGCTGCAGTGA

CSF1

>gi|300807185|ref|NM_001193295.1| Gallus gallus colony-stimulating factor 1 (CSF1), mRNA

ATGCCCCGCCTCGGATCCCAGGTGTCCCTGTTCCGCTGCACCCTGCTCTCGTCCCTCCTCCTCGTCTGCAGCATCCATGAGACGGAGCAGAACAGCTACTGCCAGCAGATCATCACCGAGCGGCACCTGGACCACCTGCAGGAGCTGGCGGACACGCAGATGCAGCAGCCGGGCACAGTGTCCTTCAGATTCATCAGCAAGATGCGGCTGAGCGACTCTGTCTGCTACGTGAAAGCCGCCTTCCCTTTGCTGGGCACCATCCTGAACAGGACGACGTTCAAGGAGAACTCAACAAACGCCAACAAGATGAAGACGGTGCGCAAGATGTACGAAAACATCGATGAGGACGTGGACCCCTGCATCAGGGACGAGGATGACGAGGAGCACGCGCTGTCCGAAATGTGCTTTGAGGAGTTCACCACGTCCCCCTACGAGATGCTGGTGCTGGTGAGGCAGTTCTTCCAGGACATCAAACAGCTGCTGCAGAACAAGGAGACCTTCGAGAAGGACTGCAGCCAGGTGTACCGCAGTGCGTGCGCGGGGCCCCGGCAGCACAGCTCCTCCCCAGAGCAGCGCAGGAAGGAGCCGCCAGCCGCCAGCGGGGGGCACCGGGAGCTCCTGGTGTACGTCACGGTGGCCAGCGTGGTGGCCGTGCTGCTGGCCATGGGCGGGCTGCTCTTCTACAAGTATAAGTCCAAGGTCCTGCAGCGGGGAGCAGCGCTAAAAGAGGGGGGCTGCGACCCCGAGGAGCCGGAGAGCAGGGCGCTGCAGGGAGCGCAGGGCTGCGCGGAGCTGGAGACGCAGGAGCTGTGA

CXCL13L2

>gi|513182487:1452-1754 PREDICTED: Gallus gallus chemokine (CXCL13L2), transcript variant X3, mRNA

ATGGCAGTGCGGGCAGCCCTGCTGCTGGGGCTGCTGCTGGTGGTTCTGTGCCCTGGGGATGCAGCCATCCTGGAAGCCAACGGCAACCTGAACTGCCGCTGCGCCAAGACCACAACTGCCTTCATTCCCTTGCGGAAATATGAGAGCGTCGAGGTGCGGCCGGTGGGGAGCAGCTGCCGGCGCCTGGAGGTGTTGATTAAACTAAAAACCCTCGAGAGGATCTGTGTGGATCCCAACACCCCCTGGGTGAGGAAGCTCCTGCAGGACCTCCCGAAGCTGAAGAAGAAAGCTGCTCCCCAGTGA

CXCLi1 – as predicted in Ensembl

ATGCGGGCGCTGCAGGCGGCGCTGGCCCTGGGCCTGCTGTTGAGCAGCCTGCTGCCGGGGGACGGTCTGTCGCTGGAGAGCCTGCTGACCAACAAGAGGTGCAAGTGTGTCAAAGTGACTGCCCAGATCATCAGCCTGGGGCTCATTCTTGCCATTGACGTGATGCCGCCGGGTATTCACTGCCGGAGGAAAGAGATCATCCTCACCCTGAAGAGGAACAAGAAGGTGTGTGTGGCCCCCGAGGCGCCCTGGATCCAGCTGCTCATCCACAAGCTGACACAGACGGATGTCAGCAAAAAAGAAGCGGCGGCGGTTGCGCGGTCACGGGGGGAAGCGGGGAGACAGCCACCGGTCCCATAG

IL12β

>gi|47087194|ref|NM_213571.1| Gallus gallus interleukin 12B (natural killer cell stimulatory factor 2, cytotoxic lymphocyte maturation factor 2, p40) (IL12B), mRNA

ATGTCTCACCTGCTATTTGCCTTACTTTCATTACTTTCCTTTGCTGCCCTTCTGGAAGCACAGTGGAAACTTAGAGAGAATGTGTATGTCATAGAATCTGAGTGGAACGATGAGACACCAGCTAAAAAAGTGAAGCTCACCTGTGACACATCTGATGAAGCACTGCCAGTTTACTGGAAAAAGGGAACAGAACTGAAAGGAACTGGAAAGACTCTGACCACCGAAGTGAAGGAGTTCCCAGATGCTGGCAACTACACCTGCCTGTCTGCTAAGACCCACGAGATTATCAGCTACAGTTTCTTTCTCATAACTAAAGTAGACTCCAATGGGCAAATGATACGGTCAATTCTGAAAAGCTATAAAGAGCCAAGCAAGACGTTCTTAAAATGTGAGGCAAAGAACTACTCTGGAATTTTCACATGTTCATGGATGACAGAAAATGAGAGTCCAAGTGTGAAGTTCACAATTAGGAGCCTAAAAGGCTCTCAAGGAGATGTAACCTGCAGCAGCCCTGTGGCTCGCACTGATAAATCTGTGACTGAATACACTGCCCAGTGCCAGAAGGAAAACTACTGTCCATTTGCCGAAGAGCACCAGCCGACTGAGATGTTCCTGGAGGTCATTGATGAGGTGGAATATGAGAACTACACTAGTAGCTTCTTCATCAGAGATATCATAAAGCCAGACCCACCTCAATGTCAGTATGCAAGCACAAATGGAACTGTGACCTGGACATATCCCAAGACCTGGAGCACACCGAAGTCCTACTTCCCTTTGACTTTCAGGGTCAAAGTTGAAAGCACAAAGAAATACAAAAGCAAGGTTTATGATGCTGATGAGCAGTCTATTCAGATTCCAAAGACTGGGCCAAAAGACAAGATCTCTGTGCAGGCCAGGGATCGCTATTACAACTCATCCTGGAGTGAGTGGTCCACGCTTTGCAGATAA

IL1β

>gi|45433513:33-836 Gallus gallus interleukin 1, beta (IL1B), mRNA

ATGGCGTTCGTTCCCGACCTGGACGTGCTGGAGAGCAGCAGCCTCAGCGAAGAGACCTTCTACGGCCCCTCCTGCCTCTGCCTGCAGAAGAAGCCTCGCCTGGATTCTGAGCACACCACAGTGGACGTGCAGGTGACGGTGCGGAAGGGACGTGGTGCCCGGAGCTTTCGGCGGGCCGCCGTGCTGGTGGTGGCCATGACCAAACTGCTGCGGAGGCCGAGGAGCAGGGACTTTGCTGACAGCGACCTGAGCGCGCTCCTGGAGGAGGTTTTTGAGCCCGTCACCTTCCAGCGGCTGGAGAGCAGCTACGCCGGGGCGCCCGCCTTCCGCTACACCCGCTCACAGTCCTTCGACATCTTCGACATCAACCAGAAGTGCTTCGTGCTGGAGTCACCCACCCAGCTGGTGGCCCTGCACCTCCAGGGGCCCTCCTCCAGCCAGAAAGTGAGGCTCAACATTGCGCTGTACCGGCCCCGAGGCCCACGGGGCAGCGCTGGAACTGGGCAGATGCCAGTGGCACTGGGCATCAAGGGCTACAAGCTCTACATGTCGTGTGTGATGAGCGGCACCGAGCCCACACTGCAGCTGGAGGAAGCCGACGTCATGCGGGACATCGACAGCGTCGAGCTGACCCGCTTCATCTTCTACCGCCTGGACAGCCCGACTGAGGGCACCACGCGCTTCGAGTCGGCCGCCTTCCCCGGGTGGTTCATCTGCACCTCCCTGCAGCCCCGGCAGCCCGTGGGCATCACCAACCAACCCGACCAGGTCAACATCGCCACCTACAAGCTAAGTGGGCGCTGA

IL13RA2

>gi|114326306:104-1246 Gallus gallus interleukin 13 receptor, alpha 2 (IL13RA2), mRNA

ATGGCTCTGCAACCCATCTCCCTCCTCTCCCTGGTGTTGGCGTGGGGCTGCGTGGCTTCCTCCTCCCTCTGGCACACAGCAGTTGCTCCCCCGCAAGACCTTCAGATCACTGATCCTGGGCTTTTAGGTTCTCTCGATATAGAGTGGAAACCTCCACCCAACATACAGACCTCCAATGGATGCACAGTGAAATACAAGTGTGAATACCGTAACGCCGGCGACAGAGAATGGAAGGTTATTTTTACTAGGAAACTAAAACTCAGAGTTGGATTTGACCTCAGCAGGACTGCTGAAGTGAAGGTCCAGACCCTGCTCAAAGGATGGTGTACCGACGACGTGGAGGTCCAGAGTGAATGGCTTTATGCCACCTTTCGGGTCCCACTGCAAGGGAAACTGGAATCCGAGGTTCAGAATTTTCATTGCATCTATCACGACTGGGAATACCTCAAGTGCACGTGGCAACCAGGTCTCCTTGCTCCTCGTGGTGTAAATTATGGACTATACTATTGGTATGAGGGTCTGGAGCACACGGTGCAGTGTGACCACTATATCCAGGATCATGGTGTAAACATGGGCTGCATGCTGCAAAACCTGAGCCAGGCAGAATACAAAGACCTGAGCATCTGTGTCAATGGGTCGGTGGCAGCCACCTTGCTGAGGCCTCTGTATGCCACCCTGCGTCTTCACAACCTGGCCAAGCCCTCGCCCCCCCAGCAGCTGGTGGTCTCCATGTCTGCAGCCGAGGAGCTCCGTGTGGTGTGGAGCCCACCTGGTGGCCAAACACCACCCCAGTGCCTGGAGTACGAGGTGCAGCTGGCAGAGGAGCAGGGGGAGGCCAAGGCTGCCTGGGCGTCTGTGTCCACCCAAGTGGAGACCGCTTTCACCATCTCCAGAGCAAATCGGAGCCGTGTTTCTTGTGTCCGTGTCAGAGGGAGAACAAACAACTTCTGTGCTGATGAGGGCTTCTGGAGCGAATGGACGCAGGAGTGCTTCTCTGTATCCAGAAAAGAAGTCAAGCAGCTATTTATCCTCATTCCAGTCATCCTGGGGTTGTCAAGTAGCCTCTTAATATTTACGTTGATTGGCCAATGCAAGAAAAAATCCCCAGCAGGAAAACCATCGCACACTTTGGTGGGATGCTAA

IL18

>gi|45382948:4-600 Gallus gallus interleukin 18 (interferon-gamma-inducing factor) (IL18), mRNA

ATGAGCTGTGAAGAGATCGCTGTGTGTGCAGTACGGCTTAGAGAAAACCTCTGCCTCTATTTTGAAGATGATGAGCTGGAATGCGATGCCTTTTGTAAGGATAAAACTATCAAACGATTCTTTCGAAACGTCAATAGCCAGTTGCTTGTGGTTCGTCCAGATTTAAACGTGGCAGCTTTTGAAGATGTAACAGATCAGGAGGTGAAATCTGGCAGTGGAATGTACTTCGACATTCACTGTTACAAAACCACCGCGCCTTCAGCAGGGATGCCTGTTGCATTCAGCGTCCAGGTAGAAGATAAGAGTTACTACATGTGTTGTGAGAAAGAGCATGGGAAAATGGTTGTTCGATTTAGGGAAGGAGAAGTTCCCAAAGACATTCCTGGTGAAAGTAACATCATATTTTTCAAAAAGACATTTACATCTTGCAGCTCCAAGGCTTTTAAGTTCGAGTACTCACTTGAACAAGGAATGTTCTTGGCCTTTGAGGAAGAAGACTCCTTAAGAAAACTAATTTTAAAGAAACTGCCGAGAGAAGATGAAGTTGATGAAACCACAAAATTCGTAACAAGTCATAATGAAAGGCACAACCTATGA

IL6

>gi|45382888:23-748 Gallus gallus interleukin 6 (interferon, beta 2) (IL6), mRNA

ATGAACTTCACCGAGGGCTGCGAGGCGACGGGACGGCGGCCGGGGAGCGCCGGGAGCCGCCGCCGGAGAGCGCCCCGTCCCGGCCCCGTCGCGCTGCTGCCGCTGCTGCTGCCGCTGCTGCTGCCGCCCGCCGCCGCCGTCCCGCTGCCCGCCGCCGCGGACTCGTCCGGAGAGGTTGGGCTGGAGGAGGAGGCGGGGGCGCGGCGGGCGCTGCTCGACTGCGAGCCGCTGGCCCGGGTGCTGCGCGACCGCGCCGTCCAGCTGCAGGACGAGATGTGCAAGAAGTTCACCGTGTGCGAGAACAGCATGGAGATGCTCGTCCGGAACAACCTCAACCTGCCCAAGGTGACGGAGGAGGACGGCTGCCTGCTCGCCGGCTTCGACGAGGAGAAATGCCTGACGAAGCTCTCCAGCGGCCTGTTCGCCTTTCAGACCTACCTGGAATTCATTCAAGAGACTTTCGATAGCGAAAAGCAGAACGTCGAGTCTCTGTGCTACAGCACAAAGCACCTGGCGGCCACGATCCGGCAGATGGTGATAAATCCCGATGAAGTGGTCATCCCAGACTCGGCCGCCCAGAAATCCCTCCTCGCCAATCTGAAGTCAGATAAGGACTGGATAGAGAAAATCACCATGCACCTCATCCTCCGAGACTTTACTTCGTTTATGGAGAAGACCGTGAGGGCCGTTCGCTATTTGAAAAAGACCAGGAGTTTCAGTGCCTGA

IL8 (CXCLi2)

>gi|49169792:78-389 Gallus gallus interleukin 8-like 2 (IL8L2), mRNA

ATGAACGGCAAGCTTGGAGCTGTCCTGGCCCTCCTCCTGGTTTCAGCTGCTCTGTCGCAAGGTAGGACGCTGGTAAAGATGGGGAATGAGCTGCGGTGCCAGTGCATTAGCACTCATTCTAAGTTCATCCACCCTAAATCCATTCAAGATGTGAAGCTGACGCCAAGCGGCCCCCACTGCAAGAATGTTGAAATCATAGCTACTCTAAAGGATGGAAGAGAGGTGTGCTTGGACCCCACTGCTCCCTGGGTACAGCTGATCGTAAAGGCACTTATGGCCAAGGCTCAGCTCAATTCTGATGCACCACTGTGA

IRF7

>gi|45382446:42-1517 Gallus gallus interferon regulatory factor 7 (IRF7), mRNA

ATGGCAGCACTGGACAGCGAGGGGGACGCCCAGAAGCTGCGCTTCGGGCCATGGCTGCTGAACGCCGTCAGCAGCGGGCTGTACCGCGGCCTCTGCTGGATCGACCCGGACCGCCGTATCTTCCGCATCCCTTGGAAGCACAACGCCAGGAAGGATGTCACCAGCAGCGACGTGGAGATCTTCAAGGCCTGGGCGAAGGCCAGCGGCAGGTACGAGGGGAACGCTGAGGATCCGGCCAAATGGAAGACCAACTTCCGCTGCGCCCTGAGGAGCACTCACATGTTCATGCTGCTGGAGGACCGCTCCAAGTGCAACGACGACCCGCACAAGGTCTACGCGGTTGCCTCAGGCGTCCCCAATGACAGAGGTTCTGGGGGCCCTGTGGCAGGCGCCCTGCAACAGCAGCCGCAGCTGTTGCTCAACCACCACGATTTGGCCTTGGAAAACACTCCCACAGACAGTACTGAAGGTGTTGCTGCAGCAGCCCTGACGCAGGTGGATTTGGACCTGCTGCAGTCCGTACTGCAGCACTGCAACATCTCTGCCCTCGGCTCCCAGCCAACCCTGTGGGCACACACAGGGGATGCCTTGCCTGAGGATGCTCTGCTGCTTCCTGGCCAAGATGGCTGCCTCCCAGGGCCACAGTTTCAGGATTGGAGGCAGCTGGAGGAGCCTCTGCTGCTGGGGAACCAGCCCCTCACAGGTGGGGGCTGTGGGCAGGACGGGGCTGGGGCCCTCCCTGTGAGTGAGGAATGTGCCATCCCTGCACCATCCCCGGCTGAGGAGCTACTCTTCCAGTCTGCCAACCCCGCGCCTCCGCCACCGGCAGGTGACATAGGAGGGCTGCCCCCCCTCCTGGACATCACTATCTACTACCGAGGAAAGATGGTCTACCAGGAGCAGGTGGACGACAGCCGCTGTGTGCTGGCCTACCAGCCCCTGGACCCGGCCGTGGCCGAGCAGCGGCTGGTGCTGTTCCCCAGCCCCGCGAGCCTGCCCGACCCCAGGCAGCGGCGCTACACTGAGGACTTGCTGGAGGTGGCGGGGCTGCGGCTGGAGCAGCGTGCCGGCCAGCTCCTGGCCACGCGCCTGAAGAAGTGCAAGGTCTTCTGGGCCTTGTCGCAGCAGCTCGAGGGCGGGGAACCCCCACTCAACCTGCTCCACCGGGATCAGGAGACCACCATCTTCGACTTCAGGGTGTTTTGCACAGAGCTCCGGGACTTCCGCGACAGCCGCAGGGAGCGCTCCCCCGACTTCACCATCTTCCTCTGCTTCGGGCAGTGCTTCTCCAGCACAAAGCCCAAGGAGTCCAAGCTCATCCTGGTGAAGCTGGTTCCCCAGTTCTGCGAGTACTGGTACGAGCAGGTGCAGCGGGGAGGAGCCTCCTCCCTCAACAGTGGCAACGTCAGCCTGCAGCTCTCTGACTCTTTCAACCTCTTCGAGCTTATCGAGCAATACCACATGCAGACAGACTGA

IRG1

>gi|71895162:57-1493 Gallus gallus immunoresponsive 1 homolog (mouse) (IRG1), mRNA

ATGTGGGCAAAGACAATTACAGGAAACTTTGCCAGTGTGATTCATGGTTTGAATACAAACCACTTGACAGATGTGGTCATTCAGAGAAGCAAGCGAATGATTCTGGATACCCTTGGAGTAGGACTTCTGGGTACCAGCACCGAGGTCTGCCAGAAAGTCAAACAGTACAGCAAGATCTACAGCTCAGATATATCCAGCACCATCTGGGGCCACTTGGATTTCCGACTGCCTCCTCTGTATGCAGCTTTTGTGAATGGAGTGGCTGTGCACTCAATGGATTTCGACGACACATGGCATCCCGCCACGCACCCATCTGGGGCCGTGCTCCCTGCTGTGATGGCTCTCTCTGAGGCCTTTCCTCAGAAGAAAAAAATCTCAGGTCTTGACCTGCTCTTAGCTTTCAATGTGGGAATTGAGGTGCAAGGCAGGTTGTTACGCTTTTCCAGCGAAGCCAGGAACATTCCCAAAAGGTTTCACCCACCAACAGTGGTTGGTACAATGGGGAGTGCAGCAGCTTGCGCAAAACTGCTAGCTCTTAACCAGATGAAATGTAAAAACACCTTGGCTATTGCTGCTTCCTATGCAGGTGCCCCACTGGCTAACGCAGCAACTCAAACAAAGCCCCTCCACATTGGCAATGCTGCCAAGCATGGACTGGAAGCGGCTTGCTTAGCATCACAGGGTCTACAAGGAAACAATCAGATCTTGGACATGGAGTCAGGGATAGGTGCCTTTTATACAGATTACAACCCACAGACTCTGCCAACCTTGCAGTCCTATCCCTGGCTGTTGGACCAGCAAGATGTGGCCATCAAACGCTTTCCTGCTCATCTTGGAACGCACTGGGTGGCTGATGCAGCATCTTCTGTAAGGAGGAAGCTTGTCGAGAACAGTGGCAACTTGCTTCCCCTTGAAACAATTGAGAAGGTTATTGTAAAAGTCCCAGAGGTCAAATACGTGAACAGACCCAGCCCTAACTCAGAACATGAAGCTCGACACTCCTTCCAGTTTGTTGCCTGCTCTACGTTGCTGGATGGCAGCATGTCCATCCAGTCCTTTGCCAGTGAGAACATTCACCGGCCAGCCTTACAGGAGCTCCTCTGCAAAACACAGCTAGAGCACCCTTCAGATAACAAACCCAGCTTTGAGAGTCTTTATTGCGAAGTGACTGTTGTGCTTCGGGATGGCAACACAATTAGCGACCGCTGCAACACGTTCTATGGACACTGGAGGAAACCTCTGACAAAGGGGGACTTGGAGAAAAAATTTCAGTCCAACGCTTCTGAAGTCCTCCCTGCAGAAGCCATAGAAGGCATTATAGAGACCGTGTACAATCTGGAAAAAGTCGAGGACTGTTCTGTATTAAACACGTTTTTGTCAGGACAGTCAGCTAGATTACTTCCAGAGAAGCTGCACGTTGAACATTCCAACTCATAA

TIMD4

>gi|57524994:43-1122 Gallus gallus T-cell immunoglobulin and mucin domain containing 4 (TIMD4), mRNA

ATGTCCCACTTTGTGTTGTTTCACTGGATTGTCATACAGACCTTCATAGTGCACACCATGTCAGAAACTGTTGTTCAAGGAGTGATAGGACAACCTGTCACATTGCCTTGCTCCTACCGGGTAGCACGAGAGAAGGACATCTCCGATATGTGCTGGGGCAGAGGCCCGTGCCCAAACTCCAAGTGCAATGGCAAACTTTTGCACACCACTGGGAGCGAGGTGACGTTCAGAACATCACAGCGGTACAACCTGCAGGGCTACATTTCCTACGGAGATGTGTCTCTGACCATTCAGGAGGTGAAGGCAGAAGATGCGGGCACATACTGCTGCCGCGTGGAGATCCCAGGCTGGTTCAATGACATCAAGAAGAACATTCAGCTGGCGGTGCTCGAAGCACCTCCATTGATGACAACAACCACGGGAAAAGCTCTCACTTCCCCCAACCATTTCAGAACAACGACTTTTGCTCTCCAAGAAACTTCTGATCTTCAAGCAACCACATGGACTGCTGTCCCCACAGTAGCACCTACAACCACTGGGTCTCCCCCAACGTTTGATGAGACAACAAGTGATAACGTTATGGAAGAAGTGATGACTATCAGTGCCCTCCCAGATTTTTCAACCAATTTCGAAACAAGTGATGCATGGATTGAAGGTGAACCTGTGTTCTGCGCAACACAGCCCGAAGTGACTACTGAATTCCCAAGTATACTTTTGACTACAGACAGAACTGAAGAGGCCAACAGTTCTCTCTTGACAGATGATGTGCCAACTGTAGCAACAGCACTGCCAGAGCCAACCACACTTCAGACACCGAAGTTAACCCTTGGTCCCTCAGAGACTTCAGTGAGTTCAGACAGCAATACAGAGAAAACTGGGATGAAACTTTCCTTTCCCATCTCCACCATTCTCACCGTATCTCTCATAGGGATATCCATTATTTTAATGTTGATAGTCTTATCGTTGCTTTGGAAACGAAGACACACAAGGAAATTTATTTTAAAAAGCCTTAGACCAGCTGAAGACCTTGAAAAAGTTTTCAGTGGCTCTGAAGGAGAAAACAACCTTTTTGTGCTGTGA

TLR15

>gb|FJ915258.1|:208-2814 Gallus gallus isolate 562 TLR15 gene, complete cds

ATGAGGATCCTTATTGGGAGTCTTTATTTCTACTTCATTTCTTTCTTATTCAGTAAAGTAAATGGATTCCTAACTCAGAGAACATCTCCAGTGTCTTCCTTCCCATTTTATAACTACTCTTACTTAAACCTCTCTTCTGTATCACAAGCACAAGCTCCCAAAACAGCGAGAGCTCTCAACTTCTCATATAATGCAATTGAAAAAATAACCAAAAGAGACTTTGAAGGTTTTCATGTACTGGAAGTTTTGGACCTTTCTCACAATCACATTAAGGACATTGAACCTGGTGCATTTGAGAACCTGCTCAGCCTTGTTTCTGTGGATTTATCATTTAATGATAAGAATCTTCTGGTATCTGGTCTTGCACCTCACCTGAAACTCATACCGACCAGCGGAGCCTCGGGGCCTTCACAGATTTATATGTATTTTCAAAAATCAGCAGAGGCTGCTCTGGAGCCTTCTGCACCAGCTGAACTGCTGCCACATTTGGAAGATCCACCCAATCCAGGAAATGTTAACCCAAGATTCAGACAAAGAAGAACAGAGGAAAATAAAACTTCCCCTCCAGCAGCCACGCTGAGGCCTGACTTGTGTGGAGCACCGATCAATGGGTTGCTTGACCTATCAAGGACCAAACTGTCTAATGAAGAGCTGACAGCAAAATTGGATGCAGACCTCTGCCAAGCTCAGCTGGGCACTGTTTTGGAGTTTAACATTAGTCACAGCGACCTGGAGATGGATCTTCTATCTCTGTTCATCCTGTTTTTGCCGATGAAAGATATACAGTCCGTTGATGCCAGCTACAACAGAATAACAATTAACAACATAGACGTTGAAGCAATCTGTCACTTCCCATTCAGCAACTTTTCATTTTTAAATATCAGCAACAATCCCATCAACAGCCTGGAAACTGTGTGTCTCCCAGCAAGCATCACAGTCATTGATCTGTCCTTCACAAACATTAGTACAATACCTGCAAATTTTGCTAAGAAGTTATCTAAATTAGAACGTATGTATGTTCAAGGAAATCAGCTCATATATACCGTACGTCCAGAAAACCCCAGTGCAACCCCAAGGCCTCCCCCTGGAACTGTACAAATCAGCGCTATTTCCTTGGTCAGAAACCAGGCTGGTACACCCATCGAAAGCCTTCCAGAGAGCGTAAGACACCTGAAAGTGTCCAACTGCTCCATCGTAGAACTTCCAGAATGGTTTGCCAACAGAATGCAAGAACTACTATTTCTGGATCTCAGCAGCAATCGGATTTCCATGCTTCCTGACCTACCTATCTCTCTGCAGCAACTTGACATAAGTAACAGCGATATTAAAATAATACCCCCTAGGTTTAAATCTCTCTCCAACTTAACTGTATTTAATATTCAAAATAATAAACTTACAGAGATGCATCCCGAATACTTCCCATCCACTTTAACGACATGCGATATTAGTAAAAATAAGTTGAAGGTGCTATCATTAACCAAAGCCCTAGAGAACCTCGAATCACTTAATGTCTCTGGAAACCTAATAACCAGACTAGAACCTGCCTGCCAACTTCCTTCACTCACTAACCTGGACAGTAGTCACAACCTGATTTCAGAACTCCCTGACCATTTGGGGCAATCTCTCCTGATGCTGAAGCATTTTAATTTATCAGGGAATAAGATCTCCTTCCTGCAGCGCGGCTCCCTCCCAGCTTCCCTGGAGGAGCTGGACATTAGCGACAACGCCATCACTACCATAGTGCAGGATACTTTTGGACAGTTAACAAGTTTGAGCGTTCTGACTGTTCAAGGTAAACATTTCTTTTGTAACTGCGACTTGTACTGGTTTGTGAACATCTACATCCGTAACCCGCATTTGCAGATAAATGGCAAAGATGATCTCAGGTGCAGCTTTCCACCAGACAGACGGGGCTCGCTGGTGAAGAGCAGTAATCTCACACTTCTGCACTGCTCCCTGGGCATTCAGATGGCCATTACAGCTTGTATGGCCATCCTTGTCGTTCTGGTGCTAACGGGCTTATGCTGGCGCTTTGATGGGCTGTGGTATGTGAGAATGGGCTGGTACTGGTGTATGGCAAAGAGGAGGCAGTACAAGAAGAGGCCAGAAAATAAGCCCTTCGATGCCTTCATTTCATATAGCGAGCACGATGCAGACTGGACGAAGGAGCATCTACTGAAAAAACTGGAAACTGATGGATTCAAGATATGTTATCATGAGAGAGATTTCAAACCAGGGCATCCTGTACTTGGCAACATTTTTTACTGCATAGAGAACAGCCACAAAGTCCTTTTTGTTCTCTCTCCCAGTTTTGTAAATAGCTGCTGGTGTCAGTATGAACTGTATTTTGCTGAACACCGGGTCCTGGATGAAAATCAGGATTCCCTCATTATGGTAGTGCTGGAAGACCTCCCGCCCGACAGCGTGCCTCAGAAGTTCAGCAAACTCAGGAAGCTGCTGAAAAGAAAAACCTACTTAAAGTGGAGCCCCGAAGAACACAAACAGAAGATTTTCTGGCATCAGCTGGCAGCTGTCCTAAAAACAACCAATGAACCACTGGTGAGAGCAGAAAATGGACCCAATGAGGATGTAATTGAGATGGAATGA

TLR4

>gi|71897340:1-2532 Gallus gallus toll-like receptor 4 (TLR4), mRNA

ATGCCCAGCAGAGCGGCTCCCACCGCGCTGACTCTTGGGGTGCTGCTGCAGCTGCTGCTCGTGCTGTCCCTGCTGGCAGGATGCATCCCCAGTCCGTGCCTGGAGGTCATCCCCAGCACAGCTTTCAGATGCACAGGACAGAACATCTCTGGAGTTCCTGCTGAAATCCCAAACACCACCCTGGACTTGGACCTCAGTTTCAACAGTCTGAAATTGCTGAGCTCAAATTATTTTTCATCAGTACCCGAACTGCAGTTTCTGGATCTTTCAAGGTGCCACATCCATACAATAGAAGATAACTCTTTTGTGGATCTTTATAACCTTTCCACCTTAATTTTAACTGCCAATTCACTCCAGCACCTGGGTCTAGCAGCCTTCCATGGCTTAACGTCGCTGAAAAAACTAGTACTGGTGGAAACAAGCATATCCTCTCTGTCTGACCTACCCATCGGACACTTGAATACCCTGCAGGAGCTGAATCTGGGCCATAACAACATTGCTTCATTGAAGCTTCCTAAGTATTTTGCCAACCTGACCTCTCTCAGGCACCTGAGCTTTTCCTCCAATAATATTACATATATCTCCAAAGGAGACCTTGATGCCCTGAGGGAAACAAACAGGCTCAACCTCACGTTGGTACTTTCTCTGAACAATATAAAATACATCCAGTCAGGATCCTTTGCAAAGATTCACCTTGGTGAACTGATTCTGAGATCCTCTTTTGAGAACCTCAATGCGATGCACTCTTCTCTTCAGGGCCTGGCAGGTTTACAGGTCAACAGACTAATAGTTGGAGAATTCACTAACATTCTGAAAATAACGGCATTTCAGAACGGACTCTTGAGTGGACTGTGTCAGGTACAGATGCAGGAGTTTGTCTTAATGTGTTTCAGAGAGTTTGAGAATGACACGGACACTCTTTTTGACTGCATAGGCAACGTCACCACTATTCGGTTGGTGGACCTGAATCTTGAAACGTTGTCAGAGGTTCCTATGTTTTCTCAAGTGAAACATCTGGAATGGAAGAGATGTAAGTTTCAGGAATTGCCTGCTGAGAAGCTGTCTCTTTTTAAGGAGTTGAGAGTGCTTCGTATTACCAAGAGCAAAGACCTAAATGGCTTCGAGCAAAAGTTTGGGAGTCTAACTTACCTGGAGGTTGTAGATTTGAGTGAGAATCGTCTCTCCTTCCTTACCTGCTGTTCCCCTAAGTTTCCCAGGTCTCCAAATTTGAAACACTTGAATCTAAGCTTCAATTCTGACATCAGTTTGACTGGAGAATTTGCTAATCTGAGAAATCTGCTATACTTGGACCTTCAACACACAAAGCTAATTCATCATGGCACCTACCCTGTCTTTCTCCTCCTTCAGAAACTCATTTACCTTGATATCTCCTACACCAAAACTCATGTTATGTCCCATCTCATCTTTCATGGCTTGAACTCTTTGCAAGTGCTGAAGATGGCAGGCAACTCCTTTGAGAACAATACATTGACCAACAACTTTGAAAATGTAAGGCGGCTCCGCATCTTGGATATTTCAAGTTGCAAATTAGTATGGGTGGACCAAAGTACATTTAATGCTCTCTCTGAACTAAAAGAGCTGATCATCAGCAACAACAAGCTATTGACTTTTGATCCTGTAACATACAAGCCACTCCAAGCCCTCACAGCTCTGGATTTCAGCAACAACCAGATGAGTTTCCTGTCGGACTCAGCCCTGGAAATCCTGCCTGACAGTCTGGTCTTGCTAGACATTTCTCACAACTTGTTTGAATGCTCTTGCACACATTTGAACTTCCTGAAATGGGTCAAGGAAAAGCAGGATCTACTGCAGAACAAGCATTCAATGATATGCCACACACCTGCCTACATGAAGAACATGAGCCTGTCAAACTTTGATATGTCCTCTTGCCATCCCAACCCAACCACAGTAGCATGCTCAGTGACTGTGTTGCTTGCTGCAGGAGTGTTTCTATTCCTCATTTATAAGTACTACTTCCAGCTATACTACTCATTGGTGCTGCTCAGTGGGTGTAAACACTCTGCAGAAAGGGGAGATATCTATGATGCCTTTGTTATCCATTCCAGCAAAGACCAAGAATGGGTGATGAAAGAGCTGGTGGAACCCTTAGAAGAAGGAAAACCTCCCTTCCAGCTTTGTCTTTACTTCAGGGATTTTTTACCAGGGGTACCCATTGTCACTAATATAATCCAAGAAGGTTTTCTGAGTAGCAGAAATGTCATTGCAGTCATCTCAGCTGACTTTCTGGAAAGCAAGTGGTGTAGCTTTGAGTTTGACATTGCTCGGTCCTGGCAGCTTGTTGAAGGGAAGGCTGGAATAATCATGATCATCCTAGGGGAAGTGGATAAGACCTTGCTGAGGCAGAGGCTGGGACTGTCCCGATATCTGAGGAGGAACACTTATCTGGAGTGGAAAAACAAGGAAATAAGCAGGCATATCTTCTGGAGGCAGCTGACATCAGTCCTGCTAGAAGGCAAAAAATGGAATCACGAGGAGATAAAACTCATGTAA
